# Environmental evaluation of plastic waste end-of-life pathways in Malang District, Indonesia: a life cycle approach

**DOI:** 10.1007/s11367-026-02751-9

**Published:** 2026-09-29

**Authors:** Alexandra Olid Stepanchuk, Sekar Aqila Salsabilla, Serius Miliyani Dwi Putri, Mochamad Chazienul Ulum, Sujarwoto Sujarwoto, Jeremy Woods, Kristine Belesova, Christopher Millett, Onesmus Mwabonje

**Affiliations:** 1 Centre for Environmental Policy, Faculty of Natural Sciences, Imperial College London, 16-18 Princes Gardens, London, SW7 1NE UK; 2 Department of Public Administration, Faculty of Administration, University of Brawijaya, JI.MT. Haryono No.163, Ketawanggede, Kec.Lowokwaru, Kota Malang, Jawa Timur 65145 Indonesia; 3 Faculty of Medicine, Universitas Brawijaya-Climate and Health Centre, Ketawanggede, Kec.Lowokwaru, Kota Malang, Jawa Timur 65145 Indonesia; 4 School of Public Health, Faculty of Medicine, Imperial College London, 86 Wood Lane, London, W12 0BZ UK

**Keywords:** Environmental impact, Plastic waste management, Air pollution, Sustainability, Mismanaged plastics, Indonesia, Developing countries

## Abstract

**Purpose:**

Malang is an Indonesian District struggling with waste management: low collection rates, fly-tipping, and burning. This study conducts a Life Cycle Assessment (LCA) on the end-of-life pathways of plastic waste (PW) in Malang. All the stages of the PW management system are covered, from PW generation and transportation to unregulated and regulated PW management strategies. Ultimately, the findings aim to support policymakers in understanding the environmental impact of PW management and how to mitigate it.

**Methods:**

Stakeholder interviews provided primary data, which informed the Life Cycle Inventory (LCI) across all stages of the PW management system in villages of Malang District. The data was then adapted into the functional unit (FU) of 1 kg of PW generated and statistically analysed to understand PW end-of-life trends. The LCA was conducted using the open-source software Activity Browser. In this case, the ReCiPe v1.03 midpoint impact categories considered were global warming, primary energy depletion, and particulate matter formation. The results of the analysed villages were compared with the national average. The PW from four villages (Bakalan, Krebet Senggrong, Krebet, and Tlogorejo) were modelled under three end-of-life scenarios i.e., landfilling, recycling, and incineration with energy recovery to inform intervention selection.

**Results and discussion:**

In the baseline scenarios of the villages, the highest impact in Global Warming Potential, Fossil Fuel Potential, and Particulate Matter Formation Potential (GWP100, FFP, and PMFP, respectively) was linked to higher rates of open burning (e.g., Krebet Senggrong where 35% of waste is open burned). Villages with the highest recycling rates however present lower GWP100 and FFP, such as the case of Bedali and Mangunrejo, where 16.6% and 14.3% of plastic is recycled, respectively. Low PMFP is tied to options such as landfilling, which occurred in Rekasan. The alternative intervention strategy with the lowest environmental impact is recycling (when considering avoided plastic production, GWP100 and FFP are negative). However, it is the alternative with the highest PMFP, even surpassing the baseline scenario.

**Conclusion:**

Open burning remains one of the most prevalent PW management strategies in Malang, which is not only detrimental to the environment but also proven to negatively impact human health, particularly respiratory health. It is essential not only to increase waste collection and treatment rates, but also to ensure that waste‑management practices meet environmental standards and minimise adverse impacts on human health and the environment.

**Supplementary Information:**

The online version contains supplementary material available at https://doi.org/10.1007/s11367-026-02751-9.

## Introduction

Plastics are a revolutionary material that have been tailored to many applications since their discovery in the 20th century (Davis 2022). The main reasons why plastics are so versatile are because of their properties, such as low density, durability, and low cost of production (Bergmann et al. 2015). Because of its popularity, waste management systems struggle to keep up with the rate of plastic production, use, and disposal. In 2021, Meijer et al. found that archipelago countries, like the Dominican Republic and Indonesia, are most affected by this, with a resulting higher ratio of discarded plastic (Meijer et al. 2021). Plastic waste (PW) poses significant environmental hazards, such as soil degradation, groundwater contamination, and harm to the marine environment, which is not only detrimental to marine ecosystems but also to human health (Fayshal 2024). This is due to low waste collection rates, at both formal and informal level, and a prevalence of waste management practices such as open dumping (both on land and waterways) (Veiga et al. 2023). It was estimated that approximately 7.8 million tonnes of PW are generated annually in Indonesia, and less than 3 million tonnes are managed appropriately, which is around 43.8% (Soliman et al. 2025; Veiga et al. 2023).

Rural Areas are responsible for two-thirds of mismanaged PW due to a lack of access to infrastructure and logistical services (Veiga et al. 2023; World Bank Group 2021). Some of the most common methods Indonesian households use to manage waste are open dumping and open burning (Brotosusilo and Naldi 2021; Ramadan et al. 2023). The latter is particularly concerning as it releases a large amount of greenhouse gases (GHG) and particulate matter (PM) into the atmosphere, which negatively impacts human health (Pathak et al. 2024). Air pollution is estimated to account for a significant proportion of national morbidity and mortality (Haryanto 2018). Another consideration within the Indonesian waste management landscape is the country’s significant role as a global importer of PW until the beginning of 2025, having received approximately 262.9 thousand tonnes in 2024 (International Union for Conservation of Nature Netherlands 2025; NIHR, 2024). Western countries chose to export their waste to countries with weaker oversight to avoid dealing with the repercussions that this waste has on the environment and the costs of responsible disposal (Gündoğdu 2024).

Over recent decades, policy initiatives and legislative measures have increasingly aimed to reduce or replace plastics in light of their documented environmental and human health impacts. Indonesia has introduced increasingly stringent regulations on plastic waste imports, with recent policy directions indicating a move toward reducing and potentially phasing out such imports. Law No.18/2008 on Waste Management and Presidential Decree No.97/2017 on National Policy & Strategy on Management of Household Waste and Household-like Waste Act, outline a national strategy to reduce waste by 30% and to handle appropriately the other 70% (Ministry of State Secretariat 2017; President of Indonesia 2008). An additional policy direction reflecting the government’s commitment to these goals is the planned phase-out of PW imports by 2025, supported by regulatory instruments such as Ministry of Trade Regulation No. 31/2016 and its amendments (Pramudita 2024; Raba 2025). While this regulation does not explicitly mandate a full ban, it establishes strict quality controls and requirements for imported non-hazardous waste, including plastic scrap. However, policy measures alone may be insufficient; several studies show that focusing on reducing plastic consumption through encouraging reuse and the appropriate disposal and recycling of PW is a more effective approach to tackling this issue (Meng et al. 2024; Ministry of State Secretariat 2017). Since waste management is highly decentralised in Indonesia, villages play an essential role in organising waste management and initiatives. Law No. 6 of 2014 grants villages the right and responsibility to manage their own governance, development, and community affairs based on local initiatives and priorities. Their authority is further supported by Law No. 18 of 2008 on Solid Waste Management, which emphasizes the role of local governments, including village administrations, in implementing community-based waste management systems (UNEP 2014). At the local level, waste management in Malang is implemented through district and village authorities, in line with Indonesia’s decentralisation policy and Law No. 6/2014 on Village Governance, which grants villages autonomy to manage community-based waste systems. In practice, this has led to a mix of formal and informal waste management approaches, including municipal collection services, landfill use, and community-based initiatives such as waste banks (*bank sampah*). However, as highlighted in this study, limited infrastructure and service coverage, particularly in rural areas, result in continued reliance on informal practices such as open burning and dumping. Overall, there are many stakeholders, both globally and in Indonesia, that are interested in understanding the environmental impact of PW and the different treatment technologies that make up the PW management system. According to the review by Alhazami et al. in 2021, many LCA studies have covered the end of life (EoL) of specific plastic products post-consumer (such as PET bottles), the recycling system as a whole in the context of a country (namely European and Asian countries including Indonesia), and the impact of the entire PW system with the EoL mix in mind.

Neo et al. (2021) conducted an LCA at the national scale using secondary data in India and Indonesia. Goh et al. (2025) LCA is even broader, focusing on Southeast Asia and covering data on the four most popular plastics (i.e., HDPE, LDPE, PET, and PP). Few studies have addressed the entire PW management system in Indonesia, and none have investigated a specific subnational context such as Malang District, which has experienced rapid economic and demographic growth. Consequently, it is struggling to manage the increased rate of waste generation, which forces the population to manage waste with practices such as open dumping and burning. This study aims to provide data and results that are specific and tailored to the context of Malang District. The National Institute of Health Research and University of Brawijaya are conducting research and working closely with the community to tackle air pollution related to plastic burning and community capacity building, as well as strengthening primary healthcare in the region (NIHR 2024). This reflects ongoing international research collaboration supporting local capacity building and evidence generation for waste management and air pollution mitigation in Malang District. Limited data exists on PW management, and material flows in Malang District, constraining the accuracy of system‑level impact assessments and underscoring the value of the insights derived from this study. This study collected primary data to better understand the flow of PW and the associated emissions and impacts on the environment and health.

This paper aims to assess the environmental implications of the existing PW management system in Malang District and to compare alternative intervention strategies (i.e., recycling, incineration with energy recovery, and landfilling) to identify those that minimise adverse impacts on both the environment and human health. The LCA is based on primary data provided by selected stakeholders from villages in Malang District, an approach which has not been used before for the same aim prior to this study. This is evidenced by the lack of LCA articles assessing PW management in the Malang District. The collected primary data is compiled into a Life Cycle Inventory (LCI) to support an LCA of all the EoL pathways. To better understand the effects of data uncertainty a sensitivity analysis is conducted. Additionally, the LCI and expanded set of results have been provided in the Supplementary Materials for transparency and reproducibility. The results and recommendations will inform interventions that will be implemented by the University of Brawijaya and the National Institute for Health and Care Research.

## Methodology

This study applies an LCA framework in accordance with ISO 14,040/44 standards for PW management in Indonesia (ISO 2006). These standards have been acknowledged by the Indonesian government and integrated within mandatory schemes (e.g., SKEM, ISPO, and PROPER). To ensure methodological rigour the study also followed the best practice guidance from the ILCD Handbook (European Commission 2010; Wiloso et al. 2018). The phases outlined in the ISO 14,040 framework ensure a systematic and transparent approach to evaluating the environmental impacts associated with products or systems throughout their life cycle.

Primary data was collected through semi-structured surveys administered to the relevant stakeholders who are involved in PW management in Malang District. In the cases where data gaps existed, secondary data was obtained from publicly available sources and the Ecoinvent database to complete the LCI. An example would be PW that was collected from a stakeholder, but then the collection or waste management allocations did not account for all the waste. This happened in villages with facilities that produced large amounts of waste, and when interviewing households.

Methods tied to air pollution were prioritised as open burning is one of the most prevalent PW management methods, and it is directly linked to respiratory illness upon the inhalation of PM. The LCA was executed via Activity Browser, an open-source software, to facilitate transparent modelling and reproducibility (Steubing et al. 2020). This software was selected as a commitment to the study to open science and to allow future validation and comparative analysis.

### Goal and scope

This study aims to quantify the impact of the baseline environmental impact of PW EoL pathways in Indonesia and to compare alternative management practices - recycling, incineration with energy recovery, and landfilling- to identify the most sustainable interventions (see Figure S2).

The functional unit defined for this study is 1 kg of PW generated, which serves as the reference flow for developing the LCI. The composition of the PW is based on proportional data obtained from primary stakeholder responses in each selected village (Alhazmi et al. 2021). The PW can be broken down into high- and low-density polyethylene (HDPE and LDPE, respectively), polypropylene (PP), polyethylene terephthalate (PET), polystyrene (PS), and expanded polystyrene (EPS) (see Figure S1). The EoL options of the PW considered in the LCA study were open burning, open dumping (on land and in water bodies), sanitary landfilling, recycling, and waste incineration with energy recovery. All practices were identified during data collection, except for incineration, which was proposed by the authors as an alternative for unrecyclable and undesirable waste from which energy can be recovered (Voss et al. 2022).

**Fig. 1:**
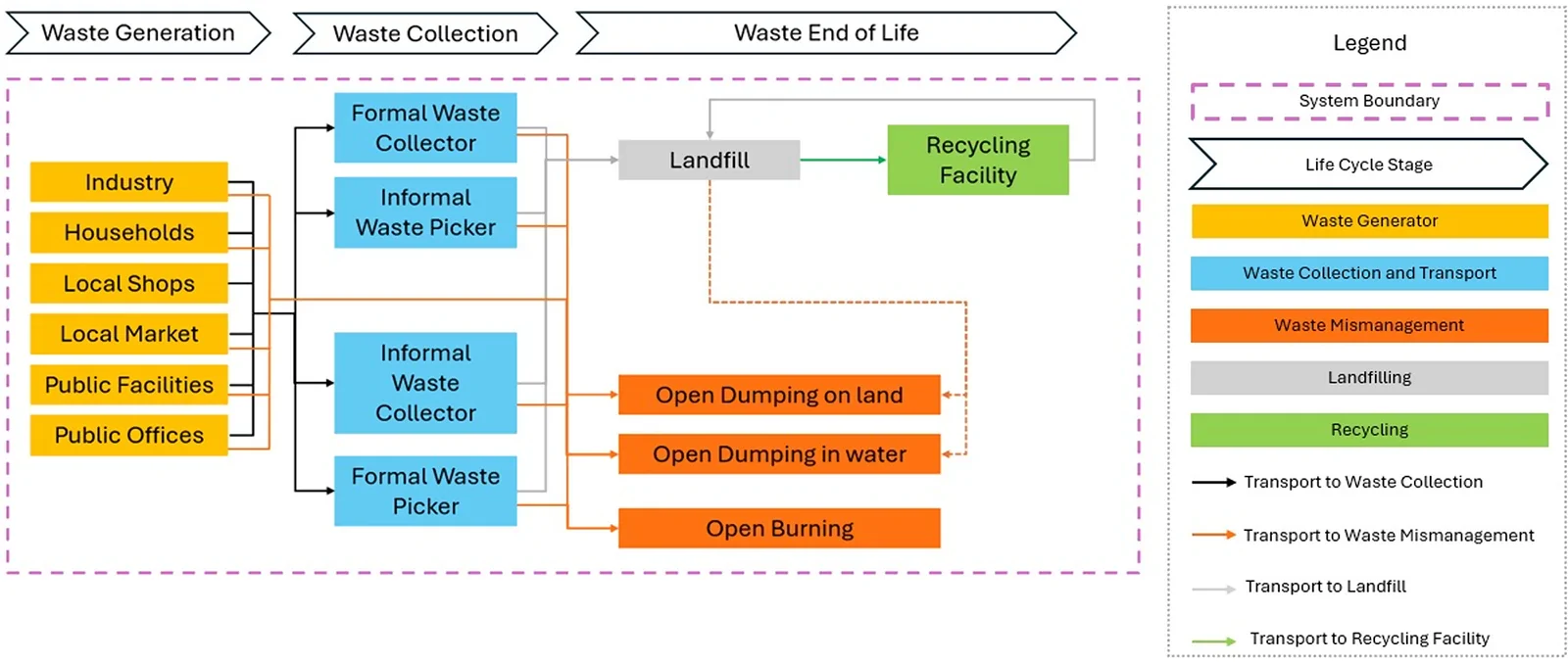
System boundaries for plastic waste management in Malang District

In this study, upstream processes, such as raw material extraction and plastic production, are excluded from the system boundary, allowing for a focused evaluation of PW management practices and their associated environmental impacts. Figure 1 presents the system boundaries of this study and includes avoided burdens, accounting for the displacement of virgin plastic production through recycling, thermal energy generation from PW being used as fuel, and electricity production offset by incineration with energy recovery.

### Life cycle inventory development

#### Study area and stakeholder interview approach

The study area is situated in the District of Malang, which is part of the Indonesian Province of East Java. Because villages have the sovereignty to manage their own waste and make decisions about it, the study focused on a sample of 11 villages selected to reflect diverse waste management conditions. Initially, 6 villages were chosen because they had widespread practices of unregulated waste disposal, particularly open dumping and burning of household waste. This selection was based on preliminary observations and local reports (International Union for Conservation of Nature Netherlands 2025; Latchem-Hastings 2020). The absence of formal waste management infrastructure (e.g., landfills, incinerators, recycling facilities, and public waste collectors and pickers) in these areas has led residents to manage their waste independently, often through environmentally harmful practices (Butarbutar et al. 2024; Meidiana and Gamse 2010). The rural study area selection reflects a broader trend in peripheral regions of Indonesia, where limited institutional capacity and lack of service provision have resulted in self-managed waste handling systems (Brotosusilo and Naldi 2021). These informal systems are shaped not only by local behaviours but also by broader social and economic networks related to waste flows, including informal recycling, resale, and disposal. Of the eleven villages, six (Bakalan, Krebet and Krebet Senggrong in Bululawang sub-district, and Pagak, Sumberejo and Tlogorejo in Pagak sub-district) were characterised through forty-household weighing audits each, while the remaining five entered the inventory through snowball tracing of waste flows and are represented by between one and three facility-level respondents — recycling industries in Bedali and Mangunrejo and a landfill in Rekesan — that receive plastic waste generated elsewhere in the district. The polymer compositions reported for these five villages are therefore properties of the individual facilities rather than village-level characterisations, and Table 1 reports the number of respondents by stakeholder group.

**Table 1:** Stakeholder information

Stakeholder	Number of Stakeholders Interviewed	Topics covered through the survey
Households	240	Types and amounts of waste generated, Types and amounts of plastic waste generated, Waste management methods
Public Office	6	Types and amounts of waste generated, Types and amounts of plastic waste generated, Waste management methods
Public Facilities	12	Types and amounts of waste generated, Types and amounts of plastic waste generated, Waste management methods
Local Shop	6	Types and amounts of waste generated, Types and amounts of plastic waste generated, Waste management methods
Market	2	Types and amounts of waste generated, Types and amounts of plastic waste generated, Waste management methods
Industry	4	Types and amounts of waste generated, Types and amounts of plastic waste generated, Waste management methods
Informal Waste Picker	9	Waste collected, Types of plastic waste collected and amounts, Transport methods and distances
Informal Waste Collector	2	Waste collected, Types of plastic waste collected and amounts, Transport methods and distances
Formal Waste Picker	4	Waste collected, Types of plastic waste collected and amounts, Transport methods and distances
Formal Waste Collector	2	Waste collected, Types of plastic waste collected and amounts, Transport methods and distances
Recycling Industry	3	Types of waste recycled, efficiency, and methods, Waste generated
Landfill	1	Types of waste landfilled, efficiency, and methods, Waste generated

To explore these dynamics, the study employed a snowball sampling method, which led to a final sample of 11 villages and allowed for all identified stakeholders to be interviewed (Parker et al. 2019). The use of snowball sampling also enabled the tracing of interconnected waste flows beyond administrative village boundaries, ensuring that the system representation reflects the broader waste management network operating at the district level. Furthermore, the inclusion of multiple stakeholder groups (*n* = 291), including households, informal waste collectors, market actors, and public service providers, strengthens the robustness of the system-level understanding. As such, the findings provide transferable insights into similar rural and peri-urban contexts within Malang District, rather than a statistically generalisable estimate. Initial respondents were identified within the 6 initially selected villages, including household heads, salespeople in local markets, staff from local industries, and community leaders responsible for maintaining public facilities and offices. When respondents indicated that their waste was transported, sold, or transferred to locations outside their village, such as to middlemen, recycling hubs, or informal aggregation points, researchers followed these leads to additional sites (Neale 2019). In this way, the research traced the actual movement of waste through its informal supply chain, rather than limiting data collection to administratively defined boundaries. This approach allowed for a more comprehensive mapping of the local waste ecosystem, including stakeholders who operate beyond the immediate village context but remain integral to the system. Importantly, this approach acknowledges that waste management in rural areas often relies on dispersed, informal networks that do not align with official jurisdictional divisions (Harfadli et al. 2025). The data could also be separated by stakeholder groups to offer a different perspective of the waste management system in Malang District and are included in the supplementary data (Sect. 3 of Supplementary Information).

To assess the uncertainty that is tied to the primary data, a sensitivity analysis was executed. It was conducted on the following parameters to understand the effects that the variations would have on the LCA results. The parameters assessed were waste collection rates, open burning rates, the rates of recycled PW, and the amount of virgin plastic production avoided by recycling. The reason why these parameters where selected is because these activities have a direct and notable impact on the results and the data on these were seen to have the most uncertainty as per the statements of the interviewed stakeholders.

For each of the parameters (i.e., waste collection rates, open burning rates, the rates of recycled PW, and the amount of virgin plastic production avoided by recycling), the inputs were varied by ± 10%. Although transport is another relevant parameter, it is shown in the baseline scenario that it has a relatively low impact.

Then, to assess potential interventions, the villages of Bakalan, Krebet Senggrong, and Tlogorejo were selected because, at the time of writing, these were the villages in which waste bank cells had already been established under the ongoing CARE intervention (Kusumo 2025). This shows that there is already an interest in the community and that active participation (e.g., some locals are receiving training to work in waste cell banks). The interventions presented in this study are applied to the PW that was extracted from the primary data collected. However, the interventions selected (i.e., recycling, incineration, and sanitary landfilling) were proposed by the authors, based on the current interventions and other technical interventions that could reduce the environmental impact of the waste management system.

#### Data collection

The LCI data for the study was obtained through stakeholder surveys from the selected villages in Malang District (see Tables S1 to S12 and Sect. 4. Surveys in Supplementary Information). In each village, interviews were conducted with a purposively selected group of stakeholders by locally based researchers and trained enumerators. The stakeholder groups included representatives from sectors involved in PW management, namely waste collectors, public area officers, household members, informal market-based waste pickers, public facility personnel, and industrial entities engaged in the production, processing, or disposal of PW (see Table 1 for a more detailed breakdown). At household nodes, quantities were obtained through a seven-day waste characterisation audit rather than by recall. Enumerators visited each participating household on seven consecutive days (13–19 January 2025); the enumerator demonstrated the segregation and weighing procedure at the first visit, and the segregated plastic fractions were weighed and recorded to the nearest gram at each visit, with the date and the start and finish time of every visit logged. Respondents were not asked to identify polymer types: the instrument presented plastics as everyday product categories (for example jugs, buckets and shampoo bottles for HDPE; grocery bags, sandwich bags and squeezable bottles for LDPE; yogurt tubs, cups and rope for PP; and water and soft-drink bottles for PET), the corresponding resin code (HDPE, LDPE, PP, PS, EPS, PVC or ‘Other’) being pre-printed against each category in the instrument. Each record identifies a named enumerator and a named editor, and the instrument additionally provides for editor review and local-supervisor verification. At market, local shop, public office, public facility, collector and picker nodes, waste flows were recorded over an equivalent seven-day period. At industrial nodes the daily weighing was carried out by the operators themselves rather than by enumerators, as the sites did not permit daily access to the research team; these quantities are therefore self-measured, and are identified as such in the Supplementary Information. The landfill node is recorded differently again: the operator reported a facility-level daily throughput of approximately 250 tonnes, of which around half was plastic, rather than a seven-day audit of segregated fractions. This is a rounded operator estimate and is identified as such (see Tables S1 to S12).

**Fig. 2:**
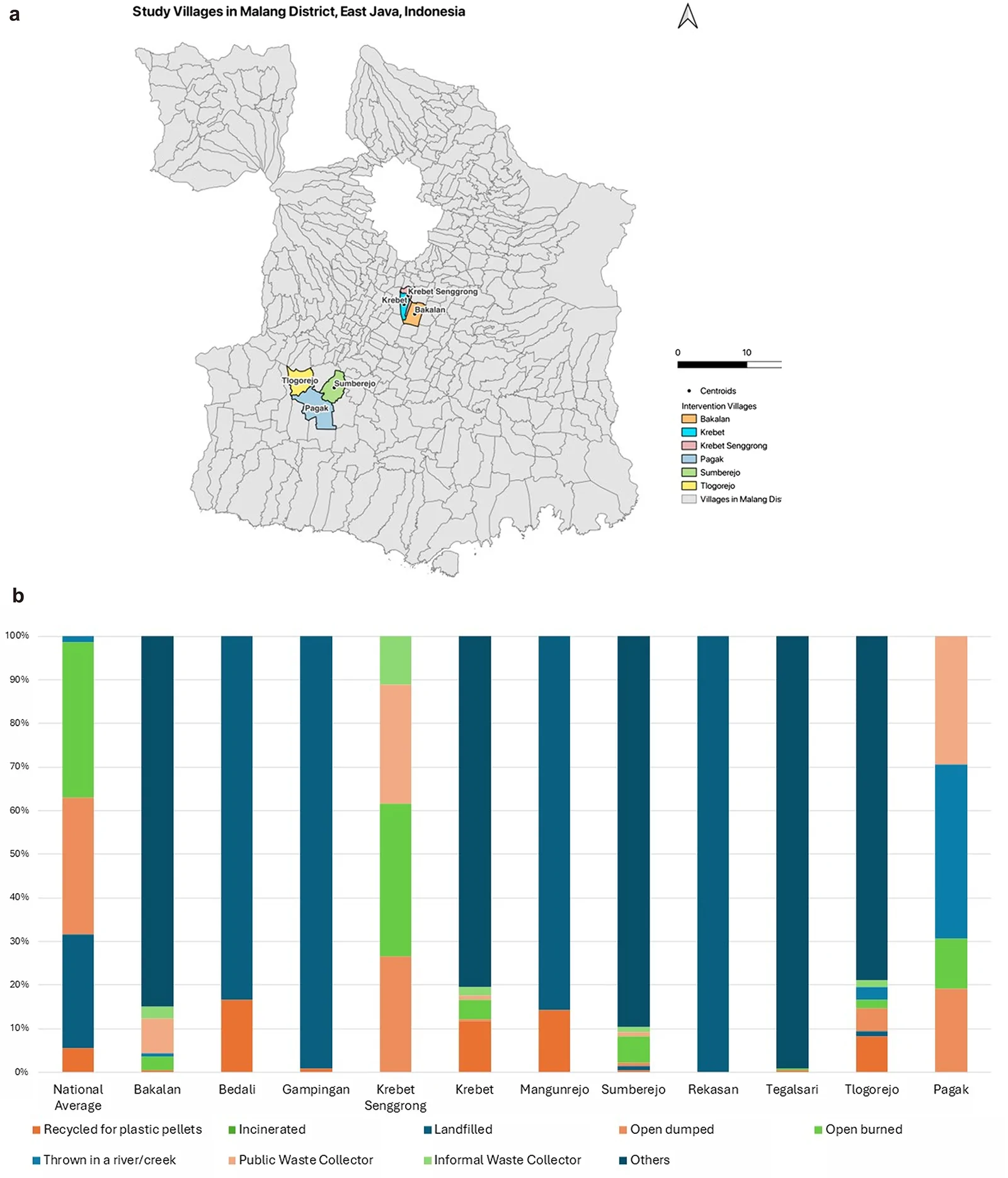
(**a**) Map of Malang District in East Java, Indonesia. (**b**) Plastic end-of-life treatments of the baseline scenario villages

Survey data were transcribed into an Excel spreadsheet for subsequent analysis as part of the Environmental Impact Assessment. As the number of stakeholders was 291 interviewees in total, the stakeholders were grouped by villages. This allowed to get more granular insights within the district and have a more manageable data set. The analysis consisted of separating stakeholders by village and adapting the data to the functional unit of 1 kg of PW generated. From this data, it was also possible to calculate the proportion of different types of plastics and mass allocation in the case where a process resulted in several products (Fig. 2). Then, data undergoes statistical analysis to understand the average amount of plastic (divided by type) produced, transported and treated in a day, as well as the standard deviation to gain insight into the uncertainties which then will appear in the results. To attain the functional unit of 1 kg of plastic generated, the stakeholders where grouped by village and then the total waste was grouped in plastic type and EoL and divided by the total waste in the village to have the values necessary for the LCI.

Primary data on PW EoL (e.g., composition of PW) was then triangulated with official reports released by governmental agencies (Flick 2020; Ministry of Environment and Forestry 2020). The data related to the type of transport and transport distance between stakeholders along the supply chain. Transport distances were taken from the primary data collected from stakeholders via surveys. For these distances, it was assumed that lorries with a capacity of 7.5 to 16 tonnes and with Euro IV emission standards, or with cars and motorcycles, when this was specified in the interviews (Indonesia | Transport Policy 2025). An Indonesia-specific electricity mix was applied to enhance the geographical relevance and accuracy of the life cycle inventory data. LCI data related to avoided burdens was primarily derived from stakeholder interviews, with supplementary validation conducted using official government reports. In the case of recycling, the replacement rate was assumed to be 50%, similar to Neo et al. (2021), in order to account for the material losses from the recycling process. Replacement rate in this instance is defined, using the same definition as in the case of Neo et al., as the amount of recycled plastic mass that replaces/displaces virgin plastics (see Tables S1 to S12 in Supplementary Information). The replacement rate was extracted from Neo et al., as this study was similar in its objectives and set in Indonesia but at the national scale (Neo et al. 2021). Heat and electricity data were directly sourced from the Ecoinvent version 3.10 inventory (Doka 2017).

Because of the complexity of the PW management system and data uncertainty, certain assumptions were made when PW EoL was not specified. In these instances, the average EoL scenario was applied to the remaining waste, where 36% is open burned, 25% is open dumped, 24.5% is sanitary landfilled, 5.6% is recycled, and the rest leaks into terrestrial ecosystems and bodies of water. This was applied to EoL that was categorised as ‘Other’ in surveys (see Sect. 4 of Supplementary Information), and the reference data was sourced from the Ministry of Environment and Forestry report (Ministry of Environment and Forestry 2020). This way, an estimate can be provided for what treatments the plastic undergoes, which is specific to the county at the very least. Similarly, for plastic types that were not specified, the Ecoinvent activities for ‘PW mixture’ were used to provide an estimate of PW that might undergo each EoL activity. Another assumption was that the PW recycling process was different from the one that appears in the Ecoinvent v3.10 cutoff database because it only has activities based on recycling factories located in Europe. Neo et al. (2021) provided activities that are closer to the Indonesian PW recycling sector, as well as estimates for the electricity and heat recovered in the incineration (Ministry of Environment and Forestry 2020). Whenever possible, entries specific to Indonesia, but for most of the information, specific entries were not available, so in these cases, ‘RoW’ and ‘Global’ entries were used. See Tables S1 to S12 in Supplementary Information for detailed LCI and the included Ecoinvent activities (from version 3.10 cutoff).

### Impact assessment methods

Life cycle impact assessment was performed using the ReCiPe v1.03 midpoint (H) methodology. The subsequent results facilitated a more nuanced understanding of trade-offs among the selected midpoint impact categories. Global Warming Potential (GWP100) was selected as an impact category due to its relevance in quantifying GHG emissions, which are a critical indicator of the system’s contribution to climate change. Primary energy depletion was assessed using Fossil Fuel Potential (FFP), defined as the ratio between the higher heating value of a fossil fuel resource and the energy content of crude oil, thereby reflecting the system’s reliance on non-renewable energy sources. Particulate Matter Formation Potential (PMFP) was included to capture variations in ambient particulate concentrations, which are associated with adverse cardiopulmonary health outcomes upon inhalation(Huijbregts et al. 2017) Particulate Matter was selected over other impact categories due to air pollution being a large public health concern in Indonesia, as it is estimated that 123,000 annual deaths in Indonesia are associated with air pollution (Susanto 2020).

## Results

The results of this study are presented in two sections. The first section comprises a hotspot analysis, which quantifies the environmental impacts associated with the management of one kilogram of PW across the villages where primary data were collected. These results were compared with a national average derived from a report published by the Indonesian Ministry of Environment and Forestry in 2020, enabling a contextualized assessment of regional variations. These results contribute to identifying the hotspots within each village and comparing villages to see which practices have a higher environmental impact. The second section, termed the intervention analysis, compares the current environmental performance of selected villages with alternative waste management scenarios, including sanitary landfilling, recycling, and incineration with energy recovery. The results offer insights into potential strategies for reducing the environmental impacts of existing waste management practices and give insight into what alternative management practices are most sustainable.

### Hotspot analysis of baseline scenarios

#### Climate change impact

**Fig. 3:**
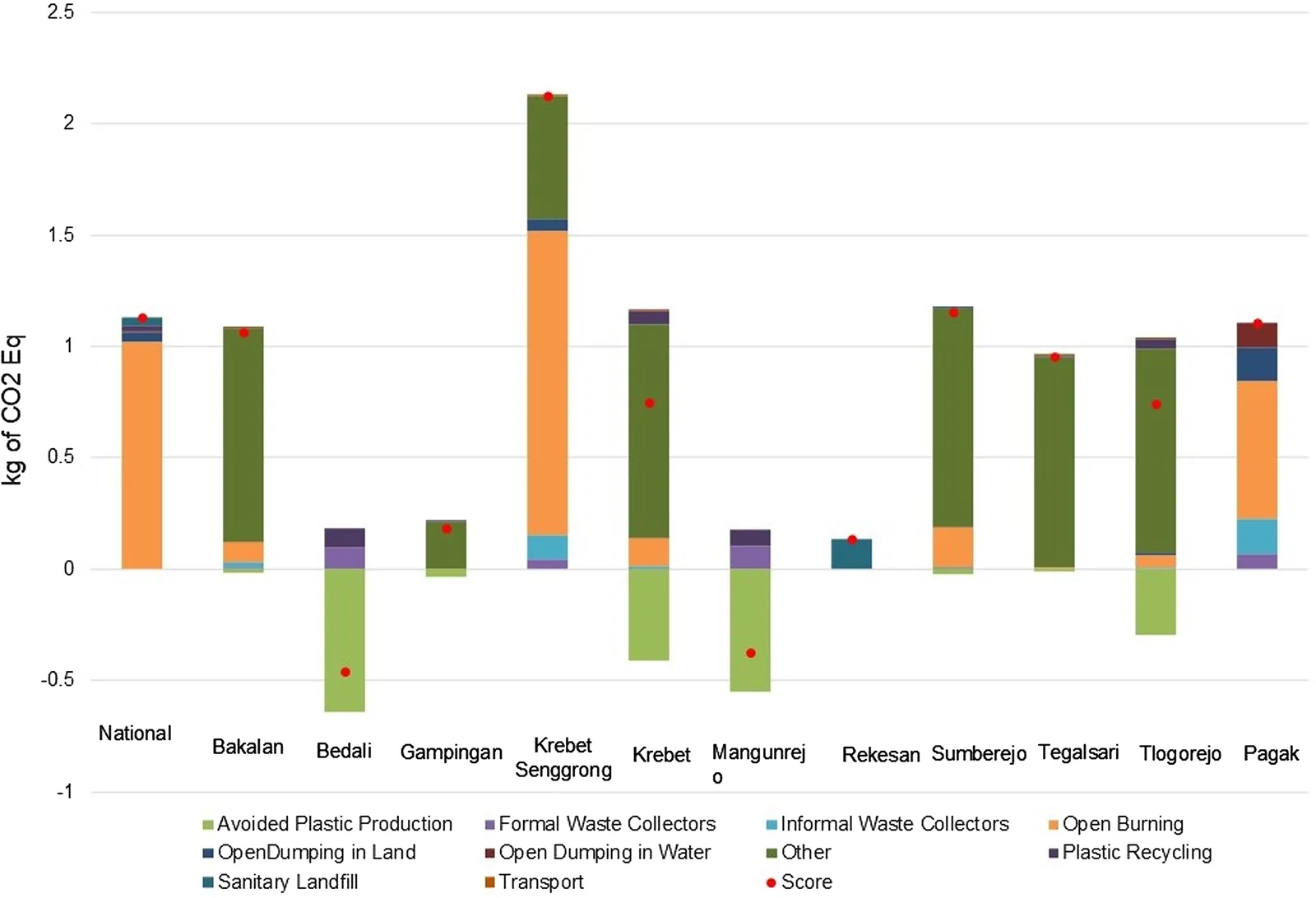
Global warming potential of baseline scenario villages

In Fig. 3, Krebet Senggrong stands out as the highest global warming potential (GWP100) by almost one kilogram at 2.12 kg of CO_2_ Eq per kg of PW generated, with villages such as Sumberejo, Bakalan, and Pagak trailing behind at 1.15, 1.07, and 1.11 kg of CO_2_ Eq, respectively. The biggest contributor to the GWP100 is open burning, which is represented directly and indirectly in ‘Other’. In the case of Krebet Senggrong, open burning alone contributes to 1.37 kg of CO_2_ Eq, which is almost 65% of its total GWP100. Open burning is also the biggest GWP100 contributor in Pagak, making up 55% of the total GHG emissions. In the case of Bakalan and Sumberejo, the biggest contributor is ‘other’ waste management strategies, which make up approximately 85% of the total GWP100 in both villages. Four of the villages in this study stand out in the GWP100 category for their low results, with two of these cases even showing negative results. Rekasan presents 0.137 kg of CO_2_ Eq per kg of PW generated; this is because the data collected in this village is only associated with sanitary landfilling, which is expected to have low GHG emissions when referring to landfilling of PW alone. In the cases of Gampingan, Bedali, and Mangunrejo, the results of GWP100 are 0.18, -0.46, and − 0.37 kg of CO_2_ Eq per kg of PW generated, respectively. These have one factor in common, there is an assumed avoided production of virgin plastics when PW is recycled, which shows up in the LCA as negative emissions and, in Bedali and Mangunrejo, results in negative GWP100.

#### Primary energy depletion

**Fig. 4:**
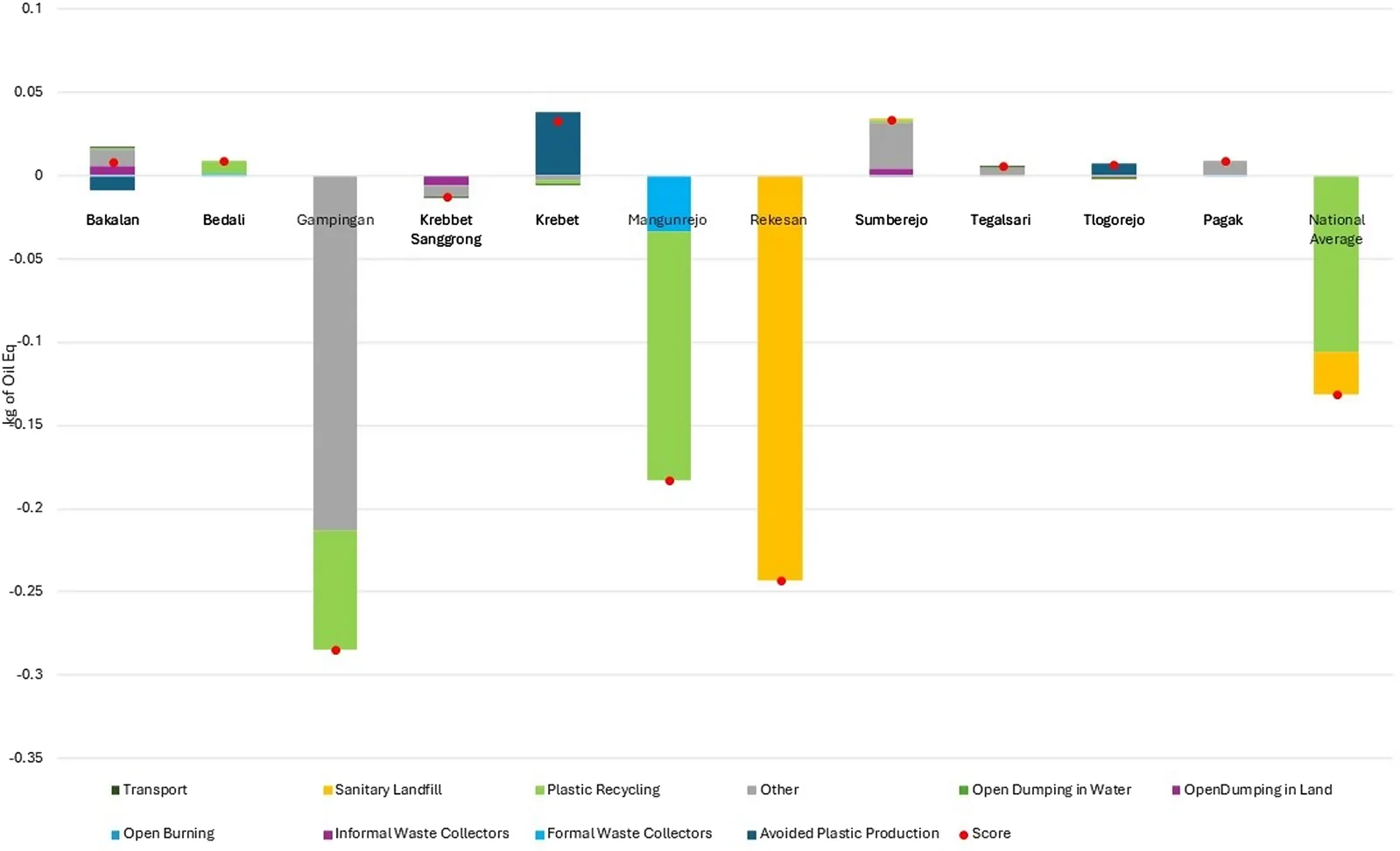
Fossil fuel potential of baseline scenario villages

In the context of primary energy depletion, five of the villages exhibit net negative FFP (see Fig. 4). This outcome is attributed to the recycling of plastic materials, which offsets the fossil fuel-intensive processes associated with virgin plastic production. The villages with the lowest FFP are Bedali, Gampingan, Krebet, Mangunrejo, and Tlogorejo, with the results of -0.29, -0.013, -0.18, -0.24, and -0.13 kg of oil Eq per kg of PW generated, respectively. Conversely, the villages of Pagak and Krebet Senggrong exhibit the highest FFP, 0.034 kg and 0.33 kg of oil Eq, respectively. The main contributors in these villages, is the larger amount of PW collected by Informal Waste Collectors. The rest of the villages have results in the range of 0.005 to 0.009 kg of oil Eq per kg of PW generated, which is closer to the national average of 0.0078 kg of oil Eq per kg of PW generated. It is important to note that waste management strategies that have had a big impact in other categories are less relevant here, as FFP is not directly linked to air pollution in this study. Some examples include open burning, open dumping, and other waste management strategies.

#### Particulate matter formation

**Fig. 5:**
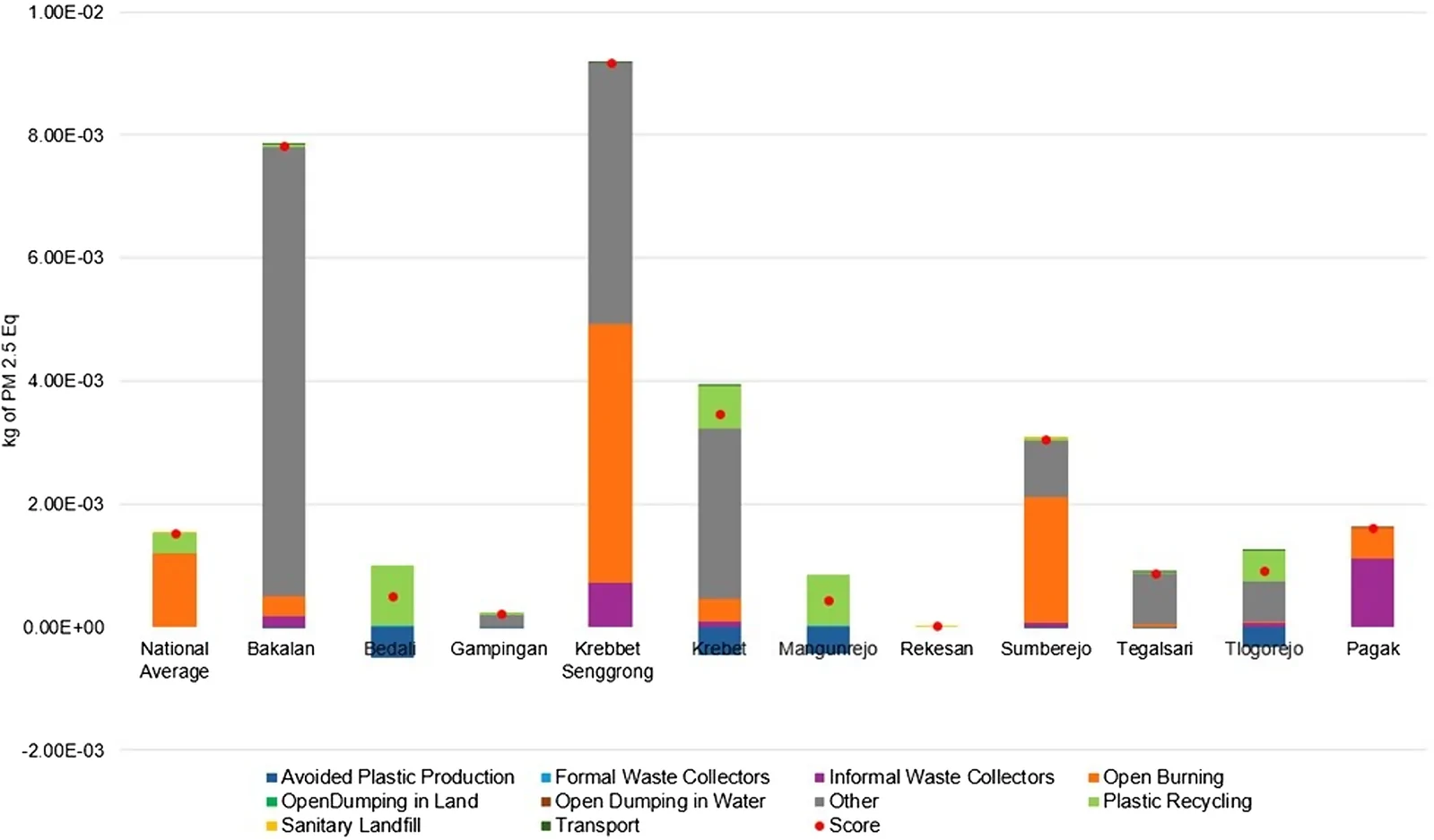
Particulate matter formation potential of baseline scenario villages

In the case of particulate matter formation (Fig. 5), Krebet Senggrong and Bakalan have the highest PMFP at 0.0078 and 0.0091 kg of PM_2.5_ Eq per kg of PW generated. The biggest contributor in Bakalan is ‘Other’, whereas in the case of Krebet Senggrong, it is open burning and ‘Other’ to a similar degree. In other villages, such as Sumberejo and Pagak, open burning is also a notable contributor to the PMFP. The villages with the lowest PMFP are Rekasan, Gampingan, Mangunrejo, and Bedali, with a PMFP of 2.25 E-05, 0.000225, 0.000432, and 0.0005 kg of PM_2.5_ Eq per kg of PW generated, respectively. In the case of Rekasan, the relatively low impact is attributed to the higher rates of sanitary landfilling of PW. In contrast, the villages of Gampingan, Mangunrejo, and Bedali show higher contributions due to PW recycling. These results include the avoided PMFP that is linked to the avoided plastic production.

### Sensitivity analysis

The results are compiled in Tables 2, 3, and 4 with both the results of each scenario and the percentage variation with respect to the baseline scenario. The scenario of Uncollected Waste was the least impacted in the sensitivity analysis as the impact indicators were tied to air pollution. Variations were rarely visible, and mostly in villages with high recycling rates, as the avoided virgin plastic production was reduced, leading to higher emissions that had previously been offset (e.g., Mangunrejo). In the case of open burning, the largest changes were observed in villages where open burning was predominant (e.g., Pagak and Krebet Senggrong), and this shows in GWP100 and PMFP as they are impact indicators that are directly associated with air pollution. This was also noticeable on the sensitivity analysis of the National Average with a variation of ± 9% in GWP100. Recycling rates mostly affected villages with notable EoL pathways that were categorised as ‘Others’ because they were unknown; particularly in the PMFP indicators as waste that had previously been dumped was now assumed to be recycled in informal settings, with pollution capture less effective than in Western facilities and no significant increases in the avoided virgin plastic production. The scenario of Avoided Virgin Plastic Production affected villages with higher recycling rates.

**Table 2:** Sensitivity analysis for GWP in modeling Indonesia’s plastic waste EOL fates

Village	Baseline Scenario	% Uncollected Plastic Waste		% Open Burning		% Plastic Recycled		% Avoided Virgin Plastic Production	
		10%	-10%	10%	-10%	10%	-10%	10%	-10%
Pagak	1.11E + 00	1.14E + 00 (2%)	1.08E + 00 (-2%)	1.17E + 00 (5%)	1.05E + 00 (-6%)	1.11E + 00 (0%)	1.11E + 00 (0%)	1.11E + 00 (0%)	1.11E + 00 (0%)
Tlogorejo	7.41E-01	7.42E-01 (0%)	7.40E-01 (0%)	7.46E-01 (1%)	7.36E-01 (-1%)	7.45E-01 (1%)	7.37E-01 (-1%)	7.11E-01 (-4%)	7.71E-01 (4%)
Tegalsari	9.53E-01	9.53E-01 (0%)	9.53E-01 (0%)	9.54E-01 (0%)	9.52E-01 (0%)	9.53E-01 (0%)	9.52E-01 (0%)	9.51E-01 (0%)	9.54E-01 (0%)
Rekasan	1.38E-01	1.38E-01 (0%)	1.38E-01 (0%)	1.38E-01 (0%)	1.38E-01 (0%)	1.38E-01 (0%)	1.38E-01 (0%)	1.38E-01 (0%)	1.38E-01 (0%)
Sumberejo	1.15E + 00	1.15E + 00 (0%)	1.15E + 00 (0%)	1.16E + 00 (1%)	1.14E + 00 (-2%)	1.15E + 00 (0%)	1.15E + 00 (0%)	1.15E + 00 (0%)	1.16E + 00 (0%)
Mangunrejo	-3.75E-01	-3.75E-01 (0%)	-3.75E-01 (0%)	-3.75E-01 (0%)	-3.75E-01 (0%)	-3.67E-01 (2%)	-3.82E-01 (-2%)	-4.30E-01 (15%)	-3.20E-01 (-15%)
Krebet	7.50E-01	7.50E-01 (0%)	7.50E-01 (0%)	7.69E-01 (2%)	7.44E-01 (-1%)	7.62E-01 (2%)	7.50E-01 (0%)	7.71E-01 (3%)	7.97E-01 (6%)
Krebet Senggrong	2.12E + 00	2.13E + 00 (0%)	2.12E + 00 (0%)	2.26E + 00 (6%)	1.99E + 00 (-6%)	2.12E + 00 (0%)	2.12E + 00 (0%)	2.12E + 00 (0%)	2.12E + 00 (0%)
Gampingan	1.83E-01	1.83E-01 (0%)	1.83E-01 (0%)	1.83E-01 (0%)	1.83E-01 (0%)	1.84E-01 (0%)	1.83E-01 (0%)	1.80E-01 (-2%)	1.87E-01 (2%)
Bedali	-4.58E-01	-4.58E-01 (0%)	-4.58E-01 (0%)	-4.58E-01 (0%)	-4.58E-01 (0%)	-4.50E-01 (-2%)	-4.66E-01 (2%)	-5.22E-01 (14%)	-3.94E-01 (-14%)
Bakalan	1.07E + 00	1.07E + 00 (0%)	1.07E + 00 (0%)	1.08E + 00 (1%)	1.06E + 00 (-1%)	1.07E + 00 (0%)	1.07E + 00 (0%)	1.07E + 00 (0%)	1.07E + 00 (0%)
National Average	1.13E + 00	1.13E + 00 (0%)	1.12E + 00 (0%)	1.23E + 00 (9%)	1.03E + 00 (-9%)	1.15E + 00 (2%)	1.14E + 00 (1%)	1.13E + 00 (0%)	1.13E + 00 (0%)

**Table 3:** Sensitivity analysis for FFP in modeling Indonesia’s plastic waste EOL fates

Village	Baseline Scenario	% Uncollected Plastic Waste		% Open Burning		% Plastic Recycled		% Avoided Virgin Plastic Production	
		10%	-10%	10%	-10%	10%	-10%	10%	-10%
Pagak	3.36E-02	3.36E-02 (0%)	3.36E-02 (0%)	3.36E-02 (0%)	3.36E-02 (0%)	3.36E-02 (0%)	3.36E-02 (0%)	3.36E-02 (0%)	3.36E-02 (0%)
Tlogorejo	-1.31E-01	-1.31E-01 (0%)	-1.31E-01 (0%)	-1.31E-01 (0%)	-1.31E-01 (0%)	-1.30E-01 (-1%)	-1.32E-01 (1%)	-1.47E-01 (12%)	-1.16E-01 (-12%)
Tegalsari	8.78E-03	8.78E-03 (0%)	8.78E-03 (0%)	8.78E-03 (0%)	8.78E-03 (0%)	8.83E-03 (1%)	8.73E-03 (-1%)	8.13E-03 (-7%)	9.45E-03 (8%)
Rekasan	5.65E-03	5.65E-03 (0%)	5.65E-03 (0%)	5.65E-03 (0%)	5.65E-03 (0%)	5.65E-03 (0%)	5.65E-03 (0%)	5.65E-03 (0%)	5.65E-03 (0%)
Sumberejo	6.34E-03	6.34E-03 (0%)	6.34E-03 (0%)	6.34E-03 (0%)	6.34E-03 (0%)	6.41E-03 (1%)	6.28E-03 (-1%)	5.35E-03 (-16%)	7.33E-03 (16%)
Mangunrejo	-2.43E-01	-2.43E-01 (0%)	-2.43E-01 (0%)	-2.43E-01 (0%)	-2.43E-01 (0%)	-2.42E-01 (-1%)	-2.45E-01 (1%)	-2.70E-01 (11%)	-2.17E-01 (-11%)
Krebet	-1.83E-01	-1.83E-01 (0%)	-1.83E-01 (0%)	-1.83E-01 (0%)	-1.83E-01 (0%)	-1.82E-01 (-1%)	-1.85E-01 (1%)	-1.80E-01 (-2%)	-1.62E-01 (-12%)
Krebet Senggrong	3.28E-02	3.28E-02 (0%)	3.28E-02 (0%)	3.28E-02 (0%)	3.28E-02 (0%)	3.28E-02 (0%)	3.28E-02 (0%)	3.28E-02 (0%)	3.28E-02 (0%)
Gampingan	-1.25E-02	-1.25E-02 (0%)	-1.25E-02 (0%)	-1.25E-02 (0%)	-1.25E-02 (0%)	-1.24E-02 (-1%)	-1.26E-02 (1%)	-1.42E-02 (13%)	-1.09E-02 (-13%)
Bedali	-2.85E-01	-2.85E-01 (0%)	-2.85E-01 (0%)	-2.85E-01 (0%)	-2.85E-01 (0%)	-2.83E-01 (-1%)	-2.87E-01 (1%)	-3.16E-01 (11%)	-2.54E-01 (-11%)
Bakalan	9.08E-03	9.08E-03 (0%)	9.08E-03 (0%)	9.08E-03 (0%)	9.08E-03 (0%)	9.14E-03 (1%)	9.01E-03 (-1%)	8.04E-03 (-11%)	1.01E-02 (11%)
National Average	7.83E-03	7.83E-03 (0%)	7.83E-03 (0%)	7.83E-03 (0%)	7.83E-03 (0%)	8.47E-03 (8%)	7.20E-03 (-8%)	7.83E-03 (0%)	7.83E-03 (0%)

Numerical change from base result is shown in parentheses

**Table 4:** Sensitivity analysis for PMFP in modelling Indonesia’s plastic waste EOL fates

Village	Baseline Scenario	% Uncollected Plastic Waste		% Open Burning		% Plastic Recycled		% Avoided Virgin Plastic Production	
		10%	-10%	10%	-10%	10%	-10%	10%	-10%
Pagak	1.61E-03	1.62E-03	1.61E-03	1.43E-03	1.56E-03	1.61E-03	1.61E-03	1.61E-03	1.61E-03
Tlogorejo	9.11E-04	1.62E-03 (0%)	1.61E-03 (0%)	1.43E-03 (-11%)	1.56E-03 (-3%)	1.61E-03 (0%)	1.61E-03 (0%)	1.61E-03 (0%)	1.61E-03 (0%)
Tegalsari	8.80E-04	9.11E-04 (0%)	9.11E-04 (0%)	9.13E-04 (0%)	9.08E-04 (0%)	9.60E-04 (5%)	8.61E-04 (-5%)	8.78E-04 (-4%)	9.43E-04 (4%)
Rekasan	2.25E-05	8.80E-04 (0%)	8.80E-04 (0%)	8.84E-04 (1%)	8.75E-04 (-1%)	8.82E-04 (0%)	8.77E-04 (0%)	8.78E-04 (0%)	8.81E-04 (0%)
Sumberejo	3.05E-03	2.25E-05 (0%)	2.25E-05 (0%)	2.25E-05 (0%)	2.25E-05 (0%)	2.25E-05 (0%)	2.25E-05 (0%)	2.25E-05 (0%)	2.25E-05 (0%)
Mangunrejo	4.32E-04	3.05E-03 (0%)	3.46E-03 (14%)	3.24E-03 (6%)	2.84E-03 (-7%)	3.05E-03 (0%)	3.04E-03 (0%)	3.04E-03 (0%)	3.05E-03 (0%)
Krebet	3.46E-03	4.32E-04 (0%)	4.32E-04 (0%)	4.32E-04 (0%)	4.32E-04 (0%)	5.17E-04 (20%)	3.48E-04 (-20%)	3.90E-04 (-10%)	4.75E-04 (10%)
Krebet Senggrong	9.18E-03	3.46E-03 (0%)	3.46E-03 (0%)	3.50E-03 (1%)	3.43E-03 (-1%)	3.53E-03 (2%)	3.39E-03 (-2%)	3.49E-03 (1%)	3.10E-03 (-10%)
Gampingan	2.22E-04	9.18E-03 (0%)	9.18E-03 (0%)	9.60E-03 (5%)	8.76E-03 (-5%)	9.18E-03 (0%)	9.18E-03 (0%)	9.18E-03 (0%)	9.18E-03 (0%)
Bedali	5.02E-04	2.22E-04 (0%)	2.22E-04 (0%)	2.22E-04 (0%)	2.22E-04 (0%)	2.28E-04 (2%)	2.17E-04 (-2%)	2.20E-04 (-1%)	2.25E-04 (1%)
Bakalan	7.82E-03	5.02E-04 (0%)	5.02E-04 (0%)	5.02E-04 (0%)	5.02E-04 (0%)	6.01E-04 (20%)	4.04E-04 (-20%)	4.53E-04 (-10%)	5.52E-04 (10%)
National Average	1.53E-03	7.82E-03 (0%)	7.82E-03 (0%)	7.86E-03 (0%)	7.79E-03 (0%)	7.82E-03 (0%)	7.82E-03 (0%)	7.82E-03 (0%)	7.82E-03 (0%)

Numerical change from base result is shown in parentheses

### Potential intervention strategies

For this section of the results, the villages of Bakalan, Krebet Senggrong, Krebet, and Tlogorejo were selected for intervention. To evaluate how the current practices and the alternative management practices compare, the PW is modelled undergoing the current practices, and for the case of alternative management, the PW is modelled undergoing sanitary landfilling, recycling, and incineration with energy recovery.

#### Bakalan

**Fig. 6:**
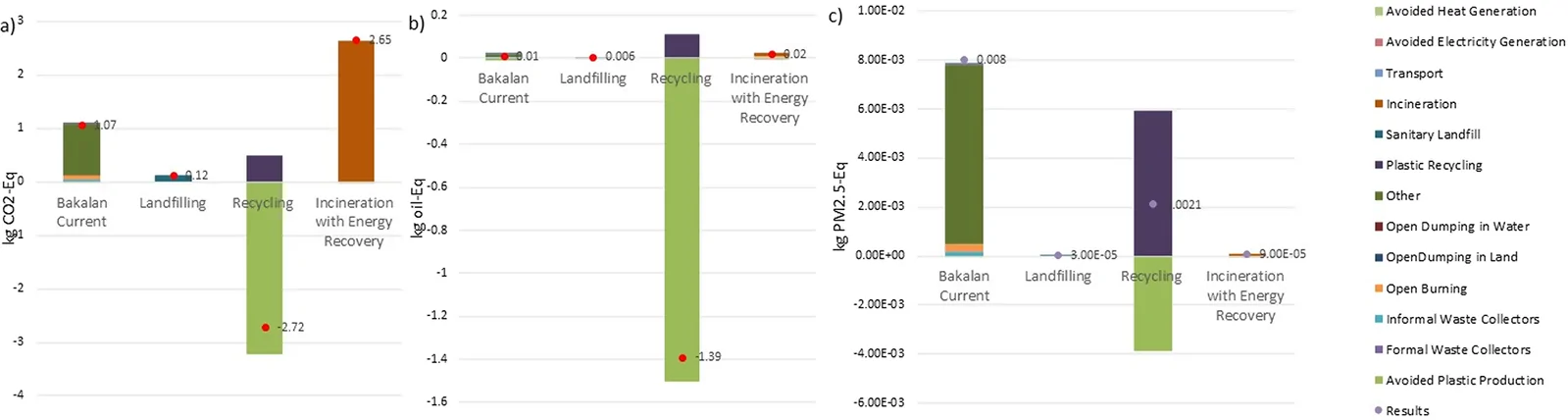
(**a**) Global warming potential of Bakalan and alternative plastic waste management methods. (**b**) Fossil fuel potential of Bakalan and alternative plastic waste management methods. (**c**) Particulate matter formation of Bakalan and alternative plastic waste management methods

In the baseline scenario of Bakalan, the GWP100 is 1.1 kg of CO_2_ Eq, per kg of PW generated with ‘other’ waste management being the largest contributor (0.96 kg of CO_2_ Eq which is over 87%) and open burning trailing in second place with GWP100 of 0.089 kg of CO_2_ Eq per kg of PW generated (approximately 8.1% of GWP100). Landfilling has ten times lower GWP100 at 0.11 kg of CO_2_ Eq per kg of PW generated, and recycling outperforms landfilling in this category with a result of -2.72 kg of CO_2_ Eq per kg of PW generated (Fig. 6a). This is due to the avoided plastic production (-3.22 kg of CO_2_ Eq per kg of PW generated), which counteracts the GWP100 of the recycling process (0.5 kg of CO_2_ Eq per kg of PW generated). Incineration is the intervention option with the highest GWP100 at (2.64 kg of CO_2_ Eq per kg of PW generated) as a total with electricity and heat recovery being included (-6.11E-03 and -4.82E-04 kg of CO_2_ Eq per kg of PW generated, respectively).

In the case of fossil fuel depletion (Fig. 6b), the results for recycling are low despite the process of recycling itself having the highest FFP because the avoided plastic production is included, resulting in -1.39 kg of oil Eq per kg of PW generated. This is because plastics are derived from fossil fuels, and there is a treatment process that also requires energy and chemical resources, which can contribute to a higher FFP. When it comes to landfilling, the results are at 0.0054 kg of oil Eq, lower than the baseline scenario in Bakalan (0.0091 kg of oil Eq per kg of PW generated). In the case of incineration, FFP is higher than the baseline scenario, with a result of 0.011 kg of oil Eq per kg of PW generated.

When it comes to PMFP (Fig. 6c), all alternatives to the baseline scenario in Bakalan are better, with landfilling and incineration being the lowest at 2.6E-05 and 8.3E-05 of PM_2.5_ Eq per kg of PW generated, respectively. Recycling in this instance has a higher PMFP at 0.002 kg of PM_2.5_ Eq per kg of PW generated but remains considerably lower than the baseline scenario (0.0078 kg of PM_2.5_ Eq per kg of PW generated).

#### Krebet Senggrong

**Fig. 7:**
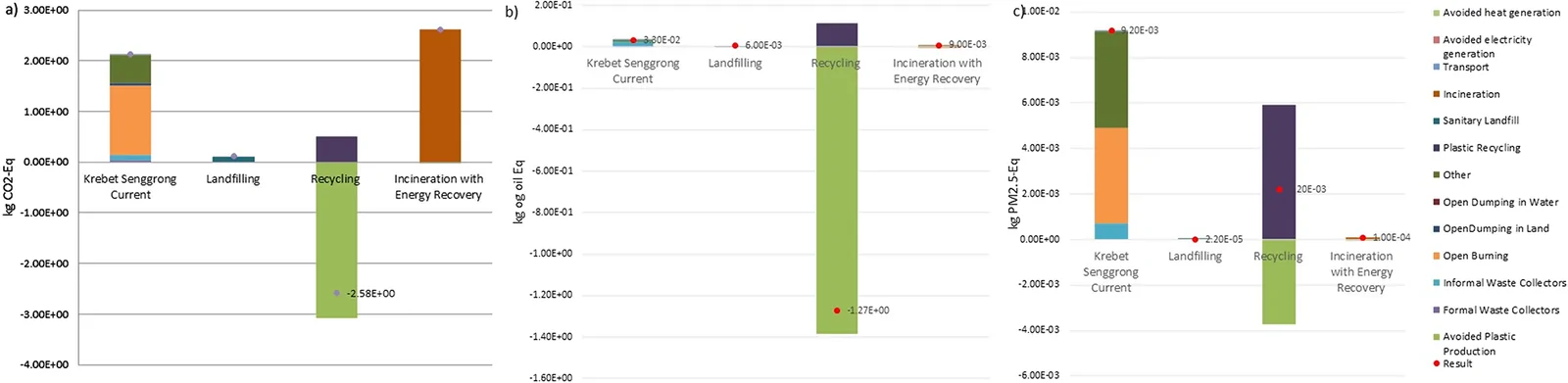
(**a**) Global warming potential of Krebet Senggrong and alternative plastic waste management methods. (**b**) Fossil fuel potential of Krebet Senggrong and alternative plastic waste management methods. (**c**) Particulate matter formation of Krebet Senggrong and alternative plastic waste management methods

In the baseline scenario, the GWP100 is at 2.12 kg of CO_2_ Eq per kg of PW generated and is mostly associated with the open burning (1.37 kg of CO_2_ Eq per kg of PW generated) and ‘Other’ (0.55 kg of CO_2_ Eq per kg of PW generated). Similarly to the case of Bakalan, the lowest GWP100 is associated with recycling at a total of -2.57 kg of CO2 Eq (when avoided plastic production is included as it amounts to -3.08 kg of CO_2_ Eq) and landfilling, which has a GWP100 of 0.11 kg of CO_2_ Eq per kg of PW generated (Fig. 7a). Incineration has a higher GWP100 than the baseline scenario for Krebet Senggrong, with a result of 2.62 kg of CO_2_ Eq per kg of PW generated.

In the case of FFP (Fig. 7b), recycling has the lowest results due to the avoided production of plastics which results in a FFP of -1.27 kg of oil Eq per kg of PW generated. This is because the FFP of the recycling process (0.11 kg of oil Eq per kg of PW generated) is offset by the avoided plastic production (-1.39 kg of CO_2_ Eq per kg of PW generated). Landfilling and incineration also have a lower FFP when compared to the baseline scenario, with a FFP of 0.005 and 0.008 kg of oil Eq per kg of PW generated. All the proposed waste management options offer a significant improvement when compared to the baseline scenario, which has an FFP of 0.03 kg of oil Eq per kg of PW generated, where informal waste collectors are the largest contributors, making up 78% of the FFP.

Oppositely to GWP100 and FFP, recycling is the proposed alternative with the highest PMFP, at 0.0022 kg of PM_2.5_ Eq per kg of PW generated, even with the avoided plastic production (Fig. 7c). But it is still an improvement from the baseline scenario, which has a PMFP of 0.0092 kg of PM_2.5_ Eq per kg of PW generated. Meanwhile, landfilling and incineration have considerably lower results at 2.13 E-05 and 7.13 E-05 kg of PM_2.5_ Eq per kg of PW generated.

#### Krebet

**Fig. 8:**
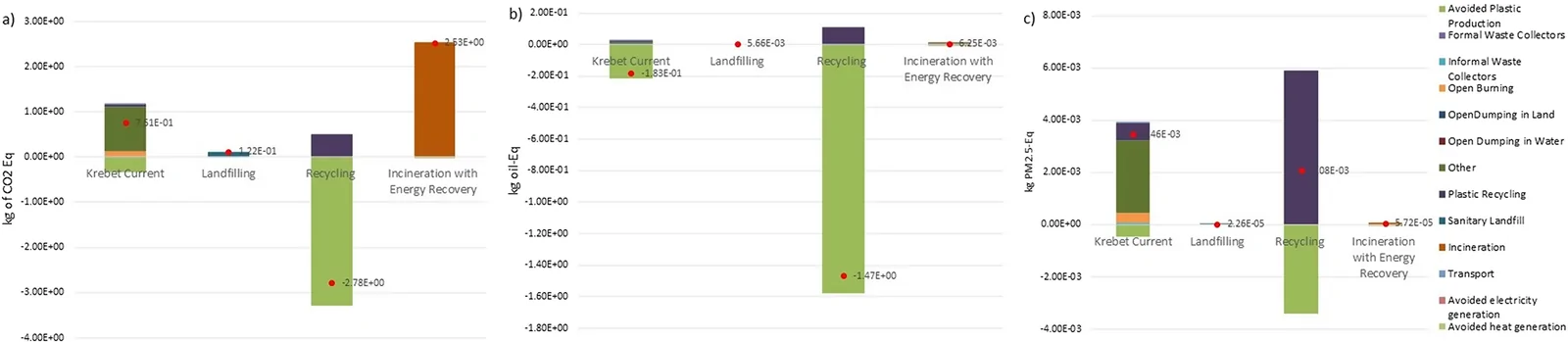
(**a**) Global warming potential of Krebet and alternative plastic waste management methods. (**b**) Fossil fuel potential of Krebet and alternative plastic waste management methods. (**c**) Particulate matter formation of Krebet and alternative plastic waste management methods

The baseline scenario of PW management has a GWP100 of 0.75 kg of CO_2_ Eq per kg of PW generated. When comparing the alternatives, landfilling and recycling offer significant reductions in GWP100, with an emission of 0.12 and − 2.77 kg of CO_2_ Eq per kg of PW generated. The negative result of recycling includes the negative emissions from avoided plastic production. In the case of plastic incineration, the GWP100 is higher than the baseline scenario, at 2.53 kg of CO_2_ Eq per kg of PW generated (Fig. 8a). The results include flue gas cleaning, air pollution control, and avoided heat and electricity generation.

FFP in the baseline scenario is negative at -0.18 kg of oil Eq per kg of PW generated; the only other option that offers an improvement is recycling with a FFP of -1.46 kg of oil Eq per kg of PW generated (Fig. 8b). In this case, landfilling and incineration have the highest FFP with 0.0057 kg and 0.0062 kg of oil Eq per kg of PW generated, respectively.

Regarding the results of PMFP (Fig. 8c), the lowest results are associated with landfilling at 2.6 E-05 kg of PM_2.5_ Eq per kg of PW generated, which is most closely followed by incineration (5.72 E-05 kg of PM_2.5_ Eq per kg of PW generated). Recycling has a significantly higher PMFP at 0.0021 kg of PM_2.5_ Eq per kg of PW generated, but still fares better than the baseline scenario, which has a result of 0.0035 kg of PM_2.5_ Eq per kg of PW generated.

#### Tlogorejo

**Fig. 9:**
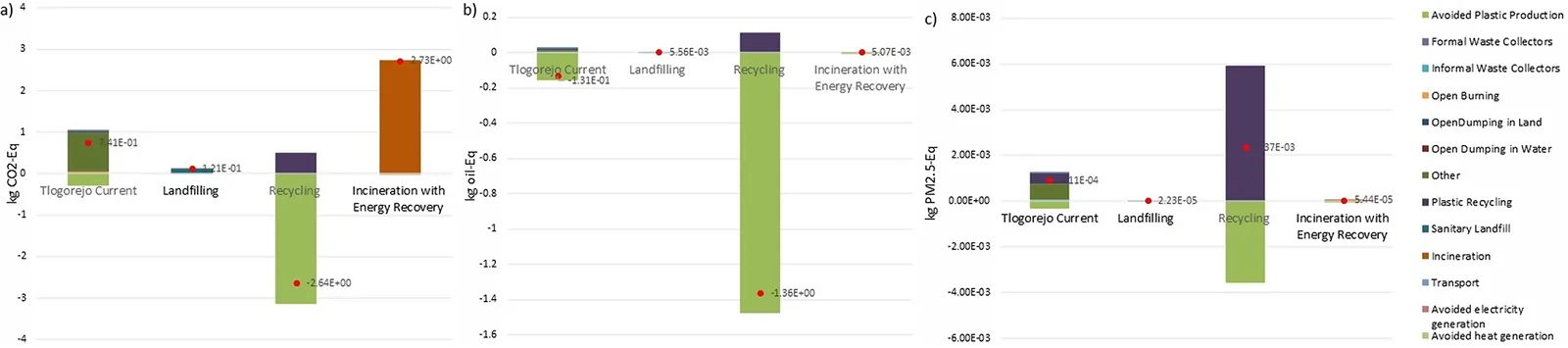
(**a**) Global warming potential of Tlogorejo and alternative plastic waste management methods. (**b**) Fossil fuel potential of Tlogorejo and alternative plastic waste management methods. (**c**) Particulate matter formation of Tlogorejo and alternative plastic waste management methods

In Fig. 9a, the results for GWP100 show that the baseline scenario has an impact of 0.74 kg of CO_2_ Eq per kg of PW generated, with the largest contributor being ‘Other’ (0.92 kg of CO_2_ Eq per kg of PW generated), counteracted by the avoided plastic production (-0.3 kg of CO_2_ Eq per kg of PW generated). Meanwhile, landfilling has a lower GWP100 at 0.12 kg of CO_2_ Eq per kg of PW generated, and recycling has the lowest results at -2.64 kg of CO_2_ Eq per kg of PW generated due to the large amount of GHG emissions that are not released due to avoided plastic production (-3.14 kg of CO_2_ Eq per kg of PW generated). However, incineration has the highest GWP100, at 2.73 kg of CO_2_ Eq per kg of PW generated.

When looking at the results of FFP (Fig. 9b), the baseline scenario has a negative result (-0.13 kg of oil Eq per kg of PW generated) due to the avoided plastic production (-0.15 kg of oil Eq per kg of PW generated). Recycling has an even lower result at -1.36 kg of oil Eq per kg of PW generated. Meanwhile, the FFP results of landfilling and incineration are higher, at 0.055 and 0.0051 kg of oil Eq per kg of PW generated, respectively.

In Fig. 9c, PMFP recycling has the highest impact at 0.0024 kg of PM_2.5_ Eq per kg of PW generated, surpassing the baseline scenario (0.00091 kg of PM_2.5_ Eq per kg of PW generated). Landfilling offers the best results in this category, followed by incineration, with results of 2.23E-05 and 5.44E-05 kg of PM_2.5_ Eq per kg of PW generated, respectively.

## Discussion

### Baseline scenarios

When looking at the results of the hotspot analysis, open burning is the waste management method that has the largest environmental impact across most of the impact categories. Open burning is explicitly represented within the ‘Open Burning’ category and implicitly captured under the ‘Other’ category, wherein approximately 36% of PW is subjected to combustion. This dual representation underscores the complexity of waste management practices and highlights the need for careful interpretation of inventory data to avoid underestimating the environmental impacts associated with burning activities. The impact of open burning is more apparent in impact categories linked to air pollution, such as GWP100 and PMFP. The selected environmental impact categories are pertinent not only for evaluating GHG contributions and PMFP, but also for assessing implications for human health, particularly, respiratory outcomes among populations exposed to open burning regularly. The inclusion of open burning in the life cycle inventory is therefore critical, as it represents a significant source of airborne pollutants, including fine particulate matter and toxic compounds, which pose direct health risks to exposed communities (Pathak et al. 2024). This is supported beyond the case of Malang, because pollution was the cause of 15% of all deaths and many more disabilities in 2017 (Pathak et al. 2024). Plastic burning is a phenomenon that affects many regions where there is no suitable waste management infrastructure, namely in the Global Majority but also in certain parts of the Global North. Bharadwaj et al. found that another reason why open burning is a popular waste management method in these scenarios is the need for energy, coupled with costly and inaccessible clean fuel technologies (Bharadwaj et al. 2026; Haryanto 2018).

The process of recycling in itself has a considerable impact across all the categories included in the study, but is offset by the avoided plastic production, as is common practice in similar studies (Neo et al. 2021). Plastic recycling is represented within the inventory. It is also implicitly present in the activities of informal waste collectors who, according to primary data, recycle the entirety of the PW they recover. Additionally, recycling is reflected to a lesser extent (approximately 5.6%) within the ‘Other’ category. This layered representation highlights the multifaceted nature of recycling practices, encompassing both formal and informal systems, and underscores the importance of accurately characterising waste flows to ensure a robust LCA. The reason for this is that recycling plastic requires considerable amounts of energy for the numerous phases, which include sorting, grinding, melting, etc. (Association of Plastic Recyclers 2025; Jung et al. 2024). While plastic recycling displaces virgin plastic production and thereby supports resource conservation, this environmental benefit does not extend uniformly across all impact categories. Specifically, PMFP is unaffected by such offsets, as emissions associated with the stages of recycling may contribute adversely to ambient air quality. This underscores the importance of a nuanced interpretation of trade-offs within life cycle impact assessment, wherein gains in climate change mitigation and resource efficiency may be accompanied by localized environmental and public health burdens. Pagak village emerges as a notable case within Malang, distinguished by the predominance of open dumping as the principal PW management practice. Plastic is open dumped (in a field or other land) in almost 20% of cases, and open dumping in a river or a creek, in 40% of cases. Uncontrolled disposal methods, such as open dumping and informal burning, pose significant risks to environmental and human health impact categories, including land degradation, leachate formation, and pollutant exposure (Siddiqua et al. 2022). Open dumping may also occur undetected in cases where landfill sites are inadequately managed or monitored, which, in the case of Indonesia, occurs frequently (Munawar et al. 2018). Such lapses in operational oversight can result in uncontrolled emissions and pollutant release, with implications for the environment and health of the nearby population. To mitigate these risks, the implementation of systematic monitoring and maintenance protocols is essential to ensure environmental compliance and data integrity within LCIs (OECD 2019; Prata et al. 2019). In future research, the conditions of the landfills should be evaluated to provide more accurate estimates as there is a lack of monitoring and sites the waste management system is struggling to meet the requirements due to the increasing amount of waste and the limited resources.

Although most villages have a considerable amount of waste treated as ‘Other’, Bakalan and Sumberejo stand out with over 80% percent of the waste management practices are categorized as ‘Other’. This indicates that none of the predefined waste management options were applicable to the respondents; however, further inquiry revealed either a lack of accurate data or limited awareness regarding actual disposal practices. Such data gaps highlight the challenges in accurately characterising waste flows and underscore the need for improved reporting and stakeholder engagement in LCI development. This absence of systematic tracking and accounting of waste flows presents a significant barrier to accurately quantifying the environmental impacts of current waste management practices. This data deficiency may hinder both the robustness of LCAs and the evidence-based policy recommendations on PW management.

The composition of PW varies across villages, with differing proportions observed in each location (see Figure S1 for detailed information). LDPE and PP are the most prevalent, followed by PET and HDPE, which also feature prominently. This distribution aligns with national composition patterns, reflecting the widespread use of these polymer types (Ministry of Environment and Forestry 2020). Similarly to the national average, it comes to the plastic mix for Krebet Senggrong, it is quite varied, with LDPE and PET being the main plastic types, similar to Sumberejo, Pagak, and Bakalan. In Sumberejo, PET (44.8%) and LDPE (42%) dominate the waste stream, whereas Pagak and Bakalan exhibit a more heterogeneous mix, with notable shares of PP and HDPE. Additionally, PS constitutes a significant fraction in Pagak. It is also important to note that in the case of both Krebet Senggrong and Bakalan, over 80% of the plastic mix is made up of HDPE, LDPE, PP, and PET. Although in the case of Bakalan, the most common plastic is LDPE (32.5%), and in the case of Krebet Senggrong, it is PET (31.2%). There are no definitive results that would indicate that a certain type of plastic in this study has a drastically different impact on the results. These variations reflect local PW composition patterns and underscore the importance of site-specific inventory data in LCAs. Although the data is quite comprehensive, it is possible that it is not completely accurate because the PW was not tested and sorted by the enumerators. Instead, the enumerators asked the surveyed stakeholders to provide the PW generated of each type (which was weighed by the enumerators) with a detailed explanation of what each type is and common uses to aid stakeholders in the sorting process.

However, when it comes to waste management practices, Krebet Senggrong stands out from the other villages assessed in Malang (see Figure S2 for more detailed information). Almost 35% of PW in Krebet Senggrong is open burned, which is similar to the national average (36%). However, when compared with the other villages with higher GWP100, Krebet Senggrong has higher open burning rates. Other notable waste management strategies include open dumping and waste collected by public waste collectors, which is assumed to end up in sanitary landfills, with some of the waste being displaced and accidentally dumped. This was based on the national report, as the interviewed stakeholders, which in this case were households, offices, industrial sites, and businesses, were unable to provide more information (Ministry of Environment and Forestry 2020). Bakalan, Pagak, and Sumberejo also had a higher GWP100 amongst the selected villages. Although waste collectors are not the primary contributors to waste volumes (see Figure S2), they play a critical role in the functioning of the waste management infrastructure by facilitating material flow and connecting key stakeholders (see Tables S1 to S12 in Supplementary Information). Enhancing their capacity through targeted training on waste management standards and guidelines could improve system performance and data reliability. Given the increasing waste generation rates, broad stakeholder engagement, including the informal sector, is essential to strengthen the overall resilience and effectiveness of the waste management (Harfadli et al. 2025; OECD 2019).

In both Krebet Senggrong and Bakalan, a considerable percentage is associated with ‘Other’ waste management strategies. This is because approximately 36% of waste categorized as being managed as ‘Other’ is open burned. In the case of Krebet Senggrong, 35% of the PW is open burned, which results in 2.89 E-03 kg of PM_2.5_ of the almost 8E-03 kg of PM_2.5_ produced in total. Additionally, the management pathways categorised as ‘Other’ include open dumping and collection by public waste collectors, which were assumed to result primarily in landfill disposal based on national reports, as respondents were generally unable to provide information regarding the final destination of collected waste (Ministry of Environment and Forestry 2020). These findings demonstrate that the technical recyclability of a material does not necessarily correspond to its observed end-of-life management pathway. This is due to the lack of infrastructure for appropriate and sanitary waste management in the region. The reason for the inability to meet the PW generation demands is due to lack of funds and growing rate of PW generation, which stems from various reasons such as economic and population growth (Pambudi et al. 2024).

This observation has important implications for the interpretation of the sensitivity analysis. The assessed scenarios represent potential changes in waste management pathways; however, the baseline results indicate that a considerable share of potentially recyclable plastics is currently managed through disposal routes rather than recovery pathways. Therefore, the sensitivity analysis should be interpreted as an assessment of the potential environmental consequences of alternative management scenarios rather than a prediction of current waste management performance. Although some uncertainty remains because plastics were assigned to polymer categories by product type during the field audit rather than by laboratory identification, the dominant polymer types observed are broadly consistent with national waste composition data. Consequently, the results suggest that observed differences in environmental impacts are closely associated with waste management pathways, particularly the prevalence of open burning, as well as the quantity and composition of PW generated.

### Sensitivity analysis

In the sensitivity analysis, not all villages were sensitive to the presented scenarios. This is because the primary data in certain villages was not collected from the stakeholders for one or several scenarios. This could be because there were no such stakeholders in those villages, as could be the case with a sanitary landfill, which is only available in Rekasan, or because the data was of low quality or unavailable.

Because there is uncertainty associated with the primary data, a sensitivity analysis provides an understanding of what EoL pathways have the biggest impact on the resulting emissions. Increase in open burning has the largest impact throughout, this is because the environmental impact indicators selected are directly associated with air pollution. For this reason, larger rates of uncollected waste, which leads to an increase in open dumping, show lesser changes in the sensitivity analysis. Recycling offered positive results in the baseline scenarios due to the avoided plastic production (Goh et al. 2025; Neo et al. 2021). However, in this case, there is a high level of uncertainty because recycling is less formalised than in countries with higher access to infrastructure. The avoidance of virgin plastic production is another factor that shows a larger variation and is feasible, as this is a number that is not always reflective of the situation (Jeswani et al. 2021; Moyer et al. 2025).

### Interventions

Recycling performs quite well across the majority of the selected environmental impact categories because of the avoided plastic production. This assumption has been made in other studies similar to this one (Neo et al. 2021). Recycling offers outstanding results for the FFP indicator, because the recycling avoids the energy-intensive process of virgin plastics production and fossil fuel from which plastic is made (Jeswani et al. 2021). Almost 6% of fossil fuel extraction is related to the production of plastics, so the numerically negative results of recycling FFP are offset by the reduction of fossil fuel extraction and associated GHG emissions. However, recycling has a higher PMFP than landfilling and incineration. This is tied to the recycling process itself and the associated electricity demand. The Indonesian electricity grid, which is predominantly sourced from coal (approximately 69% according to the International Energy Agency), has a high environmental impact (International Energy and Agency 2025).

Landfilling exhibits lower environmental impacts than the current waste management scenarios, particularly in air pollution-related categories, as evidenced by reduced GWP100 and PMFP. However, the results presented do not fully capture the broader environmental implications of landfilling, as landfill sites typically contain heterogeneous solid waste streams that, upon degradation, emit GHG into the atmosphere. This underscores the importance of considering long-term emissions and waste composition when interpreting the environmental performance of landfilling within environmental impact assessments (Kristanto and Koven 2019). Additionally, landfilling plastic has an effect on terrestrial and aquatic ecosystems. Siddiqua et al. (2022) explain that rainfall dissolves organic and inorganic elements, which can then leak into underground water systems and beyond, not only affecting ecosystems but also human health. If the landfill is well managed, this risk can be almost fully avoided (Yatoo et al. 2024). As for FFP, landfilling has a small impact but also does not offer any improvements, unlike recycling. It is also worth noting that landfills have a limited capacity and occupy a considerable amount of land, which could be used for other purposes, especially in an area like Malang that is growing and will require more land and resources for its inhabitants (Central Bureau of Statistics in Malang District 2024).

Incineration with energy recovery presents potential as a viable waste management strategy; however, it is characterised by substantial energy and heat demands. Additionally, it is essential to build capacity amongst the population of Malang to make this technology a feasible waste management strategy (Sambodo et al. 2017). In this study, incineration yields a lower FFP relative to plastic recycling when excluding the benefits of avoided virgin plastic production. Nevertheless, the deployment of flue gas treatment technologies, particularly those involving chemical agents such as ammonia, contributes to significant GWP100, illustrating the trade-offs inherent in emission control measures within environmental impact assessment (Damgaard et al. 2010). However, this same technology is the reason why incineration has such a low impact on PMFP. The GWP100 associated with incineration with energy recovery could be mitigated by installing carbon capture units and storage (Liang et al. 2021). Another quality that might appeal in the context of Malang is that it would work well for cases where recycling is not possible or the byproducts of recycling without having limited capacity.

These are all technical interventions that may contribute to a reduction in pollution associated with PW management in Malang District. However, in order to achieve such outcomes, it is important to consider increasing the local governance and capacity with tools such as waste banks (Sambodo et al. 2017).

### Limitations

In this study, although the focus is on Malang District, it was decided to break down the results by villages to obtain more digestible results and for greater granularity. Equally, the results could have been broken down by stakeholder group to assess what stakeholder behaviour impacts the environment most negatively (see Sect. 3 in Supplementary Information). This representation of the results shows a different nuance and adds further value to the results and their interpretation.

Additionally, the suggested alternative strategies were proposed by the simulators, based on suggestions of suitable technologies in this context. When selecting the studied technologies, their feasibility in the short term was not considered. Incineration could be implemented in the longer term as it requires considerable funds, capacity building and infrastructure. Other plausible strategies may have been excluded from this study. This could extend from other technologies to social and economic strategies; these options are worth studying and could offer further benefits to the community. The composition of the PW is a factor that was not prioritised as the focus was on the practices in this case but should be studied further.

Primary data collection for the construction of the LCI in this study has allowed for the results to be more accurate and to be representative of the villages in Malang District, an area for which there is no research similar to this study (Damgaard et al. 2010; Jeswani et al. 2021; Kristanto and Koven 2019; Neo et al. 2021). It is important to acknowledge that the data provided comes with a level of uncertainty as the respondents gave estimates and the waste was not wasted by the enumerators. The benefit of the selected FU is that it facilitates the examination of behavioural and operational practices amenable to change. However, it is not possible to compare the villages based on the volumes of PW handled with the FU selected. This would show which villages should focus on reducing PW generation, which will be an important issue as the Malang District continues to expand (Pambudi et al. 2024). Additionally, this FU does not show what happens with other types of waste generated by stakeholders, such as metals and organic waste.

Another limitation of the simulation is that the activities in most cases are not specific to Indonesia (e.g., the location of open burning is Global), meaning that there may be oversights of the specific emissions. However, these estimates tend to be conservative, so they might result in higher results.

## Conclusion

PW management in Indonesia presents a complex challenge, driven by increasing domestic waste generation, substantial imports of PW, and a waste management infrastructure that remains insufficiently developed. A key concern is the mismanagement of waste that remains outside formal systems, with practices such as open burning and illegal dumping contributing to significant environmental degradation and long-term ecological harm. One of the impact indicators that is connected to environmental degradation is the effect on climate change, where open burning and dumping were consistently the biggest contributors. At least 12% of GHG emissions were linked to these practices directly (in villages where they were reported), but this percentage becomes closer to 40% when considering that the GWP100 is implicitly captured in ‘Other’ EoL. The practices of open burning and dumping are particularly difficult to reverse due to their diffuse nature and persistence within ecosystems.

The frequent classification of waste under ambiguous categories such as ‘Other’ by stakeholders suggests a lack of systematic monitoring and oversight. This is evident across several villages, such as Krebet Senggrong and Sumberejo, where 75% or more of the PW undergoes an uncertain EoL. This data uncertainty impedes accurate life cycle inventory development and highlights the need for improved waste tracking and stakeholder coordination. Strengthening the capacity of waste collectors through targeted training and promoting extended producer responsibility could enhance system performance and reduce the incidence of unmanaged waste.

Recycling offers environmental benefits and avoids the extraction of fossil fuels for the production of virgin plastics. The increase in recycling would also improve air quality, which is a public health concern, and potentially generate employment opportunities. However, its implementation must be accompanied by effective controls on emissions, particularly those contributing to particulate matter formation. Despite showing an improvement of a minimum of 239% when compared to the baseline village scenario (except Tlogorejo, where recycling has 2.5 times the PMFP of the baseline scenario), it has the highest PMFP out of all the possible intervention strategies. Incineration, while technologically advanced and capable of energy recovery, is associated with elevated greenhouse gas emissions and high FFP. Nonetheless, improvements in carbon capture and storage, flue gas treatment, and energy efficiency may enhance its environmental performance over time. When considering this technology, it is important to consider the need to train and build the capacity of the local community so that they can operate incineration facilities safely and efficiently.

Landfilling, when properly maintained, demonstrates relatively lower impacts in air pollution-related categories such as GWP100 and PMFP. However, its limited capacity and potential for long-term emissions from heterogeneous waste streams necessitate cautious use and strict operational standards.

Overall, no single waste management strategy offers a universally optimal solution. A multi-faceted approach, integrating formal and informal systems, improving monitoring, and combining recycling, controlled incineration, and regulated landfilling, can significantly enhance the environmental performance and resilience of PW management systems in Malang and similar contexts.

## Supplementary Information

Below is the link to the electronic supplementary material.

Supplementary Material 1

## Data Availability

The data that support the findings of this study are available within the article of this study and its supplementary materials, and additional information is available from the corresponding author upon request.

## Abbreviations

EoL: End of Life
EPS: Expanded Polystyrene
FFP: Fossil Fuel Potential
FU: Functional Unit
GHG: Greenhouse Gases
GWP100: Global Warming Potential over 100 years
HDPE: High-Density Polyethylene
LCA: Life Cycle Assessment
LCI: Life Cycle Inventory
LDPE: Low-Density Polyethylene
PM: Particulate Matter, diameter not specified
PM_2.5_: Particulate Matter, 2.5 mm diameter
PMFP: Particulate Matter Formation Potential
PP: Polypropylene
PS: Polystyrene
PVC: Polyvinylchloride
PW: Plastic Waste
