# Supplementary material for: Environmental evaluation of plastic waste end-of-life pathways in Malang District, Indonesia: a life cycle approach
Source: Int J Life Cycle Assess. 2026 Sep 29;31(10):184. doi: 10.1007/s11367-026-02751-9 (PMC13623790; doi:10.1007/s11367-026-02751-9)
Supplement: Supplementary file 1 [file 11367_2026_2751_MOESM1_ESM.docx]

Environmental Evaluation of Plastic Waste Management in Malang District, Indonesia: A Life Cycle Approach

# -Supplementary Materials-

Alexandra Olid Stepanchuk, Sekar Aqila Salsabilla, Serius Miliyani Dwi Putri, Mochamad Chaniezul Ulum, Sujarwoto Sujarwoto, Jeremy Woods, Kristine Belesova, Christopher Millett, Onesmus Mwabonje.

# 1. Additional Figures

|  | HDPE | LDPE | PP | PET | PVC | PS | EPS | OTHER |
| --- | --- | --- | --- | --- | --- | --- | --- | --- |
| National Average | 0.152482 | 0.187943 | 0.310284 | 0.120567 | 0 | 0 | 0 | 0.228723 |
| Bakalan | 0.190159 | 0.324359 | 0.115996 | 0.172752 | 0.0826207 | 0.0536834 | 0.0300917 | 0.00441548 |
| Bedali | 0 | 0 | 1 | 0 | 0 | 0 | 0 | 0 |
| Gampingan | 0.0130897 | 0.0160622 | 0.0956913 | 0.0843196 | 0.000299973 | 0.000272702 | 0.000545405 | 0.000627216 |
| Krebbet Sanggrong | 0.125509 | 0.267901 | 0.16013 | 0.312343 | 0.04786 | 0.0372761 | 0.0413748 | 0.00366914 |
| Krebet | 0.119087 | 0.423113 | 0.128615 | 0.194578 | 0.0354314 | 0.0212994 | 0.0785096 | 0 |
| Mangunrejo | 6.25253e-05 | 0.000247532 | 0.999777 | 0 | 0 | 0 | 0 | 0 |
| Sumberejo | 0.0120434 | 0.421017 | 0.0268565 | 0.448634 | 0.0120664 | 0.0174348 | 0.0118834 | 0.0501772 |
| Rekesan | 0 | 1 | 0 | 0 | 0 | 0 | 0 | 0 |
| Tegalsari | 0.264773 | 0 | 0.177273 | 0.432386 | 0 | 0 | 0 | 0.0232955 |
| Tlogorejo | 0.22951 | 0.208609 | 0.0818941 | 0.27756 | 0.00217758 | 0.137468 | 0.0445481 | 0.00127333 |
| Pagak | 0.0616002 | 0.372539 | 0.200376 | 0.182799 | 0.00462674 | 0.165121 | 0.0129387 | 0 |

Fig S1: Proportions of Plastic

|  | Recycled for plastic pellets | Incinerated | Landfilled | Open dumped | Open burned | Thrown in a river/creek | Public Waste Collector | Informal Waste Collector | Others |
| --- | --- | --- | --- | --- | --- | --- | --- | --- | --- |
| National Average | 0.056 | 0 | 0.2641 | 0.3169 | 0.3599 | 0.0124 | 0 | 0 | 0 |
| Bakalan | 0.00573044 | 0 | 0 | 0 | 0.0296849 | 0.00935721 | 0.0800046 | 0.025813 | 0.84941 |
| Bedali | 0.166667 |  | 0.833333 | 0 | 0 | 0 | 0 | 0 | 0 |
| Gampingan | 0.00899918 | 0 | 0.991001 | 0 | 0 | 0 | 0 | 0 |  |
| Krebet Senggrong | 0 | 0 | 0 | 0.266553 | 0.349922 | 0 | 0.272633 | 0.110632 |  |
| Krebet | 0.117883 | 0 | 0 | 0.00457388 | 0.0438896 | 0 | 0.010023 | 0.0190464 | 0.805216 |
| Mangunrejo | 0.1427 | 0 | 0.8573 | 0 | 0 | 0 | 0 | 0 | 0 |
| Sumberejo | 0.00548989 | 0 | 0.00925269 | 0.00757077 | 0.0617233 | 0 | 0.00784014 | 0.0132101 | 0.895025 |
| Rekasan | 0 | 0 | 1 | 0 | 0 | 0 | 0 | 0 | 0 |
| Tegalsari | 0.00454545 | 0 | 0 | 0 | 0.00454545 | 0 | 0 |  | 0.990909 |
| Tlogorejo | 0.0840102 | 0 | 0.0108479 | 0.0534707 | 0.0189213 | 0.0296625 | 0 | 0.0138159 | 0.789271 |
| Pagak | 0 | 0 | 0 | 0.192 | 0.116 | 0.4 | 0.294 | 0 | 0 |

Fig S2: Proportions of Plastic Waste Management options

Ecoinvent geographical code abbreviations

| GLO | Global |
| --- | --- |
| RoW | Rest of World |
| ID | Indonesia |
|  |  |
|  |  |
|  |  |

# 2. Life Cycle Inventory

## 2.1. Current Scenarios

Table S1: National Average Current Scenario

| No. | Description | Input | Output | Unit | Activity(ies) Name in Ecoinvent v3.10 Database | Comment |
| --- | --- | --- | --- | --- | --- | --- |
| 1 | Recycling of Plastic | 0.056 |  | kg | -0.01 kg of plastic flake, consumer electronics, unsorted  0.1915kWh market for electricity, low voltage (ID)  0.00778 kg market for liquefied petroleum gas (RoW)  1.01 kg of plastic flake, consumer for recycling (GLO) | From Neo et al 2021 |
| 2 | Incineration of Polyethylene |  |  | kg | treatment of waste polyethylene, municipal incineration (GLO) |  |
| 3 | Incineration of Polypropylene |  |  | kg | treatment of waste polypropylene, municipal incineration (GLO) |  |
| 4 | Incineration of Polyethylene Terephthalate |  |  | kg | treatment of waste polyethylene terephthalate, municipal incineration (GLO) |  |
| 5 | Incineration of Polyvinylchloride |  |  | kg | treatment of waste polyvinylchloride, municipal incineration (GLO) |  |
| 6 | Incineration of Polystyrene |  |  | kg | treatment of waste polystyrene, municipal incineration (GLO) |  |
| 7 | Incineration of Not specified Plastic |  |  | kg | treatment of waste plastic, mixture, municipal incineration (GLO) |  |
| 8 | Landfilling of Polyethylene | -0.09 |  | kg | treatment of waste polyethylene, sanitary landfill (RoW) |  |
| 9 | Landfilling of Polypropylene | -0.08 |  | kg | treatment of waste polypropylene, sanitary landfill (RoW) |  |
| 10 | Landfilling of Polyethylene Terephthalate | -0.03 |  | kg | treatment of waste polyethylene terephthalate, sanitary landfill (RoW) |  |
| 11 | Landfilling of Polyvinylchloride | 0 |  | kg | treatment of waste polyvinylchloride, sanitary landfill (RoW) |  |
| 12 | Landfilling of Polystyrene | 0 |  | kg | treatment of waste polystyrene, sanitary landfill (RoW) |  |
| 13 | Landfilling of Not specified Plastics | -0.06 |  | kg | treatment of waste plastic, mixture, sanitary landfill (RoW) |  |
| 14 | Open dumping (on land) of Polyethylene | -0.1 |  | kg | treatment of waste polyethylene, open dump, moist infiltration class (300mm) (GLO) |  |
| 15 | Open dumping (on land) of Polypropylene | -0.09 |  | kg | treatment of waste polypropylene, open dump, moist infiltration class (300mm) (GLO) |  |
| 16 | Open dumping (on land) of Polyethylene Terephthalate | -0.038 |  | kg | treatment of waste polyethylene terephthalate, open dump, moist infiltration class (300mm) (GLO) |  |
| 17 | Open dumping (on land) of Polyvinylchloride | 0 |  | kg | treatment of waste polyvinylchloride, open dump, moist infiltration class (300mm) (GLO) |  |
| 18 | Open dumping (on land) of Polystyrene | 0 |  | kg | treatment of waste polystyrene, open dump, moist infiltration class (300mm) (GLO) |  |
| 19 | Open dumping (on land) of Not specified Plastics | -0.07248 |  | kg | treatment of waste plastic, mixture, open dump, moist infiltration class (300mm) (GLO) |  |
| 20 | Open burning of Polyethylene | -0.1225 |  | kg | treatment of waste polyethylene, open burning (GLO) |  |
| 21 | Open burning of Polypropylene | -0.117 |  | kg | treatment of waste polypropylene, open burning (GLO) |  |
| 22 | Open burning of Polyethylene Terephthalate | -0.043 |  | kg | treatment of waste polyethylene terephthalate, open burning (GLO) |  |
| 23 | Open burning of Polyvinylchloride | 0 |  | kg | treatment of waste polyvinylchloride, open burning (GLO) |  |
| 24 | Open burning of Polystyrene | 0 |  | kg | treatment of waste polystyrene, open burning (GLO) |  |
| 25 | Open burning of Not specified Plastics | -0.082 |  | kg | treatment of waste plastic, mixture, open burning (GLO) |  |
| 26 | Open dump (in water) of Polyethylene | -0.0042 |  | kg | treatment of waste polyethylene, open dump, very wet infiltration class (1000mm) (GLO) |  |
| 30 | Open dump (in water) of Polypropylene | -0.0038 |  | kg | treatment of waste polypropylene, open dump, very wet infiltration class (1000mm) (GLO) |  |
| 31 | Open dump (in water) of Polyethylene Terephthalate | -0.00015 |  | kg | treatment of waste polyethylene terephthalate, open dump, very wet infiltration class (1000mm) (GLO) |  |
| 32 | Open dump (in water) of Polyvinylchloride | 0 |  | kg | treatment of waste polyvinylchloride, open dump, very wet infiltration class (1000mm) (GLO) |  |
| 33 | Open dump (in water) of Polystyrene | 0 |  | kg | treatment of waste polystyrene, open dump, very wet infiltration class (1000mm) (GLO) |  |
| 34 | Open dump (in water) of Not specified Plastics | -0.0028 |  | kg | treatment of waste plastic, mixture, open dump, very wet infiltration class (1000mm) (GLO) |  |
| 35 | Polyethylene Collected by Formal Waste Collectors | 0 |  | kg | 79% treatment of waste polyethylene, sanitary landfill (RoW)  16% treatment of waste polyethylene, open dump, very wet infiltration class (300mm) (GLO)  4% treatment of waste polyethylene, open dump, very wet infiltration class (1000mm) (GLO) |  |
| 36 | Polypropylene Collected by Formal Waste Collectors | 0 |  | kg | 79% treatment of waste polypropylene, sanitary landfill (RoW)  16% treatment of waste polypropylene, open dump, very wet infiltration class (300mm) (GLO)  4% treatment of waste polypropylene, open dump, very wet infiltration class (1000mm) (GLO) |  |
| 37 | Polyethylene Terephthalate Collected by Formal Waste Collectors | 0 |  | kg | 79% treatment of waste polyethylene terephthalate, sanitary landfill (RoW)  16% treatment of waste polyethylene terephthalate, open dump, very wet infiltration class (300mm) (GLO)  4% treatment of waste polyethylene terephthalate, open dump, very wet infiltration class (1000mm) (GLO) |  |
| 38 | Polyvinylchloride Collected by Formal Waste Collectors | 0 |  | kg | 79% treatment of waste polyvinylchloride, sanitary landfill (RoW)  16% treatment of waste polyvinylchloride, open dump, very wet infiltration class (300mm) (GLO)  4% treatment of waste polyvinylchloride, open dump, very wet infiltration class (1000mm) (GLO) |  |
| 39 | Polystyrene Collected by Formal Waste Collectors | 0 |  | kg | 79% treatment of waste polystyrene, sanitary landfill (RoW)  16% treatment of waste polystyrene, open dump, very wet infiltration class (300mm) (GLO)  4% treatment of waste polystyrene, open dump, very wet infiltration class (1000mm) (GLO) |  |
| 40 | Not specified Plastics Collected by Formal Waste Collectors | 0 |  | kg | 79% treatment of waste plastic, mixture, sanitary landfill (RoW)  16% treatment of waste plastic, mixture, open dump, very wet infiltration class (300mm) (GLO)  4% treatment of waste plastic, mixture, open dump, very wet infiltration class (1000mm) (GLO) |  |
| 41 | Polyethylene Collected by Informal Waste Collectors | 0 |  | kg | 70% plastic, recycled  24% treatment of waste polyethylene, sanitary landfill (RoW)  4.8% treatment of waste polyethylene, open dump, very wet infiltration class (300mm) (GLO)  1.2% treatment of waste polyethylene, open dump, very wet infiltration class (1000mm) (GLO) | Refer to Item 1 |
| 42 | Polypropylene Collected by Informal Waste Collectors | 0 |  | kg | 70% plastic, recycled  24% treatment of waste polypropylene, sanitary landfill (RoW)  4.8% treatment of waste polypropylene, open dump, very wet infiltration class (300mm) (GLO)  1.2% treatment of waste polypropylene, open dump, very wet infiltration class (1000mm) (GLO) | Refer to Item 1 |
| 43 | Polyethylene Terephthalate Collected by Informal Waste Collectors | 0 |  | kg | 70% plastic, recycled  24% treatment of waste polyethylene terephthalate, sanitary landfill (RoW)  4.8% treatment of waste polyethylene terephthalate, open dump, very wet infiltration class (300mm) (GLO)  1.2% treatment of waste polyethylene, open dump, very wet infiltration class (1000mm) (GLO) | Refer to Item 1 |
| 44 | Polyvinylchloride Collected by Informal Waste Collectors | 0 |  | kg | 70% plastic, recycled  24% treatment of waste polyvinylchloride, sanitary landfill (RoW)  4.8% treatment of waste polyvinylchloride, open dump, very wet infiltration class (300mm) (GLO)  1.2% treatment of waste polyvinylchloride, open dump, very wet infiltration class (1000mm) (GLO) | Refer to Item 1 |
| 45 | Polystyrene Collected by Informal Waste Collectors | 0 |  | kg | 70% plastic, recycled  24% treatment of waste polystyrene, sanitary landfill (RoW)  4.8% treatment of waste polystyrene, open dump, very wet infiltration class (300mm) (GLO)  1.2% treatment of waste polystyrene, open dump, very wet infiltration class (1000mm) (GLO) | Refer to Item 1 |
| 46 | Not specified Plastics Collected by Informal Waste Collectors | 0 |  | kg | 70% plastic, recycled  24% treatment of waste plastic, mixture, sanitary landfill (RoW)  4.8% treatment of waste plastic, mixture, open dump, very wet infiltration class (300mm) (GLO)  1.2% treatment of waste plastic mixture, open dump, very wet infiltration class (1000mm) (GLO) | Refer to Item 1 |
| 47 | Polyethylene Other End-of-Life | 0 |  | kg | 31% Polyethylene Collected by Formal Waste Collectors  8% Polyethylene Collected by Formal Waste Collectors  61% Uncollected Waste (59% treatment of waste polyethylene, open burning (GLO)  32.8% treatment of waste polyethylene, open dump, very wet infiltration class (300mm) (GLO)  8.2% treatment of polyethylene, open dump, very wet infiltration class (1000mm) (GLO)) | Refer to items 35 &41 |
| 48 | Polypropylene Other End-of-Life | 0 |  | kg | 31% Polypropylene Collected by Formal Waste Collectors  8% Polypropylene Collected by Formal Waste Collectors  61% Uncollected Waste (59% treatment of waste polypropylene, open burning (GLO)  32.8% treatment of waste polypropylene, open dump, very wet infiltration class (300mm) (GLO)  8.2% treatment of polypropylene, open dump, very wet infiltration class (1000mm) (GLO)) | Refer to items 36 &42 |
| 49 | Polyethylene Terephthalate Other End-of-Life | 0 |  | kg | 31% Polyethylene Terephthalate Collected by Formal Waste Collectors  8% Polyethylene Terephthalate Collected by Formal Waste Collectors  61% Uncollected Waste (59% treatment of waste polyethylene terephthalate, open burning (GLO)  32.8% treatment of waste polyethylene terephthalate, open dump, very wet infiltration class (300mm) (GLO)  8.2% treatment of polyethylene terephthalate, open dump, very wet infiltration class (1000mm) (GLO)) | Refer to items 37 &43 |
| 50 | Polyvinylchloride Other End-of-Life | 0 |  | kg | 31% Polyvinylchloride Collected by Formal Waste Collectors  8% Polyvinylchloride Collected by Formal Waste Collectors  61% Uncollected Waste (59% treatment of waste polyvinylchloride, open burning (GLO)  32.8% treatment of waste polyvinylchloride, open dump, very wet infiltration class (300mm) (GLO)  8.2% treatment of polyvinylchloride, open dump, very wet infiltration class (1000mm) (GLO)) | Refer to items 38 &44 |
| 51 | Polystyrene Other End-of-Life | 0 |  | kg | 31% Polystyrene Collected by Formal Waste Collectors  8% Polystyrene Collected by Formal Waste Collectors  61% Uncollected Waste (59% treatment of waste polystyrene, open burning (GLO)  32.8% treatment of waste polystyrene, open dump, very wet infiltration class (300mm) (GLO)  8.2% treatment of polystyrene, open dump, very wet infiltration class (1000mm) (GLO)) | Refer to items 39 &45 |
| 52 | Not specified Plastics Other End-of-Life | 0 |  | kg | 31% Not specified Plastic Collected by Formal Waste Collectors  8% Not specified Plastic Collected by Formal Waste Collectors  61% Uncollected Waste (59% treatment of waste plastic, mixture, open burning (GLO)  32.8% treatment of waste plastic, mixture, open dump, very wet infiltration class (300mm) (GLO)  8.2% treatment of plastic, mixture, open dump, very wet infiltration class (1000mm) (GLO)) | Refer to items 40 &46 |

Table S2: Bakalan Current Scenario

| No. | Description | Input | Output | Unit | Activity(ies) Name in Ecoinvent v3.10 Database | Comment |
| --- | --- | --- | --- | --- | --- | --- |
| 1 | Recycling of Plastic | 0.0057 |  | kg | -0.01 kg of plastic flake, consumer electronics, unsorted  0.1915kWh market for electricity, low voltage (ID)  00.00778 kg market for liquefied petroleum gas (RoW)  1.01 kg of plastic flake, consumer for recycling (GLO) | From Neo et al 2021 |
| 2 | Incineration of Polyethylene | 0 |  | kg | treatment of waste polyethylene, municipal incineration (GLO) |  |
| 3 | Incineration of Polypropylene | 0 |  | kg | treatment of waste polypropylene, municipal incineration (GLO) |  |
| 4 | Incineration of Polyethylene Terephthalate | 0 |  | kg | treatment of waste polyethylene terephthalate, municipal incineration (GLO) |  |
| 5 | Incineration of Polyvinylchloride | 0 |  | kg | treatment of waste polyvinylchloride, municipal incineration (GLO) |  |
| 6 | Incineration of Polystyrene | 0 |  | kg | treatment of waste polystyrene, municipal incineration (GLO) |  |
| 7 | Incineration of Not specified Plastic | 0 |  | kg | treatment of waste plastic, mixture, municipal incineration (GLO) |  |
| 8 | Landfilling of Polyethylene | 0 |  | kg | treatment of waste polyethylene, sanitary landfill (RoW) |  |
| 9 | Landfilling of Polypropylene | 0 |  | kg | treatment of waste polypropylene, sanitary landfill (RoW) |  |
| 10 | Landfilling of Polyethylene Terephthalate | 0 |  | kg | treatment of waste polyethylene terephthalate, sanitary landfill (RoW) |  |
| 11 | Landfilling of Polyvinylchloride | 0 |  | kg | treatment of waste polyvinylchloride, sanitary landfill (RoW) |  |
| 12 | Landfilling of Polystyrene | 0 |  | kg | treatment of waste polystyrene, sanitary landfill (RoW) |  |
| 13 | Landfilling of Not specified Plastics | 0 |  | kg | treatment of waste plastic, mixture, sanitary landfill (RoW) |  |
| 14 | Open dumping (on land) of Polyethylene | 0 |  | kg | treatment of waste polyethylene, open dump, moist infiltration class (300mm) (GLO) |  |
| 15 | Open dumping (on land) of Polypropylene | 0 |  | kg | treatment of waste polypropylene, open dump, moist infiltration class (300mm) (GLO) |  |
| 16 | Open dumping (on land) of Polyethylene Terephthalate | 0 |  | kg | treatment of waste polyethylene terephthalate, open dump, moist infiltration class (300mm) (GLO) |  |
| 17 | Open dumping (on land) of Polyvinylchloride | 0 |  | kg | treatment of waste polyvinylchloride, open dump, moist infiltration class (300mm) (GLO) |  |
| 18 | Open dumping (on land) of Polystyrene | 0 |  | kg | treatment of waste polystyrene, open dump, moist infiltration class (300mm) (GLO) |  |
| 19 | Open dumping (on land) of Not specified Plastics | 0 |  | kg | treatment of waste plastic, mixture, open dump, moist infiltration class (300mm) (GLO) |  |
| 20 | Open burning of Polyethylene | -0.0176 |  | kg | treatment of waste polyethylene, open burning (GLO) |  |
| 21 | Open burning of Polypropylene | -0.00061 |  | kg | treatment of waste polypropylene, open burning (GLO) |  |
| 22 | Open burning of Polyethylene Terephthalate | -0.0054 |  | kg | treatment of waste polyethylene terephthalate, open burning (GLO) |  |
| 23 | Open burning of Polyvinylchloride | -0.00125 |  | kg | treatment of waste polyvinylchloride, open burning (GLO) |  |
| 24 | Open burning of Polystyrene | -0.0048 |  | kg | treatment of waste polystyrene, open burning (GLO) |  |
| 25 | Open burning of Not specified Plastics | 0 |  | kg | treatment of waste plastic, mixture, open burning (GLO) |  |
| 26 | Open dump (in water) of Polyethylene | -0.0056 |  | kg | treatment of waste polyethylene, open dump, very wet infiltration class (1000mm) (GLO) |  |
| 30 | Open dump (in water) of Polypropylene | -0.00019 |  | kg | treatment of waste polypropylene, open dump, very wet infiltration class (1000mm) (GLO) |  |
| 31 | Open dump (in water) of Polyethylene Terephthalate | -0.0017 |  | kg | treatment of waste polyethylene terephthalate, open dump, very wet infiltration class (1000mm) (GLO) |  |
| 32 | Open dump (in water) of Polyvinylchloride | -0.000394 |  | kg | treatment of waste polyvinylchloride, open dump, very wet infiltration class (1000mm) (GLO) |  |
| 33 | Open dump (in water) of Polystyrene | -0.0015 |  | kg | treatment of waste polystyrene, open dump, very wet infiltration class (1000mm) (GLO) |  |
| 34 | Open dump (in water) of Not specified Plastics | 0 |  | kg | treatment of waste plastic, mixture, open dump, very wet infiltration class (1000mm) (GLO) |  |
| 35 | Polyethylene Collected by Formal Waste Collectors | 0.044 |  | kg | 79% treatment of waste polyethylene, sanitary landfill (RoW)  16% treatment of waste polyethylene, open dump, very wet infiltration class (300mm) (GLO)  4% treatment of waste polyethylene, open dump, very wet infiltration class (1000mm) (GLO) |  |
| 36 | Polypropylene Collected by Formal Waste Collectors | 0.0014 |  | kg | 79% treatment of waste polypropylene, sanitary landfill (RoW)  16% treatment of waste polypropylene, open dump, very wet infiltration class (300mm) (GLO)  4% treatment of waste polypropylene, open dump, very wet infiltration class (1000mm) (GLO) |  |
| 37 | Polyethylene Terephthalate Collected by Formal Waste Collectors | 0.019 |  | kg | 79% treatment of waste polyethylene terephthalate, sanitary landfill (RoW)  16% treatment of waste polyethylene terephthalate, open dump, very wet infiltration class (300mm) (GLO)  4% treatment of waste polyethylene terephthalate, open dump, very wet infiltration class (1000mm) (GLO) |  |
| 38 | Polyvinylchloride Collected by Formal Waste Collectors | 0.00284 |  | kg | 79% treatment of waste polyvinylchloride, sanitary landfill (RoW)  16% treatment of waste polyvinylchloride, open dump, very wet infiltration class (300mm) (GLO)  4% treatment of waste polyvinylchloride, open dump, very wet infiltration class (1000mm) (GLO) |  |
| 39 | Polystyrene Collected by Formal Waste Collectors | 0.013 |  | kg | 79% treatment of waste polystyrene, sanitary landfill (RoW)  16% treatment of waste polystyrene, open dump, very wet infiltration class (300mm) (GLO)  4% treatment of waste polystyrene, open dump, very wet infiltration class (1000mm) (GLO) |  |
| 40 | Not specified Plastics Collected by Formal Waste Collectors | 0 |  | kg | 79% treatment of waste plastic, mixture, sanitary landfill (RoW)  16% treatment of waste plastic, mixture, open dump, very wet infiltration class (300mm) (GLO)  4% treatment of waste plastic, mixture, open dump, very wet infiltration class (1000mm) (GLO) |  |
| 41 | Polyethylene Collected by Informal Waste Collectors | 0.015 |  | kg | 70% plastic, recycled  24% treatment of waste polyethylene, sanitary landfill (RoW)  4.8% treatment of waste polyethylene, open dump, very wet infiltration class (300mm) (GLO)  1.2% treatment of waste polyethylene, open dump, very wet infiltration class (1000mm) (GLO) | Refer to Item 1 |
| 42 | Polypropylene Collected by Informal Waste Collectors | 0.00052 |  | kg | 70% plastic, recycled  24% treatment of waste polypropylene, sanitary landfill (RoW)  4.8% treatment of waste polypropylene, open dump, very wet infiltration class (300mm) (GLO)  1.2% treatment of waste polypropylene, open dump, very wet infiltration class (1000mm) (GLO) | Refer to Item 1 |
| 43 | Polyethylene Terephthalate Collected by Informal Waste Collectors | 0.0047 |  | kg | 70% plastic, recycled  24% treatment of waste polyethylene terephthalate, sanitary landfill (RoW)  4.8% treatment of waste polyethylene terephthalate, open dump, very wet infiltration class (300mm) (GLO)  1.2% treatment of waste polyethylene, open dump, very wet infiltration class (1000mm) (GLO) | Refer to Item 1 |
| 44 | Polyvinylchloride Collected by Informal Waste Collectors | 0.011 |  | kg | 70% plastic, recycled  24% treatment of waste polyvinylchloride, sanitary landfill (RoW)  4.8% treatment of waste polyvinylchloride, open dump, very wet infiltration class (300mm) (GLO)  1.2% treatment of waste polyvinylchloride, open dump, very wet infiltration class (1000mm) (GLO) | Refer to Item 1 |
| 45 | Polystyrene Collected by Informal Waste Collectors | 0.0042 |  | kg | 70% plastic, recycled  24% treatment of waste polystyrene, sanitary landfill (RoW)  4.8% treatment of waste polystyrene, open dump, very wet infiltration class (300mm) (GLO)  1.2% treatment of waste polystyrene, open dump, very wet infiltration class (1000mm) (GLO) | Refer to Item 1 |
| 46 | Not specified Plastics Collected by Informal Waste Collectors | 0 |  | kg | 70% plastic, recycled  24% treatment of waste plastic, mixture, sanitary landfill (RoW)  4.8% treatment of waste plastic, mixture, open dump, very wet infiltration class (300mm) (GLO)  1.2% treatment of waste plastic mixture, open dump, very wet infiltration class (1000mm) (GLO) | Refer to Item 1 |
| 47 | Polyethylene Other End-of-Life | 0.427 |  | kg | 31% Polyethylene Collected by Formal Waste Collectors  8% Polyethylene Collected by Formal Waste Collectors  61% Uncollected Waste (59% treatment of waste polyethylene, open burning (GLO)  32.8% treatment of waste polyethylene, open dump, very wet infiltration class (300mm) (GLO)  8.2% treatment of polyethylene, open dump, very wet infiltration class (1000mm) (GLO)) | Refer to items 35 &41 |
| 48 | Polypropylene Other End-of-Life | 0.113 |  | kg | 31% Polypropylene Collected by Formal Waste Collectors  8% Polypropylene Collected by Formal Waste Collectors  61% Uncollected Waste (59% treatment of waste polypropylene, open burning (GLO)  32.8% treatment of waste polypropylene, open dump, very wet infiltration class (300mm) (GLO)  8.2% treatment of polypropylene, open dump, very wet infiltration class (1000mm) (GLO)) | Refer to items 36 &42 |
| 49 | Polyethylene Terephthalate Other End-of-Life | 0.141 |  | kg | 31% Polyethylene Terephthalate Collected by Formal Waste Collectors  8% Polyethylene Terephthalate Collected by Formal Waste Collectors  61% Uncollected Waste (59% treatment of waste polyethylene terephthalate, open burning (GLO)  32.8% treatment of waste polyethylene terephthalate, open dump, very wet infiltration class (300mm) (GLO)  8.2% treatment of polyethylene terephthalate, open dump, very wet infiltration class (1000mm) (GLO)) | Refer to items 37 &43 |
| 50 | Polyvinylchloride Other End-of-Life | 0.077 |  | kg | 31% Polyvinylchloride Collected by Formal Waste Collectors  8% Polyvinylchloride Collected by Formal Waste Collectors  61% Uncollected Waste (59% treatment of waste polyvinylchloride, open burning (GLO)  32.8% treatment of waste polyvinylchloride, open dump, very wet infiltration class (300mm) (GLO)  8.2% treatment of polyvinylchloride, open dump, very wet infiltration class (1000mm) (GLO)) | Refer to items 38 &44 |
| 51 | Polystyrene Other End-of-Life | 0.06 |  | kg | 31% Polystyrene Collected by Formal Waste Collectors  8% Polystyrene Collected by Formal Waste Collectors  61% Uncollected Waste (59% treatment of waste polystyrene, open burning (GLO)  32.8% treatment of waste polystyrene, open dump, very wet infiltration class (300mm) (GLO)  8.2% treatment of polystyrene, open dump, very wet infiltration class (1000mm) (GLO)) | Refer to items 39 &45 |
| 52 | Not specified Plastics Other End-of-Life | 0.0044 |  | kg | 31% Not specified Plastic Collected by Formal Waste Collectors  8% Not specified Plastic Collected by Formal Waste Collectors  61% Uncollected Waste (59% treatment of waste plastic, mixture, open burning (GLO)  32.8% treatment of waste plastic, mixture, open dump, very wet infiltration class (300mm) (GLO)  8.2% treatment of plastic, mixture, open dump, very wet infiltration class (1000mm) (GLO)) | Refer to items 40 &46 |
| 53 | Transport of plastic waste with scooter | 0.0045 |  | Person kilometer | Transport, passenger, motor scooter (RoW) |  |
| 54 | Avoided Polyethylene Production | -0.0057 |  | kg | market for polyethylene, high density granulate (GLO) |  |

Table S3: Bedali Current Scenario

| No. | Description | Input | Output | Unit | Activity(ies) Name in Ecoinvent v3.10 Database | Comment |
| --- | --- | --- | --- | --- | --- | --- |
| 1 | Recycling of Plastic | 0.167 |  | kg | -0.01 kg of plastic flake, consumer electronics, unsorted  0.1915kWh market for electricity, low voltage (ID)  0.00778 kg market for liquefied petroleum gas (RoW)  1.01 kg of plastic flake, consumer for recycling (GLO) | From Neo et al 2021 |
| 2 | Incineration of Polyethylene | 0 |  | kg | treatment of waste polyethylene, municipal incineration (GLO) |  |
| 3 | Incineration of Polypropylene | 0 |  | kg | treatment of waste polypropylene, municipal incineration (GLO) |  |
| 4 | Incineration of Polyethylene Terephthalate | 0 |  | kg | treatment of waste polyethylene terephthalate, municipal incineration (GLO) |  |
| 5 | Incineration of Polyvinylchloride | 0 |  | kg | treatment of waste polyvinylchloride, municipal incineration (GLO) |  |
| 6 | Incineration of Polystyrene | 0 |  | kg | treatment of waste polystyrene, municipal incineration (GLO) |  |
| 7 | Incineration of Not specified Plastic | 0 |  | kg | treatment of waste plastic, mixture, municipal incineration (GLO) |  |
| 8 | Landfilling of Polyethylene | 0 |  | kg | treatment of waste polyethylene, sanitary landfill (RoW) |  |
| 9 | Landfilling of Polypropylene | 0 |  | kg | treatment of waste polypropylene, sanitary landfill (RoW) |  |
| 10 | Landfilling of Polyethylene Terephthalate | 0 |  | kg | treatment of waste polyethylene terephthalate, sanitary landfill (RoW) |  |
| 11 | Landfilling of Polyvinylchloride | 0 |  | kg | treatment of waste polyvinylchloride, sanitary landfill (RoW) |  |
| 12 | Landfilling of Polystyrene | 0 |  | kg | treatment of waste polystyrene, sanitary landfill (RoW) |  |
| 13 | Landfilling of Not specified Plastics | 0 |  | kg | treatment of waste plastic, mixture, sanitary landfill (RoW) |  |
| 14 | Open dumping (on land) of Polyethylene | 0 |  | kg | treatment of waste polyethylene, open dump, moist infiltration class (300mm) (GLO) |  |
| 15 | Open dumping (on land) of Polypropylene | 0 |  | kg | treatment of waste polypropylene, open dump, moist infiltration class (300mm) (GLO) |  |
| 16 | Open dumping (on land) of Polyethylene Terephthalate | 0 |  | kg | treatment of waste polyethylene terephthalate, open dump, moist infiltration class (300mm) (GLO) |  |
| 17 | Open dumping (on land) of Polyvinylchloride | 0 |  | kg | treatment of waste polyvinylchloride, open dump, moist infiltration class (300mm) (GLO) |  |
| 18 | Open dumping (on land) of Polystyrene | 0 |  | kg | treatment of waste polystyrene, open dump, moist infiltration class (300mm) (GLO) |  |
| 19 | Open dumping (on land) of Not specified Plastics | 0 |  | kg | treatment of waste plastic, mixture, open dump, moist infiltration class (300mm) (GLO) |  |
| 20 | Open burning of Polyethylene | 0 |  | kg | treatment of waste polyethylene, open burning (GLO) |  |
| 21 | Open burning of Polypropylene | 0 |  | kg | treatment of waste polypropylene, open burning (GLO) |  |
| 22 | Open burning of Polyethylene Terephthalate | 0 |  | kg | treatment of waste polyethylene terephthalate, open burning (GLO) |  |
| 23 | Open burning of Polyvinylchloride | 0 |  | kg | treatment of waste polyvinylchloride, open burning (GLO) |  |
| 24 | Open burning of Polystyrene | 0 |  | kg | treatment of waste polystyrene, open burning (GLO) |  |
| 25 | Open burning of Not specified Plastics | 0 |  | kg | treatment of waste plastic, mixture, open burning (GLO) |  |
| 26 | Open dump (in water) of Polyethylene | 0 |  | kg | treatment of waste polyethylene, open dump, very wet infiltration class (1000mm) (GLO) |  |
| 30 | Open dump (in water) of Polypropylene | 0 |  | kg | treatment of waste polypropylene, open dump, very wet infiltration class (1000mm) (GLO) |  |
| 31 | Open dump (in water) of Polyethylene Terephthalate | 0 |  | kg | treatment of waste polyethylene terephthalate, open dump, very wet infiltration class (1000mm) (GLO) |  |
| 32 | Open dump (in water) of Polyvinylchloride | 0 |  | kg | treatment of waste polyvinylchloride, open dump, very wet infiltration class (1000mm) (GLO) |  |
| 33 | Open dump (in water) of Polystyrene | 0 |  | kg | treatment of waste polystyrene, open dump, very wet infiltration class (1000mm) (GLO) |  |
| 34 | Open dump (in water) of Not specified Plastics | 0 |  | kg | treatment of waste plastic, mixture, open dump, very wet infiltration class (1000mm) (GLO) |  |
| 35 | Polyethylene Collected by Formal Waste Collectors | 0.833 |  | kg | 79% treatment of waste polyethylene, sanitary landfill (RoW)  16% treatment of waste polyethylene, open dump, very wet infiltration class (300mm) (GLO)  4% treatment of waste polyethylene, open dump, very wet infiltration class (1000mm) (GLO) |  |
| 36 | Polypropylene Collected by Formal Waste Collectors | 0 |  | kg | 79% treatment of waste polypropylene, sanitary landfill (RoW)  16% treatment of waste polypropylene, open dump, very wet infiltration class (300mm) (GLO)  4% treatment of waste polypropylene, open dump, very wet infiltration class (1000mm) (GLO) |  |
| 37 | Polyethylene Terephthalate Collected by Formal Waste Collectors | 0 |  | kg | 79% treatment of waste polyethylene terephthalate, sanitary landfill (RoW)  16% treatment of waste polyethylene terephthalate, open dump, very wet infiltration class (300mm) (GLO)  4% treatment of waste polyethylene terephthalate, open dump, very wet infiltration class (1000mm) (GLO) |  |
| 38 | Polyvinylchloride Collected by Formal Waste Collectors | 0 |  | kg | 79% treatment of waste polyvinylchloride, sanitary landfill (RoW)  16% treatment of waste polyvinylchloride, open dump, very wet infiltration class (300mm) (GLO)  4% treatment of waste polyvinylchloride, open dump, very wet infiltration class (1000mm) (GLO) |  |
| 39 | Polystyrene Collected by Formal Waste Collectors | 0 |  | kg | 79% treatment of waste polystyrene, sanitary landfill (RoW)  16% treatment of waste polystyrene, open dump, very wet infiltration class (300mm) (GLO)  4% treatment of waste polystyrene, open dump, very wet infiltration class (1000mm) (GLO) |  |
| 40 | Not specified Plastics Collected by Formal Waste Collectors | 0 |  | kg | 79% treatment of waste plastic, mixture, sanitary landfill (RoW)  16% treatment of waste plastic, mixture, open dump, very wet infiltration class (300mm) (GLO)  4% treatment of waste plastic, mixture, open dump, very wet infiltration class (1000mm) (GLO) |  |
| 41 | Polyethylene Collected by Informal Waste Collectors | 0 |  | kg | 70% plastic, recycled  24% treatment of waste polyethylene, sanitary landfill (RoW)  4.8% treatment of waste polyethylene, open dump, very wet infiltration class (300mm) (GLO)  1.2% treatment of waste polyethylene, open dump, very wet infiltration class (1000mm) (GLO) | Refer to Item 1 |
| 42 | Polypropylene Collected by Informal Waste Collectors | 0 |  | kg | 70% plastic, recycled  24% treatment of waste polypropylene, sanitary landfill (RoW)  4.8% treatment of waste polypropylene, open dump, very wet infiltration class (300mm) (GLO)  1.2% treatment of waste polypropylene, open dump, very wet infiltration class (1000mm) (GLO) | Refer to Item 1 |
| 43 | Polyethylene Terephthalate Collected by Informal Waste Collectors | 0 |  | kg | 70% plastic, recycled  24% treatment of waste polyethylene terephthalate, sanitary landfill (RoW)  4.8% treatment of waste polyethylene terephthalate, open dump, very wet infiltration class (300mm) (GLO)  1.2% treatment of waste polyethylene, open dump, very wet infiltration class (1000mm) (GLO) | Refer to Item 1 |
| 44 | Polyvinylchloride Collected by Informal Waste Collectors | 0 |  | kg | 70% plastic, recycled  24% treatment of waste polyvinylchloride, sanitary landfill (RoW)  4.8% treatment of waste polyvinylchloride, open dump, very wet infiltration class (300mm) (GLO)  1.2% treatment of waste polyvinylchloride, open dump, very wet infiltration class (1000mm) (GLO) | Refer to Item 1 |
| 45 | Polystyrene Collected by Informal Waste Collectors | 0 |  | kg | 70% plastic, recycled  24% treatment of waste polystyrene, sanitary landfill (RoW)  4.8% treatment of waste polystyrene, open dump, very wet infiltration class (300mm) (GLO)  1.2% treatment of waste polystyrene, open dump, very wet infiltration class (1000mm) (GLO) | Refer to Item 1 |
| 46 | Not specified Plastics Collected by Informal Waste Collectors | 0 |  | kg | 70% plastic, recycled  24% treatment of waste plastic, mixture, sanitary landfill (RoW)  4.8% treatment of waste plastic, mixture, open dump, very wet infiltration class (300mm) (GLO)  1.2% treatment of waste plastic mixture, open dump, very wet infiltration class (1000mm) (GLO) | Refer to Item 1 |
| 47 | Polyethylene Other End-of-Life | 0 |  | kg | 31% Polyethylene Collected by Formal Waste Collectors  8% Polyethylene Collected by Formal Waste Collectors  61% Uncollected Waste (59% treatment of waste polyethylene, open burning (GLO)  32.8% treatment of waste polyethylene, open dump, very wet infiltration class (300mm) (GLO)  8.2% treatment of polyethylene, open dump, very wet infiltration class (1000mm) (GLO)) | Refer to items 35 &41 |
| 48 | Polypropylene Other End-of-Life | 0 |  | kg | 31% Polypropylene Collected by Formal Waste Collectors  8% Polypropylene Collected by Formal Waste Collectors  61% Uncollected Waste (59% treatment of waste polypropylene, open burning (GLO)  32.8% treatment of waste polypropylene, open dump, very wet infiltration class (300mm) (GLO)  8.2% treatment of polypropylene, open dump, very wet infiltration class (1000mm) (GLO)) | Refer to items 36 &42 |
| 49 | Polyethylene Terephthalate Other End-of-Life | 0 |  | kg | 31% Polyethylene Terephthalate Collected by Formal Waste Collectors  8% Polyethylene Terephthalate Collected by Formal Waste Collectors  61% Uncollected Waste (59% treatment of waste polyethylene terephthalate, open burning (GLO)  32.8% treatment of waste polyethylene terephthalate, open dump, very wet infiltration class (300mm) (GLO)  8.2% treatment of polyethylene terephthalate, open dump, very wet infiltration class (1000mm) (GLO)) | Refer to items 37 &43 |
| 50 | Polyvinylchloride Other End-of-Life | 0 |  | kg | 31% Polyvinylchloride Collected by Formal Waste Collectors  8% Polyvinylchloride Collected by Formal Waste Collectors  61% Uncollected Waste (59% treatment of waste polyvinylchloride, open burning (GLO)  32.8% treatment of waste polyvinylchloride, open dump, very wet infiltration class (300mm) (GLO)  8.2% treatment of polyvinylchloride, open dump, very wet infiltration class (1000mm) (GLO)) | Refer to items 38 &44 |
| 51 | Polystyrene Other End-of-Life | 0 |  | kg | 31% Polystyrene Collected by Formal Waste Collectors  8% Polystyrene Collected by Formal Waste Collectors  61% Uncollected Waste (59% treatment of waste polystyrene, open burning (GLO)  32.8% treatment of waste polystyrene, open dump, very wet infiltration class (300mm) (GLO)  8.2% treatment of polystyrene, open dump, very wet infiltration class (1000mm) (GLO)) | Refer to items 39 &45 |
| 52 | Not specified Plastics Other End-of-Life | 0 |  | kg | 31% Not specified Plastic Collected by Formal Waste Collectors  8% Not specified Plastic Collected by Formal Waste Collectors  61% Uncollected Waste (59% treatment of waste plastic, mixture, open burning (GLO)  32.8% treatment of waste plastic, mixture, open dump, very wet infiltration class (300mm) (GLO)  8.2% treatment of plastic, mixture, open dump, very wet infiltration class (1000mm) (GLO)) | Refer to items 40 &46 |
| 53 | Avoided Polystyrene Production | -0.167 |  | kg | Market for polystyrene,general purpose (GLO) |  |

Table S4: Gampingan Current Scenario

| No. | Description | Input | Output | Unit | Activity(ies) Name in Ecoinvent v3.10 Database | Comment |
| --- | --- | --- | --- | --- | --- | --- |
| 1 | Recycling of Plastic | 0.0089 |  | kg | -0.01 kg of plastic flake, consumer electronics, unsorted  0.1915kWh market for electricity, low voltage (ID)  0.00778 kg market for liquefied petroleum gas (RoW)  1.01 kg of plastic flake, consumer for recycling (GLO) | From Neo et al 2021 |
| 2 | Incineration of Polyethylene | 0 |  | kg | treatment of waste polyethylene, municipal incineration (GLO) |  |
| 3 | Incineration of Polypropylene | 0 |  | kg | treatment of waste polypropylene, municipal incineration (GLO) |  |
| 4 | Incineration of Polyethylene Terephthalate | 0 |  | kg | treatment of waste polyethylene terephthalate, municipal incineration (GLO) |  |
| 5 | Incineration of Polyvinylchloride | 0 |  | kg | treatment of waste polyvinylchloride, municipal incineration (GLO) |  |
| 6 | Incineration of Polystyrene | 0 |  | kg | treatment of waste polystyrene, municipal incineration (GLO) |  |
| 7 | Incineration of Not specified Plastic | 0 |  | kg | treatment of waste plastic, mixture, municipal incineration (GLO) |  |
| 8 | Landfilling of Polyethylene | 0 |  | kg | treatment of waste polyethylene, sanitary landfill (RoW) |  |
| 9 | Landfilling of Polypropylene | 0 |  | kg | treatment of waste polypropylene, sanitary landfill (RoW) |  |
| 10 | Landfilling of Polyethylene Terephthalate | 0 |  | kg | treatment of waste polyethylene terephthalate, sanitary landfill (RoW) |  |
| 11 | Landfilling of Polyvinylchloride | 0 |  | kg | treatment of waste polyvinylchloride, sanitary landfill (RoW) |  |
| 12 | Landfilling of Polystyrene | 0 |  | kg | treatment of waste polystyrene, sanitary landfill (RoW) |  |
| 13 | Landfilling of Not specified Plastics | 0 |  | kg | treatment of waste plastic, mixture, sanitary landfill (RoW) |  |
| 14 | Open dumping (on land) of Polyethylene | 0 |  | kg | treatment of waste polyethylene, open dump, moist infiltration class (300mm) (GLO) |  |
| 15 | Open dumping (on land) of Polypropylene | 0 |  | kg | treatment of waste polypropylene, open dump, moist infiltration class (300mm) (GLO) |  |
| 16 | Open dumping (on land) of Polyethylene Terephthalate | 0 |  | kg | treatment of waste polyethylene terephthalate, open dump, moist infiltration class (300mm) (GLO) |  |
| 17 | Open dumping (on land) of Polyvinylchloride | 0 |  | kg | treatment of waste polyvinylchloride, open dump, moist infiltration class (300mm) (GLO) |  |
| 18 | Open dumping (on land) of Polystyrene | 0 |  | kg | treatment of waste polystyrene, open dump, moist infiltration class (300mm) (GLO) |  |
| 19 | Open dumping (on land) of Not specified Plastics | 0 |  | kg | treatment of waste plastic, mixture, open dump, moist infiltration class (300mm) (GLO) |  |
| 20 | Open burning of Polyethylene | 0 |  | kg | treatment of waste polyethylene, open burning (GLO) |  |
| 21 | Open burning of Polypropylene | 0 |  | kg | treatment of waste polypropylene, open burning (GLO) |  |
| 22 | Open burning of Polyethylene Terephthalate | 0 |  | kg | treatment of waste polyethylene terephthalate, open burning (GLO) |  |
| 23 | Open burning of Polyvinylchloride | 0 |  | kg | treatment of waste polyvinylchloride, open burning (GLO) |  |
| 24 | Open burning of Polystyrene | 0 |  | kg | treatment of waste polystyrene, open burning (GLO) |  |
| 25 | Open burning of Not specified Plastics | 0 |  | kg | treatment of waste plastic, mixture, open burning (GLO) |  |
| 26 | Open dump (in water) of Polyethylene | 0 |  | kg | treatment of waste polyethylene, open dump, very wet infiltration class (1000mm) (GLO) |  |
| 30 | Open dump (in water) of Polypropylene | 0 |  | kg | treatment of waste polypropylene, open dump, very wet infiltration class (1000mm) (GLO) |  |
| 31 | Open dump (in water) of Polyethylene Terephthalate | 0 |  | kg | treatment of waste polyethylene terephthalate, open dump, very wet infiltration class (1000mm) (GLO) |  |
| 32 | Open dump (in water) of Polyvinylchloride | 0 |  | kg | treatment of waste polyvinylchloride, open dump, very wet infiltration class (1000mm) (GLO) |  |
| 33 | Open dump (in water) of Polystyrene | 0 |  | kg | treatment of waste polystyrene, open dump, very wet infiltration class (1000mm) (GLO) |  |
| 34 | Open dump (in water) of Not specified Plastics | 0 |  | kg | treatment of waste plastic, mixture, open dump, very wet infiltration class (1000mm) (GLO) |  |
| 35 | Polyethylene Collected by Formal Waste Collectors | 0 |  | kg | 79% treatment of waste polyethylene, sanitary landfill (RoW)  16% treatment of waste polyethylene, open dump, very wet infiltration class (300mm) (GLO)  4% treatment of waste polyethylene, open dump, very wet infiltration class (1000mm) (GLO) |  |
| 36 | Polypropylene Collected by Formal Waste Collectors | 0 |  | kg | 79% treatment of waste polypropylene, sanitary landfill (RoW)  16% treatment of waste polypropylene, open dump, very wet infiltration class (300mm) (GLO)  4% treatment of waste polypropylene, open dump, very wet infiltration class (1000mm) (GLO) |  |
| 37 | Polyethylene Terephthalate Collected by Formal Waste Collectors | 0 |  | kg | 79% treatment of waste polyethylene terephthalate, sanitary landfill (RoW)  16% treatment of waste polyethylene terephthalate, open dump, very wet infiltration class (300mm) (GLO)  4% treatment of waste polyethylene terephthalate, open dump, very wet infiltration class (1000mm) (GLO) |  |
| 38 | Polyvinylchloride Collected by Formal Waste Collectors | 0 |  | kg | 79% treatment of waste polyvinylchloride, sanitary landfill (RoW)  16% treatment of waste polyvinylchloride, open dump, very wet infiltration class (300mm) (GLO)  4% treatment of waste polyvinylchloride, open dump, very wet infiltration class (1000mm) (GLO) |  |
| 39 | Polystyrene Collected by Formal Waste Collectors | 0 |  | kg | 79% treatment of waste polystyrene, sanitary landfill (RoW)  16% treatment of waste polystyrene, open dump, very wet infiltration class (300mm) (GLO)  4% treatment of waste polystyrene, open dump, very wet infiltration class (1000mm) (GLO) |  |
| 40 | Not specified Plastics Collected by Formal Waste Collectors | 0 |  | kg | 79% treatment of waste plastic, mixture, sanitary landfill (RoW)  16% treatment of waste plastic, mixture, open dump, very wet infiltration class (300mm) (GLO)  4% treatment of waste plastic, mixture, open dump, very wet infiltration class (1000mm) (GLO) |  |
| 41 | Polyethylene Collected by Informal Waste Collectors | 0 |  | kg | 70% plastic, recycled  24% treatment of waste polyethylene, sanitary landfill (RoW)  4.8% treatment of waste polyethylene, open dump, very wet infiltration class (300mm) (GLO)  1.2% treatment of waste polyethylene, open dump, very wet infiltration class (1000mm) (GLO) | Refer to Item 1 |
| 42 | Polypropylene Collected by Informal Waste Collectors | 0 |  | kg | 70% plastic, recycled  24% treatment of waste polypropylene, sanitary landfill (RoW)  4.8% treatment of waste polypropylene, open dump, very wet infiltration class (300mm) (GLO)  1.2% treatment of waste polypropylene, open dump, very wet infiltration class (1000mm) (GLO) | Refer to Item 1 |
| 43 | Polyethylene Terephthalate Collected by Informal Waste Collectors | 0 |  | kg | 70% plastic, recycled  24% treatment of waste polyethylene terephthalate, sanitary landfill (RoW)  4.8% treatment of waste polyethylene terephthalate, open dump, very wet infiltration class (300mm) (GLO)  1.2% treatment of waste polyethylene, open dump, very wet infiltration class (1000mm) (GLO) | Refer to Item 1 |
| 44 | Polyvinylchloride Collected by Informal Waste Collectors | 0 |  | kg | 70% plastic, recycled  24% treatment of waste polyvinylchloride, sanitary landfill (RoW)  4.8% treatment of waste polyvinylchloride, open dump, very wet infiltration class (300mm) (GLO)  1.2% treatment of waste polyvinylchloride, open dump, very wet infiltration class (1000mm) (GLO) | Refer to Item 1 |
| 45 | Polystyrene Collected by Informal Waste Collectors | 0 |  | kg | 70% plastic, recycled  24% treatment of waste polystyrene, sanitary landfill (RoW)  4.8% treatment of waste polystyrene, open dump, very wet infiltration class (300mm) (GLO)  1.2% treatment of waste polystyrene, open dump, very wet infiltration class (1000mm) (GLO) | Refer to Item 1 |
| 46 | Not specified Plastics Collected by Informal Waste Collectors | 0 |  | kg | 70% plastic, recycled  24% treatment of waste plastic, mixture, sanitary landfill (RoW)  4.8% treatment of waste plastic, mixture, open dump, very wet infiltration class (300mm) (GLO)  1.2% treatment of waste plastic mixture, open dump, very wet infiltration class (1000mm) (GLO) | Refer to Item 1 |
| 47 | Polyethylene Other End-of-Life | 0.029 |  | kg | 31% Polyethylene Collected by Formal Waste Collectors  8% Polyethylene Collected by Formal Waste Collectors  61% Uncollected Waste (59% treatment of waste polyethylene, open burning (GLO)  32.8% treatment of waste polyethylene, open dump, very wet infiltration class (300mm) (GLO)  8.2% treatment of polyethylene, open dump, very wet infiltration class (1000mm) (GLO)) | Refer to items 35 &41 |
| 48 | Polypropylene Other End-of-Life | 0.087 |  | kg | 31% Polypropylene Collected by Formal Waste Collectors  8% Polypropylene Collected by Formal Waste Collectors  61% Uncollected Waste (59% treatment of waste polypropylene, open burning (GLO)  32.8% treatment of waste polypropylene, open dump, very wet infiltration class (300mm) (GLO)  8.2% treatment of polypropylene, open dump, very wet infiltration class (1000mm) (GLO)) | Refer to items 36 &42 |
| 49 | Polyethylene Terephthalate Other End-of-Life | 0.0843 |  | kg | 31% Polyethylene Terephthalate Collected by Formal Waste Collectors  8% Polyethylene Terephthalate Collected by Formal Waste Collectors  61% Uncollected Waste (59% treatment of waste polyethylene terephthalate, open burning (GLO)  32.8% treatment of waste polyethylene terephthalate, open dump, very wet infiltration class (300mm) (GLO)  8.2% treatment of polyethylene terephthalate, open dump, very wet infiltration class (1000mm) (GLO)) | Refer to items 37 &43 |
| 50 | Polyvinylchloride Other End-of-Life | 0.0003 |  | kg | 31% Polyvinylchloride Collected by Formal Waste Collectors  8% Polyvinylchloride Collected by Formal Waste Collectors  61% Uncollected Waste (59% treatment of waste polyvinylchloride, open burning (GLO)  32.8% treatment of waste polyvinylchloride, open dump, very wet infiltration class (300mm) (GLO)  8.2% treatment of polyvinylchloride, open dump, very wet infiltration class (1000mm) (GLO)) | Refer to items 38 &44 |
| 51 | Polystyrene Other End-of-Life | 0.00082 |  | kg | 31% Polystyrene Collected by Formal Waste Collectors  8% Polystyrene Collected by Formal Waste Collectors  61% Uncollected Waste (59% treatment of waste polystyrene, open burning (GLO)  32.8% treatment of waste polystyrene, open dump, very wet infiltration class (300mm) (GLO)  8.2% treatment of polystyrene, open dump, very wet infiltration class (1000mm) (GLO)) | Refer to items 39 &45 |
| 52 | Not specified Plastics Other End-of-Life | 0.00063 |  | kg | 31% Not specified Plastic Collected by Formal Waste Collectors  8% Not specified Plastic Collected by Formal Waste Collectors  61% Uncollected Waste (59% treatment of waste plastic, mixture, open burning (GLO)  32.8% treatment of waste plastic, mixture, open dump, very wet infiltration class (300mm) (GLO)  8.2% treatment of plastic, mixture, open dump, very wet infiltration class (1000mm) (GLO)) | Refer to items 40 &46 |
| 53 | Avoided Polystyrene Production | -0.0089 |  | kg | rket for polystyrene,general purpose (RoW) |  |

Table S5: Krebet Senggrong Current Scenario

| No. | Description | Input | Output | Unit | Activity(ies) Name in Ecoinvent v3.10 Database | Comment |
| --- | --- | --- | --- | --- | --- | --- |
| 1 | Recycling of Plastic | 0 |  | kg | -0.01 kg of plastic flake, consumer electronics, unsorted  0.1915kWh market for electricity, low voltage (ID)  0.00778 kg market for liquefied petroleum gas (RoW)  1.01 kg of plastic flake, consumer for recycling (GLO) | From Neo et al 2021 |
| 2 | Incineration of Polyethylene | 0 |  | kg | treatment of waste polyethylene, municipal incineration (GLO) |  |
| 3 | Incineration of Polypropylene | 0 |  | kg | treatment of waste polypropylene, municipal incineration (GLO) |  |
| 4 | Incineration of Polyethylene Terephthalate | 0 |  | kg | treatment of waste polyethylene terephthalate, municipal incineration (GLO) |  |
| 5 | Incineration of Polyvinylchloride | 0 |  | kg | treatment of waste polyvinylchloride, municipal incineration (GLO) |  |
| 6 | Incineration of Polystyrene | 0 |  | kg | treatment of waste polystyrene, municipal incineration (GLO) |  |
| 7 | Incineration of Not specified Plastic | 0 |  | kg | treatment of waste plastic, mixture, municipal incineration (GLO) |  |
| 8 | Landfilling of Polyethylene | 0 |  | kg | treatment of waste polyethylene, sanitary landfill (RoW) |  |
| 9 | Landfilling of Polypropylene | 0 |  | kg | treatment of waste polypropylene, sanitary landfill (RoW) |  |
| 10 | Landfilling of Polyethylene Terephthalate | 0 |  | kg | treatment of waste polyethylene terephthalate, sanitary landfill (RoW) |  |
| 11 | Landfilling of Polyvinylchloride | 0 |  | kg | treatment of waste polyvinylchloride, sanitary landfill (RoW) |  |
| 12 | Landfilling of Polystyrene | 0 |  | kg | treatment of waste polystyrene, sanitary landfill (RoW) |  |
| 13 | Landfilling of Not specified Plastics | 0 |  | kg | treatment of waste plastic, mixture, sanitary landfill (RoW) |  |
| 14 | Open dumping (on land) of Polyethylene | -0.103 |  | kg | treatment of waste polyethylene, open dump, moist infiltration class (300mm) (GLO) |  |
| 15 | Open dumping (on land) of Polypropylene | -0.034 |  | kg | treatment of waste polypropylene, open dump, moist infiltration class (300mm) (GLO) |  |
| 16 | Open dumping (on land) of Polyethylene Terephthalate | -0.032 |  | kg | treatment of waste polyethylene terephthalate, open dump, moist infiltration class (300mm) (GLO) |  |
| 17 | Open dumping (on land) of Polyvinylchloride | -0.0037 |  | kg | treatment of waste polyvinylchloride, open dump, moist infiltration class (300mm) (GLO) |  |
| 18 | Open dumping (on land) of Polystyrene | -0.021 |  | kg | treatment of waste polystyrene, open dump, moist infiltration class (300mm) (GLO) |  |
| 19 | Open dumping (on land) of Not specified Plastics | -0.19 |  | kg | treatment of waste plastic, mixture, open dump, moist infiltration class (300mm) (GLO) |  |
| 20 | Open burning of Polyethylene | -0.14 |  | kg | treatment of waste polyethylene, open burning (GLO) |  |
| 21 | Open burning of Polypropylene | -0.04 |  | kg | treatment of waste polypropylene, open burning (GLO) |  |
| 22 | Open burning of Polyethylene Terephthalate | -0.037 |  | kg | treatment of waste polyethylene terephthalate, open burning (GLO) |  |
| 23 | Open burning of Polyvinylchloride | -0.0055 |  | kg | treatment of waste polyvinylchloride, open burning (GLO) |  |
| 24 | Open burning of Polystyrene | -0.0226 |  | kg | treatment of waste polystyrene, open burning (GLO) |  |
| 25 | Open burning of Not specified Plastics | -0.251 |  | kg | treatment of waste plastic, mixture, open burning (GLO) |  |
| 26 | Open dump (in water) of Polyethylene | 0 |  | kg | treatment of waste polyethylene, open dump, very wet infiltration class (1000mm) (GLO) |  |
| 30 | Open dump (in water) of Polypropylene | 0 |  | kg | treatment of waste polypropylene, open dump, very wet infiltration class (1000mm) (GLO) |  |
| 31 | Open dump (in water) of Polyethylene Terephthalate | 0 |  | kg | treatment of waste polyethylene terephthalate, open dump, very wet infiltration class (1000mm) (GLO) |  |
| 32 | Open dump (in water) of Polyvinylchloride | 0 |  | kg | treatment of waste polyvinylchloride, open dump, very wet infiltration class (1000mm) (GLO) |  |
| 33 | Open dump (in water) of Polystyrene | 0 |  | kg | treatment of waste polystyrene, open dump, very wet infiltration class (1000mm) (GLO) |  |
| 34 | Open dump (in water) of Not specified Plastics | 0 |  | kg | treatment of waste plastic, mixture, open dump, very wet infiltration class (1000mm) (GLO) |  |
| 35 | Polyethylene Collected by Formal Waste Collectors | 0.097 |  | kg | 79% treatment of waste polyethylene, sanitary landfill (RoW)  16% treatment of waste polyethylene, open dump, very wet infiltration class (300mm) (GLO)  4% treatment of waste polyethylene, open dump, very wet infiltration class (1000mm) (GLO) |  |
| 36 | Polypropylene Collected by Formal Waste Collectors | 0.037 |  | kg | 79% treatment of waste polypropylene, sanitary landfill (RoW)  16% treatment of waste polypropylene, open dump, very wet infiltration class (300mm) (GLO)  4% treatment of waste polypropylene, open dump, very wet infiltration class (1000mm) (GLO) |  |
| 37 | Polyethylene Terephthalate Collected by Formal Waste Collectors | 0.035 |  | kg | 79% treatment of waste polyethylene terephthalate, sanitary landfill (RoW)  16% treatment of waste polyethylene terephthalate, open dump, very wet infiltration class (300mm) (GLO)  4% treatment of waste polyethylene terephthalate, open dump, very wet infiltration class (1000mm) (GLO) |  |
| 38 | Polyvinylchloride Collected by Formal Waste Collectors | 0.004 |  | kg | 79% treatment of waste polyvinylchloride, sanitary landfill (RoW)  16% treatment of waste polyvinylchloride, open dump, very wet infiltration class (300mm) (GLO)  4% treatment of waste polyvinylchloride, open dump, very wet infiltration class (1000mm) (GLO) |  |
| 39 | Polystyrene Collected by Formal Waste Collectors | 0.021 |  | kg | 79% treatment of waste polystyrene, sanitary landfill (RoW)  16% treatment of waste polystyrene, open dump, very wet infiltration class (300mm) (GLO)  4% treatment of waste polystyrene, open dump, very wet infiltration class (1000mm) (GLO) |  |
| 40 | Not specified Plastics Collected by Formal Waste Collectors | 0.195 |  | kg | 79% treatment of waste plastic, mixture, sanitary landfill (RoW)  16% treatment of waste plastic, mixture, open dump, very wet infiltration class (300mm) (GLO)  4% treatment of waste plastic, mixture, open dump, very wet infiltration class (1000mm) (GLO) |  |
| 41 | Polyethylene Collected by Informal Waste Collectors | 0.039 |  | kg | 70% plastic, recycled  24% treatment of waste polyethylene, sanitary landfill (RoW)  4.8% treatment of waste polyethylene, open dump, very wet infiltration class (300mm) (GLO)  1.2% treatment of waste polyethylene, open dump, very wet infiltration class (1000mm) (GLO) | Refer to Item 1 |
| 42 | Polypropylene Collected by Informal Waste Collectors | 0.015 |  | kg | 70% plastic, recycled  24% treatment of waste polypropylene, sanitary landfill (RoW)  4.8% treatment of waste polypropylene, open dump, very wet infiltration class (300mm) (GLO)  1.2% treatment of waste polypropylene, open dump, very wet infiltration class (1000mm) (GLO) | Refer to Item 1 |
| 43 | Polyethylene Terephthalate Collected by Informal Waste Collectors | 0.017 |  | kg | 70% plastic, recycled  24% treatment of waste polyethylene terephthalate, sanitary landfill (RoW)  4.8% treatment of waste polyethylene terephthalate, open dump, very wet infiltration class (300mm) (GLO)  1.2% treatment of waste polyethylene, open dump, very wet infiltration class (1000mm) (GLO) | Refer to Item 1 |
| 44 | Polyvinylchloride Collected by Informal Waste Collectors | 0.0014 |  | kg | 70% plastic, recycled  24% treatment of waste polyvinylchloride, sanitary landfill (RoW)  4.8% treatment of waste polyvinylchloride, open dump, very wet infiltration class (300mm) (GLO)  1.2% treatment of waste polyvinylchloride, open dump, very wet infiltration class (1000mm) (GLO) | Refer to Item 1 |
| 45 | Polystyrene Collected by Informal Waste Collectors | 0.0073 |  | kg | 70% plastic, recycled  24% treatment of waste polystyrene, sanitary landfill (RoW)  4.8% treatment of waste polystyrene, open dump, very wet infiltration class (300mm) (GLO)  1.2% treatment of waste polystyrene, open dump, very wet infiltration class (1000mm) (GLO) | Refer to Item 1 |
| 46 | Not specified Plastics Collected by Informal Waste Collectors | 0.079 |  | kg | 70% plastic, recycled  24% treatment of waste plastic, mixture, sanitary landfill (RoW)  4.8% treatment of waste plastic, mixture, open dump, very wet infiltration class (300mm) (GLO)  1.2% treatment of waste plastic mixture, open dump, very wet infiltration class (1000mm) (GLO) | Refer to Item 1 |
| 47 | Polyethylene Other End-of-Life | 0.0116 |  | kg | 31% Polyethylene Collected by Formal Waste Collectors  8% Polyethylene Collected by Formal Waste Collectors  61% Uncollected Waste (59% treatment of waste polyethylene, open burning (GLO)  32.8% treatment of waste polyethylene, open dump, very wet infiltration class (300mm) (GLO)  8.2% treatment of polyethylene, open dump, very wet infiltration class (1000mm) (GLO)) | Refer to items 35 &41 |
| 48 | Polypropylene Other End-of-Life | 0.0335 |  | kg | 31% Polypropylene Collected by Formal Waste Collectors  8% Polypropylene Collected by Formal Waste Collectors  61% Uncollected Waste (59% treatment of waste polypropylene, open burning (GLO)  32.8% treatment of waste polypropylene, open dump, very wet infiltration class (300mm) (GLO)  8.2% treatment of polypropylene, open dump, very wet infiltration class (1000mm) (GLO)) | Refer to items 36 &42 |
| 49 | Polyethylene Terephthalate Other End-of-Life | 0.191 |  | kg | 31% Polyethylene Terephthalate Collected by Formal Waste Collectors  8% Polyethylene Terephthalate Collected by Formal Waste Collectors  61% Uncollected Waste (59% treatment of waste polyethylene terephthalate, open burning (GLO)  32.8% treatment of waste polyethylene terephthalate, open dump, very wet infiltration class (300mm) (GLO)  8.2% treatment of polyethylene terephthalate, open dump, very wet infiltration class (1000mm) (GLO)) | Refer to items 37 &43 |
| 50 | Polyvinylchloride Other End-of-Life | 0.0331 |  | kg | 31% Polyvinylchloride Collected by Formal Waste Collectors  8% Polyvinylchloride Collected by Formal Waste Collectors  61% Uncollected Waste (59% treatment of waste polyvinylchloride, open burning (GLO)  32.8% treatment of waste polyvinylchloride, open dump, very wet infiltration class (300mm) (GLO)  8.2% treatment of polyvinylchloride, open dump, very wet infiltration class (1000mm) (GLO)) | Refer to items 38 &44 |
| 51 | Polystyrene Other End-of-Life | 0.0069 |  | kg | 31% Polystyrene Collected by Formal Waste Collectors  8% Polystyrene Collected by Formal Waste Collectors  61% Uncollected Waste (59% treatment of waste polystyrene, open burning (GLO)  32.8% treatment of waste polystyrene, open dump, very wet infiltration class (300mm) (GLO)  8.2% treatment of polystyrene, open dump, very wet infiltration class (1000mm) (GLO)) | Refer to items 39 &45 |
| 52 | Not specified Plastics Other End-of-Life | 0.286 |  | kg | 31% Not specified Plastic Collected by Formal Waste Collectors  8% Not specified Plastic Collected by Formal Waste Collectors  61% Uncollected Waste (59% treatment of waste plastic, mixture, open burning (GLO)  32.8% treatment of waste plastic, mixture, open dump, very wet infiltration class (300mm) (GLO)  8.2% treatment of plastic, mixture, open dump, very wet infiltration class (1000mm) (GLO)) | Refer to items 40 &46 |
| 53 | Transport of plastic waste with scooter | 0.0071 |  | Person kilometer | Transport, passenger, motor scooter |  |

Table S6: Krebet Current Scenario

| No. | Description | Input | Output | Unit | Activity(ies) Name in Ecoinvent v3.10 Database | Comment |
| --- | --- | --- | --- | --- | --- | --- |
| 1 | Recycling of Plastic | 0.118 |  | kg | -0.01 kg of plastic flake, consumer electronics, unsorted  0.1915kWh market for electricity, low voltage (ID)  0.00778 kg market for liquefied petroleum gas (RoW)  1.01 kg of plastic flake, consumer for recycling (GLO) | From Neo et al 2021 |
| 2 | Incineration of Polyethylene | 0 |  | kg | treatment of waste polyethylene, municipal incineration (GLO) |  |
| 3 | Incineration of Polypropylene | 0 |  | kg | treatment of waste polypropylene, municipal incineration (GLO) |  |
| 4 | Incineration of Polyethylene Terephthalate | 0 |  | kg | treatment of waste polyethylene terephthalate, municipal incineration (GLO) |  |
| 5 | Incineration of Polyvinylchloride | 0 |  | kg | treatment of waste polyvinylchloride, municipal incineration (GLO) |  |
| 6 | Incineration of Polystyrene | 0 |  | kg | treatment of waste polystyrene, municipal incineration (GLO) |  |
| 7 | Incineration of Not specified Plastic | 0 |  | kg | treatment of waste plastic, mixture, municipal incineration (GLO) |  |
| 8 | Landfilling of Polyethylene | 0 |  | kg | treatment of waste polyethylene, sanitary landfill (RoW) |  |
| 9 | Landfilling of Polypropylene | 0 |  | kg | treatment of waste polypropylene, sanitary landfill (RoW) |  |
| 10 | Landfilling of Polyethylene Terephthalate | 0 |  | kg | treatment of waste polyethylene terephthalate, sanitary landfill (RoW) |  |
| 11 | Landfilling of Polyvinylchloride | 0 |  | kg | treatment of waste polyvinylchloride, sanitary landfill (RoW) |  |
| 12 | Landfilling of Polystyrene | 0 |  | kg | treatment of waste polystyrene, sanitary landfill (RoW) |  |
| 13 | Landfilling of Not specified Plastics | 0 |  | kg | treatment of waste plastic, mixture, sanitary landfill (RoW) |  |
| 14 | Open dumping (on land) of Polyethylene | -0.0024 |  | kg | treatment of waste polyethylene, open dump, moist infiltration class (300mm) (GLO) |  |
| 15 | Open dumping (on land) of Polypropylene | -0.00058 |  | kg | treatment of waste polypropylene, open dump, moist infiltration class (300mm) (GLO) |  |
| 16 | Open dumping (on land) of Polyethylene Terephthalate | -0.0012 |  | kg | treatment of waste polyethylene terephthalate, open dump, moist infiltration class (300mm) (GLO) |  |
| 17 | Open dumping (on land) of Polyvinylchloride | -0.00016 |  | kg | treatment of waste polyvinylchloride, open dump, moist infiltration class (300mm) (GLO) |  |
| 18 | Open dumping (on land) of Polystyrene | -0.00022 |  | kg | treatment of waste polystyrene, open dump, moist infiltration class (300mm) (GLO) |  |
| 19 | Open dumping (on land) of Not specified Plastics | 0 |  | kg | treatment of waste plastic, mixture, open dump, moist infiltration class (300mm) (GLO) |  |
| 20 | Open burning of Polyethylene | -0.02211 |  | kg | treatment of waste polyethylene, open burning (GLO) |  |
| 21 | Open burning of Polypropylene | -0.0058 |  | kg | treatment of waste polypropylene, open burning (GLO) |  |
| 22 | Open burning of Polyethylene Terephthalate | -0.0121 |  | kg | treatment of waste polyethylene terephthalate, open burning (GLO) |  |
| 23 | Open burning of Polyvinylchloride | -0.00136 |  | kg | treatment of waste polyvinylchloride, open burning (GLO) |  |
| 24 | Open burning of Polystyrene | -0.00243 |  | kg | treatment of waste polystyrene, open burning (GLO) |  |
| 25 | Open burning of Not specified Plastics | 0 |  | kg | treatment of waste plastic, mixture, open burning (GLO) |  |
| 26 | Open dump (in water) of Polyethylene | 0 |  | kg | treatment of waste polyethylene, open dump, very wet infiltration class (1000mm) (GLO) |  |
| 30 | Open dump (in water) of Polypropylene | 0 |  | kg | treatment of waste polypropylene, open dump, very wet infiltration class (1000mm) (GLO) |  |
| 31 | Open dump (in water) of Polyethylene Terephthalate | 0 |  | kg | treatment of waste polyethylene terephthalate, open dump, very wet infiltration class (1000mm) (GLO) |  |
| 32 | Open dump (in water) of Polyvinylchloride | 0 |  | kg | treatment of waste polyvinylchloride, open dump, very wet infiltration class (1000mm) (GLO) |  |
| 33 | Open dump (in water) of Polystyrene | 0 |  | kg | treatment of waste polystyrene, open dump, very wet infiltration class (1000mm) (GLO) |  |
| 34 | Open dump (in water) of Not specified Plastics | 0 |  | kg | treatment of waste plastic, mixture, open dump, very wet infiltration class (1000mm) (GLO) |  |
| 35 | Polyethylene Collected by Formal Waste Collectors | 0.0064 |  | kg | 79% treatment of waste polyethylene, sanitary landfill (RoW)  16% treatment of waste polyethylene, open dump, very wet infiltration class (300mm) (GLO)  4% treatment of waste polyethylene, open dump, very wet infiltration class (1000mm) (GLO) |  |
| 36 | Polypropylene Collected by Formal Waste Collectors | 0.00082 |  | kg | 79% treatment of waste polypropylene, sanitary landfill (RoW)  16% treatment of waste polypropylene, open dump, very wet infiltration class (300mm) (GLO)  4% treatment of waste polypropylene, open dump, very wet infiltration class (1000mm) (GLO) |  |
| 37 | Polyethylene Terephthalate Collected by Formal Waste Collectors | 0.0019 |  | kg | 79% treatment of waste polyethylene terephthalate, sanitary landfill (RoW)  16% treatment of waste polyethylene terephthalate, open dump, very wet infiltration class (300mm) (GLO)  4% treatment of waste polyethylene terephthalate, open dump, very wet infiltration class (1000mm) (GLO) |  |
| 38 | Polyvinylchloride Collected by Formal Waste Collectors | 0.00018 |  | kg | 79% treatment of waste polyvinylchloride, sanitary landfill (RoW)  16% treatment of waste polyvinylchloride, open dump, very wet infiltration class (300mm) (GLO)  4% treatment of waste polyvinylchloride, open dump, very wet infiltration class (1000mm) (GLO) |  |
| 39 | Polystyrene Collected by Formal Waste Collectors | 0.00077 |  | kg | 79% treatment of waste polystyrene, sanitary landfill (RoW)  16% treatment of waste polystyrene, open dump, very wet infiltration class (300mm) (GLO)  4% treatment of waste polystyrene, open dump, very wet infiltration class (1000mm) (GLO) |  |
| 40 | Not specified Plastics Collected by Formal Waste Collectors | 0 |  | kg | 79% treatment of waste plastic, mixture, sanitary landfill (RoW)  16% treatment of waste plastic, mixture, open dump, very wet infiltration class (300mm) (GLO)  4% treatment of waste plastic, mixture, open dump, very wet infiltration class (1000mm) (GLO) |  |
| 41 | Polyethylene Collected by Informal Waste Collectors | 0.0096 |  | kg | 70% plastic, recycled  24% treatment of waste polyethylene, sanitary landfill (RoW)  4.8% treatment of waste polyethylene, open dump, very wet infiltration class (300mm) (GLO)  1.2% treatment of waste polyethylene, open dump, very wet infiltration class (1000mm) (GLO) | Refer to Item 1 |
| 42 | Polypropylene Collected by Informal Waste Collectors | 0.0032 |  | kg | 70% plastic, recycled  24% treatment of waste polypropylene, sanitary landfill (RoW)  4.8% treatment of waste polypropylene, open dump, very wet infiltration class (300mm) (GLO)  1.2% treatment of waste polypropylene, open dump, very wet infiltration class (1000mm) (GLO) | Refer to Item 1 |
| 43 | Polyethylene Terephthalate Collected by Informal Waste Collectors | 0.00422 |  | kg | 70% plastic, recycled  24% treatment of waste polyethylene terephthalate, sanitary landfill (RoW)  4.8% treatment of waste polyethylene terephthalate, open dump, very wet infiltration class (300mm) (GLO)  1.2% treatment of waste polyethylene, open dump, very wet infiltration class (1000mm) (GLO) | Refer to Item 1 |
| 44 | Polyvinylchloride Collected by Informal Waste Collectors | 0.00031 |  | kg | 70% plastic, recycled  24% treatment of waste polyvinylchloride, sanitary landfill (RoW)  4.8% treatment of waste polyvinylchloride, open dump, very wet infiltration class (300mm) (GLO)  1.2% treatment of waste polyvinylchloride, open dump, very wet infiltration class (1000mm) (GLO) | Refer to Item 1 |
| 45 | Polystyrene Collected by Informal Waste Collectors | 0.00167 |  | kg | 70% plastic, recycled  24% treatment of waste polystyrene, sanitary landfill (RoW)  4.8% treatment of waste polystyrene, open dump, very wet infiltration class (300mm) (GLO)  1.2% treatment of waste polystyrene, open dump, very wet infiltration class (1000mm) (GLO) | Refer to Item 1 |
| 46 | Not specified Plastics Collected by Informal Waste Collectors | 0 |  | kg | 70% plastic, recycled  24% treatment of waste plastic, mixture, sanitary landfill (RoW)  4.8% treatment of waste plastic, mixture, open dump, very wet infiltration class (300mm) (GLO)  1.2% treatment of waste plastic mixture, open dump, very wet infiltration class (1000mm) (GLO) | Refer to Item 1 |
| 47 | Polyethylene Other End-of-Life | 0.44 |  | kg | 31% Polyethylene Collected by Formal Waste Collectors  8% Polyethylene Collected by Formal Waste Collectors  61% Uncollected Waste (59% treatment of waste polyethylene, open burning (GLO)  32.8% treatment of waste polyethylene, open dump, very wet infiltration class (300mm) (GLO)  8.2% treatment of polyethylene, open dump, very wet infiltration class (1000mm) (GLO)) | Refer to items 35 &41 |
| 48 | Polypropylene Other End-of-Life | 0.097 |  | kg | 31% Polypropylene Collected by Formal Waste Collectors  8% Polypropylene Collected by Formal Waste Collectors  61% Uncollected Waste (59% treatment of waste polypropylene, open burning (GLO)  32.8% treatment of waste polypropylene, open dump, very wet infiltration class (300mm) (GLO)  8.2% treatment of polypropylene, open dump, very wet infiltration class (1000mm) (GLO)) | Refer to items 36 &42 |
| 49 | Polyethylene Terephthalate Other End-of-Life | 0.154 |  | kg | 31% Polyethylene Terephthalate Collected by Formal Waste Collectors  8% Polyethylene Terephthalate Collected by Formal Waste Collectors  61% Uncollected Waste (59% treatment of waste polyethylene terephthalate, open burning (GLO)  32.8% treatment of waste polyethylene terephthalate, open dump, very wet infiltration class (300mm) (GLO)  8.2% treatment of polyethylene terephthalate, open dump, very wet infiltration class (1000mm) (GLO)) | Refer to items 37 &43 |
| 50 | Polyvinylchloride Other End-of-Life | 0.0243 |  | kg | 31% Polyvinylchloride Collected by Formal Waste Collectors  8% Polyvinylchloride Collected by Formal Waste Collectors  61% Uncollected Waste (59% treatment of waste polyvinylchloride, open burning (GLO)  32.8% treatment of waste polyvinylchloride, open dump, very wet infiltration class (300mm) (GLO)  8.2% treatment of polyvinylchloride, open dump, very wet infiltration class (1000mm) (GLO)) | Refer to items 38 &44 |
| 51 | Polystyrene Other End-of-Life | 0.08866 |  | kg | 31% Polystyrene Collected by Formal Waste Collectors  8% Polystyrene Collected by Formal Waste Collectors  61% Uncollected Waste (59% treatment of waste polystyrene, open burning (GLO)  32.8% treatment of waste polystyrene, open dump, very wet infiltration class (300mm) (GLO)  8.2% treatment of polystyrene, open dump, very wet infiltration class (1000mm) (GLO)) | Refer to items 39 &45 |
| 52 | Not specified Plastics Other End-of-Life | 0 |  | kg | 31% Not specified Plastic Collected by Formal Waste Collectors  8% Not specified Plastic Collected by Formal Waste Collectors  61% Uncollected Waste (59% treatment of waste plastic, mixture, open burning (GLO)  32.8% treatment of waste plastic, mixture, open dump, very wet infiltration class (300mm) (GLO)  8.2% treatment of plastic, mixture, open dump, very wet infiltration class (1000mm) (GLO)) | Refer to items 40 &46 |
| 53 | Transport of plastic waste with car | 0.005 |  | kg | Transport, passenger car, small size, diesel, EURO4 |  |
| 54 | Avoided high density polyethylene production | -0.0241 |  | kg | Market for polyethylene, high density, granulate |  |
| 55 | Avoided low density polyethylene production | -0.0363 |  | kg | Market for polyethylene, low density, granulate |  |
| 56 | Avoided polypropylene production | -0.0212 |  | kg | Market for polypropylene, granulate |  |
| 57 | Avoided polyethylene terephthalate production | -0.0212 |  | kg | Market for polyethylene terephthalate, granulate amorphous |  |
| 58 | Avoided polyvinylchloride production | -0.009 |  | kg | Market for polyvinylchloride, bulk polymerised |  |
| 59 | Avoided polystyrene production | -0.006 |  | kg | Market for polystyrene, general purpose |  |

Table S7: Mangunrejo Current Scenario

| No. | Description | Input | Output | Unit | Activity(ies) Name in Ecoinvent v3.10 Database | Comment |
| --- | --- | --- | --- | --- | --- | --- |
| 1 | Recycling of Plastic | 0.143 |  | kg | -0.01 kg of plastic flake, consumer electronics, unsorted  0.1915kWh market for electricity, low voltage (ID)  0.00778 kg market for liquefied petroleum gas (RoW)  1.01 kg of plastic flake, consumer for recycling (GLO) | From Neo et al 2021 |
| 2 | Incineration of Polyethylene | 0 |  | kg | treatment of waste polyethylene, municipal incineration (GLO) |  |
| 3 | Incineration of Polypropylene | 0 |  | kg | treatment of waste polypropylene, municipal incineration (GLO) |  |
| 4 | Incineration of Polyethylene Terephthalate | 0 |  | kg | treatment of waste polyethylene terephthalate, municipal incineration (GLO) |  |
| 5 | Incineration of Polyvinylchloride | 0 |  | kg | treatment of waste polyvinylchloride, municipal incineration (GLO) |  |
| 6 | Incineration of Polystyrene | 0 |  | kg | treatment of waste polystyrene, municipal incineration (GLO) |  |
| 7 | Incineration of Not specified Plastic | 0 |  | kg | treatment of waste plastic, mixture, municipal incineration (GLO) |  |
| 8 | Landfilling of Polyethylene | 0 |  | kg | treatment of waste polyethylene, sanitary landfill (RoW) |  |
| 9 | Landfilling of Polypropylene | 0 |  | kg | treatment of waste polypropylene, sanitary landfill (RoW) |  |
| 10 | Landfilling of Polyethylene Terephthalate | 0 |  | kg | treatment of waste polyethylene terephthalate, sanitary landfill (RoW) |  |
| 11 | Landfilling of Polyvinylchloride | 0 |  | kg | treatment of waste polyvinylchloride, sanitary landfill (RoW) |  |
| 12 | Landfilling of Polystyrene | 0 |  | kg | treatment of waste polystyrene, sanitary landfill (RoW) |  |
| 13 | Landfilling of Not specified Plastics | 0 |  | kg | treatment of waste plastic, mixture, sanitary landfill (RoW) |  |
| 14 | Open dumping (on land) of Polyethylene | 0 |  | kg | treatment of waste polyethylene, open dump, moist infiltration class (300mm) (GLO) |  |
| 15 | Open dumping (on land) of Polypropylene | 0 |  | kg | treatment of waste polypropylene, open dump, moist infiltration class (300mm) (GLO) |  |
| 16 | Open dumping (on land) of Polyethylene Terephthalate | 0 |  | kg | treatment of waste polyethylene terephthalate, open dump, moist infiltration class (300mm) (GLO) |  |
| 17 | Open dumping (on land) of Polyvinylchloride | 0 |  | kg | treatment of waste polyvinylchloride, open dump, moist infiltration class (300mm) (GLO) |  |
| 18 | Open dumping (on land) of Polystyrene | 0 |  | kg | treatment of waste polystyrene, open dump, moist infiltration class (300mm) (GLO) |  |
| 19 | Open dumping (on land) of Not specified Plastics | 0 |  | kg | treatment of waste plastic, mixture, open dump, moist infiltration class (300mm) (GLO) |  |
| 20 | Open burning of Polyethylene | 0 |  | kg | treatment of waste polyethylene, open burning (GLO) |  |
| 21 | Open burning of Polypropylene | 0 |  | kg | treatment of waste polypropylene, open burning (GLO) |  |
| 22 | Open burning of Polyethylene Terephthalate | 0 |  | kg | treatment of waste polyethylene terephthalate, open burning (GLO) |  |
| 23 | Open burning of Polyvinylchloride | 0 |  | kg | treatment of waste polyvinylchloride, open burning (GLO) |  |
| 24 | Open burning of Polystyrene | 0 |  | kg | treatment of waste polystyrene, open burning (GLO) |  |
| 25 | Open burning of Not specified Plastics | 0 |  | kg | treatment of waste plastic, mixture, open burning (GLO) |  |
| 26 | Open dump (in water) of Polyethylene | 0 |  | kg | treatment of waste polyethylene, open dump, very wet infiltration class (1000mm) (GLO) |  |
| 30 | Open dump (in water) of Polypropylene | 0 |  | kg | treatment of waste polypropylene, open dump, very wet infiltration class (1000mm) (GLO) |  |
| 31 | Open dump (in water) of Polyethylene Terephthalate | 0 |  | kg | treatment of waste polyethylene terephthalate, open dump, very wet infiltration class (1000mm) (GLO) |  |
| 32 | Open dump (in water) of Polyvinylchloride | 0 |  | kg | treatment of waste polyvinylchloride, open dump, very wet infiltration class (1000mm) (GLO) |  |
| 33 | Open dump (in water) of Polystyrene | 0 |  | kg | treatment of waste polystyrene, open dump, very wet infiltration class (1000mm) (GLO) |  |
| 34 | Open dump (in water) of Not specified Plastics | 0 |  | kg | treatment of waste plastic, mixture, open dump, very wet infiltration class (1000 mm) (GLO) |  |
| 35 | Polyethylene Collected by Formal Waste Collectors | 0 |  | kg | 79% treatment of waste polyethylene, sanitary landfill (RoW)  16% treatment of waste polyethylene, open dump, very wet infiltration class (300mm) (GLO)  4% treatment of waste polyethylene, open dump, very wet infiltration class (1000mm) (GLO) |  |
| 36 | Polypropylene Collected by Formal Waste Collectors | 0.857 |  | kg | 79% treatment of waste polypropylene, sanitary landfill (RoW)  16% treatment of waste polypropylene, open dump, very wet infiltration class (300mm) (GLO)  4% treatment of waste polypropylene, open dump, very wet infiltration class (1000mm) (GLO) |  |
| 37 | Polyethylene Terephthalate Collected by Formal Waste Collectors | 0 |  | kg | 79% treatment of waste polyethylene terephthalate, sanitary landfill (RoW)  16% treatment of waste polyethylene terephthalate, open dump, very wet infiltration class (300mm) (GLO)  4% treatment of waste polyethylene terephthalate, open dump, very wet infiltration class (1000mm) (GLO) |  |
| 38 | Polyvinylchloride Collected by Formal Waste Collectors | 0 |  | kg | 79% treatment of waste polyvinylchloride, sanitary landfill (RoW)  16% treatment of waste polyvinylchloride, open dump, very wet infiltration class (300mm) (GLO)  4% treatment of waste polyvinylchloride, open dump, very wet infiltration class (1000mm) (GLO) |  |
| 39 | Polystyrene Collected by Formal Waste Collectors | 0 |  | kg | 79% treatment of waste polystyrene, sanitary landfill (RoW)  16% treatment of waste polystyrene, open dump, very wet infiltration class (300mm) (GLO)  4% treatment of waste polystyrene, open dump, very wet infiltration class (1000mm) (GLO) |  |
| 40 | Not specified Plastics Collected by Formal Waste Collectors | 0 |  | kg | 79% treatment of waste plastic, mixture, sanitary landfill (RoW)  16% treatment of waste plastic, mixture, open dump, very wet infiltration class (300mm) (GLO)  4% treatment of waste plastic, mixture, open dump, very wet infiltration class (1000mm) (GLO) |  |
| 41 | Polyethylene Collected by Informal Waste Collectors | 0 |  | kg | 70% plastic, recycled  24% treatment of waste polyethylene, sanitary landfill (RoW)  4.8% treatment of waste polyethylene, open dump, very wet infiltration class (300mm) (GLO)  1.2% treatment of waste polyethylene, open dump, very wet infiltration class (1000mm) (GLO) | Refer to Item 1 |
| 42 | Polypropylene Collected by Informal Waste Collectors | 0 |  | kg | 70% plastic, recycled  24% treatment of waste polypropylene, sanitary landfill (RoW)  4.8% treatment of waste polypropylene, open dump, very wet infiltration class (300mm) (GLO)  1.2% treatment of waste polypropylene, open dump, very wet infiltration class (1000mm) (GLO) | Refer to Item 1 |
| 43 | Polyethylene Terephthalate Collected by Informal Waste Collectors | 0 |  | kg | 70% plastic, recycled  24% treatment of waste polyethylene terephthalate, sanitary landfill (RoW)  4.8% treatment of waste polyethylene terephthalate, open dump, very wet infiltration class (300mm) (GLO)  1.2% treatment of waste polyethylene, open dump, very wet infiltration class (1000mm) (GLO) | Refer to Item 1 |
| 44 | Polyvinylchloride Collected by Informal Waste Collectors | 0 |  | kg | 70% plastic, recycled  24% treatment of waste polyvinylchloride, sanitary landfill (RoW)  4.8% treatment of waste polyvinylchloride, open dump, very wet infiltration class (300mm) (GLO)  1.2% treatment of waste polyvinylchloride, open dump, very wet infiltration class (1000mm) (GLO) | Refer to Item 1 |
| 45 | Polystyrene Collected by Informal Waste Collectors | 0 |  | kg | 70% plastic, recycled  24% treatment of waste polystyrene, sanitary landfill (RoW)  4.8% treatment of waste polystyrene, open dump, very wet infiltration class (300mm) (GLO)  1.2% treatment of waste polystyrene, open dump, very wet infiltration class (1000mm) (GLO) | Refer to Item 1 |
| 46 | Not specified Plastics Collected by Informal Waste Collectors | 0 |  | kg | 70% plastic, recycled  24% treatment of waste plastic, mixture, sanitary landfill (RoW)  4.8% treatment of waste plastic, mixture, open dump, very wet infiltration class (300mm) (GLO)  1.2% treatment of waste plastic mixture, open dump, very wet infiltration class (1000mm) (GLO) | Refer to Item 1 |
| 47 | Polyethylene Other End-of-Life | 0 |  | kg | 31% Polyethylene Collected by Formal Waste Collectors  8% Polyethylene Collected by Formal Waste Collectors  61% Uncollected Waste (59% treatment of waste polyethylene, open burning (GLO)  32.8% treatment of waste polyethylene, open dump, very wet infiltration class (300mm) (GLO)  8.2% treatment of polyethylene, open dump, very wet infiltration class (1000mm) (GLO)) | Refer to items 35 &41 |
| 48 | Polypropylene Other End-of-Life | 0 |  | kg | 31% Polypropylene Collected by Formal Waste Collectors  8% Polypropylene Collected by Formal Waste Collectors  61% Uncollected Waste (59% treatment of waste polypropylene, open burning (GLO)  32.8% treatment of waste polypropylene, open dump, very wet infiltration class (300mm) (GLO)  8.2% treatment of polypropylene, open dump, very wet infiltration class (1000mm) (GLO)) | Refer to items 36 &42 |
| 49 | Polyethylene Terephthalate Other End-of-Life | 0 |  | kg | 31% Polyethylene Terephthalate Collected by Formal Waste Collectors  8% Polyethylene Terephthalate Collected by Formal Waste Collectors  61% Uncollected Waste (59% treatment of waste polyethylene terephthalate, open burning (GLO)  32.8% treatment of waste polyethylene terephthalate, open dump, very wet infiltration class (300mm) (GLO)  8.2% treatment of polyethylene terephthalate, open dump, very wet infiltration class (1000mm) (GLO)) | Refer to items 37 &43 |
| 50 | Polyvinylchloride Other End-of-Life | 0 |  | kg | 31% Polyvinylchloride Collected by Formal Waste Collectors  8% Polyvinylchloride Collected by Formal Waste Collectors  61% Uncollected Waste (59% treatment of waste polyvinylchloride, open burning (GLO)  32.8% treatment of waste polyvinylchloride, open dump, very wet infiltration class (300mm) (GLO)  8.2% treatment of polyvinylchloride, open dump, very wet infiltration class (1000mm) (GLO)) | Refer to items 38 &44 |
| 51 | Polystyrene Other End-of-Life | 0 |  | kg | 31% Polystyrene Collected by Formal Waste Collectors  8% Polystyrene Collected by Formal Waste Collectors  61% Uncollected Waste (59% treatment of waste polystyrene, open burning (GLO)  32.8% treatment of waste polystyrene, open dump, very wet infiltration class (300mm) (GLO)  8.2% treatment of polystyrene, open dump, very wet infiltration class (1000mm) (GLO)) | Refer to items 39 &45 |
| 52 | Not specified Plastics Other End-of-Life | 0 |  | kg | 31% Not specified Plastic Collected by Formal Waste Collectors  8% Not specified Plastic Collected by Formal Waste Collectors  61% Uncollected Waste (59% treatment of waste plastic, mixture, open burning (GLO)  32.8% treatment of waste plastic, mixture, open dump, very wet infiltration class (300mm) (GLO)  8.2% treatment of plastic, mixture, open dump, very wet infiltration class (1000mm) (GLO)) | Refer to items 40 &46 |
| 53 | Avoided Polystyrene Production | -0.1427 |  | kg | Market for polystyrene |  |

Table S8: Rekasan Current Scenario

| No. | Description | Input | Output | Unit | Activity(ies) Name in Ecoinvent v3.10 Database | Comment |
| --- | --- | --- | --- | --- | --- | --- |
| 1 | Recycling of Plastic | 0 |  | kg | -0.01 kg of plastic flake, consumer electronics, unsorted  0.1915kWh market for electricity, low voltage (ID)  0.00778 kg market for liquefied petroleum gas (RoW)  1.01 kg of plastic flake, consumer for recycling (GLO) | From Neo et al 2021 |
| 2 | Incineration of Polyethylene | 0 |  | kg | treatment of waste polyethylene, municipal incineration (GLO) |  |
| 3 | Incineration of Polypropylene | 0 |  | kg | treatment of waste polypropylene, municipal incineration (GLO) |  |
| 4 | Incineration of Polyethylene Terephthalate | 0 |  | kg | treatment of waste polyethylene terephthalate, municipal incineration (GLO) |  |
| 5 | Incineration of Polyvinylchloride | 0 |  | kg | treatment of waste polyvinylchloride, municipal incineration (GLO) |  |
| 6 | Incineration of Polystyrene | 0 |  | kg | treatment of waste polystyrene, municipal incineration (GLO) |  |
| 7 | Incineration of Not specified Plastic | 0 |  | kg | treatment of waste plastic, mixture, municipal incineration (GLO) |  |
| 8 | Landfilling of Polyethylene | -1 |  | kg | treatment of waste polyethylene, sanitary landfill (RoW) |  |
| 9 | Landfilling of Polypropylene | 0 |  | kg | treatment of waste polypropylene, sanitary landfill (RoW) |  |
| 10 | Landfilling of Polyethylene Terephthalate | 0 |  | kg | treatment of waste polyethylene terephthalate, sanitary landfill (RoW) |  |
| 11 | Landfilling of Polyvinylchloride | 0 |  | kg | treatment of waste polyvinylchloride, sanitary landfill (RoW) |  |
| 12 | Landfilling of Polystyrene | 0 |  | kg | treatment of waste polystyrene, sanitary landfill (RoW) |  |
| 13 | Landfilling of Not specified Plastics | 0 |  | kg | treatment of waste plastic, mixture, sanitary landfill (RoW) |  |
| 14 | Open dumping (on land) of Polyethylene | 0 |  | kg | treatment of waste polyethylene, open dump, moist infiltration class (300mm) (GLO) |  |
| 15 | Open dumping (on land) of Polypropylene | 0 |  | kg | treatment of waste polypropylene, open dump, moist infiltration class (300mm) (GLO) |  |
| 16 | Open dumping (on land) of Polyethylene Terephthalate | 0 |  | kg | treatment of waste polyethylene terephthalate, open dump, moist infiltration class (300mm) (GLO) |  |
| 17 | Open dumping (on land) of Polyvinylchloride | 0 |  | kg | treatment of waste polyvinylchloride, open dump, moist infiltration class (300mm) (GLO) |  |
| 18 | Open dumping (on land) of Polystyrene | 0 |  | kg | treatment of waste polystyrene, open dump, moist infiltration class (300mm) (GLO) |  |
| 19 | Open dumping (on land) of Not specified Plastics | 0 |  | kg | treatment of waste plastic, mixture, open dump, moist infiltration class (300mm) (GLO) |  |
| 20 | Open burning of Polyethylene | 0 |  | kg | treatment of waste polyethylene, open burning (GLO) |  |
| 21 | Open burning of Polypropylene | 0 |  | kg | treatment of waste polypropylene, open burning (GLO) |  |
| 22 | Open burning of Polyethylene Terephthalate | 0 |  | kg | treatment of waste polyethylene terephthalate, open burning (GLO) |  |
| 23 | Open burning of Polyvinylchloride | 0 |  | kg | treatment of waste polyvinylchloride, open burning (GLO) |  |
| 24 | Open burning of Polystyrene | 0 |  | kg | treatment of waste polystyrene, open burning (GLO) |  |
| 25 | Open burning of Not specified Plastics | 0 |  | kg | treatment of waste plastic, mixture, open burning (GLO) |  |
| 26 | Open dump (in water) of Polyethylene | 0 |  | kg | treatment of waste polyethylene, open dump, very wet infiltration class (1000mm) (GLO) |  |
| 30 | Open dump (in water) of Polypropylene | 0 |  | kg | treatment of waste polypropylene, open dump, very wet infiltration class (1000mm) (GLO) |  |
| 31 | Open dump (in water) of Polyethylene Terephthalate | 0 |  | kg | treatment of waste polyethylene terephthalate, open dump, very wet infiltration class (1000mm) (GLO) |  |
| 32 | Open dump (in water) of Polyvinylchloride | 0 |  | kg | treatment of waste polyvinylchloride, open dump, very wet infiltration class (1000mm) (GLO) |  |
| 33 | Open dump (in water) of Polystyrene | 0 |  | kg | treatment of waste polystyrene, open dump, very wet infiltration class (1000mm) (GLO) |  |
| 34 | Open dump (in water) of Not specified Plastics | 0 |  | kg | treatment of waste plastic, mixture, open dump, very wet infiltration class (1000mm) (GLO) |  |
| 35 | Polyethylene Collected by Formal Waste Collectors | 0 |  | kg | 79% treatment of waste polyethylene, sanitary landfill (RoW)  16% treatment of waste polyethylene, open dump, very wet infiltration class (300mm) (GLO)  4% treatment of waste polyethylene, open dump, very wet infiltration class (1000mm) (GLO) |  |
| 36 | Polypropylene Collected by Formal Waste Collectors | 0 |  | kg | 79% treatment of waste polypropylene, sanitary landfill (RoW)  16% treatment of waste polypropylene, open dump, very wet infiltration class (300mm) (GLO)  4% treatment of waste polypropylene, open dump, very wet infiltration class (1000mm) (GLO) |  |
| 37 | Polyethylene Terephthalate Collected by Formal Waste Collectors | 0 |  | kg | 79% treatment of waste polyethylene terephthalate, sanitary landfill (RoW)  16% treatment of waste polyethylene terephthalate, open dump, very wet infiltration class (300mm) (GLO)  4% treatment of waste polyethylene terephthalate, open dump, very wet infiltration class (1000mm) (GLO) |  |
| 38 | Polyvinylchloride Collected by Formal Waste Collectors | 0 |  | kg | 79% treatment of waste polyvinylchloride, sanitary landfill (RoW)  16% treatment of waste polyvinylchloride, open dump, very wet infiltration class (300mm) (GLO)  4% treatment of waste polyvinylchloride, open dump, very wet infiltration class (1000mm) (GLO) |  |
| 39 | Polystyrene Collected by Formal Waste Collectors | 0 |  | kg | 79% treatment of waste polystyrene, sanitary landfill (RoW)  16% treatment of waste polystyrene, open dump, very wet infiltration class (300mm) (GLO)  4% treatment of waste polystyrene, open dump, very wet infiltration class (1000mm) (GLO) |  |
| 40 | Not specified Plastics Collected by Formal Waste Collectors | 0 |  | kg | 79% treatment of waste plastic, mixture, sanitary landfill (RoW)  16% treatment of waste plastic, mixture, open dump, very wet infiltration class (300mm) (GLO)  4% treatment of waste plastic, mixture, open dump, very wet infiltration class (1000mm) (GLO) |  |
| 41 | Polyethylene Collected by Informal Waste Collectors | 0 |  | kg | 70% plastic, recycled  24% treatment of waste polyethylene, sanitary landfill (RoW)  4.8% treatment of waste polyethylene, open dump, very wet infiltration class (300mm) (GLO)  1.2% treatment of waste polyethylene, open dump, very wet infiltration class (1000mm) (GLO) | Refer to Item 1 |
| 42 | Polypropylene Collected by Informal Waste Collectors | 0 |  | kg | 70% plastic, recycled  24% treatment of waste polypropylene, sanitary landfill (RoW)  4.8% treatment of waste polypropylene, open dump, very wet infiltration class (300mm) (GLO)  1.2% treatment of waste polypropylene, open dump, very wet infiltration class (1000mm) (GLO) | Refer to Item 1 |
| 43 | Polyethylene Terephthalate Collected by Informal Waste Collectors | 0 |  | kg | 70% plastic, recycled  24% treatment of waste polyethylene terephthalate, sanitary landfill (RoW)  4.8% treatment of waste polyethylene terephthalate, open dump, very wet infiltration class (300mm) (GLO)  1.2% treatment of waste polyethylene, open dump, very wet infiltration class (1000mm) (GLO) | Refer to Item 1 |
| 44 | Polyvinylchloride Collected by Informal Waste Collectors | 0 |  | kg | 70% plastic, recycled  24% treatment of waste polyvinylchloride, sanitary landfill (RoW)  4.8% treatment of waste polyvinylchloride, open dump, very wet infiltration class (300mm) (GLO)  1.2% treatment of waste polyvinylchloride, open dump, very wet infiltration class (1000mm) (GLO) | Refer to Item 1 |
| 45 | Polystyrene Collected by Informal Waste Collectors | 0 |  | kg | 70% plastic, recycled  24% treatment of waste polystyrene, sanitary landfill (RoW)  4.8% treatment of waste polystyrene, open dump, very wet infiltration class (300mm) (GLO)  1.2% treatment of waste polystyrene, open dump, very wet infiltration class (1000mm) (GLO) | Refer to Item 1 |
| 46 | Not specified Plastics Collected by Informal Waste Collectors | 0 |  | kg | 70% plastic, recycled  24% treatment of waste plastic, mixture, sanitary landfill (RoW)  4.8% treatment of waste plastic, mixture, open dump, very wet infiltration class (300mm) (GLO)  1.2% treatment of waste plastic mixture, open dump, very wet infiltration class (1000mm) (GLO) | Refer to Item 1 |
| 47 | Polyethylene Other End-of-Life | 0 |  | kg | 31% Polyethylene Collected by Formal Waste Collectors  8% Polyethylene Collected by Formal Waste Collectors  61% Uncollected Waste (59% treatment of waste polyethylene, open burning (GLO)  32.8% treatment of waste polyethylene, open dump, very wet infiltration class (300mm) (GLO)  8.2% treatment of polyethylene, open dump, very wet infiltration class (1000mm) (GLO)) | Refer to items 35 &41 |
| 48 | Polypropylene Other End-of-Life | 0 |  | kg | 31% Polypropylene Collected by Formal Waste Collectors  8% Polypropylene Collected by Formal Waste Collectors  61% Uncollected Waste (59% treatment of waste polypropylene, open burning (GLO)  32.8% treatment of waste polypropylene, open dump, very wet infiltration class (300mm) (GLO)  8.2% treatment of polypropylene, open dump, very wet infiltration class (1000mm) (GLO)) | Refer to items 36 &42 |
| 49 | Polyethylene Terephthalate Other End-of-Life | 0 |  | kg | 31% Polyethylene Terephthalate Collected by Formal Waste Collectors  8% Polyethylene Terephthalate Collected by Formal Waste Collectors  61% Uncollected Waste (59% treatment of waste polyethylene terephthalate, open burning (GLO)  32.8% treatment of waste polyethylene terephthalate, open dump, very wet infiltration class (300mm) (GLO)  8.2% treatment of polyethylene terephthalate, open dump, very wet infiltration class (1000mm) (GLO)) | Refer to items 37 &43 |
| 50 | Polyvinylchloride Other End-of-Life | 0 |  | kg | 31% Polyvinylchloride Collected by Formal Waste Collectors  8% Polyvinylchloride Collected by Formal Waste Collectors  61% Uncollected Waste (59% treatment of waste polyvinylchloride, open burning (GLO)  32.8% treatment of waste polyvinylchloride, open dump, very wet infiltration class (300mm) (GLO)  8.2% treatment of polyvinylchloride, open dump, very wet infiltration class (1000mm) (GLO)) | Refer to items 38 &44 |
| 51 | Polystyrene Other End-of-Life | 0 |  | kg | 31% Polystyrene Collected by Formal Waste Collectors  8% Polystyrene Collected by Formal Waste Collectors  61% Uncollected Waste (59% treatment of waste polystyrene, open burning (GLO)  32.8% treatment of waste polystyrene, open dump, very wet infiltration class (300mm) (GLO)  8.2% treatment of polystyrene, open dump, very wet infiltration class (1000mm) (GLO)) | Refer to items 39 &45 |
| 52 | Not specified Plastics Other End-of-Life | 0 |  | kg | 31% Not specified Plastic Collected by Formal Waste Collectors  8% Not specified Plastic Collected by Formal Waste Collectors  61% Uncollected Waste (59% treatment of waste plastic, mixture, open burning (GLO)  32.8% treatment of waste plastic, mixture, open dump, very wet infiltration class (300mm) (GLO)  8.2% treatment of plastic, mixture, open dump, very wet infiltration class (1000mm) (GLO)) | Refer to items 40 &46 |

Table S9: Sumberejo Current Scenario

| No. | Description | Input | Output | Unit | Activity(ies) Name in Ecoinvent v3.10 Database | Comment |
| --- | --- | --- | --- | --- | --- | --- |
| 1 | Recycling of Plastic | 0.0055 |  | kg | -0.01 kg of plastic flake, consumer electronics, unsorted  0.1915kWh market for electricity, low voltage (ID)  0.00778 kg market for liquefied petroleum gas (RoW)  1.01 kg of plastic flake, consumer for recycling (GLO) | From Neo et al 2021 |
| 2 | Incineration of Polyethylene | 0 |  | kg | treatment of waste polyethylene, municipal incineration (GLO) |  |
| 3 | Incineration of Polypropylene | 0 |  | kg | treatment of waste polypropylene, municipal incineration (GLO) |  |
| 4 | Incineration of Polyethylene Terephthalate | 0 |  | kg | treatment of waste polyethylene terephthalate, municipal incineration (GLO) |  |
| 5 | Incineration of Polyvinylchloride | 0 |  | kg | treatment of waste polyvinylchloride, municipal incineration (GLO) |  |
| 6 | Incineration of Polystyrene | 0 |  | kg | treatment of waste polystyrene, municipal incineration (GLO) |  |
| 7 | Incineration of Not specified Plastic | 0 |  | kg | treatment of waste plastic, mixture, municipal incineration (GLO) |  |
| 8 | Landfilling of Polyethylene | -0.0028 |  | kg | treatment of waste polyethylene, sanitary landfill (RoW) |  |
| 9 | Landfilling of Polypropylene | -0.0014 |  | kg | treatment of waste polypropylene, sanitary landfill (RoW) |  |
| 10 | Landfilling of Polyethylene Terephthalate | -0.000123 |  | kg | treatment of waste polyethylene terephthalate, sanitary landfill (RoW) |  |
| 11 | Landfilling of Polyvinylchloride | -0.0011 |  | kg | treatment of waste polyvinylchloride, sanitary landfill (RoW) |  |
| 12 | Landfilling of Polystyrene | -0.002615 |  | kg | treatment of waste polystyrene, sanitary landfill (RoW) |  |
| 13 | Landfilling of Not specified Plastics | -0.00016 |  | kg | treatment of waste plastic, mixture, sanitary landfill (RoW) |  |
| 14 | Open dumping (on land) of Polyethylene | -0.0021 |  | kg | treatment of waste polyethylene, open dump, moist infiltration class (300mm) (GLO) |  |
| 15 | Open dumping (on land) of Polypropylene | -0.00102 |  | kg | treatment of waste polypropylene, open dump, moist infiltration class (300mm) (GLO) |  |
| 16 | Open dumping (on land) of Polyethylene Terephthalate | -0.0011 |  | kg | treatment of waste polyethylene terephthalate, open dump, moist infiltration class (300mm) (GLO) |  |
| 17 | Open dumping (on land) of Polyvinylchloride | -0.00096 |  | kg | treatment of waste polyvinylchloride, open dump, moist infiltration class (300mm) (GLO) |  |
| 18 | Open dumping (on land) of Polystyrene | -0.002248 |  | kg | treatment of waste polystyrene, open dump, moist infiltration class (300mm) (GLO) |  |
| 19 | Open dumping (on land) of Not specified Plastics | -0.000148 |  | kg | treatment of waste plastic, mixture, open dump, moist infiltration class (300mm) (GLO) |  |
| 20 | Open burning of Polyethylene | -0.019 |  | kg | treatment of waste polyethylene, open burning (GLO) |  |
| 21 | Open burning of Polypropylene | -0.00559 |  | kg | treatment of waste polypropylene, open burning (GLO) |  |
| 22 | Open burning of Polyethylene Terephthalate | -0.00918 |  | kg | treatment of waste polyethylene terephthalate, open burning (GLO) |  |
| 23 | Open burning of Polyvinylchloride | -0.0082 |  | kg | treatment of waste polyvinylchloride, open burning (GLO) |  |
| 24 | Open burning of Polystyrene | -0.01877 |  | kg | treatment of waste polystyrene, open burning (GLO) |  |
| 25 | Open burning of Not specified Plastics | - 0.001264 |  | kg | treatment of waste plastic, mixture, open burning (GLO) |  |
| 26 | Open dump (in water) of Polyethylene | 0 |  | kg | treatment of waste polyethylene, open dump, very wet infiltration class (1000mm) (GLO) |  |
| 30 | Open dump (in water) of Polypropylene | 0 |  | kg | treatment of waste polypropylene, open dump, very wet infiltration class (1000mm) (GLO) |  |
| 31 | Open dump (in water) of Polyethylene Terephthalate | 0 |  | kg | treatment of waste polyethylene terephthalate, open dump, very wet infiltration class (1000mm) (GLO) |  |
| 32 | Open dump (in water) of Polyvinylchloride | 0 |  | kg | treatment of waste polyvinylchloride, open dump, very wet infiltration class (1000mm) (GLO) |  |
| 33 | Open dump (in water) of Polystyrene | 0 |  | kg | treatment of waste polystyrene, open dump, very wet infiltration class (1000mm) (GLO) |  |
| 34 | Open dump (in water) of Not specified Plastics | 0 |  | kg | treatment of waste plastic, mixture, open dump, very wet infiltration class (1000mm) (GLO) |  |
| 35 | Polyethylene Collected by Formal Waste Collectors | 0.00224 |  | kg | 79% treatment of waste polyethylene, sanitary landfill (RoW)  16% treatment of waste polyethylene, open dump, very wet infiltration class (300mm) (GLO)  4% treatment of waste polyethylene, open dump, very wet infiltration class (1000mm) (GLO) |  |
| 36 | Polypropylene Collected by Formal Waste Collectors | 0.00072 |  | kg | 79% treatment of waste polypropylene, sanitary landfill (RoW)  16% treatment of waste polypropylene, open dump, very wet infiltration class (300mm) (GLO)  4% treatment of waste polypropylene, open dump, very wet infiltration class (1000mm) (GLO) |  |
| 37 | Polyethylene Terephthalate Collected by Formal Waste Collectors | 0.0011 |  | kg | 79% treatment of waste polyethylene terephthalate, sanitary landfill (RoW)  16% treatment of waste polyethylene terephthalate, open dump, very wet infiltration class (300mm) (GLO)  4% treatment of waste polyethylene terephthalate, open dump, very wet infiltration class (1000mm) (GLO) |  |
| 38 | Polyvinylchloride Collected by Formal Waste Collectors | 0.0011 |  | kg | 79% treatment of waste polyvinylchloride, sanitary landfill (RoW)  16% treatment of waste polyvinylchloride, open dump, very wet infiltration class (300mm) (GLO)  4% treatment of waste polyvinylchloride, open dump, very wet infiltration class (1000mm) (GLO) |  |
| 39 | Polystyrene Collected by Formal Waste Collectors | 0.00252 |  | kg | 79% treatment of waste polystyrene, sanitary landfill (RoW)  16% treatment of waste polystyrene, open dump, very wet infiltration class (300mm) (GLO)  4% treatment of waste polystyrene, open dump, very wet infiltration class (1000mm) (GLO) |  |
| 40 | Not specified Plastics Collected by Formal Waste Collectors | 0.00017 |  | kg | 79% treatment of waste plastic, mixture, sanitary landfill (RoW)  16% treatment of waste plastic, mixture, open dump, very wet infiltration class (300mm) (GLO)  4% treatment of waste plastic, mixture, open dump, very wet infiltration class (1000mm) (GLO) |  |
| 41 | Polyethylene Collected by Informal Waste Collectors | 0.00377 |  | kg | 70% plastic, recycled  24% treatment of waste polyethylene, sanitary landfill (RoW)  4.8% treatment of waste polyethylene, open dump, very wet infiltration class (300mm) (GLO)  1.2% treatment of waste polyethylene, open dump, very wet infiltration class (1000mm) (GLO) | Refer to Item 1 |
| 42 | Polypropylene Collected by Informal Waste Collectors | 0.00122 |  | kg | 70% plastic, recycled  24% treatment of waste polypropylene, sanitary landfill (RoW)  4.8% treatment of waste polypropylene, open dump, very wet infiltration class (300mm) (GLO)  1.2% treatment of waste polypropylene, open dump, very wet infiltration class (1000mm) (GLO) | Refer to Item 1 |
| 43 | Polyethylene Terephthalate Collected by Informal Waste Collectors | 0.0018 |  | kg | 70% plastic, recycled  24% treatment of waste polyethylene terephthalate, sanitary landfill (RoW)  4.8% treatment of waste polyethylene terephthalate, open dump, very wet infiltration class (300mm) (GLO)  1.2% treatment of waste polyethylene, open dump, very wet infiltration class (1000mm) (GLO) | Refer to Item 1 |
| 44 | Polyvinylchloride Collected by Informal Waste Collectors | 0.00186 |  | kg | 70% plastic, recycled  24% treatment of waste polyvinylchloride, sanitary landfill (RoW)  4.8% treatment of waste polyvinylchloride, open dump, very wet infiltration class (300mm) (GLO)  1.2% treatment of waste polyvinylchloride, open dump, very wet infiltration class (1000mm) (GLO) | Refer to Item 1 |
| 45 | Polystyrene Collected by Informal Waste Collectors | 0.0042 |  | kg | 70% plastic, recycled  24% treatment of waste polystyrene, sanitary landfill (RoW)  4.8% treatment of waste polystyrene, open dump, very wet infiltration class (300mm) (GLO)  1.2% treatment of waste polystyrene, open dump, very wet infiltration class (1000mm) (GLO) | Refer to Item 1 |
| 46 | Not specified Plastics Collected by Informal Waste Collectors | 0.00029 |  | kg | 70% plastic, recycled  24% treatment of waste plastic, mixture, sanitary landfill (RoW)  4.8% treatment of waste plastic, mixture, open dump, very wet infiltration class (300mm) (GLO)  1.2% treatment of waste plastic mixture, open dump, very wet infiltration class (1000mm) (GLO) | Refer to Item 1 |
| 47 | Polyethylene Other End-of-Life | 0.4 |  | kg | 31% Polyethylene Collected by Formal Waste Collectors  8% Polyethylene Collected by Formal Waste Collectors  61% Uncollected Waste (59% treatment of waste polyethylene, open burning (GLO)  32.8% treatment of waste polyethylene, open dump, very wet infiltration class (300mm) (GLO)  8.2% treatment of polyethylene, open dump, very wet infiltration class (1000mm) (GLO)) | Refer to items 35 &41 |
| 48 | Polypropylene Other End-of-Life | 0.017 |  | kg | 31% Polypropylene Collected by Formal Waste Collectors  8% Polypropylene Collected by Formal Waste Collectors  61% Uncollected Waste (59% treatment of waste polypropylene, open burning (GLO)  32.8% treatment of waste polypropylene, open dump, very wet infiltration class (300mm) (GLO)  8.2% treatment of polypropylene, open dump, very wet infiltration class (1000mm) (GLO)) | Refer to items 36 &42 |
| 49 | Polyethylene Terephthalate Other End-of-Life | 0.43 |  | kg | 31% Polyethylene Terephthalate Collected by Formal Waste Collectors  8% Polyethylene Terephthalate Collected by Formal Waste Collectors  61% Uncollected Waste (59% treatment of waste polyethylene terephthalate, open burning (GLO)  32.8% treatment of waste polyethylene terephthalate, open dump, very wet infiltration class (300mm) (GLO)  8.2% treatment of polyethylene terephthalate, open dump, very wet infiltration class (1000mm) (GLO)) | Refer to items 37 &43 |
| 50 | Polyvinylchloride Other End-of-Life | 0 |  | kg | 31% Polyvinylchloride Collected by Formal Waste Collectors  8% Polyvinylchloride Collected by Formal Waste Collectors  61% Uncollected Waste (59% treatment of waste polyvinylchloride, open burning (GLO)  32.8% treatment of waste polyvinylchloride, open dump, very wet infiltration class (300mm) (GLO)  8.2% treatment of polyvinylchloride, open dump, very wet infiltration class (1000mm) (GLO)) | Refer to items 38 &44 |
| 51 | Polystyrene Other End-of-Life | 0 |  | kg | 31% Polystyrene Collected by Formal Waste Collectors  8% Polystyrene Collected by Formal Waste Collectors  61% Uncollected Waste (59% treatment of waste polystyrene, open burning (GLO)  32.8% treatment of waste polystyrene, open dump, very wet infiltration class (300mm) (GLO)  8.2% treatment of polystyrene, open dump, very wet infiltration class (1000mm) (GLO)) | Refer to items 39 &45 |
| 52 | Not specified Plastics Other End-of-Life | 0.048 |  | kg | 31% Not specified Plastic Collected by Formal Waste Collectors  8% Not specified Plastic Collected by Formal Waste Collectors  61% Uncollected Waste (59% treatment of waste plastic, mixture, open burning (GLO)  32.8% treatment of waste plastic, mixture, open dump, very wet infiltration class (300mm) (GLO)  8.2% treatment of plastic, mixture, open dump, very wet infiltration class (1000mm) (GLO)) | Refer to items 40 &46 |
| 53 | Avoided high density polyethylene production | -0.001464 |  | kg | Market for polyethylene, high density, granulate |  |
| 54 | Avoided low density polyethylene production | -0.000732 |  | kg | Market for polyethylene, low density, granulate |  |
| 55 | Avoided polyethylene terephthalate production | -0.003294 |  | kg | Market for polyethylene terephthalate, granulate, amorphous |  |

Table S10: Tegalsari Current Scenario

| No. | Description | Input | Output | Unit | Activity(ies) Name in Ecoinvent v3.10 Database | Comment |
| --- | --- | --- | --- | --- | --- | --- |
| 1 | Recycling of Plastic | 0.00454 |  | kg | -0.01 kg of plastic flake, consumer electronics, unsorted  0.1915kWh market for electricity, low voltage (ID)  0.00778 kg market for liquefied petroleum gas (RoW)  1.01 kg of plastic flake, consumer for recycling (GLO) | From Neo et al 2021 |
| 2 | Incineration of Polyethylene | 0 |  | kg | treatment of waste polyethylene, municipal incineration (GLO) |  |
| 3 | Incineration of Polypropylene | 0 |  | kg | treatment of waste polypropylene, municipal incineration (GLO) |  |
| 4 | Incineration of Polyethylene Terephthalate | 0 |  | kg | treatment of waste polyethylene terephthalate, municipal incineration (GLO) |  |
| 5 | Incineration of Polyvinylchloride | 0 |  | kg | treatment of waste polyvinylchloride, municipal incineration (GLO) |  |
| 6 | Incineration of Polystyrene | 0 |  | kg | treatment of waste polystyrene, municipal incineration (GLO) |  |
| 7 | Incineration of Not specified Plastic | 0 |  | kg | treatment of waste plastic, mixture, municipal incineration (GLO) |  |
| 8 | Landfilling of Polyethylene | 0 |  | kg | treatment of waste polyethylene, sanitary landfill (RoW) |  |
| 9 | Landfilling of Polypropylene | 0 |  | kg | treatment of waste polypropylene, sanitary landfill (RoW) |  |
| 10 | Landfilling of Polyethylene Terephthalate | 0 |  | kg | treatment of waste polyethylene terephthalate, sanitary landfill (RoW) |  |
| 11 | Landfilling of Polyvinylchloride | 0 |  | kg | treatment of waste polyvinylchloride, sanitary landfill (RoW) |  |
| 12 | Landfilling of Polystyrene | 0 |  | kg | treatment of waste polystyrene, sanitary landfill (RoW) |  |
| 13 | Landfilling of Not specified Plastics | 0 |  | kg | treatment of waste plastic, mixture, sanitary landfill (RoW) |  |
| 14 | Open dumping (on land) of Polyethylene | 0 |  | kg | treatment of waste polyethylene, open dump, moist infiltration class (300mm) (GLO) |  |
| 15 | Open dumping (on land) of Polypropylene | 0 |  | kg | treatment of waste polypropylene, open dump, moist infiltration class (300mm) (GLO) |  |
| 16 | Open dumping (on land) of Polyethylene Terephthalate | 0 |  | kg | treatment of waste polyethylene terephthalate, open dump, moist infiltration class (300mm) (GLO) |  |
| 17 | Open dumping (on land) of Polyvinylchloride | 0 |  | kg | treatment of waste polyvinylchloride, open dump, moist infiltration class (300mm) (GLO) |  |
| 18 | Open dumping (on land) of Polystyrene | 0 |  | kg | treatment of waste polystyrene, open dump, moist infiltration class (300mm) (GLO) |  |
| 19 | Open dumping (on land) of Not specified Plastics | 0 |  | kg | treatment of waste plastic, mixture, open dump, moist infiltration class (300mm) (GLO) |  |
| 20 | Open burning of Polyethylene | 0 |  | kg | treatment of waste polyethylene, open burning (GLO) |  |
| 21 | Open burning of Polypropylene | 0 |  | kg | treatment of waste polypropylene, open burning (GLO) |  |
| 22 | Open burning of Polyethylene Terephthalate | 0 |  | kg | treatment of waste polyethylene terephthalate, open burning (GLO) |  |
| 23 | Open burning of Polyvinylchloride | 0 |  | kg | treatment of waste polyvinylchloride, open burning (GLO) |  |
| 24 | Open burning of Polystyrene | 0 |  | kg | treatment of waste polystyrene, open burning (GLO) |  |
| 25 | Open burning of Not specified Plastics | -0.00454 |  | kg | treatment of waste plastic, mixture, open burning (GLO) |  |
| 26 | Open dump (in water) of Polyethylene | 0 |  | kg | treatment of waste polyethylene, open dump, very wet infiltration class (1000mm) (GLO) |  |
| 30 | Open dump (in water) of Polypropylene | 0 |  | kg | treatment of waste polypropylene, open dump, very wet infiltration class (1000mm) (GLO) |  |
| 31 | Open dump (in water) of Polyethylene Terephthalate | 0 |  | kg | treatment of waste polyethylene terephthalate, open dump, very wet infiltration class (1000mm) (GLO) |  |
| 32 | Open dump (in water) of Polyvinylchloride | 0 |  | kg | treatment of waste polyvinylchloride, open dump, very wet infiltration class (1000mm) (GLO) |  |
| 33 | Open dump (in water) of Polystyrene | 0 |  | kg | treatment of waste polystyrene, open dump, very wet infiltration class (1000mm) (GLO) |  |
| 34 | Open dump (in water) of Not specified Plastics | 0 |  | kg | treatment of waste plastic, mixture, open dump, very wet infiltration class (1000mm) (GLO) |  |
| 35 | Polyethylene Collected by Formal Waste Collectors | 0 |  | kg | 79% treatment of waste polyethylene, sanitary landfill (RoW)  16% treatment of waste polyethylene, open dump, very wet infiltration class (300mm) (GLO)  4% treatment of waste polyethylene, open dump, very wet infiltration class (1000mm) (GLO) |  |
| 36 | Polypropylene Collected by Formal Waste Collectors | 0 |  | kg | 79% treatment of waste polypropylene, sanitary landfill (RoW)  16% treatment of waste polypropylene, open dump, very wet infiltration class (300mm) (GLO)  4% treatment of waste polypropylene, open dump, very wet infiltration class (1000mm) (GLO) |  |
| 37 | Polyethylene Terephthalate Collected by Formal Waste Collectors | 0 |  | kg | 79% treatment of waste polyethylene terephthalate, sanitary landfill (RoW)  16% treatment of waste polyethylene terephthalate, open dump, very wet infiltration class (300mm) (GLO)  4% treatment of waste polyethylene terephthalate, open dump, very wet infiltration class (1000mm) (GLO) |  |
| 38 | Polyvinylchloride Collected by Formal Waste Collectors | 0 |  | kg | 79% treatment of waste polyvinylchloride, sanitary landfill (RoW)  16% treatment of waste polyvinylchloride, open dump, very wet infiltration class (300mm) (GLO)  4% treatment of waste polyvinylchloride, open dump, very wet infiltration class (1000mm) (GLO) |  |
| 39 | Polystyrene Collected by Formal Waste Collectors | 0 |  | kg | 79% treatment of waste polystyrene, sanitary landfill (RoW)  16% treatment of waste polystyrene, open dump, very wet infiltration class (300mm) (GLO)  4% treatment of waste polystyrene, open dump, very wet infiltration class (1000mm) (GLO) |  |
| 40 | Not specified Plastics Collected by Formal Waste Collectors | 0 |  | kg | 79% treatment of waste plastic, mixture, sanitary landfill (RoW)  16% treatment of waste plastic, mixture, open dump, very wet infiltration class (300mm) (GLO)  4% treatment of waste plastic, mixture, open dump, very wet infiltration class (1000mm) (GLO) |  |
| 41 | Polyethylene Collected by Informal Waste Collectors | 0 |  | kg | 70% plastic, recycled  24% treatment of waste polyethylene, sanitary landfill (RoW)  4.8% treatment of waste polyethylene, open dump, very wet infiltration class (300mm) (GLO)  1.2% treatment of waste polyethylene, open dump, very wet infiltration class (1000mm) (GLO) | Refer to Item 1 |
| 42 | Polypropylene Collected by Informal Waste Collectors | 0 |  | kg | 70% plastic, recycled  24% treatment of waste polypropylene, sanitary landfill (RoW)  4.8% treatment of waste polypropylene, open dump, very wet infiltration class (300mm) (GLO)  1.2% treatment of waste polypropylene, open dump, very wet infiltration class (1000mm) (GLO) | Refer to Item 1 |
| 43 | Polyethylene Terephthalate Collected by Informal Waste Collectors | 0 |  | kg | 70% plastic, recycled  24% treatment of waste polyethylene terephthalate, sanitary landfill (RoW)  4.8% treatment of waste polyethylene terephthalate, open dump, very wet infiltration class (300mm) (GLO)  1.2% treatment of waste polyethylene, open dump, very wet infiltration class (1000mm) (GLO) | Refer to Item 1 |
| 44 | Polyvinylchloride Collected by Informal Waste Collectors | 0 |  | kg | 70% plastic, recycled  24% treatment of waste polyvinylchloride, sanitary landfill (RoW)  4.8% treatment of waste polyvinylchloride, open dump, very wet infiltration class (300mm) (GLO)  1.2% treatment of waste polyvinylchloride, open dump, very wet infiltration class (1000mm) (GLO) | Refer to Item 1 |
| 45 | Polystyrene Collected by Informal Waste Collectors | 0 |  | kg | 70% plastic, recycled  24% treatment of waste polystyrene, sanitary landfill (RoW)  4.8% treatment of waste polystyrene, open dump, very wet infiltration class (300mm) (GLO)  1.2% treatment of waste polystyrene, open dump, very wet infiltration class (1000mm) (GLO) | Refer to Item 1 |
| 46 | Not specified Plastics Collected by Informal Waste Collectors | 0 |  | kg | 70% plastic, recycled  24% treatment of waste plastic, mixture, sanitary landfill (RoW)  4.8% treatment of waste plastic, mixture, open dump, very wet infiltration class (300mm) (GLO)  1.2% treatment of waste plastic mixture, open dump, very wet infiltration class (1000mm) (GLO) | Refer to Item 1 |
| 47 | Polyethylene Other End-of-Life | 0.26 |  | kg | 31% Polyethylene Collected by Formal Waste Collectors  8% Polyethylene Collected by Formal Waste Collectors  61% Uncollected Waste (59% treatment of waste polyethylene, open burning (GLO)  32.8% treatment of waste polyethylene, open dump, very wet infiltration class (300mm) (GLO)  8.2% treatment of polyethylene, open dump, very wet infiltration class (1000mm) (GLO)) | Refer to items 35 &41 |
| 48 | Polypropylene Other End-of-Life | 0.175 |  | kg | 31% Polypropylene Collected by Formal Waste Collectors  8% Polypropylene Collected by Formal Waste Collectors  61% Uncollected Waste (59% treatment of waste polypropylene, open burning (GLO)  32.8% treatment of waste polypropylene, open dump, very wet infiltration class (300mm) (GLO)  8.2% treatment of polypropylene, open dump, very wet infiltration class (1000mm) (GLO)) | Refer to items 36 &42 |
| 49 | Polyethylene Terephthalate Other End-of-Life | 0.432 |  | kg | 31% Polyethylene Terephthalate Collected by Formal Waste Collectors  8% Polyethylene Terephthalate Collected by Formal Waste Collectors  61% Uncollected Waste (59% treatment of waste polyethylene terephthalate, open burning (GLO)  32.8% treatment of waste polyethylene terephthalate, open dump, very wet infiltration class (300mm) (GLO)  8.2% treatment of polyethylene terephthalate, open dump, very wet infiltration class (1000mm) (GLO)) | Refer to items 37 &43 |
| 50 | Polyvinylchloride Other End-of-Life | 0 |  | kg | 31% Polyvinylchloride Collected by Formal Waste Collectors  8% Polyvinylchloride Collected by Formal Waste Collectors  61% Uncollected Waste (59% treatment of waste polyvinylchloride, open burning (GLO)  32.8% treatment of waste polyvinylchloride, open dump, very wet infiltration class (300mm) (GLO)  8.2% treatment of polyvinylchloride, open dump, very wet infiltration class (1000mm) (GLO)) | Refer to items 38 &44 |
| 51 | Polystyrene Other End-of-Life | 0 |  | kg | 31% Polystyrene Collected by Formal Waste Collectors  8% Polystyrene Collected by Formal Waste Collectors  61% Uncollected Waste (59% treatment of waste polystyrene, open burning (GLO)  32.8% treatment of waste polystyrene, open dump, very wet infiltration class (300mm) (GLO)  8.2% treatment of polystyrene, open dump, very wet infiltration class (1000mm) (GLO)) | Refer to items 39 &45 |
| 52 | Not specified Plastics Other End-of-Life | 0.0176 |  | kg | 31% Not specified Plastic Collected by Formal Waste Collectors  8% Not specified Plastic Collected by Formal Waste Collectors  61% Uncollected Waste (59% treatment of waste plastic, mixture, open burning (GLO)  32.8% treatment of waste plastic, mixture, open dump, very wet infiltration class (300mm) (GLO)  8.2% treatment of plastic, mixture, open dump, very wet infiltration class (1000mm) (GLO)) | Refer to items 40 &46 |
| 53 | Transport of plastic waste with lorry | 0.0083 |  | kg | Transport freight lorry 3.5-7.5 metric ton, EURO4 |  |
| 54 | Avoided high density polyethylene production | -0.00114 |  | kg | Market for polyethylene, high density, granulate |  |
| 55 | Avoided low density polyethylene production | -0.00227 |  | kg | Market for polyethylene, low density, granulate |  |

Table S11: Tlogorejo Current Scenario

| No. | Description | Input | Output | Unit | Activity(ies) Name in Ecoinvent v3.10 Database | Comment |
| --- | --- | --- | --- | --- | --- | --- |
| 1 | Recycling of Plastic | 0.084 |  | kg | -0.01 kg of plastic flake, consumer electronics, unsorted  0.1915kWh market for electricity, low voltage (ID)  0.00778 kg market for liquefied petroleum gas (RoW)  1.01 kg of plastic flake, consumer for recycling (GLO) | From Neo et al 2021 |
| 2 | Incineration of Polyethylene | 0 |  | kg | treatment of waste polyethylene, municipal incineration (GLO) |  |
| 3 | Incineration of Polypropylene | 0 |  | kg | treatment of waste polypropylene, municipal incineration (GLO) |  |
| 4 | Incineration of Polyethylene Terephthalate | 0 |  | kg | treatment of waste polyethylene terephthalate, municipal incineration (GLO) |  |
| 5 | Incineration of Polyvinylchloride | 0 |  | kg | treatment of waste polyvinylchloride, municipal incineration (GLO) |  |
| 6 | Incineration of Polystyrene | 0 |  | kg | treatment of waste polystyrene, municipal incineration (GLO) |  |
| 7 | Incineration of Not specified Plastic | 0 |  | kg | treatment of waste plastic, mixture, municipal incineration (GLO) |  |
| 8 | Landfilling of Polyethylene | -0.00061 |  | kg | treatment of waste polyethylene, sanitary landfill (RoW) |  |
| 9 | Landfilling of Polypropylene | -0.00589 |  | kg | treatment of waste polypropylene, sanitary landfill (RoW) |  |
| 10 | Landfilling of Polyethylene Terephthalate | -0.00126 |  | kg | treatment of waste polyethylene terephthalate, sanitary landfill (RoW) |  |
| 11 | Landfilling of Polyvinylchloride | -0.00232 |  | kg | treatment of waste polyvinylchloride, sanitary landfill (RoW) |  |
| 12 | Landfilling of Polystyrene | -0.0007 |  | kg | treatment of waste polystyrene, sanitary landfill (RoW) |  |
| 13 | Landfilling of Not specified Plastics | 0 |  | kg | treatment of waste plastic, mixture, sanitary landfill (RoW) |  |
| 14 | Open dumping (on land) of Polyethylene | -0.032 |  | kg | treatment of waste polyethylene, open dump, moist infiltration class (300mm) (GLO) |  |
| 15 | Open dumping (on land) of Polypropylene | -0.0057 |  | kg | treatment of waste polypropylene, open dump, moist infiltration class (300mm) (GLO) |  |
| 16 | Open dumping (on land) of Polyethylene Terephthalate | -0.0124 |  | kg | treatment of waste polyethylene terephthalate, open dump, moist infiltration class (300mm) (GLO) |  |
| 17 | Open dumping (on land) of Polyvinylchloride | -0.000332 |  | kg | treatment of waste polyvinylchloride, open dump, moist infiltration class (300mm) (GLO) |  |
| 18 | Open dumping (on land) of Polystyrene | -0.0031 |  | kg | treatment of waste polystyrene, open dump, moist infiltration class (300mm) (GLO) |  |
| 19 | Open dumping (on land) of Not specified Plastics | 0 |  | kg | treatment of waste plastic, mixture, open dump, moist infiltration class (300mm) (GLO) |  |
| 20 | Open burning of Polyethylene | -0.0116 |  | kg | treatment of waste polyethylene, open burning (GLO) |  |
| 21 | Open burning of Polypropylene | -0.00127 |  | kg | treatment of waste polypropylene, open burning (GLO) |  |
| 22 | Open burning of Polyethylene Terephthalate | -0.00534 |  | kg | treatment of waste polyethylene terephthalate, open burning (GLO) |  |
| 23 | Open burning of Polyvinylchloride | 0 |  | kg | treatment of waste polyvinylchloride, open burning (GLO) |  |
| 24 | Open burning of Polystyrene | -0.000528 |  | kg | treatment of waste polystyrene, open burning (GLO) |  |
| 25 | Open burning of Not specified Plastics | 0 |  | kg | treatment of waste plastic, mixture, open burning (GLO) |  |
| 26 | Open dump (in water) of Polyethylene | -0.0176 |  | kg | treatment of waste polyethylene, open dump, very wet infiltration class (1000mm) (GLO) |  |
| 30 | Open dump (in water) of Polypropylene | -0.0035 |  | kg | treatment of waste polypropylene, open dump, very wet infiltration class (1000mm) (GLO) |  |
| 31 | Open dump (in water) of Polyethylene Terephthalate | -0.00645 |  | kg | treatment of waste polyethylene terephthalate, open dump, very wet infiltration class (1000mm) (GLO) |  |
| 32 | Open dump (in water) of Polyvinylchloride | -0.000205 |  | kg | treatment of waste polyvinylchloride, open dump, very wet infiltration class (1000mm) (GLO) |  |
| 33 | Open dump (in water) of Polystyrene | -0.0019 |  | kg | treatment of waste polystyrene, open dump, very wet infiltration class (1000mm) (GLO) |  |
| 34 | Open dump (in water) of Not specified Plastics | 0 |  | kg | treatment of waste plastic, mixture, open dump, very wet infiltration class (1000mm) (GLO) |  |
| 35 | Polyethylene Collected by Formal Waste Collectors | 0 |  | kg | 79% treatment of waste polyethylene, sanitary landfill (RoW)  16% treatment of waste polyethylene, open dump, very wet infiltration class (300mm) (GLO)  4% treatment of waste polyethylene, open dump, very wet infiltration class (1000mm) (GLO) |  |
| 36 | Polypropylene Collected by Formal Waste Collectors | 0 |  | kg | 79% treatment of waste polypropylene, sanitary landfill (RoW)  16% treatment of waste polypropylene, open dump, very wet infiltration class (300mm) (GLO)  4% treatment of waste polypropylene, open dump, very wet infiltration class (1000mm) (GLO) |  |
| 37 | Polyethylene Terephthalate Collected by Formal Waste Collectors | 0 |  | kg | 79% treatment of waste polyethylene terephthalate, sanitary landfill (RoW)  16% treatment of waste polyethylene terephthalate, open dump, very wet infiltration class (300mm) (GLO)  4% treatment of waste polyethylene terephthalate, open dump, very wet infiltration class (1000mm) (GLO) |  |
| 38 | Polyvinylchloride Collected by Formal Waste Collectors | 0 |  | kg | 79% treatment of waste polyvinylchloride, sanitary landfill (RoW)  16% treatment of waste polyvinylchloride, open dump, very wet infiltration class (300mm) (GLO)  4% treatment of waste polyvinylchloride, open dump, very wet infiltration class (1000mm) (GLO) |  |
| 39 | Polystyrene Collected by Formal Waste Collectors | 0 |  | kg | 79% treatment of waste polystyrene, sanitary landfill (RoW)  16% treatment of waste polystyrene, open dump, very wet infiltration class (300mm) (GLO)  4% treatment of waste polystyrene, open dump, very wet infiltration class (1000mm) (GLO) |  |
| 40 | Not specified Plastics Collected by Formal Waste Collectors | 0 |  | kg | 79% treatment of waste plastic, mixture, sanitary landfill (RoW)  16% treatment of waste plastic, mixture, open dump, very wet infiltration class (300mm) (GLO)  4% treatment of waste plastic, mixture, open dump, very wet infiltration class (1000mm) (GLO) |  |
| 41 | Polyethylene Collected by Informal Waste Collectors | 0.0085 |  | kg | 70% plastic, recycled  24% treatment of waste polyethylene, sanitary landfill (RoW)  4.8% treatment of waste polyethylene, open dump, very wet infiltration class (300mm) (GLO)  1.2% treatment of waste polyethylene, open dump, very wet infiltration class (1000mm) (GLO) | Refer to Item 1 |
| 42 | Polypropylene Collected by Informal Waste Collectors | 0.00157 |  | kg | 70% plastic, recycled  24% treatment of waste polypropylene, sanitary landfill (RoW)  4.8% treatment of waste polypropylene, open dump, very wet infiltration class (300mm) (GLO)  1.2% treatment of waste polypropylene, open dump, very wet infiltration class (1000mm) (GLO) | Refer to Item 1 |
| 43 | Polyethylene Terephthalate Collected by Informal Waste Collectors | 0.00301 |  | kg | 70% plastic, recycled  24% treatment of waste polyethylene terephthalate, sanitary landfill (RoW)  4.8% treatment of waste polyethylene terephthalate, open dump, very wet infiltration class (300mm) (GLO)  1.2% treatment of waste polyethylene, open dump, very wet infiltration class (1000mm) (GLO) | Refer to Item 1 |
| 44 | Polyvinylchloride Collected by Informal Waste Collectors | 0 |  | kg | 70% plastic, recycled  24% treatment of waste polyvinylchloride, sanitary landfill (RoW)  4.8% treatment of waste polyvinylchloride, open dump, very wet infiltration class (300mm) (GLO)  1.2% treatment of waste polyvinylchloride, open dump, very wet infiltration class (1000mm) (GLO) | Refer to Item 1 |
| 45 | Polystyrene Collected by Informal Waste Collectors | 0.00069 |  | kg | 70% plastic, recycled  24% treatment of waste polystyrene, sanitary landfill (RoW)  4.8% treatment of waste polystyrene, open dump, very wet infiltration class (300mm) (GLO)  1.2% treatment of waste polystyrene, open dump, very wet infiltration class (1000mm) (GLO) | Refer to Item 1 |
| 46 | Not specified Plastics Collected by Informal Waste Collectors | 0 |  | kg | 70% plastic, recycled  24% treatment of waste plastic, mixture, sanitary landfill (RoW)  4.8% treatment of waste plastic, mixture, open dump, very wet infiltration class (300mm) (GLO)  1.2% treatment of waste plastic mixture, open dump, very wet infiltration class (1000mm) (GLO) | Refer to Item 1 |
| 47 | Polyethylene Other End-of-Life | 0.325 |  | kg | 31% Polyethylene Collected by Formal Waste Collectors  8% Polyethylene Collected by Formal Waste Collectors  61% Uncollected Waste (59% treatment of waste polyethylene, open burning (GLO)  32.8% treatment of waste polyethylene, open dump, very wet infiltration class (300mm) (GLO)  8.2% treatment of polyethylene, open dump, very wet infiltration class (1000mm) (GLO)) | Refer to items 35 &41 |
| 48 | Polypropylene Other End-of-Life | 0.04044 |  | kg | 31% Polypropylene Collected by Formal Waste Collectors  8% Polypropylene Collected by Formal Waste Collectors  61% Uncollected Waste (59% treatment of waste polypropylene, open burning (GLO)  32.8% treatment of waste polypropylene, open dump, very wet infiltration class (300mm) (GLO)  8.2% treatment of polypropylene, open dump, very wet infiltration class (1000mm) (GLO)) | Refer to items 36 &42 |
| 49 | Polyethylene Terephthalate Other End-of-Life | 0.231 |  | kg | 31% Polyethylene Terephthalate Collected by Formal Waste Collectors  8% Polyethylene Terephthalate Collected by Formal Waste Collectors  61% Uncollected Waste (59% treatment of waste polyethylene terephthalate, open burning (GLO)  32.8% treatment of waste polyethylene terephthalate, open dump, very wet infiltration class (300mm) (GLO)  8.2% treatment of polyethylene terephthalate, open dump, very wet infiltration class (1000mm) (GLO)) | Refer to items 37 &43 |
| 50 | Polyvinylchloride Other End-of-Life | 0 |  | kg | 31% Polyvinylchloride Collected by Formal Waste Collectors  8% Polyvinylchloride Collected by Formal Waste Collectors  61% Uncollected Waste (59% treatment of waste polyvinylchloride, open burning (GLO)  32.8% treatment of waste polyvinylchloride, open dump, very wet infiltration class (300mm) (GLO)  8.2% treatment of polyvinylchloride, open dump, very wet infiltration class (1000mm) (GLO)) | Refer to items 38 &44 |
| 51 | Polystyrene Other End-of-Life | 0.175 |  | kg | 31% Polystyrene Collected by Formal Waste Collectors  8% Polystyrene Collected by Formal Waste Collectors  61% Uncollected Waste (59% treatment of waste polystyrene, open burning (GLO)  32.8% treatment of waste polystyrene, open dump, very wet infiltration class (300mm) (GLO)  8.2% treatment of polystyrene, open dump, very wet infiltration class (1000mm) (GLO)) | Refer to items 39 &45 |
| 52 | Not specified Plastics Other End-of-Life | 0.00012 |  | kg | 31% Not specified Plastic Collected by Formal Waste Collectors  8% Not specified Plastic Collected by Formal Waste Collectors  61% Uncollected Waste (59% treatment of waste plastic, mixture, open burning (GLO)  32.8% treatment of waste plastic, mixture, open dump, very wet infiltration class (300mm) (GLO)  8.2% treatment of plastic, mixture, open dump, very wet infiltration class (1000mm) (GLO)) | Refer to items 40 &46 |
|  |  | 0.002 |  |  | Transport, passenger car, small size, petrol, EURO 4 |  |
| 53 | Transport of plastic waste with scooter | 0.001 |  | kg | Transport, passenger, motor scooter |  |
| 54 | Avoided production of high density polyethylene | -0.225 |  | kg | Market for polyethylene, high density, granulate |  |
| 55 | Avoided production of low density polyethylene | -0.0199 |  | kg | Market for polyethylene, low density, granulate |  |
| 56 | Avoided production of polypropylene | -0.0235 |  | kg | Market for polypropylene, granulate |  |

Table S12: Pagak Current Scenario

| No. | Description | Input | Output | Unit | Activity(ies) Name in Ecoinvent v3.10 Database | Comment |
| --- | --- | --- | --- | --- | --- | --- |
| 1 | Recycling of Plastic | 0 |  | kg | -0.01 kg of plastic flake, consumer electronics, unsorted  0.1915kWh market for electricity, low voltage (ID)  0.00778 kg market for liquefied petroleum gas (RoW)  1.01 kg of plastic flake, consumer for recycling (GLO) | From Neo et al 2021 |
| 2 | Incineration of Polyethylene | 0 |  | kg | treatment of waste polyethylene, municipal incineration (GLO) |  |
| 3 | Incineration of Polypropylene | 0 |  | kg | treatment of waste polypropylene, municipal incineration (GLO) |  |
| 4 | Incineration of Polyethylene Terephthalate | 0 |  | kg | treatment of waste polyethylene terephthalate, municipal incineration (GLO) |  |
| 5 | Incineration of Polyvinylchloride | 0 |  | kg | treatment of waste polyvinylchloride, municipal incineration (GLO) |  |
| 6 | Incineration of Polystyrene | 0 |  | kg | treatment of waste polystyrene, municipal incineration (GLO) |  |
| 7 | Incineration of Not specified Plastic | 0 |  | kg | treatment of waste plastic, mixture, municipal incineration (GLO) |  |
| 8 | Landfilling of Polyethylene | 0 |  | kg | treatment of waste polyethylene, sanitary landfill (RoW) |  |
| 9 | Landfilling of Polypropylene | 0 |  | kg | treatment of waste polypropylene, sanitary landfill (RoW) |  |
| 10 | Landfilling of Polyethylene Terephthalate | 0 |  | kg | treatment of waste polyethylene terephthalate, sanitary landfill (RoW) |  |
| 11 | Landfilling of Polyvinylchloride | 0 |  | kg | treatment of waste polyvinylchloride, sanitary landfill (RoW) |  |
| 12 | Landfilling of Polystyrene | 0 |  | kg | treatment of waste polystyrene, sanitary landfill (RoW) |  |
| 13 | Landfilling of Not specified Plastics | 0 |  | kg | treatment of waste plastic, mixture, sanitary landfill (RoW) |  |
| 14 | Open dumping (on land) of Polyethylene | -0.15 |  | kg | treatment of waste polyethylene, open dump, moist infiltration class (300mm) (GLO) |  |
| 15 | Open dumping (on land) of Polypropylene | -0.69 |  | kg | treatment of waste polypropylene, open dump, moist infiltration class (300mm) (GLO) |  |
| 16 | Open dumping (on land) of Polyethylene Terephthalate | -0.07 |  | kg | treatment of waste polyethylene terephthalate, open dump, moist infiltration class (300mm) (GLO) |  |
| 17 | Open dumping (on land) of Polyvinylchloride | -0.06 |  | kg | treatment of waste polyvinylchloride, open dump, moist infiltration class (300mm) (GLO) |  |
| 18 | Open dumping (on land) of Polystyrene | -0.002 |  | kg | treatment of waste polystyrene, open dump, moist infiltration class (300mm) (GLO) |  |
| 19 | Open dumping (on land) of Not specified Plastics | -0.06 |  | kg | treatment of waste plastic, mixture, open dump, moist infiltration class (300mm) (GLO) |  |
| 20 | Open burning of Polyethylene | -0.09 |  | kg | treatment of waste polyethylene, open burning (GLO) |  |
| 21 | Open burning of Polypropylene | -0.042 |  | kg | treatment of waste polypropylene, open burning (GLO) |  |
| 22 | Open burning of Polyethylene Terephthalate | -0.038 |  | kg | treatment of waste polyethylene terephthalate, open burning (GLO) |  |
| 23 | Open burning of Polyvinylchloride | -0.00097 |  | kg | treatment of waste polyvinylchloride, open burning (GLO) |  |
| 24 | Open burning of Polystyrene | -0.038 |  | kg | treatment of waste polystyrene, open burning (GLO) |  |
| 25 | Open burning of Not specified Plastics | 0 |  | kg | treatment of waste plastic, mixture, open burning (GLO) |  |
| 26 | Open dump (in water) of Polyethylene | -0.31 |  | kg | treatment of waste polyethylene, open dump, very wet infiltration class (1000mm) (GLO) |  |
| 30 | Open dump (in water) of Polypropylene | -0.14 |  | kg | treatment of waste polypropylene, open dump, very wet infiltration class (1000mm) (GLO) |  |
| 31 | Open dump (in water) of Polyethylene Terephthalate | -0.13 |  | kg | treatment of waste polyethylene terephthalate, open dump, very wet infiltration class (1000mm) (GLO) |  |
| 32 | Open dump (in water) of Polyvinylchloride | -0.003 |  | kg | treatment of waste polyvinylchloride, open dump, very wet infiltration class (1000mm) (GLO) |  |
| 33 | Open dump (in water) of Polystyrene | -0.13 |  | kg | treatment of waste polystyrene, open dump, very wet infiltration class (1000mm) (GLO) |  |
| 34 | Open dump (in water) of Not specified Plastics | 0 |  | kg | treatment of waste plastic, mixture, open dump, very wet infiltration class (1000mm) (GLO) |  |
| 35 | Polyethylene Collected by Formal Waste Collectors | 0.23 |  | kg | 79% treatment of waste polyethylene, sanitary landfill (RoW)  16% treatment of waste polyethylene, open dump, very wet infiltration class (300mm) (GLO)  4% treatment of waste polyethylene, open dump, very wet infiltration class (1000mm) (GLO) |  |
| 36 | Polypropylene Collected by Formal Waste Collectors | 0.11 |  | kg | 79% treatment of waste polypropylene, sanitary landfill (RoW)  16% treatment of waste polypropylene, open dump, very wet infiltration class (300mm) (GLO)  4% treatment of waste polypropylene, open dump, very wet infiltration class (1000mm) (GLO) |  |
| 37 | Polyethylene Terephthalate Collected by Formal Waste Collectors | 0.1 |  | kg | 79% treatment of waste polyethylene terephthalate, sanitary landfill (RoW)  16% treatment of waste polyethylene terephthalate, open dump, very wet infiltration class (300mm) (GLO)  4% treatment of waste polyethylene terephthalate, open dump, very wet infiltration class (1000mm) (GLO) |  |
| 38 | Polyvinylchloride Collected by Formal Waste Collectors | 0.0025 |  | kg | 79% treatment of waste polyvinylchloride, sanitary landfill (RoW)  16% treatment of waste polyvinylchloride, open dump, very wet infiltration class (300mm) (GLO)  4% treatment of waste polyvinylchloride, open dump, very wet infiltration class (1000mm) (GLO) |  |
| 39 | Polystyrene Collected by Formal Waste Collectors | 0.095 |  | kg | 79% treatment of waste polystyrene, sanitary landfill (RoW)  16% treatment of waste polystyrene, open dump, very wet infiltration class (300mm) (GLO)  4% treatment of waste polystyrene, open dump, very wet infiltration class (1000mm) (GLO) |  |
| 40 | Not specified Plastics Collected by Formal Waste Collectors | 0 |  | kg | 79% treatment of waste plastic, mixture, sanitary landfill (RoW)  16% treatment of waste plastic, mixture, open dump, very wet infiltration class (300mm) (GLO)  4% treatment of waste plastic, mixture, open dump, very wet infiltration class (1000mm) (GLO) |  |
| 41 | Polyethylene Collected by Informal Waste Collectors | 0 |  | kg | 70% plastic, recycled  24% treatment of waste polyethylene, sanitary landfill (RoW)  4.8% treatment of waste polyethylene, open dump, very wet infiltration class (300mm) (GLO)  1.2% treatment of waste polyethylene, open dump, very wet infiltration class (1000mm) (GLO) | Refer to Item 1 |
| 42 | Polypropylene Collected by Informal Waste Collectors | 0 |  | kg | 70% plastic, recycled  24% treatment of waste polypropylene, sanitary landfill (RoW)  4.8% treatment of waste polypropylene, open dump, very wet infiltration class (300mm) (GLO)  1.2% treatment of waste polypropylene, open dump, very wet infiltration class (1000mm) (GLO) | Refer to Item 1 |
| 43 | Polyethylene Terephthalate Collected by Informal Waste Collectors | 0 |  | kg | 70% plastic, recycled  24% treatment of waste polyethylene terephthalate, sanitary landfill (RoW)  4.8% treatment of waste polyethylene terephthalate, open dump, very wet infiltration class (300mm) (GLO)  1.2% treatment of waste polyethylene, open dump, very wet infiltration class (1000mm) (GLO) | Refer to Item 1 |
| 44 | Polyvinylchloride Collected by Informal Waste Collectors | 0 |  | kg | 70% plastic, recycled  24% treatment of waste polyvinylchloride, sanitary landfill (RoW)  4.8% treatment of waste polyvinylchloride, open dump, very wet infiltration class (300mm) (GLO)  1.2% treatment of waste polyvinylchloride, open dump, very wet infiltration class (1000mm) (GLO) | Refer to Item 1 |
| 45 | Polystyrene Collected by Informal Waste Collectors | 0 |  | kg | 70% plastic, recycled  24% treatment of waste polystyrene, sanitary landfill (RoW)  4.8% treatment of waste polystyrene, open dump, very wet infiltration class (300mm) (GLO)  1.2% treatment of waste polystyrene, open dump, very wet infiltration class (1000mm) (GLO) | Refer to Item 1 |
| 46 | Not specified Plastics Collected by Informal Waste Collectors | 0.25 |  | kg | 70% plastic, recycled  24% treatment of waste plastic, mixture, sanitary landfill (RoW)  4.8% treatment of waste plastic, mixture, open dump, very wet infiltration class (300mm) (GLO)  1.2% treatment of waste plastic mixture, open dump, very wet infiltration class (1000mm) (GLO) | Refer to Item 1 |
| 47 | Polyethylene Other End-of-Life |  |  | kg | 31% Polyethylene Collected by Formal Waste Collectors  8% Polyethylene Collected by Formal Waste Collectors  61% Uncollected Waste (59% treatment of waste polyethylene, open burning (GLO)  32.8% treatment of waste polyethylene, open dump, very wet infiltration class (300mm) (GLO)  8.2% treatment of polyethylene, open dump, very wet infiltration class (1000mm) (GLO)) | Refer to items 35 &41 |
| 48 | Polypropylene Other End-of-Life | 0 |  | kg | 31% Polypropylene Collected by Formal Waste Collectors  8% Polypropylene Collected by Formal Waste Collectors  61% Uncollected Waste (59% treatment of waste polypropylene, open burning (GLO)  32.8% treatment of waste polypropylene, open dump, very wet infiltration class (300mm) (GLO)  8.2% treatment of polypropylene, open dump, very wet infiltration class (1000mm) (GLO)) | Refer to items 36 &42 |
| 49 | Polyethylene Terephthalate Other End-of-Life | 0 |  | kg | 31% Polyethylene Terephthalate Collected by Formal Waste Collectors  8% Polyethylene Terephthalate Collected by Formal Waste Collectors  61% Uncollected Waste (59% treatment of waste polyethylene terephthalate, open burning (GLO)  32.8% treatment of waste polyethylene terephthalate, open dump, very wet infiltration class (300mm) (GLO)  8.2% treatment of polyethylene terephthalate, open dump, very wet infiltration class (1000mm) (GLO)) | Refer to items 37 &43 |
| 50 | Polyvinylchloride Other End-of-Life | 0 |  | kg | 31% Polyvinylchloride Collected by Formal Waste Collectors  8% Polyvinylchloride Collected by Formal Waste Collectors  61% Uncollected Waste (59% treatment of waste polyvinylchloride, open burning (GLO)  32.8% treatment of waste polyvinylchloride, open dump, very wet infiltration class (300mm) (GLO)  8.2% treatment of polyvinylchloride, open dump, very wet infiltration class (1000mm) (GLO)) | Refer to items 38 &44 |
| 51 | Polystyrene Other End-of-Life | 0 |  | kg | 31% Polystyrene Collected by Formal Waste Collectors  8% Polystyrene Collected by Formal Waste Collectors  61% Uncollected Waste (59% treatment of waste polystyrene, open burning (GLO)  32.8% treatment of waste polystyrene, open dump, very wet infiltration class (300mm) (GLO)  8.2% treatment of polystyrene, open dump, very wet infiltration class (1000mm) (GLO)) | Refer to items 39 &45 |
| 52 | Not specified Plastics Other End-of-Life | 0 |  | kg | 31% Not specified Plastic Collected by Formal Waste Collectors  8% Not specified Plastic Collected by Formal Waste Collectors  61% Uncollected Waste (59% treatment of waste plastic, mixture, open burning (GLO)  32.8% treatment of waste plastic, mixture, open dump, very wet infiltration class (300mm) (GLO)  8.2% treatment of plastic, mixture, open dump, very wet infiltration class (1000mm) (GLO)) | Refer to items 40 &46 |

## 2.2. Alternative Interventions

Table S13: Bakalan Recycling

| No. | Description | Input | Output | Unit | Ecoinvent 3.10 | Comment |
| --- | --- | --- | --- | --- | --- | --- |
| 1 | Recycling Process | 1 |  | kg | -0.0001 kg market for waste plastic, consumer electronics, unsorted (RoW)  0.1915kWh market for electricity, low voltage (ID)  0.0078 kg market for liquefied petroleum gas (RoW)  1.0001 kg market for plastic flake, consumer electronics, for recycling (GLO) |  |
| 2 | Avoided production of high density polyethylene | -0.19 |  | kg | Market for polyethylene production, high density, granulate (RoW) |  |
| 3 | Avoided production of low density polyethylene | -0.32 |  | kg | Market for polyethylene production, low density, granulate (RoW) |  |
|  | Avoided production of polypropylene | -0.11 |  | kg | Market for polypropylene production, granulate (RoW) |  |
| 4 | Avoided production of polyethylene terephthalate | -0.17 |  | kg | Market for polyethylene terephthalate, granulate, amorphous, recycled (RoW) |  |
| 5 | Avoided production of polyvinylchloride | -0.08 |  | kg | Market for polyvinylchloride production, granulate (RoW) |  |
| 6 | Avoided production of polystyrene | -0.05 |  |  | Market for polystyrene foam slab production (RoW) |  |
| 7 | Avoided production of expandable polystyrene | -0.03 |  | kg | Market for polystyrene production, expandable (RoW) |  |

Table S14: Bakalan Landfilling

| No. | Description | Input | Output | Unit | Ecoinvent 3.10 | Comment |
| --- | --- | --- | --- | --- | --- | --- |
| 1 | Landfilling of Polyethylene | -0.51 |  | kg | treatment of waste polyethylene, sanitary landfill (RoW) |  |
| 2 | Landfilling of Polypropylene | -0.11 |  | kg | treatment of waste polypropylene, sanitary landfill (RoW) |  |
| 3 | Landfilling of Polyethylene Terephthalate | -0.17 |  | kg | treatment of waste polyethylene terephthalate, sanitary landfill (RoW) |  |
| 4 | Landfilling of Polyvinylchloride | -0.08 |  | kg | treatment of waste polyvinylchloride, sanitary landfill (RoW) |  |
| 5 | Landfilling of Polystyrene | -0.083 |  | kg | treatment of waste polystyrene, sanitary landfill (RoW) |  |
| 6 | Landfilling of Not specified Plastics | -0.0044 |  | kg | treatment of waste plastic, mixture, sanitary landfill (RoW) |  |

Table S15: Bakalan Incineration

| No. | Description | Input | Output | Unit | Ecoinvent 3.10 | Comment |
| --- | --- | --- | --- | --- | --- | --- |
| 1 | Polyethylene Incinerated | -0.51 |  | kg | Waste polyethylene / treatment of waste polyethylene, municipal incineration (GLO) |  |
| 2 | Reused heat from polyethylene incineration | -5.45 |  | MJ | Heat, for reuse in municipal waste incineration only / treatment of waste polyethylene, municipal incineration (GLO) |  |
| 3 | Polypropylene Incinerated | -0.11 |  | kg | Waste polypropylene / treatment of waste polypropylene, municipal incineration (GLO) |  |
| 4 | Reused heat from polypropylene incineration | -0.89 |  | MJ | Heat, for reuse in municipal waste incineration only / treatment of waste polypropylene, municipal incineration (GLO) |  |
| 5 | Polyethylene terephthalate Incinerated | -0.17 |  | kg | Waste polyethylene terephthalate / treatment of waste polyethylene terephthalate, municipal incineration (GLO) |  |
| 6 | Reused heat from polyethylene terephthalate incineration | -0.99 |  | MJ | Heat, for reuse in municipal waste incineration only / treatment of waste polyethylene terephthalate, municipal incineration (GLO) |  |
| 7 | Polyvinylchloride Incinerated | -0.08 |  | kg | Waste polyvinylchloride/ treatment of waste polyvinylchloride, municipal incineration (GLO) |  |
| 8 | Reused heat from polyvinylchloride incineration | 0 |  | MJ | Heat, for reuse in municipal waste incineration only / treatment of waste polyvinylchloride, municipal incineration (GLO) |  |
| 9 | Polystyrene Incinerated | -0.08 |  | kg | Waste polystyrene / treatment of waste polystyrene, municipal incineration |  |
| 10 | Reused heat from polystyrene incineration | 0 |  | MJ | Heat, for reuse in municipal waste incineration only / treatment of waste polystyrene, municipal incineration (GLO) |  |
| 11 | Avoided Electricity production | -1.05 |  | kWh | electricity, from municipal waste incineration to generic market for electricity, medium voltage (ID) |  |
| 12 | Avoided Heat production | -1.19 |  | MJ | / market for heat, for reuse in municipal waste incineration only (RoW) |  |

Table S16: Krebet Sengrong Recycling

| No. | Description | Input | Output | Unit | Ecoinvent 3.10 | Comment |
| --- | --- | --- | --- | --- | --- | --- |
| 1 | Recycling Process | 1 |  | kg | -0.0001 kg market for waste plastic, consumer electronics, unsorted (RoW)  0.1915kWh market for electricity, low voltage (ID)  0.0078 kg market for liquefied petroleum gas (RoW)  1.0001 kg market for plastic flake, consumer electronics, for recycling (GLO) |  |
| 2 | Avoided production of high density polyethylene | -0.13 |  | kg | Market for polyethylene production, high density, granulate (RoW) |  |
| 3 | Avoided production of low density polyethylene | -0.27 |  | kg | Market for polyethylene production, low density, granulate (RoW) |  |
|  | Avoided production of polypropylene | -0.16 |  | kg | Market for polypropylene production, granulate (RoW) |  |
| 4 | Avoided production of polyethylene terephthalate | -0.31 |  | kg | Market for polyethylene terephthalate, granulate, amorphous, recycled (RoW) |  |
| 5 | Avoided production of polyvinylchloride | -0.05 |  | kg | Market for polyvinylchloride production, granulate (RoW) |  |
| 6 | Avoided production of polystyrene | -0.03 |  |  | Market for polystyrene foam slab production (RoW) |  |
| 7 | Avoided production of expandable polystyrene | -0.04 |  | kg | Market for polystyrene production, expandable (RoW) |  |

Table S17: Krebet Sengrong Landfilling

| No. | Description | Input | Output | Unit | Ecoinvent 3.10 | Comment |
| --- | --- | --- | --- | --- | --- | --- |
| 1 | Landfilling of Polyethylene | -0.39 |  | kg | treatment of waste polyethylene, sanitary landfill (RoW) |  |
| 2 | Landfilling of Polypropylene | -0.16 |  | kg | treatment of waste polypropylene, sanitary landfill (RoW) |  |
| 3 | Landfilling of Polyethylene Terephthalate | -0.31 |  | kg | treatment of waste polyethylene terephthalate, sanitary landfill (RoW) |  |
| 4 | Landfilling of Polyvinylchloride | -0.04 |  | kg | treatment of waste polyvinylchloride, sanitary landfill (RoW) |  |
| 5 | Landfilling of Polystyrene | -0.04 |  | kg | treatment of waste polystyrene, sanitary landfill (RoW) |  |
| 6 | Landfilling of Not specified Plastics | -0.0036 |  | kg | treatment of waste plastic, mixture, sanitary landfill (RoW) |  |

Table S18: Krebet Sengrong Incineration

|  | Description | Input | Output | Unit | Ecoinvent 3.10 | Comment |
| --- | --- | --- | --- | --- | --- | --- |
| 1 | Polyethylene Incinerated | -0.3925 |  | kg | Waste polyethylene / treatment of waste polyethylene, municipal incineration (GLO) |  |
| 2 | Reused heat from polyethylene incineration | -4.2 |  | MJ | Heat, for reuse in municipal waste incineration only / treatment of waste polyethylene, municipal incineration (GLO) |  |
| 3 | Polypropylene Incinerated | -0.16 |  | kg | Waste polypropylene / treatment of waste polypropylene, municipal incineration (GLO) |  |
| 4 | Reused heat from polypropylene incineration | -1.3 |  | MJ | Heat, for reuse in municipal waste incineration only / treatment of waste polypropylene, municipal incineration (GLO) |  |
| 5 | Polyethylene terephthalate Incinerated | -0.32 |  | kg | Waste polyethylene terephthalate / treatment of waste polyethylene terephthalate, municipal incineration (GLO) |  |
| 6 | Reused heat from polyethylene terephthalate incineration | -1.86 |  | MJ | Heat, for reuse in municipal waste incineration only / treatment of waste polyethylene terephthalate, municipal incineration (GLO) |  |
| 7 | Polyvinylchloride Incinerated | -0.05 |  | kg | Waste polyvinylchloride/ treatment of waste polyvinylchloride, municipal incineration (GLO) |  |
| 8 | Reused heat from polyvinylchloride incineration | 0 |  | MJ | Heat, for reuse in municipal waste incineration only / treatment of waste polyvinylchloride, municipal incineration (GLO) |  |
| 9 | Polystyrene Incinerated | -0.07 |  | kg | Waste polystyrene / treatment of waste polystyrene, municipal incineration |  |
| 10 | Reused heat from polystyrene incineration | 0 |  | MJ | Heat, for reuse in municipal waste incineration only / treatment of waste polystyrene, municipal incineration (GLO) |  |
| 11 | Avoided Electricity production | -1.05 |  | kWh | electricity, from municipal waste incineration to generic market for electricity, medium voltage (ID) |  |
| 12 | Avoided Heat production | -7.36 |  | MJ | market for heat, for reuse in municipal waste incineration only (RoW) |  |

Table S19: Krebet Recycling

| No. | Description | Input | Output | Unit | Ecoinvent 3.10 | Comment |
| --- | --- | --- | --- | --- | --- | --- |
| 1 | Recycling Process | 1 |  | kg | -0.0001 kg market for waste plastic, consumer electronics, unsorted (RoW)  0.1915kWh market for electricity, low voltage (ID)  0.0078 kg market for liquefied petroleum gas (RoW)  1.0001 kg market for plastic flake, consumer electronics, for recycling (GLO) |  |
| 2 | Avoided production of high density polyethylene | -0.12 |  | kg | Market for polyethylene production, high density, granulate (RoW) |  |
| 3 | Avoided production of low density polyethylene | -0.42 |  | kg | Market for polyethylene production, low density, granulate (RoW) |  |
|  | Avoided production of polypropylene | -0.13 |  | kg | Market for polypropylene production, granulate (RoW) |  |
| 4 | Avoided production of polyethylene terephthalate | -0.19 |  | kg | Market for polyethylene terephthalate, granulate, amorphous, recycled (RoW) |  |
| 5 | Avoided production of polyvinylchloride | -0.04 |  | kg | Market for polyvinylchloride production, granulate (RoW) |  |
| 6 | Avoided production of polystyrene | -0.02 |  |  | Market for polystyrene foam slab production (RoW) |  |
| 7 | Avoided production of expandable polystyrene | -0.08 |  | kg | Market for polystyrene production, expandable (RoW) |  |

Table S20: Krebet Landfilling

| No. | Description | Input | Output | Unit | Ecoinvent 3.10 | Comment |
| --- | --- | --- | --- | --- | --- | --- |
| 1 | Landfilling of Polyethylene | -0.54 |  | kg | treatment of waste polyethylene, sanitary landfill (RoW) |  |
| 2 | Landfilling of Polypropylene | -0.13 |  | kg | treatment of waste polypropylene, sanitary landfill (RoW) |  |
| 3 | Landfilling of Polyethylene Terephthalate | -0.2 |  | kg | treatment of waste polyethylene terephthalate, sanitary landfill (RoW) |  |
| 4 | Landfilling of Polyvinylchloride | -0.04 |  | kg | treatment of waste polyvinylchloride, sanitary landfill (RoW) |  |
| 5 | Landfilling of Polystyrene | -0.02 |  | kg | treatment of waste polystyrene, sanitary landfill (RoW) |  |
| 6 | Landfilling of Not specified Plastics | -0.07 |  | kg | treatment of waste plastic, mixture, sanitary landfill (RoW) |  |

Table S21: Krebet Incineration

| No. | Description | Input | Output | Unit | Ecoinvent 3.10 | Comment |
| --- | --- | --- | --- | --- | --- | --- |
| 1 | Polyethylene Incinerated | -0.54 |  | kg | Waste polyethylene / treatment of waste polyethylene, municipal incineration (GLO) |  |
| 2 | Reused heat from polyethylene incineration | -5.6 |  | MJ | Heat, for reuse in municipal waste incineration only / treatment of waste polyethylene, municipal incineration (GLO) |  |
| 3 | Polypropylene Incinerated | -0.13 |  | kg | Waste polypropylene / treatment of waste polypropylene, municipal incineration (GLO) |  |
| 4 | Reused heat from polypropylene incineration | 0 |  | MJ | Heat, for reuse in municipal waste incineration only / treatment of waste polypropylene, municipal incineration (GLO) |  |
| 5 | Polyethylene terephthalate Incinerated | -0.19 |  | kg | Waste polyethylene terephthalate / treatment of waste polyethylene terephthalate, municipal incineration (GLO) |  |
| 6 | Reused heat from polyethylene terephthalate incineration | 0 |  | MJ | Heat, for reuse in municipal waste incineration only / treatment of waste polyethylene terephthalate, municipal incineration (GLO) |  |
| 7 | Polyvinylchloride Incinerated | -0.03 |  | kg | Waste polyvinylchloride/ treatment of waste polyvinylchloride, municipal incineration (GLO) |  |
| 8 | Reused heat from polyvinylchloride incineration | 0 |  | MJ | Heat, for reuse in municipal waste incineration only / treatment of waste polyvinylchloride, municipal incineration (GLO) |  |
| 9 | Polystyrene Incinerated | -0.02 |  | kg | Waste polystyrene / treatment of waste polystyrene, municipal incineration |  |
| 10 | Reused heat from polystyrene incineration | 0 |  | MJ | Heat, for reuse in municipal waste incineration only / treatment of waste polystyrene, municipal incineration (GLO) |  |
| 11 | Avoided Electricity production | -1.141 |  | kWh | electricity, from municipal waste incineration to generic market for electricity, medium voltage (ID) |  |
| 12 | Avoided Heat production | -7.94 |  | MJ | market for heat, for reuse in municipal waste incineration only (RoW) |  |

Table S22: Tlogorejo Recycling

| No. | Description | Input | Output | Unit | Ecoinvent 3.10 | Comment |
| --- | --- | --- | --- | --- | --- | --- |
| 1 | Recycling Process | 1 |  | kg | -0.0001 kg market for waste plastic, consumer electronics, unsorted (RoW)  0.1915kWh market for electricity, low voltage (ID)  0.0078 kg market for liquefied petroleum gas (RoW)  1.0001 kg market for plastic flake, consumer electronics, for recycling (GLO) |  |
| 2 | Avoided production of high density polyethylene | -0.23 |  | kg | Market for polyethylene production, high density, granulate (RoW) |  |
| 3 | Avoided production of low density polyethylene | -0.21 |  | kg | Market for polyethylene production, low density, granulate (RoW) |  |
|  | Avoided production of polypropylene | -0.08 |  | kg | Market for polypropylene production, granulate (RoW) |  |
| 4 | Avoided production of polyethylene terephthalate | -0.28 |  | kg | Market for polyethylene terephthalate, granulate, amorphous, recycled (RoW) |  |
| 5 | Avoided production of polyvinylchloride | -0.0021 |  | kg | Market for polyvinylchloride production, granulate (RoW) |  |
| 6 | Avoided production of polystyrene | -0.14 |  |  | Market for polystyrene foam slab production (RoW) |  |
| 7 | Avoided production of expandable polystyrene | -0.05 |  | kg | Market for polystyrene production, expandable (RoW) |  |

Table S23: Tlogorejo Landfilling

| No | Description | Input | Output | Unit | Ecoinvent 3.10 | Comment |
| --- | --- | --- | --- | --- | --- | --- |
| 1 | Landfilling of Polyethylene | -0.44 |  | kg | treatment of waste polyethylene, sanitary landfill (RoW) |  |
| 2 | Landfilling of Polypropylene | -0.08 |  | kg | treatment of waste polypropylene, sanitary landfill (RoW) |  |
| 3 | Landfilling of Polyethylene Terephthalate | -0.28 |  | kg | treatment of waste polyethylene terephthalate, sanitary landfill (RoW) |  |
| 4 | Landfilling of Polyvinylchloride | -0.0021 |  | kg | treatment of waste polyvinylchloride, sanitary landfill (RoW) |  |
| 5 | Landfilling of Polystyrene | -0.14 |  | kg | treatment of waste polystyrene, sanitary landfill (RoW) |  |
| 6 | Landfilling of Not specified Plastics | -0.04 |  | kg | treatment of waste plastic, mixture, sanitary landfill (RoW) |  |

Table S24: Tlogorejo Incineration

| No. | Description | Input | Output | Unit | Ecoinvent 3.10 | Comment |
| --- | --- | --- | --- | --- | --- | --- |
| 1 | Polyethylene Incinerated | -0.44 |  | kg | Waste polyethylene / treatment of waste polyethylene, municipal incineration (GLO) |  |
| 2 | Reused heat from polyethylene incineration | -5.6 |  | MJ | Heat, for reuse in municipal waste incineration only / treatment of waste polyethylene, municipal incineration (GLO) |  |
| 3 | Polypropylene Incinerated | -0.08 |  | kg | Waste polypropylene / treatment of waste polypropylene, municipal incineration (GLO) |  |
| 4 | Reused heat from polypropylene incineration | 0 |  | MJ | Heat, for reuse in municipal waste incineration only / treatment of waste polypropylene, municipal incineration (GLO) |  |
| 5 | Polyethylene terephthalate Incinerated | -0.28 |  | kg | Waste polyethylene terephthalate / treatment of waste polyethylene terephthalate, municipal incineration (GLO) |  |
| 6 | Reused heat from polyethylene terephthalate incineration | 0 |  | MJ | Heat, for reuse in municipal waste incineration only / treatment of waste polyethylene terephthalate, municipal incineration (GLO) |  |
| 7 | Polyvinylchloride Incinerated | -0.0021 |  | kg | Waste polyvinylchloride/ treatment of waste polyvinylchloride, municipal incineration (GLO) |  |
| 8 | Reused heat from polyvinylchloride incineration | 0 |  | MJ | Heat, for reuse in municipal waste incineration only / treatment of waste polyvinylchloride, municipal incineration (GLO) |  |
| 9 | Polystyrene Incinerated | -0.181 |  | kg | Waste polystyrene / treatment of waste polystyrene, municipal incineration |  |
| 10 | Reused heat from polystyrene incineration | 0 |  | MJ | Heat, for reuse in municipal waste incineration only / treatment of waste polystyrene, municipal incineration (GLO) |  |
| 11 | Avoided Electricity production | -1 |  | kWh | electricity, from municipal waste incineration to generic market for electricity, medium voltage (ID) |  |
| 12 | Avoided Heat production | -6.98 |  | MJ | market for heat, for reuse in municipal waste incineration only (RoW) |  |

## 2.3. Current Scenario by Stakeholder Group

Table S25: Formal Waste Collector

| No. | Description | Input | Output | Unit | Activity(ies) Name in Ecoinvent v3.10 Database | Comment |
| --- | --- | --- | --- | --- | --- | --- |
| 1 | Recycling of Plastic | 0.0204 |  | kg | -0.01 kg of plastic flake, consumer electronics, unsorted  0.1915kWh market for electricity, low voltage (ID)  0.00778 kg market for liquefied petroleum gas (RoW)  1.01 kg of plastic flake, consumer for recycling (GLO) | From Neo et al 2021 |
| 2 | Incineration of Polyethylene | 0 |  | kg | treatment of waste polyethylene, municipal incineration (GLO) |  |
| 3 | Incineration of Polypropylene | 0 |  | kg | treatment of waste polypropylene, municipal incineration (GLO) |  |
| 4 | Incineration of Polyethylene Terephthalate | 0 |  | kg | treatment of waste polyethylene terephthalate, municipal incineration (GLO) |  |
| 5 | Incineration of Polyvinylchloride | 0 |  | kg | treatment of waste polyvinylchloride, municipal incineration (GLO) |  |
| 6 | Incineration of Polystyrene | 0 |  | kg | treatment of waste polystyrene, municipal incineration (GLO) |  |
| 7 | Incineration of Not specified Plastic | 0 |  | kg | treatment of waste plastic, mixture, municipal incineration (GLO) |  |
| 8 | Landfilling of Polyethylene | 0 |  | kg | treatment of waste polyethylene, sanitary landfill (RoW) |  |
| 9 | Landfilling of Polypropylene | 0 |  | kg | treatment of waste polypropylene, sanitary landfill (RoW) |  |
| 10 | Landfilling of Polyethylene Terephthalate | 0 |  | kg | treatment of waste polyethylene terephthalate, sanitary landfill (RoW) |  |
| 11 | Landfilling of Polyvinylchloride | 0 |  | kg | treatment of waste polyvinylchloride, sanitary landfill (RoW) |  |
| 12 | Landfilling of Polystyrene | 0 |  | kg | treatment of waste polystyrene, sanitary landfill (RoW) |  |
| 13 | Landfilling of Not specified Plastics | 0 |  | kg | treatment of waste plastic, mixture, sanitary landfill (RoW) |  |
| 14 | Open dumping (on land) of Polyethylene | 0 |  | kg | treatment of waste polyethylene, open dump, moist infiltration class (300mm) (GLO) |  |
| 15 | Open dumping (on land) of Polypropylene | 0 |  | kg | treatment of waste polypropylene, open dump, moist infiltration class (300mm) (GLO) |  |
| 16 | Open dumping (on land) of Polyethylene Terephthalate | 0 |  | kg | treatment of waste polyethylene terephthalate, open dump, moist infiltration class (300mm) (GLO) |  |
| 17 | Open dumping (on land) of Polyvinylchloride | 0 |  | kg | treatment of waste polyvinylchloride, open dump, moist infiltration class (300mm) (GLO) |  |
| 18 | Open dumping (on land) of Polystyrene | 0 |  | kg | treatment of waste polystyrene, open dump, moist infiltration class (300mm) (GLO) |  |
| 19 | Open dumping (on land) of Not specified Plastics | 0 |  | kg | treatment of waste plastic, mixture, open dump, moist infiltration class (300mm) (GLO) |  |
| 20 | Open burning of Polyethylene | 0 |  | kg | treatment of waste polyethylene, open burning (GLO) |  |
| 21 | Open burning of Polypropylene | 0 |  | kg | treatment of waste polypropylene, open burning (GLO) |  |
| 22 | Open burning of Polyethylene Terephthalate | 0 |  | kg | treatment of waste polyethylene terephthalate, open burning (GLO) |  |
| 23 | Open burning of Polyvinylchloride | 0 |  | kg | treatment of waste polyvinylchloride, open burning (GLO) |  |
| 24 | Open burning of Polystyrene | 0 |  | kg | treatment of waste polystyrene, open burning (GLO) |  |
| 25 | Open burning of Not specified Plastics | 0 |  | kg | treatment of waste plastic, mixture, open burning (GLO) |  |
| 26 | Open dump (in water) of Polyethylene | 0 |  | kg | treatment of waste polyethylene, open dump, very wet infiltration class (1000mm) (GLO) |  |
| 30 | Open dump (in water) of Polypropylene | 0 |  | kg | treatment of waste polypropylene, open dump, very wet infiltration class (1000mm) (GLO) |  |
| 31 | Open dump (in water) of Polyethylene Terephthalate | 0 |  | kg | treatment of waste polyethylene terephthalate, open dump, very wet infiltration class (1000mm) (GLO) |  |
| 32 | Open dump (in water) of Polyvinylchloride | 0 |  | kg | treatment of waste polyvinylchloride, open dump, very wet infiltration class (1000mm) (GLO) |  |
| 33 | Open dump (in water) of Polystyrene | 0 |  | kg | treatment of waste polystyrene, open dump, very wet infiltration class (1000mm) (GLO) |  |
| 34 | Open dump (in water) of Not specified Plastics | 0 |  | kg | treatment of waste plastic, mixture, open dump, very wet infiltration class (1000mm) (GLO) |  |
| 35 | Polyethylene Collected by Formal Waste Collectors | 0 |  | kg | 79% treatment of waste polyethylene, sanitary landfill (RoW)  16% treatment of waste polyethylene, open dump, very wet infiltration class (300mm) (GLO)  4% treatment of waste polyethylene, open dump, very wet infiltration class (1000mm) (GLO) |  |
| 36 | Polypropylene Collected by Formal Waste Collectors | 0 |  | kg | 79% treatment of waste polypropylene, sanitary landfill (RoW)  16% treatment of waste polypropylene, open dump, very wet infiltration class (300mm) (GLO)  4% treatment of waste polypropylene, open dump, very wet infiltration class (1000mm) (GLO) |  |
| 37 | Polyethylene Terephthalate Collected by Formal Waste Collectors | 0 |  | kg | 79% treatment of waste polyethylene terephthalate, sanitary landfill (RoW)  16% treatment of waste polyethylene terephthalate, open dump, very wet infiltration class (300mm) (GLO)  4% treatment of waste polyethylene terephthalate, open dump, very wet infiltration class (1000mm) (GLO) |  |
| 38 | Polyvinylchloride Collected by Formal Waste Collectors | 0 |  | kg | 79% treatment of waste polyvinylchloride, sanitary landfill (RoW)  16% treatment of waste polyvinylchloride, open dump, very wet infiltration class (300mm) (GLO)  4% treatment of waste polyvinylchloride, open dump, very wet infiltration class (1000mm) (GLO) |  |
| 39 | Polystyrene Collected by Formal Waste Collectors | 0 |  | kg | 79% treatment of waste polystyrene, sanitary landfill (RoW)  16% treatment of waste polystyrene, open dump, very wet infiltration class (300mm) (GLO)  4% treatment of waste polystyrene, open dump, very wet infiltration class (1000mm) (GLO) |  |
| 40 | Not specified Plastics Collected by Formal Waste Collectors | 0 |  | kg | 79% treatment of waste plastic, mixture, sanitary landfill (RoW)  16% treatment of waste plastic, mixture, open dump, very wet infiltration class (300mm) (GLO)  4% treatment of waste plastic, mixture, open dump, very wet infiltration class (1000mm) (GLO) |  |
| 41 | Polyethylene Collected by Informal Waste Collectors | 0 |  | kg | 70% plastic, recycled  24% treatment of waste polyethylene, sanitary landfill (RoW)  4.8% treatment of waste polyethylene, open dump, very wet infiltration class (300mm) (GLO)  1.2% treatment of waste polyethylene, open dump, very wet infiltration class (1000mm) (GLO) | Refer to Item 1 |
| 42 | Polypropylene Collected by Informal Waste Collectors | 0 |  | kg | 70% plastic, recycled  24% treatment of waste polypropylene, sanitary landfill (RoW)  4.8% treatment of waste polypropylene, open dump, very wet infiltration class (300mm) (GLO)  1.2% treatment of waste polypropylene, open dump, very wet infiltration class (1000mm) (GLO) | Refer to Item 1 |
| 43 | Polyethylene Terephthalate Collected by Informal Waste Collectors | 0 |  | kg | 70% plastic, recycled  24% treatment of waste polyethylene terephthalate, sanitary landfill (RoW)  4.8% treatment of waste polyethylene terephthalate, open dump, very wet infiltration class (300mm) (GLO)  1.2% treatment of waste polyethylene, open dump, very wet infiltration class (1000mm) (GLO) | Refer to Item 1 |
| 44 | Polyvinylchloride Collected by Informal Waste Collectors | 0 |  | kg | 70% plastic, recycled  24% treatment of waste polyvinylchloride, sanitary landfill (RoW)  4.8% treatment of waste polyvinylchloride, open dump, very wet infiltration class (300mm) (GLO)  1.2% treatment of waste polyvinylchloride, open dump, very wet infiltration class (1000mm) (GLO) | Refer to Item 1 |
| 45 | Polystyrene Collected by Informal Waste Collectors | 0 |  | kg | 70% plastic, recycled  24% treatment of waste polystyrene, sanitary landfill (RoW)  4.8% treatment of waste polystyrene, open dump, very wet infiltration class (300mm) (GLO)  1.2% treatment of waste polystyrene, open dump, very wet infiltration class (1000mm) (GLO) | Refer to Item 1 |
| 46 | Not specified Plastics Collected by Informal Waste Collectors | 0 |  | kg | 70% plastic, recycled  24% treatment of waste plastic, mixture, sanitary landfill (RoW)  4.8% treatment of waste plastic, mixture, open dump, very wet infiltration class (300mm) (GLO)  1.2% treatment of waste plastic mixture, open dump, very wet infiltration class (1000mm) (GLO) | Refer to Item 1 |
| 47 | Polyethylene Other End-of-Life | 0.7 |  | kg | 31% Polyethylene Collected by Formal Waste Collectors  8% Polyethylene Collected by Formal Waste Collectors  61% Uncollected Waste (59% treatment of waste polyethylene, open burning (GLO)  32.8% treatment of waste polyethylene, open dump, very wet infiltration class (300mm) (GLO)  8.2% treatment of polyethylene, open dump, very wet infiltration class (1000mm) (GLO)) | Refer to items 35 &41 |
| 48 | Polypropylene Other End-of-Life | 0.00141 |  | kg | 31% Polypropylene Collected by Formal Waste Collectors  8% Polypropylene Collected by Formal Waste Collectors  61% Uncollected Waste (59% treatment of waste polypropylene, open burning (GLO)  32.8% treatment of waste polypropylene, open dump, very wet infiltration class (300mm) (GLO)  8.2% treatment of polypropylene, open dump, very wet infiltration class (1000mm) (GLO)) | Refer to items 36 &42 |
| 49 | Polyethylene Terephthalate Other End-of-Life | 0.0125 |  | kg | 31% Polyethylene Terephthalate Collected by Formal Waste Collectors  8% Polyethylene Terephthalate Collected by Formal Waste Collectors  61% Uncollected Waste (59% treatment of waste polyethylene terephthalate, open burning (GLO)  32.8% treatment of waste polyethylene terephthalate, open dump, very wet infiltration class (300mm) (GLO)  8.2% treatment of polyethylene terephthalate, open dump, very wet infiltration class (1000mm) (GLO)) | Refer to items 37 &43 |
| 50 | Polyvinylchloride Other End-of-Life | 0.00235 |  | kg | 31% Polyvinylchloride Collected by Formal Waste Collectors  8% Polyvinylchloride Collected by Formal Waste Collectors  61% Uncollected Waste (59% treatment of waste polyvinylchloride, open burning (GLO)  32.8% treatment of waste polyvinylchloride, open dump, very wet infiltration class (300mm) (GLO)  8.2% treatment of polyvinylchloride, open dump, very wet infiltration class (1000mm) (GLO)) | Refer to items 38 &44 |
| 51 | Polystyrene Other End-of-Life | 0.249 |  | kg | 31% Polystyrene Collected by Formal Waste Collectors  8% Polystyrene Collected by Formal Waste Collectors  61% Uncollected Waste (59% treatment of waste polystyrene, open burning (GLO)  32.8% treatment of waste polystyrene, open dump, very wet infiltration class (300mm) (GLO)  8.2% treatment of polystyrene, open dump, very wet infiltration class (1000mm) (GLO)) | Refer to items 39 &45 |
| 52 | Not specified Plastics Other End-of-Life | 0.002742 |  | kg | 31% Not specified Plastic Collected by Formal Waste Collectors  8% Not specified Plastic Collected by Formal Waste Collectors  61% Uncollected Waste (59% treatment of waste plastic, mixture, open burning (GLO)  32.8% treatment of waste plastic, mixture, open dump, very wet infiltration class (300mm) (GLO)  8.2% treatment of plastic, mixture, open dump, very wet infiltration class (1000mm) (GLO)) | Refer to items 40 &46 |
| 53 | Transport with car | 0.185 |  | km | Transport, passenger car, medium size, diesel, EURO 4 (RoW) |  |

Table S26: Formal Waste Picker

| No. | Description | Input | Output | Unit | Activity(ies) Name in Ecoinvent v3.10 Database | Comment |
| --- | --- | --- | --- | --- | --- | --- |
| 1 | Recycling of Plastic | 0 |  | kg | -0.01 kg of plastic flake, consumer electronics, unsorted  0.1915kWh market for electricity, low voltage (ID)  0.00778 kg market for liquefied petroleum gas (RoW)  1.01 kg of plastic flake, consumer for recycling (GLO) | From Neo et al 2021 |
| 2 | Incineration of Polyethylene | 0 |  | kg | treatment of waste polyethylene, municipal incineration (GLO) |  |
| 3 | Incineration of Polypropylene | 0 |  | kg | treatment of waste polypropylene, municipal incineration (GLO) |  |
| 4 | Incineration of Polyethylene Terephthalate | 0 |  | kg | treatment of waste polyethylene terephthalate, municipal incineration (GLO) |  |
| 5 | Incineration of Polyvinylchloride | 0 |  | kg | treatment of waste polyvinylchloride, municipal incineration (GLO) |  |
| 6 | Incineration of Polystyrene | 0 |  | kg | treatment of waste polystyrene, municipal incineration (GLO) |  |
| 7 | Incineration of Not specified Plastic | 0 |  | kg | treatment of waste plastic, mixture, municipal incineration (GLO) |  |
| 8 | Landfilling of Polyethylene | 0 |  | kg | treatment of waste polyethylene, sanitary landfill (RoW) |  |
| 9 | Landfilling of Polypropylene | 0 |  | kg | treatment of waste polypropylene, sanitary landfill (RoW) |  |
| 10 | Landfilling of Polyethylene Terephthalate | 0 |  | kg | treatment of waste polyethylene terephthalate, sanitary landfill (RoW) |  |
| 11 | Landfilling of Polyvinylchloride | 0 |  | kg | treatment of waste polyvinylchloride, sanitary landfill (RoW) |  |
| 12 | Landfilling of Polystyrene | 0 |  | kg | treatment of waste polystyrene, sanitary landfill (RoW) |  |
| 13 | Landfilling of Not specified Plastics | 0 |  | kg | treatment of waste plastic, mixture, sanitary landfill (RoW) |  |
| 14 | Open dumping (on land) of Polyethylene | -0.001 |  | kg | treatment of waste polyethylene, open dump, moist infiltration class (300mm) (GLO) |  |
| 15 | Open dumping (on land) of Polypropylene | 0 |  | kg | treatment of waste polypropylene, open dump, moist infiltration class (300mm) (GLO) |  |
| 16 | Open dumping (on land) of Polyethylene Terephthalate | 0 |  | kg | treatment of waste polyethylene terephthalate, open dump, moist infiltration class (300mm) (GLO) |  |
| 17 | Open dumping (on land) of Polyvinylchloride | 0 |  | kg | treatment of waste polyvinylchloride, open dump, moist infiltration class (300mm) (GLO) |  |
| 18 | Open dumping (on land) of Polystyrene | 0 |  | kg | treatment of waste polystyrene, open dump, moist infiltration class (300mm) (GLO) |  |
| 19 | Open dumping (on land) of Not specified Plastics | 0 |  | kg | treatment of waste plastic, mixture, open dump, moist infiltration class (300mm) (GLO) |  |
| 20 | Open burning of Polyethylene | 0 |  | kg | treatment of waste polyethylene, open burning (GLO) |  |
| 21 | Open burning of Polypropylene | 0 |  | kg | treatment of waste polypropylene, open burning (GLO) |  |
| 22 | Open burning of Polyethylene Terephthalate | 0 |  | kg | treatment of waste polyethylene terephthalate, open burning (GLO) |  |
| 23 | Open burning of Polyvinylchloride | -0.000683 |  | kg | treatment of waste polyvinylchloride, open burning (GLO) |  |
| 24 | Open burning of Polystyrene | 0 |  | kg | treatment of waste polystyrene, open burning (GLO) |  |
| 25 | Open burning of Not specified Plastics | 0 |  | kg | treatment of waste plastic, mixture, open burning (GLO) |  |
| 26 | Open dump (in water) of Polyethylene | 0 |  | kg | treatment of waste polyethylene, open dump, very wet infiltration class (1000mm) (GLO) |  |
| 30 | Open dump (in water) of Polypropylene | 0 |  | kg | treatment of waste polypropylene, open dump, very wet infiltration class (1000mm) (GLO) |  |
| 31 | Open dump (in water) of Polyethylene Terephthalate | 0 |  | kg | treatment of waste polyethylene terephthalate, open dump, very wet infiltration class (1000mm) (GLO) |  |
| 32 | Open dump (in water) of Polyvinylchloride | 0 |  | kg | treatment of waste polyvinylchloride, open dump, very wet infiltration class (1000mm) (GLO) |  |
| 33 | Open dump (in water) of Polystyrene | 0 |  | kg | treatment of waste polystyrene, open dump, very wet infiltration class (1000mm) (GLO) |  |
| 34 | Open dump (in water) of Not specified Plastics | 0 |  | kg | treatment of waste plastic, mixture, open dump, very wet infiltration class (1000mm) (GLO) |  |
| 35 | Polyethylene Collected by Formal Waste Collectors | 0 |  | kg | 79% treatment of waste polyethylene, sanitary landfill (RoW)  16% treatment of waste polyethylene, open dump, very wet infiltration class (300mm) (GLO)  4% treatment of waste polyethylene, open dump, very wet infiltration class (1000mm) (GLO) |  |
| 36 | Polypropylene Collected by Formal Waste Collectors | 0 |  | kg | 79% treatment of waste polypropylene, sanitary landfill (RoW)  16% treatment of waste polypropylene, open dump, very wet infiltration class (300mm) (GLO)  4% treatment of waste polypropylene, open dump, very wet infiltration class (1000mm) (GLO) |  |
| 37 | Polyethylene Terephthalate Collected by Formal Waste Collectors | 0 |  | kg | 79% treatment of waste polyethylene terephthalate, sanitary landfill (RoW)  16% treatment of waste polyethylene terephthalate, open dump, very wet infiltration class (300mm) (GLO)  4% treatment of waste polyethylene terephthalate, open dump, very wet infiltration class (1000mm) (GLO) |  |
| 38 | Polyvinylchloride Collected by Formal Waste Collectors | 0 |  | kg | 79% treatment of waste polyvinylchloride, sanitary landfill (RoW)  16% treatment of waste polyvinylchloride, open dump, very wet infiltration class (300mm) (GLO)  4% treatment of waste polyvinylchloride, open dump, very wet infiltration class (1000mm) (GLO) |  |
| 39 | Polystyrene Collected by Formal Waste Collectors | 0 |  | kg | 79% treatment of waste polystyrene, sanitary landfill (RoW)  16% treatment of waste polystyrene, open dump, very wet infiltration class (300mm) (GLO)  4% treatment of waste polystyrene, open dump, very wet infiltration class (1000mm) (GLO) |  |
| 40 | Not specified Plastics Collected by Formal Waste Collectors | 0 |  | kg | 79% treatment of waste plastic, mixture, sanitary landfill (RoW)  16% treatment of waste plastic, mixture, open dump, very wet infiltration class (300mm) (GLO)  4% treatment of waste plastic, mixture, open dump, very wet infiltration class (1000mm) (GLO) |  |
| 41 | Polyethylene Collected by Informal Waste Collectors | 0 |  | kg | 70% plastic, recycled  24% treatment of waste polyethylene, sanitary landfill (RoW)  4.8% treatment of waste polyethylene, open dump, very wet infiltration class (300mm) (GLO)  1.2% treatment of waste polyethylene, open dump, very wet infiltration class (1000mm) (GLO) | Refer to Item 1 |
| 42 | Polypropylene Collected by Informal Waste Collectors | 0 |  | kg | 70% plastic, recycled  24% treatment of waste polypropylene, sanitary landfill (RoW)  4.8% treatment of waste polypropylene, open dump, very wet infiltration class (300mm) (GLO)  1.2% treatment of waste polypropylene, open dump, very wet infiltration class (1000mm) (GLO) | Refer to Item 1 |
| 43 | Polyethylene Terephthalate Collected by Informal Waste Collectors | 0 |  | kg | 70% plastic, recycled  24% treatment of waste polyethylene terephthalate, sanitary landfill (RoW)  4.8% treatment of waste polyethylene terephthalate, open dump, very wet infiltration class (300mm) (GLO)  1.2% treatment of waste polyethylene, open dump, very wet infiltration class (1000mm) (GLO) | Refer to Item 1 |
| 44 | Polyvinylchloride Collected by Informal Waste Collectors | 0 |  | kg | 70% plastic, recycled  24% treatment of waste polyvinylchloride, sanitary landfill (RoW)  4.8% treatment of waste polyvinylchloride, open dump, very wet infiltration class (300mm) (GLO)  1.2% treatment of waste polyvinylchloride, open dump, very wet infiltration class (1000mm) (GLO) | Refer to Item 1 |
| 45 | Polystyrene Collected by Informal Waste Collectors | 0 |  | kg | 70% plastic, recycled  24% treatment of waste polystyrene, sanitary landfill (RoW)  4.8% treatment of waste polystyrene, open dump, very wet infiltration class (300mm) (GLO)  1.2% treatment of waste polystyrene, open dump, very wet infiltration class (1000mm) (GLO) | Refer to Item 1 |
| 46 | Not specified Plastics Collected by Informal Waste Collectors | 0 |  | kg | 70% plastic, recycled  24% treatment of waste plastic, mixture, sanitary landfill (RoW)  4.8% treatment of waste plastic, mixture, open dump, very wet infiltration class (300mm) (GLO)  1.2% treatment of waste plastic mixture, open dump, very wet infiltration class (1000mm) (GLO) | Refer to Item 1 |
| 47 | Polyethylene Other End-of-Life | 0.359 |  | kg | 31% Polyethylene Collected by Formal Waste Collectors  8% Polyethylene Collected by Formal Waste Collectors  61% Uncollected Waste (59% treatment of waste polyethylene, open burning (GLO)  32.8% treatment of waste polyethylene, open dump, very wet infiltration class (300mm) (GLO)  8.2% treatment of polyethylene, open dump, very wet infiltration class (1000mm) (GLO)) | Refer to items 35 &41 |
| 48 | Polypropylene Other End-of-Life | 0.23 |  | kg | 31% Polypropylene Collected by Formal Waste Collectors  8% Polypropylene Collected by Formal Waste Collectors  61% Uncollected Waste (59% treatment of waste polypropylene, open burning (GLO)  32.8% treatment of waste polypropylene, open dump, very wet infiltration class (300mm) (GLO)  8.2% treatment of polypropylene, open dump, very wet infiltration class (1000mm) (GLO)) | Refer to items 36 &42 |
| 49 | Polyethylene Terephthalate Other End-of-Life | 0.3 |  | kg | 31% Polyethylene Terephthalate Collected by Formal Waste Collectors  8% Polyethylene Terephthalate Collected by Formal Waste Collectors  61% Uncollected Waste (59% treatment of waste polyethylene terephthalate, open burning (GLO)  32.8% treatment of waste polyethylene terephthalate, open dump, very wet infiltration class (300mm) (GLO)  8.2% treatment of polyethylene terephthalate, open dump, very wet infiltration class (1000mm) (GLO)) | Refer to items 37 &43 |
| 50 | Polyvinylchloride Other End-of-Life | 0.0114 |  | kg | 31% Polyvinylchloride Collected by Formal Waste Collectors  8% Polyvinylchloride Collected by Formal Waste Collectors  61% Uncollected Waste (59% treatment of waste polyvinylchloride, open burning (GLO)  32.8% treatment of waste polyvinylchloride, open dump, very wet infiltration class (300mm) (GLO)  8.2% treatment of polyvinylchloride, open dump, very wet infiltration class (1000mm) (GLO)) | Refer to items 38 &44 |
| 51 | Polystyrene Other End-of-Life | 0.06 |  | kg | 31% Polystyrene Collected by Formal Waste Collectors  8% Polystyrene Collected by Formal Waste Collectors  61% Uncollected Waste (59% treatment of waste polystyrene, open burning (GLO)  32.8% treatment of waste polystyrene, open dump, very wet infiltration class (300mm) (GLO)  8.2% treatment of polystyrene, open dump, very wet infiltration class (1000mm) (GLO)) | Refer to items 39 &45 |
| 52 | Not specified Plastics Other End-of-Life | 0.003944 |  | kg | 31% Not specified Plastic Collected by Formal Waste Collectors  8% Not specified Plastic Collected by Formal Waste Collectors  61% Uncollected Waste (59% treatment of waste plastic, mixture, open burning (GLO)  32.8% treatment of waste plastic, mixture, open dump, very wet infiltration class (300mm) (GLO)  8.2% treatment of plastic, mixture, open dump, very wet infiltration class (1000mm) (GLO)) | Refer to items 40 &46 |
| 53 | Transport with scooter | 0.523 |  | Person km | Transport, passenger, motor scooter (RoW) |  |

Table S27: Household

| No. | Description | Input | Output | Unit | Activity(ies) Name in Ecoinvent v3.10 Database | Comment |
| --- | --- | --- | --- | --- | --- | --- |
| 1 | Recycling of Plastic | 0 |  | kg | -0.01 kg of plastic flake, consumer electronics, unsorted  0.1915kWh market for electricity, low voltage (ID)  0.00778 kg market for liquefied petroleum gas (RoW)  1.01 kg of plastic flake, consumer for recycling (GLO) | From Neo et al 2021 |
| 2 | Incineration of Polyethylene | 0 |  | kg | treatment of waste polyethylene, municipal incineration (GLO) |  |
| 3 | Incineration of Polypropylene | 0 |  | kg | treatment of waste polypropylene, municipal incineration (GLO) |  |
| 4 | Incineration of Polyethylene Terephthalate | 0 |  | kg | treatment of waste polyethylene terephthalate, municipal incineration (GLO) |  |
| 5 | Incineration of Polyvinylchloride | 0 |  | kg | treatment of waste polyvinylchloride, municipal incineration (GLO) |  |
| 6 | Incineration of Polystyrene | 0 |  | kg | treatment of waste polystyrene, municipal incineration (GLO) |  |
| 7 | Incineration of Not specified Plastic | 0 |  | kg | treatment of waste plastic, mixture, municipal incineration (GLO) |  |
| 8 | Landfilling of Polyethylene | 0 |  | kg | treatment of waste polyethylene, sanitary landfill (RoW) |  |
| 9 | Landfilling of Polypropylene | 0 |  | kg | treatment of waste polypropylene, sanitary landfill (RoW) |  |
| 10 | Landfilling of Polyethylene Terephthalate | 0 |  | kg | treatment of waste polyethylene terephthalate, sanitary landfill (RoW) |  |
| 11 | Landfilling of Polyvinylchloride | 0 |  | kg | treatment of waste polyvinylchloride, sanitary landfill (RoW) |  |
| 12 | Landfilling of Polystyrene | 0 |  | kg | treatment of waste polystyrene, sanitary landfill (RoW) |  |
| 13 | Landfilling of Not specified Plastics | 0 |  | kg | treatment of waste plastic, mixture, sanitary landfill (RoW) |  |
| 14 | Open dumping (on land) of Polyethylene | -0.11 |  | kg | treatment of waste polyethylene, open dump, moist infiltration class (300mm) (GLO) |  |
| 15 | Open dumping (on land) of Polypropylene | -0.03 |  | kg | treatment of waste polypropylene, open dump, moist infiltration class (300mm) (GLO) |  |
| 16 | Open dumping (on land) of Polyethylene Terephthalate | -0.05 |  | kg | treatment of waste polyethylene terephthalate, open dump, moist infiltration class (300mm) (GLO) |  |
| 17 | Open dumping (on land) of Polyvinylchloride | -0.02 |  | kg | treatment of waste polyvinylchloride, open dump, moist infiltration class (300mm) (GLO) |  |
| 18 | Open dumping (on land) of Polystyrene | -0.05 |  | kg | treatment of waste polystyrene, open dump, moist infiltration class (300mm) (GLO) |  |
| 19 | Open dumping (on land) of Not specified Plastics | -0.01 |  | kg | treatment of waste plastic, mixture, open dump, moist infiltration class (300mm) (GLO) |  |
| 20 | Open burning of Polyethylene | -0.2 |  | kg | treatment of waste polyethylene, open burning (GLO) |  |
| 21 | Open burning of Polypropylene | -0.06 |  | kg | treatment of waste polypropylene, open burning (GLO) |  |
| 22 | Open burning of Polyethylene Terephthalate | -0.09 |  | kg | treatment of waste polyethylene terephthalate, open burning (GLO) |  |
| 23 | Open burning of Polyvinylchloride | -0.01 |  | kg | treatment of waste polyvinylchloride, open burning (GLO) |  |
| 24 | Open burning of Polystyrene | 0 |  | kg | treatment of waste polystyrene, open burning (GLO) |  |
| 25 | Open burning of Not specified Plastics | 0 |  | kg | treatment of waste plastic, mixture, open burning (GLO) |  |
| 26 | Open dump (in water) of Polyethylene | 0 |  | kg | treatment of waste polyethylene, open dump, very wet infiltration class (1000mm) (GLO) |  |
| 30 | Open dump (in water) of Polypropylene | 0 |  | kg | treatment of waste polypropylene, open dump, very wet infiltration class (1000mm) (GLO) |  |
| 31 | Open dump (in water) of Polyethylene Terephthalate | 0 |  | kg | treatment of waste polyethylene terephthalate, open dump, very wet infiltration class (1000mm) (GLO) |  |
| 32 | Open dump (in water) of Polyvinylchloride | 0 |  | kg | treatment of waste polyvinylchloride, open dump, very wet infiltration class (1000mm) (GLO) |  |
| 33 | Open dump (in water) of Polystyrene | 0 |  | kg | treatment of waste polystyrene, open dump, very wet infiltration class (1000mm) (GLO) |  |
| 34 | Open dump (in water) of Not specified Plastics | 0 |  | kg | treatment of waste plastic, mixture, open dump, very wet infiltration class (1000mm) (GLO) |  |
| 35 | Polyethylene Collected by Formal Waste Collectors | 0.05 |  | kg | 79% treatment of waste polyethylene, sanitary landfill (RoW)  16% treatment of waste polyethylene, open dump, very wet infiltration class (300mm) (GLO)  4% treatment of waste polyethylene, open dump, very wet infiltration class (1000mm) (GLO) |  |
| 36 | Polypropylene Collected by Formal Waste Collectors | 0.02 |  | kg | 79% treatment of waste polypropylene, sanitary landfill (RoW)  16% treatment of waste polypropylene, open dump, very wet infiltration class (300mm) (GLO)  4% treatment of waste polypropylene, open dump, very wet infiltration class (1000mm) (GLO) |  |
| 37 | Polyethylene Terephthalate Collected by Formal Waste Collectors | 0.02 |  | kg | 79% treatment of waste polyethylene terephthalate, sanitary landfill (RoW)  16% treatment of waste polyethylene terephthalate, open dump, very wet infiltration class (300mm) (GLO)  4% treatment of waste polyethylene terephthalate, open dump, very wet infiltration class (1000mm) (GLO) |  |
| 38 | Polyvinylchloride Collected by Formal Waste Collectors | 0.01 |  | kg | 79% treatment of waste polyvinylchloride, sanitary landfill (RoW)  16% treatment of waste polyvinylchloride, open dump, very wet infiltration class (300mm) (GLO)  4% treatment of waste polyvinylchloride, open dump, very wet infiltration class (1000mm) (GLO) |  |
| 39 | Polystyrene Collected by Formal Waste Collectors | 0.03 |  | kg | 79% treatment of waste polystyrene, sanitary landfill (RoW)  16% treatment of waste polystyrene, open dump, very wet infiltration class (300mm) (GLO)  4% treatment of waste polystyrene, open dump, very wet infiltration class (1000mm) (GLO) |  |
| 40 | Not specified Plastics Collected by Formal Waste Collectors | 0 |  | kg | 79% treatment of waste plastic, mixture, sanitary landfill (RoW)  16% treatment of waste plastic, mixture, open dump, very wet infiltration class (300mm) (GLO)  4% treatment of waste plastic, mixture, open dump, very wet infiltration class (1000mm) (GLO) |  |
| 41 | Polyethylene Collected by Informal Waste Collectors | 0.06 |  | kg | 70% plastic, recycled  24% treatment of waste polyethylene, sanitary landfill (RoW)  4.8% treatment of waste polyethylene, open dump, very wet infiltration class (300mm) (GLO)  1.2% treatment of waste polyethylene, open dump, very wet infiltration class (1000mm) (GLO) | Refer to Item 1 |
| 42 | Polypropylene Collected by Informal Waste Collectors | 0.02 |  | kg | 70% plastic, recycled  24% treatment of waste polypropylene, sanitary landfill (RoW)  4.8% treatment of waste polypropylene, open dump, very wet infiltration class (300mm) (GLO)  1.2% treatment of waste polypropylene, open dump, very wet infiltration class (1000mm) (GLO) | Refer to Item 1 |
| 43 | Polyethylene Terephthalate Collected by Informal Waste Collectors | 0.02 |  | kg | 70% plastic, recycled  24% treatment of waste polyethylene terephthalate, sanitary landfill (RoW)  4.8% treatment of waste polyethylene terephthalate, open dump, very wet infiltration class (300mm) (GLO)  1.2% treatment of waste polyethylene, open dump, very wet infiltration class (1000mm) (GLO) | Refer to Item 1 |
| 44 | Polyvinylchloride Collected by Informal Waste Collectors | 0.01 |  | kg | 70% plastic, recycled  24% treatment of waste polyvinylchloride, sanitary landfill (RoW)  4.8% treatment of waste polyvinylchloride, open dump, very wet infiltration class (300mm) (GLO)  1.2% treatment of waste polyvinylchloride, open dump, very wet infiltration class (1000mm) (GLO) | Refer to Item 1 |
| 45 | Polystyrene Collected by Informal Waste Collectors | 0.03 |  | kg | 70% plastic, recycled  24% treatment of waste polystyrene, sanitary landfill (RoW)  4.8% treatment of waste polystyrene, open dump, very wet infiltration class (300mm) (GLO)  1.2% treatment of waste polystyrene, open dump, very wet infiltration class (1000mm) (GLO) | Refer to Item 1 |
| 46 | Not specified Plastics Collected by Informal Waste Collectors | 0 |  | kg | 70% plastic, recycled  24% treatment of waste plastic, mixture, sanitary landfill (RoW)  4.8% treatment of waste plastic, mixture, open dump, very wet infiltration class (300mm) (GLO)  1.2% treatment of waste plastic mixture, open dump, very wet infiltration class (1000mm) (GLO) | Refer to Item 1 |
| 47 | Polyethylene Other End-of-Life | 0 |  | kg | 31% Polyethylene Collected by Formal Waste Collectors  8% Polyethylene Collected by Formal Waste Collectors  61% Uncollected Waste (59% treatment of waste polyethylene, open burning (GLO)  32.8% treatment of waste polyethylene, open dump, very wet infiltration class (300mm) (GLO)  8.2% treatment of polyethylene, open dump, very wet infiltration class (1000mm) (GLO)) | Refer to items 35 &41 |
| 48 | Polypropylene Other End-of-Life | 0 |  | kg | 31% Polypropylene Collected by Formal Waste Collectors  8% Polypropylene Collected by Formal Waste Collectors  61% Uncollected Waste (59% treatment of waste polypropylene, open burning (GLO)  32.8% treatment of waste polypropylene, open dump, very wet infiltration class (300mm) (GLO)  8.2% treatment of polypropylene, open dump, very wet infiltration class (1000mm) (GLO)) | Refer to items 36 &42 |
| 49 | Polyethylene Terephthalate Other End-of-Life | 0 |  | kg | 31% Polyethylene Terephthalate Collected by Formal Waste Collectors  8% Polyethylene Terephthalate Collected by Formal Waste Collectors  61% Uncollected Waste (59% treatment of waste polyethylene terephthalate, open burning (GLO)  32.8% treatment of waste polyethylene terephthalate, open dump, very wet infiltration class (300mm) (GLO)  8.2% treatment of polyethylene terephthalate, open dump, very wet infiltration class (1000mm) (GLO)) | Refer to items 37 &43 |
| 50 | Polyvinylchloride Other End-of-Life | 0 |  | kg | 31% Polyvinylchloride Collected by Formal Waste Collectors  8% Polyvinylchloride Collected by Formal Waste Collectors  61% Uncollected Waste (59% treatment of waste polyvinylchloride, open burning (GLO)  32.8% treatment of waste polyvinylchloride, open dump, very wet infiltration class (300mm) (GLO)  8.2% treatment of polyvinylchloride, open dump, very wet infiltration class (1000mm) (GLO)) | Refer to items 38 &44 |
| 51 | Polystyrene Other End-of-Life | 0 |  | kg | 31% Polystyrene Collected by Formal Waste Collectors  8% Polystyrene Collected by Formal Waste Collectors  61% Uncollected Waste (59% treatment of waste polystyrene, open burning (GLO)  32.8% treatment of waste polystyrene, open dump, very wet infiltration class (300mm) (GLO)  8.2% treatment of polystyrene, open dump, very wet infiltration class (1000mm) (GLO)) | Refer to items 39 &45 |
| 52 | Not specified Plastics Other End-of-Life | 0 |  | kg | 31% Not specified Plastic Collected by Formal Waste Collectors  8% Not specified Plastic Collected by Formal Waste Collectors  61% Uncollected Waste (59% treatment of waste plastic, mixture, open burning (GLO)  32.8% treatment of waste plastic, mixture, open dump, very wet infiltration class (300mm) (GLO)  8.2% treatment of plastic, mixture, open dump, very wet infiltration class (1000mm) (GLO)) | Refer to items 40 &46 |

Table S28: Informal Waste Collectors

| No. | Description | Input | Output | Unit | Activity(ies) Name in Ecoinvent v3.10 Database | Comment |
| --- | --- | --- | --- | --- | --- | --- |
| 1 | Recycling of Plastic | 0.965 |  | kg | -0.01 kg of plastic flake, consumer electronics, unsorted  0.1915kWh market for electricity, low voltage (ID)  0.00778 kg market for liquefied petroleum gas (RoW)  1.01 kg of plastic flake, consumer for recycling (GLO) | From Neo et al 2021 |
| 2 | Incineration of Polyethylene | 0 |  | kg | treatment of waste polyethylene, municipal incineration (GLO) |  |
| 3 | Incineration of Polypropylene | 0 |  | kg | treatment of waste polypropylene, municipal incineration (GLO) |  |
| 4 | Incineration of Polyethylene Terephthalate | 0 |  | kg | treatment of waste polyethylene terephthalate, municipal incineration (GLO) |  |
| 5 | Incineration of Polyvinylchloride | 0 |  | kg | treatment of waste polyvinylchloride, municipal incineration (GLO) |  |
| 6 | Incineration of Polystyrene | 0 |  | kg | treatment of waste polystyrene, municipal incineration (GLO) |  |
| 7 | Incineration of Not specified Plastic | 0 |  | kg | treatment of waste plastic, mixture, municipal incineration (GLO) |  |
| 8 | Landfilling of Polyethylene | 0 |  | kg | treatment of waste polyethylene, sanitary landfill (RoW) |  |
| 9 | Landfilling of Polypropylene | 0 |  | kg | treatment of waste polypropylene, sanitary landfill (RoW) |  |
| 10 | Landfilling of Polyethylene Terephthalate | 0 |  | kg | treatment of waste polyethylene terephthalate, sanitary landfill (RoW) |  |
| 11 | Landfilling of Polyvinylchloride | 0 |  | kg | treatment of waste polyvinylchloride, sanitary landfill (RoW) |  |
| 12 | Landfilling of Polystyrene | 0 |  | kg | treatment of waste polystyrene, sanitary landfill (RoW) |  |
| 13 | Landfilling of Not specified Plastics | 0 |  | kg | treatment of waste plastic, mixture, sanitary landfill (RoW) |  |
| 14 | Open dumping (on land) of Polyethylene | 0 |  | kg | treatment of waste polyethylene, open dump, moist infiltration class (300mm) (GLO) |  |
| 15 | Open dumping (on land) of Polypropylene | 0 |  | kg | treatment of waste polypropylene, open dump, moist infiltration class (300mm) (GLO) |  |
| 16 | Open dumping (on land) of Polyethylene Terephthalate | 0 |  | kg | treatment of waste polyethylene terephthalate, open dump, moist infiltration class (300mm) (GLO) |  |
| 17 | Open dumping (on land) of Polyvinylchloride | 0 |  | kg | treatment of waste polyvinylchloride, open dump, moist infiltration class (300mm) (GLO) |  |
| 18 | Open dumping (on land) of Polystyrene | 0 |  | kg | treatment of waste polystyrene, open dump, moist infiltration class (300mm) (GLO) |  |
| 19 | Open dumping (on land) of Not specified Plastics | 0 |  | kg | treatment of waste plastic, mixture, open dump, moist infiltration class (300mm) (GLO) |  |
| 20 | Open burning of Polyethylene | -0.013 |  | kg | treatment of waste polyethylene, open burning (GLO) |  |
| 21 | Open burning of Polypropylene | -0.005 |  | kg | treatment of waste polypropylene, open burning (GLO) |  |
| 22 | Open burning of Polyethylene Terephthalate | -0.01 |  | kg | treatment of waste polyethylene terephthalate, open burning (GLO) |  |
| 23 | Open burning of Polyvinylchloride | 0 |  | kg | treatment of waste polyvinylchloride, open burning (GLO) |  |
| 24 | Open burning of Polystyrene | -0.001 |  | kg | treatment of waste polystyrene, open burning (GLO) |  |
| 25 | Open burning of Not specified Plastics | 0 |  | kg | treatment of waste plastic, mixture, open burning (GLO) |  |
| 26 | Open dump (in water) of Polyethylene | 0 |  | kg | treatment of waste polyethylene, open dump, very wet infiltration class (1000mm) (GLO) |  |
| 30 | Open dump (in water) of Polypropylene | 0 |  | kg | treatment of waste polypropylene, open dump, very wet infiltration class (1000mm) (GLO) |  |
| 31 | Open dump (in water) of Polyethylene Terephthalate | 0 |  | kg | treatment of waste polyethylene terephthalate, open dump, very wet infiltration class (1000mm) (GLO) |  |
| 32 | Open dump (in water) of Polyvinylchloride | 0 |  | kg | treatment of waste polyvinylchloride, open dump, very wet infiltration class (1000mm) (GLO) |  |
| 33 | Open dump (in water) of Polystyrene | 0 |  | kg | treatment of waste polystyrene, open dump, very wet infiltration class (1000mm) (GLO) |  |
| 34 | Open dump (in water) of Not specified Plastics | 0 |  | kg | treatment of waste plastic, mixture, open dump, very wet infiltration class (1000mm) (GLO) |  |
| 35 | Polyethylene Collected by Formal Waste Collectors | 0.05 |  | kg | 79% treatment of waste polyethylene, sanitary landfill (RoW)  16% treatment of waste polyethylene, open dump, very wet infiltration class (300mm) (GLO)  4% treatment of waste polyethylene, open dump, very wet infiltration class (1000mm) (GLO) |  |
| 36 | Polypropylene Collected by Formal Waste Collectors | 0.02 |  | kg | 79% treatment of waste polypropylene, sanitary landfill (RoW)  16% treatment of waste polypropylene, open dump, very wet infiltration class (300mm) (GLO)  4% treatment of waste polypropylene, open dump, very wet infiltration class (1000mm) (GLO) |  |
| 37 | Polyethylene Terephthalate Collected by Formal Waste Collectors | 0.02 |  | kg | 79% treatment of waste polyethylene terephthalate, sanitary landfill (RoW)  16% treatment of waste polyethylene terephthalate, open dump, very wet infiltration class (300mm) (GLO)  4% treatment of waste polyethylene terephthalate, open dump, very wet infiltration class (1000mm) (GLO) |  |
| 38 | Polyvinylchloride Collected by Formal Waste Collectors | 0.01 |  | kg | 79% treatment of waste polyvinylchloride, sanitary landfill (RoW)  16% treatment of waste polyvinylchloride, open dump, very wet infiltration class (300mm) (GLO)  4% treatment of waste polyvinylchloride, open dump, very wet infiltration class (1000mm) (GLO) |  |
| 39 | Polystyrene Collected by Formal Waste Collectors | 0.03 |  | kg | 79% treatment of waste polystyrene, sanitary landfill (RoW)  16% treatment of waste polystyrene, open dump, very wet infiltration class (300mm) (GLO)  4% treatment of waste polystyrene, open dump, very wet infiltration class (1000mm) (GLO) |  |
| 40 | Not specified Plastics Collected by Formal Waste Collectors | 0 |  | kg | 79% treatment of waste plastic, mixture, sanitary landfill (RoW)  16% treatment of waste plastic, mixture, open dump, very wet infiltration class (300mm) (GLO)  4% treatment of waste plastic, mixture, open dump, very wet infiltration class (1000mm) (GLO) |  |
| 41 | Polyethylene Collected by Informal Waste Collectors | 0.06 |  | kg | 70% plastic, recycled  24% treatment of waste polyethylene, sanitary landfill (RoW)  4.8% treatment of waste polyethylene, open dump, very wet infiltration class (300mm) (GLO)  1.2% treatment of waste polyethylene, open dump, very wet infiltration class (1000mm) (GLO) | Refer to Item 1 |
| 42 | Polypropylene Collected by Informal Waste Collectors | 0.02 |  | kg | 70% plastic, recycled  24% treatment of waste polypropylene, sanitary landfill (RoW)  4.8% treatment of waste polypropylene, open dump, very wet infiltration class (300mm) (GLO)  1.2% treatment of waste polypropylene, open dump, very wet infiltration class (1000mm) (GLO) | Refer to Item 1 |
| 43 | Polyethylene Terephthalate Collected by Informal Waste Collectors | 0.02 |  | kg | 70% plastic, recycled  24% treatment of waste polyethylene terephthalate, sanitary landfill (RoW)  4.8% treatment of waste polyethylene terephthalate, open dump, very wet infiltration class (300mm) (GLO)  1.2% treatment of waste polyethylene, open dump, very wet infiltration class (1000mm) (GLO) | Refer to Item 1 |
| 44 | Polyvinylchloride Collected by Informal Waste Collectors | 0.01 |  | kg | 70% plastic, recycled  24% treatment of waste polyvinylchloride, sanitary landfill (RoW)  4.8% treatment of waste polyvinylchloride, open dump, very wet infiltration class (300mm) (GLO)  1.2% treatment of waste polyvinylchloride, open dump, very wet infiltration class (1000mm) (GLO) | Refer to Item 1 |
| 45 | Polystyrene Collected by Informal Waste Collectors | 0.03 |  | kg | 70% plastic, recycled  24% treatment of waste polystyrene, sanitary landfill (RoW)  4.8% treatment of waste polystyrene, open dump, very wet infiltration class (300mm) (GLO)  1.2% treatment of waste polystyrene, open dump, very wet infiltration class (1000mm) (GLO) | Refer to Item 1 |
| 46 | Not specified Plastics Collected by Informal Waste Collectors | 0 |  | kg | 70% plastic, recycled  24% treatment of waste plastic, mixture, sanitary landfill (RoW)  4.8% treatment of waste plastic, mixture, open dump, very wet infiltration class (300mm) (GLO)  1.2% treatment of waste plastic mixture, open dump, very wet infiltration class (1000mm) (GLO) | Refer to Item 1 |
| 47 | Polyethylene Other End-of-Life | 0 |  | kg | 31% Polyethylene Collected by Formal Waste Collectors  8% Polyethylene Collected by Formal Waste Collectors  61% Uncollected Waste (59% treatment of waste polyethylene, open burning (GLO)  32.8% treatment of waste polyethylene, open dump, very wet infiltration class (300mm) (GLO)  8.2% treatment of polyethylene, open dump, very wet infiltration class (1000mm) (GLO)) | Refer to items 35 &41 |
| 48 | Polypropylene Other End-of-Life | 0 |  | kg | 31% Polypropylene Collected by Formal Waste Collectors  8% Polypropylene Collected by Formal Waste Collectors  61% Uncollected Waste (59% treatment of waste polypropylene, open burning (GLO)  32.8% treatment of waste polypropylene, open dump, very wet infiltration class (300mm) (GLO)  8.2% treatment of polypropylene, open dump, very wet infiltration class (1000mm) (GLO)) | Refer to items 36 &42 |
| 49 | Polyethylene Terephthalate Other End-of-Life | 0 |  | kg | 31% Polyethylene Terephthalate Collected by Formal Waste Collectors  8% Polyethylene Terephthalate Collected by Formal Waste Collectors  61% Uncollected Waste (59% treatment of waste polyethylene terephthalate, open burning (GLO)  32.8% treatment of waste polyethylene terephthalate, open dump, very wet infiltration class (300mm) (GLO)  8.2% treatment of polyethylene terephthalate, open dump, very wet infiltration class (1000mm) (GLO)) | Refer to items 37 &43 |
| 50 | Polyvinylchloride Other End-of-Life | 0 |  | kg | 31% Polyvinylchloride Collected by Formal Waste Collectors  8% Polyvinylchloride Collected by Formal Waste Collectors  61% Uncollected Waste (59% treatment of waste polyvinylchloride, open burning (GLO)  32.8% treatment of waste polyvinylchloride, open dump, very wet infiltration class (300mm) (GLO)  8.2% treatment of polyvinylchloride, open dump, very wet infiltration class (1000mm) (GLO)) | Refer to items 38 &44 |
| 51 | Polystyrene Other End-of-Life | 0 |  | kg | 31% Polystyrene Collected by Formal Waste Collectors  8% Polystyrene Collected by Formal Waste Collectors  61% Uncollected Waste (59% treatment of waste polystyrene, open burning (GLO)  32.8% treatment of waste polystyrene, open dump, very wet infiltration class (300mm) (GLO)  8.2% treatment of polystyrene, open dump, very wet infiltration class (1000mm) (GLO)) | Refer to items 39 &45 |
| 52 | Not specified Plastics Other End-of-Life | 0 |  | kg | 31% Not specified Plastic Collected by Formal Waste Collectors  8% Not specified Plastic Collected by Formal Waste Collectors  61% Uncollected Waste (59% treatment of waste plastic, mixture, open burning (GLO)  32.8% treatment of waste plastic, mixture, open dump, very wet infiltration class (300mm) (GLO)  8.2% treatment of plastic, mixture, open dump, very wet infiltration class (1000mm) (GLO)) | Refer to items 40 &46 |
| 53 | Transport with scooter | 1 |  | person kilometer | Transport, passenger, motor scooter |  |
| 54 | Transport with car | 0.031 |  | km | Transport, passenger car, medium size, diesel, EURO 4 |  |
| 55 | Transport with truck | 0.04 |  | ton km | Transport, freight, lorry, 3.5-7.5 metric ton, EURO 4 |  |
|  | Avoided polyethylene production | -0.41 |  | kg | Polyethylene production, high density, granulate (RoW) |  |
|  | Avoided Polypropylene | -0.16 |  | kg | Polypropylene production, granulate (RoW) |  |
|  | Avoided polyethylene terephthalate | -0.32 |  | kg | Polyethylene product, granulate (RoW) |  |
|  | Avoided polyvinylchloride | -0.0169 |  | kg | Polyvinylchloride production, bulk polymerization (RoW) |  |
|  | Avoided polystyrene production | -0.0424 |  | kg | Polystyrene foam slab production (RoW) |  |

Table S29: Informal Waste Pickers

| No. | Description | Input | Output | Unit | Activity(ies) Name in Ecoinvent v3.10 Database | Comment |
| --- | --- | --- | --- | --- | --- | --- |
| 1 | Recycling of Plastic | 0.00944 |  | kg | -0.01 kg of plastic flake, consumer electronics, unsorted  0.1915kWh market for electricity, low voltage (ID)  0.00778 kg market for liquefied petroleum gas (RoW)  1.01 kg of plastic flake, consumer for recycling (GLO) | From Neo et al 2021 |
| 2 | Incineration of Polyethylene | 0 |  | kg | treatment of waste polyethylene, municipal incineration (GLO) |  |
| 3 | Incineration of Polypropylene | 0 |  | kg | treatment of waste polypropylene, municipal incineration (GLO) |  |
| 4 | Incineration of Polyethylene Terephthalate | 0 |  | kg | treatment of waste polyethylene terephthalate, municipal incineration (GLO) |  |
| 5 | Incineration of Polyvinylchloride | 0 |  | kg | treatment of waste polyvinylchloride, municipal incineration (GLO) |  |
| 6 | Incineration of Polystyrene | 0 |  | kg | treatment of waste polystyrene, municipal incineration (GLO) |  |
| 7 | Incineration of Not specified Plastic | 0 |  | kg | treatment of waste plastic, mixture, municipal incineration (GLO) |  |
| 8 | Landfilling of Polyethylene | 0 |  | kg | treatment of waste polyethylene, sanitary landfill (RoW) |  |
| 9 | Landfilling of Polypropylene | 0 |  | kg | treatment of waste polypropylene, sanitary landfill (RoW) |  |
| 10 | Landfilling of Polyethylene Terephthalate | 0 |  | kg | treatment of waste polyethylene terephthalate, sanitary landfill (RoW) |  |
| 11 | Landfilling of Polyvinylchloride | 0 |  | kg | treatment of waste polyvinylchloride, sanitary landfill (RoW) |  |
| 12 | Landfilling of Polystyrene | 0 |  | kg | treatment of waste polystyrene, sanitary landfill (RoW) |  |
| 13 | Landfilling of Not specified Plastics | 0 |  | kg | treatment of waste plastic, mixture, sanitary landfill (RoW) |  |
| 14 | Open dumping (on land) of Polyethylene | 0 |  | kg | treatment of waste polyethylene, open dump, moist infiltration class (300mm) (GLO) |  |
| 15 | Open dumping (on land) of Polypropylene | 0 |  | kg | treatment of waste polypropylene, open dump, moist infiltration class (300mm) (GLO) |  |
| 16 | Open dumping (on land) of Polyethylene Terephthalate | 0 |  | kg | treatment of waste polyethylene terephthalate, open dump, moist infiltration class (300mm) (GLO) |  |
| 17 | Open dumping (on land) of Polyvinylchloride | 0 |  | kg | treatment of waste polyvinylchloride, open dump, moist infiltration class (300mm) (GLO) |  |
| 18 | Open dumping (on land) of Polystyrene | 0 |  | kg | treatment of waste polystyrene, open dump, moist infiltration class (300mm) (GLO) |  |
| 19 | Open dumping (on land) of Not specified Plastics | 0 |  | kg | treatment of waste plastic, mixture, open dump, moist infiltration class (300mm) (GLO) |  |
| 20 | Open burning of Polyethylene | -0.00315 |  | kg | treatment of waste polyethylene, open burning (GLO) |  |
| 21 | Open burning of Polypropylene | 0 |  | kg | treatment of waste polypropylene, open burning (GLO) |  |
| 22 | Open burning of Polyethylene Terephthalate | 0 |  | kg | treatment of waste polyethylene terephthalate, open burning (GLO) |  |
| 23 | Open burning of Polyvinylchloride | 0 |  | kg | treatment of waste polyvinylchloride, open burning (GLO) |  |
| 24 | Open burning of Polystyrene | 0 |  | kg | treatment of waste polystyrene, open burning (GLO) |  |
| 25 | Open burning of Not specified Plastics | 0 |  | kg | treatment of waste plastic, mixture, open burning (GLO) |  |
| 26 | Open dump (in water) of Polyethylene | 0 |  | kg | treatment of waste polyethylene, open dump, very wet infiltration class (1000mm) (GLO) |  |
| 30 | Open dump (in water) of Polypropylene | 0 |  | kg | treatment of waste polypropylene, open dump, very wet infiltration class (1000mm) (GLO) |  |
| 31 | Open dump (in water) of Polyethylene Terephthalate | 0 |  | kg | treatment of waste polyethylene terephthalate, open dump, very wet infiltration class (1000mm) (GLO) |  |
| 32 | Open dump (in water) of Polyvinylchloride | 0 |  | kg | treatment of waste polyvinylchloride, open dump, very wet infiltration class (1000mm) (GLO) |  |
| 33 | Open dump (in water) of Polystyrene | 0 |  | kg | treatment of waste polystyrene, open dump, very wet infiltration class (1000mm) (GLO) |  |
| 34 | Open dump (in water) of Not specified Plastics | 0 |  | kg | treatment of waste plastic, mixture, open dump, very wet infiltration class (1000mm) (GLO) |  |
| 35 | Polyethylene Collected by Formal Waste Collectors | 0 |  | kg | 79% treatment of waste polyethylene, sanitary landfill (RoW)  16% treatment of waste polyethylene, open dump, very wet infiltration class (300mm) (GLO)  4% treatment of waste polyethylene, open dump, very wet infiltration class (1000mm) (GLO) |  |
| 36 | Polypropylene Collected by Formal Waste Collectors | 0 |  | kg | 79% treatment of waste polypropylene, sanitary landfill (RoW)  16% treatment of waste polypropylene, open dump, very wet infiltration class (300mm) (GLO)  4% treatment of waste polypropylene, open dump, very wet infiltration class (1000mm) (GLO) |  |
| 37 | Polyethylene Terephthalate Collected by Formal Waste Collectors | 0 |  | kg | 79% treatment of waste polyethylene terephthalate, sanitary landfill (RoW)  16% treatment of waste polyethylene terephthalate, open dump, very wet infiltration class (300mm) (GLO)  4% treatment of waste polyethylene terephthalate, open dump, very wet infiltration class (1000mm) (GLO) |  |
| 38 | Polyvinylchloride Collected by Formal Waste Collectors | 0 |  | kg | 79% treatment of waste polyvinylchloride, sanitary landfill (RoW)  16% treatment of waste polyvinylchloride, open dump, very wet infiltration class (300mm) (GLO)  4% treatment of waste polyvinylchloride, open dump, very wet infiltration class (1000mm) (GLO) |  |
| 39 | Polystyrene Collected by Formal Waste Collectors | 0 |  | kg | 79% treatment of waste polystyrene, sanitary landfill (RoW)  16% treatment of waste polystyrene, open dump, very wet infiltration class (300mm) (GLO)  4% treatment of waste polystyrene, open dump, very wet infiltration class (1000mm) (GLO) |  |
| 40 | Not specified Plastics Collected by Formal Waste Collectors | 0 |  | kg | 79% treatment of waste plastic, mixture, sanitary landfill (RoW)  16% treatment of waste plastic, mixture, open dump, very wet infiltration class (300mm) (GLO)  4% treatment of waste plastic, mixture, open dump, very wet infiltration class (1000mm) (GLO) |  |
| 41 | Polyethylene Collected by Informal Waste Collectors | 0 |  | kg | 70% plastic, recycled  24% treatment of waste polyethylene, sanitary landfill (RoW)  4.8% treatment of waste polyethylene, open dump, very wet infiltration class (300mm) (GLO)  1.2% treatment of waste polyethylene, open dump, very wet infiltration class (1000mm) (GLO) | Refer to Item 1 |
| 42 | Polypropylene Collected by Informal Waste Collectors | 0 |  | kg | 70% plastic, recycled  24% treatment of waste polypropylene, sanitary landfill (RoW)  4.8% treatment of waste polypropylene, open dump, very wet infiltration class (300mm) (GLO)  1.2% treatment of waste polypropylene, open dump, very wet infiltration class (1000mm) (GLO) | Refer to Item 1 |
| 43 | Polyethylene Terephthalate Collected by Informal Waste Collectors | 0 |  | kg | 70% plastic, recycled  24% treatment of waste polyethylene terephthalate, sanitary landfill (RoW)  4.8% treatment of waste polyethylene terephthalate, open dump, very wet infiltration class (300mm) (GLO)  1.2% treatment of waste polyethylene, open dump, very wet infiltration class (1000mm) (GLO) | Refer to Item 1 |
| 44 | Polyvinylchloride Collected by Informal Waste Collectors | 0 |  | kg | 70% plastic, recycled  24% treatment of waste polyvinylchloride, sanitary landfill (RoW)  4.8% treatment of waste polyvinylchloride, open dump, very wet infiltration class (300mm) (GLO)  1.2% treatment of waste polyvinylchloride, open dump, very wet infiltration class (1000mm) (GLO) | Refer to Item 1 |
| 45 | Polystyrene Collected by Informal Waste Collectors | 0 |  | kg | 70% plastic, recycled  24% treatment of waste polystyrene, sanitary landfill (RoW)  4.8% treatment of waste polystyrene, open dump, very wet infiltration class (300mm) (GLO)  1.2% treatment of waste polystyrene, open dump, very wet infiltration class (1000mm) (GLO) | Refer to Item 1 |
| 46 | Not specified Plastics Collected by Informal Waste Collectors | 0 |  | kg | 70% plastic, recycled  24% treatment of waste plastic, mixture, sanitary landfill (RoW)  4.8% treatment of waste plastic, mixture, open dump, very wet infiltration class (300mm) (GLO)  1.2% treatment of waste plastic mixture, open dump, very wet infiltration class (1000mm) (GLO) | Refer to Item 1 |
| 47 | Polyethylene Other End-of-Life | 0.39 |  | kg | 31% Polyethylene Collected by Formal Waste Collectors  8% Polyethylene Collected by Formal Waste Collectors  61% Uncollected Waste (59% treatment of waste polyethylene, open burning (GLO)  32.8% treatment of waste polyethylene, open dump, very wet infiltration class (300mm) (GLO)  8.2% treatment of polyethylene, open dump, very wet infiltration class (1000mm) (GLO)) | Refer to items 35 &41 |
| 48 | Polypropylene Other End-of-Life | 0.17 |  | kg | 31% Polypropylene Collected by Formal Waste Collectors  8% Polypropylene Collected by Formal Waste Collectors  61% Uncollected Waste (59% treatment of waste polypropylene, open burning (GLO)  32.8% treatment of waste polypropylene, open dump, very wet infiltration class (300mm) (GLO)  8.2% treatment of polypropylene, open dump, very wet infiltration class (1000mm) (GLO)) | Refer to items 36 &42 |
| 49 | Polyethylene Terephthalate Other End-of-Life | 0.09691 |  | kg | 31% Polyethylene Terephthalate Collected by Formal Waste Collectors  8% Polyethylene Terephthalate Collected by Formal Waste Collectors  61% Uncollected Waste (59% treatment of waste polyethylene terephthalate, open burning (GLO)  32.8% treatment of waste polyethylene terephthalate, open dump, very wet infiltration class (300mm) (GLO)  8.2% treatment of polyethylene terephthalate, open dump, very wet infiltration class (1000mm) (GLO)) | Refer to items 37 &43 |
| 50 | Polyvinylchloride Other End-of-Life | 0.138 |  | kg | 31% Polyvinylchloride Collected by Formal Waste Collectors  8% Polyvinylchloride Collected by Formal Waste Collectors  61% Uncollected Waste (59% treatment of waste polyvinylchloride, open burning (GLO)  32.8% treatment of waste polyvinylchloride, open dump, very wet infiltration class (300mm) (GLO)  8.2% treatment of polyvinylchloride, open dump, very wet infiltration class (1000mm) (GLO)) | Refer to items 38 &44 |
| 51 | Polystyrene Other End-of-Life | 0.11 |  | kg | 31% Polystyrene Collected by Formal Waste Collectors  8% Polystyrene Collected by Formal Waste Collectors  61% Uncollected Waste (59% treatment of waste polystyrene, open burning (GLO)  32.8% treatment of waste polystyrene, open dump, very wet infiltration class (300mm) (GLO)  8.2% treatment of polystyrene, open dump, very wet infiltration class (1000mm) (GLO)) | Refer to items 39 &45 |
| 52 | Not specified Plastics Other End-of-Life | 0.33 |  | kg | 31% Not specified Plastic Collected by Formal Waste Collectors  8% Not specified Plastic Collected by Formal Waste Collectors  61% Uncollected Waste (59% treatment of waste plastic, mixture, open burning (GLO)  32.8% treatment of waste plastic, mixture, open dump, very wet infiltration class (300mm) (GLO)  8.2% treatment of plastic, mixture, open dump, very wet infiltration class (1000mm) (GLO)) | Refer to items 40 &46 |
| 53 | Transport with scooter | 3.18 |  | Person kilometer |  |  |

Table S30: Landfill

| No. | Description | Input | Output | Unit | Activity(ies) Name in Ecoinvent v3.10 Database | Comment |
| --- | --- | --- | --- | --- | --- | --- |
| 1 | Recycling of Plastic | 0 |  | kg | -0.01 kg of plastic flake, consumer electronics, unsorted  0.1915kWh market for electricity, low voltage (ID)  0.00778 kg market for liquefied petroleum gas (RoW)  1.01 kg of plastic flake, consumer for recycling (GLO) | From Neo et al 2021 |
| 2 | Incineration of Polyethylene | 0 |  | kg | treatment of waste polyethylene, municipal incineration (GLO) |  |
| 3 | Incineration of Polypropylene | 0 |  | kg | treatment of waste polypropylene, municipal incineration (GLO) |  |
| 4 | Incineration of Polyethylene Terephthalate | 0 |  | kg | treatment of waste polyethylene terephthalate, municipal incineration (GLO) |  |
| 5 | Incineration of Polyvinylchloride | 0 |  | kg | treatment of waste polyvinylchloride, municipal incineration (GLO) |  |
| 6 | Incineration of Polystyrene | 0 |  | kg | treatment of waste polystyrene, municipal incineration (GLO) |  |
| 7 | Incineration of Not specified Plastic | 0 |  | kg | treatment of waste plastic, mixture, municipal incineration (GLO) |  |
| 8 | Landfilling of Polyethylene | 0 |  | kg | treatment of waste polyethylene, sanitary landfill (RoW) |  |
| 9 | Landfilling of Polypropylene | 0 |  | kg | treatment of waste polypropylene, sanitary landfill (RoW) |  |
| 10 | Landfilling of Polyethylene Terephthalate | 0 |  | kg | treatment of waste polyethylene terephthalate, sanitary landfill (RoW) |  |
| 11 | Landfilling of Polyvinylchloride | 0 |  | kg | treatment of waste polyvinylchloride, sanitary landfill (RoW) |  |
| 12 | Landfilling of Polystyrene | 0 |  | kg | treatment of waste polystyrene, sanitary landfill (RoW) |  |
| 13 | Landfilling of Not specified Plastics | 0 |  | kg | treatment of waste plastic, mixture, sanitary landfill (RoW) |  |
| 14 | Open dumping (on land) of Polyethylene | 0 |  | kg | treatment of waste polyethylene, open dump, moist infiltration class (300mm) (GLO) |  |
| 15 | Open dumping (on land) of Polypropylene | 0 |  | kg | treatment of waste polypropylene, open dump, moist infiltration class (300mm) (GLO) |  |
| 16 | Open dumping (on land) of Polyethylene Terephthalate | 0 |  | kg | treatment of waste polyethylene terephthalate, open dump, moist infiltration class (300mm) (GLO) |  |
| 17 | Open dumping (on land) of Polyvinylchloride | 0 |  | kg | treatment of waste polyvinylchloride, open dump, moist infiltration class (300mm) (GLO) |  |
| 18 | Open dumping (on land) of Polystyrene | 0 |  | kg | treatment of waste polystyrene, open dump, moist infiltration class (300mm) (GLO) |  |
| 19 | Open dumping (on land) of Not specified Plastics | -1 |  | kg | treatment of waste plastic, mixture, open dump, moist infiltration class (300mm) (GLO) |  |
| 20 | Open burning of Polyethylene | 0 |  | kg | treatment of waste polyethylene, open burning (GLO) |  |
| 21 | Open burning of Polypropylene | 0 |  | kg | treatment of waste polypropylene, open burning (GLO) |  |
| 22 | Open burning of Polyethylene Terephthalate | 0 |  | kg | treatment of waste polyethylene terephthalate, open burning (GLO) |  |
| 23 | Open burning of Polyvinylchloride | 0 |  | kg | treatment of waste polyvinylchloride, open burning (GLO) |  |
| 24 | Open burning of Polystyrene | 0 |  | kg | treatment of waste polystyrene, open burning (GLO) |  |
| 25 | Open burning of Not specified Plastics |  |  | kg | treatment of waste plastic, mixture, open burning (GLO) |  |
| 26 | Open dump (in water) of Polyethylene | 0 |  | kg | treatment of waste polyethylene, open dump, very wet infiltration class (1000mm) (GLO) |  |
| 30 | Open dump (in water) of Polypropylene | 0 |  | kg | treatment of waste polypropylene, open dump, very wet infiltration class (1000mm) (GLO) |  |
| 31 | Open dump (in water) of Polyethylene Terephthalate | 0 |  | kg | treatment of waste polyethylene terephthalate, open dump, very wet infiltration class (1000mm) (GLO) |  |
| 32 | Open dump (in water) of Polyvinylchloride | 0 |  | kg | treatment of waste polyvinylchloride, open dump, very wet infiltration class (1000mm) (GLO) |  |
| 33 | Open dump (in water) of Polystyrene | 0 |  | kg | treatment of waste polystyrene, open dump, very wet infiltration class (1000mm) (GLO) |  |
| 34 | Open dump (in water) of Not specified Plastics | 0 |  | kg | treatment of waste plastic, mixture, open dump, very wet infiltration class (1000mm) (GLO) |  |
| 35 | Polyethylene Collected by Formal Waste Collectors | 0 |  | kg | 79% treatment of waste polyethylene, sanitary landfill (RoW)  16% treatment of waste polyethylene, open dump, very wet infiltration class (300mm) (GLO)  4% treatment of waste polyethylene, open dump, very wet infiltration class (1000mm) (GLO) |  |
| 36 | Polypropylene Collected by Formal Waste Collectors | 0 |  | kg | 79% treatment of waste polypropylene, sanitary landfill (RoW)  16% treatment of waste polypropylene, open dump, very wet infiltration class (300mm) (GLO)  4% treatment of waste polypropylene, open dump, very wet infiltration class (1000mm) (GLO) |  |
| 37 | Polyethylene Terephthalate Collected by Formal Waste Collectors | 0 |  | kg | 79% treatment of waste polyethylene terephthalate, sanitary landfill (RoW)  16% treatment of waste polyethylene terephthalate, open dump, very wet infiltration class (300mm) (GLO)  4% treatment of waste polyethylene terephthalate, open dump, very wet infiltration class (1000mm) (GLO) |  |
| 38 | Polyvinylchloride Collected by Formal Waste Collectors | 0 |  | kg | 79% treatment of waste polyvinylchloride, sanitary landfill (RoW)  16% treatment of waste polyvinylchloride, open dump, very wet infiltration class (300mm) (GLO)  4% treatment of waste polyvinylchloride, open dump, very wet infiltration class (1000mm) (GLO) |  |
| 39 | Polystyrene Collected by Formal Waste Collectors | 0 |  | kg | 79% treatment of waste polystyrene, sanitary landfill (RoW)  16% treatment of waste polystyrene, open dump, very wet infiltration class (300mm) (GLO)  4% treatment of waste polystyrene, open dump, very wet infiltration class (1000mm) (GLO) |  |
| 40 | Not specified Plastics Collected by Formal Waste Collectors | 0 |  | kg | 79% treatment of waste plastic, mixture, sanitary landfill (RoW)  16% treatment of waste plastic, mixture, open dump, very wet infiltration class (300mm) (GLO)  4% treatment of waste plastic, mixture, open dump, very wet infiltration class (1000mm) (GLO) |  |
| 41 | Polyethylene Collected by Informal Waste Collectors | 0 |  | kg | 70% plastic, recycled  24% treatment of waste polyethylene, sanitary landfill (RoW)  4.8% treatment of waste polyethylene, open dump, very wet infiltration class (300mm) (GLO)  1.2% treatment of waste polyethylene, open dump, very wet infiltration class (1000mm) (GLO) | Refer to Item 1 |
| 42 | Polypropylene Collected by Informal Waste Collectors | 0 |  | kg | 70% plastic, recycled  24% treatment of waste polypropylene, sanitary landfill (RoW)  4.8% treatment of waste polypropylene, open dump, very wet infiltration class (300mm) (GLO)  1.2% treatment of waste polypropylene, open dump, very wet infiltration class (1000mm) (GLO) | Refer to Item 1 |
| 43 | Polyethylene Terephthalate Collected by Informal Waste Collectors | 0 |  | kg | 70% plastic, recycled  24% treatment of waste polyethylene terephthalate, sanitary landfill (RoW)  4.8% treatment of waste polyethylene terephthalate, open dump, very wet infiltration class (300mm) (GLO)  1.2% treatment of waste polyethylene, open dump, very wet infiltration class (1000mm) (GLO) | Refer to Item 1 |
| 44 | Polyvinylchloride Collected by Informal Waste Collectors | 0 |  | kg | 70% plastic, recycled  24% treatment of waste polyvinylchloride, sanitary landfill (RoW)  4.8% treatment of waste polyvinylchloride, open dump, very wet infiltration class (300mm) (GLO)  1.2% treatment of waste polyvinylchloride, open dump, very wet infiltration class (1000mm) (GLO) | Refer to Item 1 |
| 45 | Polystyrene Collected by Informal Waste Collectors | 0 |  | kg | 70% plastic, recycled  24% treatment of waste polystyrene, sanitary landfill (RoW)  4.8% treatment of waste polystyrene, open dump, very wet infiltration class (300mm) (GLO)  1.2% treatment of waste polystyrene, open dump, very wet infiltration class (1000mm) (GLO) | Refer to Item 1 |
| 46 | Not specified Plastics Collected by Informal Waste Collectors | 0 |  | kg | 70% plastic, recycled  24% treatment of waste plastic, mixture, sanitary landfill (RoW)  4.8% treatment of waste plastic, mixture, open dump, very wet infiltration class (300mm) (GLO)  1.2% treatment of waste plastic mixture, open dump, very wet infiltration class (1000mm) (GLO) | Refer to Item 1 |
| 47 | Polyethylene Other End-of-Life | 0 |  | kg | 31% Polyethylene Collected by Formal Waste Collectors  8% Polyethylene Collected by Formal Waste Collectors  61% Uncollected Waste (59% treatment of waste polyethylene, open burning (GLO)  32.8% treatment of waste polyethylene, open dump, very wet infiltration class (300mm) (GLO)  8.2% treatment of polyethylene, open dump, very wet infiltration class (1000mm) (GLO)) | Refer to items 35 &41 |
| 48 | Polypropylene Other End-of-Life | 0 |  | kg | 31% Polypropylene Collected by Formal Waste Collectors  8% Polypropylene Collected by Formal Waste Collectors  61% Uncollected Waste (59% treatment of waste polypropylene, open burning (GLO)  32.8% treatment of waste polypropylene, open dump, very wet infiltration class (300mm) (GLO)  8.2% treatment of polypropylene, open dump, very wet infiltration class (1000mm) (GLO)) | Refer to items 36 &42 |
| 49 | Polyethylene Terephthalate Other End-of-Life | 0 |  | kg | 31% Polyethylene Terephthalate Collected by Formal Waste Collectors  8% Polyethylene Terephthalate Collected by Formal Waste Collectors  61% Uncollected Waste (59% treatment of waste polyethylene terephthalate, open burning (GLO)  32.8% treatment of waste polyethylene terephthalate, open dump, very wet infiltration class (300mm) (GLO)  8.2% treatment of polyethylene terephthalate, open dump, very wet infiltration class (1000mm) (GLO)) | Refer to items 37 &43 |
| 50 | Polyvinylchloride Other End-of-Life | 0 |  | kg | 31% Polyvinylchloride Collected by Formal Waste Collectors  8% Polyvinylchloride Collected by Formal Waste Collectors  61% Uncollected Waste (59% treatment of waste polyvinylchloride, open burning (GLO)  32.8% treatment of waste polyvinylchloride, open dump, very wet infiltration class (300mm) (GLO)  8.2% treatment of polyvinylchloride, open dump, very wet infiltration class (1000mm) (GLO)) | Refer to items 38 &44 |
| 51 | Polystyrene Other End-of-Life | 0 |  | kg | 31% Polystyrene Collected by Formal Waste Collectors  8% Polystyrene Collected by Formal Waste Collectors  61% Uncollected Waste (59% treatment of waste polystyrene, open burning (GLO)  32.8% treatment of waste polystyrene, open dump, very wet infiltration class (300mm) (GLO)  8.2% treatment of polystyrene, open dump, very wet infiltration class (1000mm) (GLO)) | Refer to items 39 &45 |
| 52 | Not specified Plastics Other End-of-Life | 0 |  | kg | 31% Not specified Plastic Collected by Formal Waste Collectors  8% Not specified Plastic Collected by Formal Waste Collectors  61% Uncollected Waste (59% treatment of waste plastic, mixture, open burning (GLO)  32.8% treatment of waste plastic, mixture, open dump, very wet infiltration class (300mm) (GLO)  8.2% treatment of plastic, mixture, open dump, very wet infiltration class (1000mm) (GLO)) | Refer to items 40 &46 |

Table S31: Local Shop

| No. | Description | Input | Output | Unit | Activity(ies) Name in Ecoinvent v3.10 Database | Comment |
| --- | --- | --- | --- | --- | --- | --- |
| 1 | Recycling of Plastic | 0 |  | kg | -0.01 kg of plastic flake, consumer electronics, unsorted  0.1915kWh market for electricity, low voltage (ID)  0.00778 kg market for liquefied petroleum gas (RoW)  1.01 kg of plastic flake, consumer for recycling (GLO) | From Neo et al 2021 |
| 2 | Incineration of Polyethylene | 0 |  | kg | treatment of waste polyethylene, municipal incineration (GLO) |  |
| 3 | Incineration of Polypropylene | 0 |  | kg | treatment of waste polypropylene, municipal incineration (GLO) |  |
| 4 | Incineration of Polyethylene Terephthalate | 0 |  | kg | treatment of waste polyethylene terephthalate, municipal incineration (GLO) |  |
| 5 | Incineration of Polyvinylchloride | 0 |  | kg | treatment of waste polyvinylchloride, municipal incineration (GLO) |  |
| 6 | Incineration of Polystyrene | 0 |  | kg | treatment of waste polystyrene, municipal incineration (GLO) |  |
| 7 | Incineration of Not specified Plastic | 0 |  | kg | treatment of waste plastic, mixture, municipal incineration (GLO) |  |
| 8 | Landfilling of Polyethylene | 0 |  | kg | treatment of waste polyethylene, sanitary landfill (RoW) |  |
| 9 | Landfilling of Polypropylene | 0 |  | kg | treatment of waste polypropylene, sanitary landfill (RoW) |  |
| 10 | Landfilling of Polyethylene Terephthalate | 0 |  | kg | treatment of waste polyethylene terephthalate, sanitary landfill (RoW) |  |
| 11 | Landfilling of Polyvinylchloride | 0 |  | kg | treatment of waste polyvinylchloride, sanitary landfill (RoW) |  |
| 12 | Landfilling of Polystyrene | 0 |  | kg | treatment of waste polystyrene, sanitary landfill (RoW) |  |
| 13 | Landfilling of Not specified Plastics | 0 |  | kg | treatment of waste plastic, mixture, sanitary landfill (RoW) |  |
| 14 | Open dumping (on land) of Polyethylene | 0 |  | kg | treatment of waste polyethylene, open dump, moist infiltration class (300mm) (GLO) |  |
| 15 | Open dumping (on land) of Polypropylene | 0 |  | kg | treatment of waste polypropylene, open dump, moist infiltration class (300mm) (GLO) |  |
| 16 | Open dumping (on land) of Polyethylene Terephthalate | 0 |  | kg | treatment of waste polyethylene terephthalate, open dump, moist infiltration class (300mm) (GLO) |  |
| 17 | Open dumping (on land) of Polyvinylchloride | 0 |  | kg | treatment of waste polyvinylchloride, open dump, moist infiltration class (300mm) (GLO) |  |
| 18 | Open dumping (on land) of Polystyrene | 0 |  | kg | treatment of waste polystyrene, open dump, moist infiltration class (300mm) (GLO) |  |
| 19 | Open dumping (on land) of Not specified Plastics | 0 |  | kg | treatment of waste plastic, mixture, open dump, moist infiltration class (300mm) (GLO) |  |
| 20 | Open burning of Polyethylene | -0.3543 |  | kg | treatment of waste polyethylene, open burning (GLO) |  |
| 21 | Open burning of Polypropylene | 0 |  | kg | treatment of waste polypropylene, open burning (GLO) |  |
| 22 | Open burning of Polyethylene Terephthalate | -0.1225 |  | kg | treatment of waste polyethylene terephthalate, open burning (GLO) |  |
| 23 | Open burning of Polyvinylchloride | -0.011 |  | kg | treatment of waste polyvinylchloride, open burning (GLO) |  |
| 24 | Open burning of Polystyrene | -0.073 |  | kg | treatment of waste polystyrene, open burning (GLO) |  |
| 25 | Open burning of Not specified Plastics | -0.01398 |  | kg | treatment of waste plastic, mixture, open burning (GLO) |  |
| 26 | Open dump (in water) of Polyethylene | -0.051 |  | kg | treatment of waste polyethylene, open dump, very wet infiltration class (1000mm) (GLO) |  |
| 30 | Open dump (in water) of Polypropylene | 0 |  | kg | treatment of waste polypropylene, open dump, very wet infiltration class (1000mm) (GLO) |  |
| 31 | Open dump (in water) of Polyethylene Terephthalate | -0.0175 |  | kg | treatment of waste polyethylene terephthalate, open dump, very wet infiltration class (1000mm) (GLO) |  |
| 32 | Open dump (in water) of Polyvinylchloride | -0.00158 |  | kg | treatment of waste polyvinylchloride, open dump, very wet infiltration class (1000mm) (GLO) |  |
| 33 | Open dump (in water) of Polystyrene | -0.002544 |  | kg | treatment of waste polystyrene, open dump, very wet infiltration class (1000mm) (GLO) |  |
| 34 | Open dump (in water) of Not specified Plastics | -0.0833 |  | kg | treatment of waste plastic, mixture, open dump, very wet infiltration class (1000mm) (GLO) |  |
| 35 | Polyethylene Collected by Formal Waste Collectors | 0 |  | kg | 79% treatment of waste polyethylene, sanitary landfill (RoW)  16% treatment of waste polyethylene, open dump, very wet infiltration class (300mm) (GLO)  4% treatment of waste polyethylene, open dump, very wet infiltration class (1000mm) (GLO) |  |
| 36 | Polypropylene Collected by Formal Waste Collectors | 0 |  | kg | 79% treatment of waste polypropylene, sanitary landfill (RoW)  16% treatment of waste polypropylene, open dump, very wet infiltration class (300mm) (GLO)  4% treatment of waste polypropylene, open dump, very wet infiltration class (1000mm) (GLO) |  |
| 37 | Polyethylene Terephthalate Collected by Formal Waste Collectors | 0 |  | kg | 79% treatment of waste polyethylene terephthalate, sanitary landfill (RoW)  16% treatment of waste polyethylene terephthalate, open dump, very wet infiltration class (300mm) (GLO)  4% treatment of waste polyethylene terephthalate, open dump, very wet infiltration class (1000mm) (GLO) |  |
| 38 | Polyvinylchloride Collected by Formal Waste Collectors | 0 |  | kg | 79% treatment of waste polyvinylchloride, sanitary landfill (RoW)  16% treatment of waste polyvinylchloride, open dump, very wet infiltration class (300mm) (GLO)  4% treatment of waste polyvinylchloride, open dump, very wet infiltration class (1000mm) (GLO) |  |
| 39 | Polystyrene Collected by Formal Waste Collectors | 0 |  | kg | 79% treatment of waste polystyrene, sanitary landfill (RoW)  16% treatment of waste polystyrene, open dump, very wet infiltration class (300mm) (GLO)  4% treatment of waste polystyrene, open dump, very wet infiltration class (1000mm) (GLO) |  |
| 40 | Not specified Plastics Collected by Formal Waste Collectors | 0 |  | kg | 79% treatment of waste plastic, mixture, sanitary landfill (RoW)  16% treatment of waste plastic, mixture, open dump, very wet infiltration class (300mm) (GLO)  4% treatment of waste plastic, mixture, open dump, very wet infiltration class (1000mm) (GLO) |  |
| 41 | Polyethylene Collected by Informal Waste Collectors | 0 |  | kg | 70% plastic, recycled  24% treatment of waste polyethylene, sanitary landfill (RoW)  4.8% treatment of waste polyethylene, open dump, very wet infiltration class (300mm) (GLO)  1.2% treatment of waste polyethylene, open dump, very wet infiltration class (1000mm) (GLO) | Refer to Item 1 |
| 42 | Polypropylene Collected by Informal Waste Collectors | 0 |  | kg | 70% plastic, recycled  24% treatment of waste polypropylene, sanitary landfill (RoW)  4.8% treatment of waste polypropylene, open dump, very wet infiltration class (300mm) (GLO)  1.2% treatment of waste polypropylene, open dump, very wet infiltration class (1000mm) (GLO) | Refer to Item 1 |
| 43 | Polyethylene Terephthalate Collected by Informal Waste Collectors | 0 |  | kg | 70% plastic, recycled  24% treatment of waste polyethylene terephthalate, sanitary landfill (RoW)  4.8% treatment of waste polyethylene terephthalate, open dump, very wet infiltration class (300mm) (GLO)  1.2% treatment of waste polyethylene, open dump, very wet infiltration class (1000mm) (GLO) | Refer to Item 1 |
| 44 | Polyvinylchloride Collected by Informal Waste Collectors | 0 |  | kg | 70% plastic, recycled  24% treatment of waste polyvinylchloride, sanitary landfill (RoW)  4.8% treatment of waste polyvinylchloride, open dump, very wet infiltration class (300mm) (GLO)  1.2% treatment of waste polyvinylchloride, open dump, very wet infiltration class (1000mm) (GLO) | Refer to Item 1 |
| 45 | Polystyrene Collected by Informal Waste Collectors | 0 |  | kg | 70% plastic, recycled  24% treatment of waste polystyrene, sanitary landfill (RoW)  4.8% treatment of waste polystyrene, open dump, very wet infiltration class (300mm) (GLO)  1.2% treatment of waste polystyrene, open dump, very wet infiltration class (1000mm) (GLO) | Refer to Item 1 |
| 46 | Not specified Plastics Collected by Informal Waste Collectors | 0 |  | kg | 70% plastic, recycled  24% treatment of waste plastic, mixture, sanitary landfill (RoW)  4.8% treatment of waste plastic, mixture, open dump, very wet infiltration class (300mm) (GLO)  1.2% treatment of waste plastic mixture, open dump, very wet infiltration class (1000mm) (GLO) | Refer to Item 1 |
| 47 | Polyethylene Other End-of-Life | 0.2022 |  | kg | 31% Polyethylene Collected by Formal Waste Collectors  8% Polyethylene Collected by Formal Waste Collectors  61% Uncollected Waste (59% treatment of waste polyethylene, open burning (GLO)  32.8% treatment of waste polyethylene, open dump, very wet infiltration class (300mm) (GLO)  8.2% treatment of polyethylene, open dump, very wet infiltration class (1000mm) (GLO)) | Refer to items 35 &41 |
| 48 | Polypropylene Other End-of-Life | 0 |  | kg | 31% Polypropylene Collected by Formal Waste Collectors  8% Polypropylene Collected by Formal Waste Collectors  61% Uncollected Waste (59% treatment of waste polypropylene, open burning (GLO)  32.8% treatment of waste polypropylene, open dump, very wet infiltration class (300mm) (GLO)  8.2% treatment of polypropylene, open dump, very wet infiltration class (1000mm) (GLO)) | Refer to items 36 &42 |
| 49 | Polyethylene Terephthalate Other End-of-Life | 0.07 |  | kg | 31% Polyethylene Terephthalate Collected by Formal Waste Collectors  8% Polyethylene Terephthalate Collected by Formal Waste Collectors  61% Uncollected Waste (59% treatment of waste polyethylene terephthalate, open burning (GLO)  32.8% treatment of waste polyethylene terephthalate, open dump, very wet infiltration class (300mm) (GLO)  8.2% treatment of polyethylene terephthalate, open dump, very wet infiltration class (1000mm) (GLO)) | Refer to items 37 &43 |
| 50 | Polyvinylchloride Other End-of-Life | 0.0063 |  | kg | 31% Polyvinylchloride Collected by Formal Waste Collectors  8% Polyvinylchloride Collected by Formal Waste Collectors  61% Uncollected Waste (59% treatment of waste polyvinylchloride, open burning (GLO)  32.8% treatment of waste polyvinylchloride, open dump, very wet infiltration class (300mm) (GLO)  8.2% treatment of polyvinylchloride, open dump, very wet infiltration class (1000mm) (GLO)) | Refer to items 38 &44 |
| 51 | Polystyrene Other End-of-Life | 0.416 |  | kg | 31% Polystyrene Collected by Formal Waste Collectors  8% Polystyrene Collected by Formal Waste Collectors  61% Uncollected Waste (59% treatment of waste polystyrene, open burning (GLO)  32.8% treatment of waste polystyrene, open dump, very wet infiltration class (300mm) (GLO)  8.2% treatment of polystyrene, open dump, very wet infiltration class (1000mm) (GLO)) | Refer to items 39 &45 |
| 52 | Not specified Plastics Other End-of-Life | 0.33 |  | kg | 31% Not specified Plastic Collected by Formal Waste Collectors  8% Not specified Plastic Collected by Formal Waste Collectors  61% Uncollected Waste (59% treatment of waste plastic, mixture, open burning (GLO)  32.8% treatment of waste plastic, mixture, open dump, very wet infiltration class (300mm) (GLO)  8.2% treatment of plastic, mixture, open dump, very wet infiltration class (1000mm) (GLO)) | Refer to items 40 &46 |

Table S32: Local Market

| No. | Description | Input | Output | Unit | Activity(ies) Name in Ecoinvent v3.10 Database | Comment |
| --- | --- | --- | --- | --- | --- | --- |
| 1 | Recycling of Plastic | 0 |  | kg | -0.01 kg of plastic flake, consumer electronics, unsorted  0.1915kWh market for electricity, low voltage (ID)  0.00778 kg market for liquefied petroleum gas (RoW)  1.01 kg of plastic flake, consumer for recycling (GLO) | From Neo et al 2021 |
| 2 | Incineration of Polyethylene | 0 |  | kg | treatment of waste polyethylene, municipal incineration (GLO) |  |
| 3 | Incineration of Polypropylene | 0 |  | kg | treatment of waste polypropylene, municipal incineration (GLO) |  |
| 4 | Incineration of Polyethylene Terephthalate | 0 |  | kg | treatment of waste polyethylene terephthalate, municipal incineration (GLO) |  |
| 5 | Incineration of Polyvinylchloride | 0 |  | kg | treatment of waste polyvinylchloride, municipal incineration (GLO) |  |
| 6 | Incineration of Polystyrene | 0 |  | kg | treatment of waste polystyrene, municipal incineration (GLO) |  |
| 7 | Incineration of Not specified Plastic | 0 |  | kg | treatment of waste plastic, mixture, municipal incineration (GLO) |  |
| 8 | Landfilling of Polyethylene | 0 |  | kg | treatment of waste polyethylene, sanitary landfill (RoW) |  |
| 9 | Landfilling of Polypropylene | 0 |  | kg | treatment of waste polypropylene, sanitary landfill (RoW) |  |
| 10 | Landfilling of Polyethylene Terephthalate | 0 |  | kg | treatment of waste polyethylene terephthalate, sanitary landfill (RoW) |  |
| 11 | Landfilling of Polyvinylchloride | 0 |  | kg | treatment of waste polyvinylchloride, sanitary landfill (RoW) |  |
| 12 | Landfilling of Polystyrene | 0 |  | kg | treatment of waste polystyrene, sanitary landfill (RoW) |  |
| 13 | Landfilling of Not specified Plastics | 0 |  | kg | treatment of waste plastic, mixture, sanitary landfill (RoW) |  |
| 14 | Open dumping (on land) of Polyethylene | 0 |  | kg | treatment of waste polyethylene, open dump, moist infiltration class (300mm) (GLO) |  |
| 15 | Open dumping (on land) of Polypropylene | 0 |  | kg | treatment of waste polypropylene, open dump, moist infiltration class (300mm) (GLO) |  |
| 16 | Open dumping (on land) of Polyethylene Terephthalate | 0 |  | kg | treatment of waste polyethylene terephthalate, open dump, moist infiltration class (300mm) (GLO) |  |
| 17 | Open dumping (on land) of Polyvinylchloride | 0 |  | kg | treatment of waste polyvinylchloride, open dump, moist infiltration class (300mm) (GLO) |  |
| 18 | Open dumping (on land) of Polystyrene | 0 |  | kg | treatment of waste polystyrene, open dump, moist infiltration class (300mm) (GLO) |  |
| 19 | Open dumping (on land) of Not specified Plastics | 0 |  | kg | treatment of waste plastic, mixture, open dump, moist infiltration class (300mm) (GLO) |  |
| 20 | Open burning of Polyethylene | 0 |  | kg | treatment of waste polyethylene, open burning (GLO) |  |
| 21 | Open burning of Polypropylene | 0 |  | kg | treatment of waste polypropylene, open burning (GLO) |  |
| 22 | Open burning of Polyethylene Terephthalate | 0 |  | kg | treatment of waste polyethylene terephthalate, open burning (GLO) |  |
| 23 | Open burning of Polyvinylchloride | 0 |  | kg | treatment of waste polyvinylchloride, open burning (GLO) |  |
| 24 | Open burning of Polystyrene | 0 |  | kg | treatment of waste polystyrene, open burning (GLO) |  |
| 25 | Open burning of Not specified Plastics | 0 |  | kg | treatment of waste plastic, mixture, open burning (GLO) |  |
| 26 | Open dump (in water) of Polyethylene | 0 |  | kg | treatment of waste polyethylene, open dump, very wet infiltration class (1000mm) (GLO) |  |
| 30 | Open dump (in water) of Polypropylene | 0 |  | kg | treatment of waste polypropylene, open dump, very wet infiltration class (1000mm) (GLO) |  |
| 31 | Open dump (in water) of Polyethylene Terephthalate | 0 |  | kg | treatment of waste polyethylene terephthalate, open dump, very wet infiltration class (1000mm) (GLO) |  |
| 32 | Open dump (in water) of Polyvinylchloride | 0 |  | kg | treatment of waste polyvinylchloride, open dump, very wet infiltration class (1000mm) (GLO) |  |
| 33 | Open dump (in water) of Polystyrene | 0 |  | kg | treatment of waste polystyrene, open dump, very wet infiltration class (1000mm) (GLO) |  |
| 34 | Open dump (in water) of Not specified Plastics | 0.7 |  | kg | treatment of waste plastic, mixture, open dump, very wet infiltration class (1000mm) (GLO) |  |
| 35 | Polyethylene Collected by Formal Waste Collectors | 0 |  | kg | 79% treatment of waste polyethylene, sanitary landfill (RoW)  16% treatment of waste polyethylene, open dump, very wet infiltration class (300mm) (GLO)  4% treatment of waste polyethylene, open dump, very wet infiltration class (1000mm) (GLO) |  |
| 36 | Polypropylene Collected by Formal Waste Collectors | 0 |  | kg | 79% treatment of waste polypropylene, sanitary landfill (RoW)  16% treatment of waste polypropylene, open dump, very wet infiltration class (300mm) (GLO)  4% treatment of waste polypropylene, open dump, very wet infiltration class (1000mm) (GLO) |  |
| 37 | Polyethylene Terephthalate Collected by Formal Waste Collectors | 0 |  | kg | 79% treatment of waste polyethylene terephthalate, sanitary landfill (RoW)  16% treatment of waste polyethylene terephthalate, open dump, very wet infiltration class (300mm) (GLO)  4% treatment of waste polyethylene terephthalate, open dump, very wet infiltration class (1000mm) (GLO) |  |
| 38 | Polyvinylchloride Collected by Formal Waste Collectors | 0 |  | kg | 79% treatment of waste polyvinylchloride, sanitary landfill (RoW)  16% treatment of waste polyvinylchloride, open dump, very wet infiltration class (300mm) (GLO)  4% treatment of waste polyvinylchloride, open dump, very wet infiltration class (1000mm) (GLO) |  |
| 39 | Polystyrene Collected by Formal Waste Collectors | 0 |  | kg | 79% treatment of waste polystyrene, sanitary landfill (RoW)  16% treatment of waste polystyrene, open dump, very wet infiltration class (300mm) (GLO)  4% treatment of waste polystyrene, open dump, very wet infiltration class (1000mm) (GLO) |  |
| 40 | Not specified Plastics Collected by Formal Waste Collectors | 0 |  | kg | 79% treatment of waste plastic, mixture, sanitary landfill (RoW)  16% treatment of waste plastic, mixture, open dump, very wet infiltration class (300mm) (GLO)  4% treatment of waste plastic, mixture, open dump, very wet infiltration class (1000mm) (GLO) |  |
| 41 | Polyethylene Collected by Informal Waste Collectors | 0.3 |  | kg | 70% plastic, recycled  24% treatment of waste polyethylene, sanitary landfill (RoW)  4.8% treatment of waste polyethylene, open dump, very wet infiltration class (300mm) (GLO)  1.2% treatment of waste polyethylene, open dump, very wet infiltration class (1000mm) (GLO) | Refer to Item 1 |
| 42 | Polypropylene Collected by Informal Waste Collectors | 0 |  | kg | 70% plastic, recycled  24% treatment of waste polypropylene, sanitary landfill (RoW)  4.8% treatment of waste polypropylene, open dump, very wet infiltration class (300mm) (GLO)  1.2% treatment of waste polypropylene, open dump, very wet infiltration class (1000mm) (GLO) | Refer to Item 1 |
| 43 | Polyethylene Terephthalate Collected by Informal Waste Collectors | 0 |  | kg | 70% plastic, recycled  24% treatment of waste polyethylene terephthalate, sanitary landfill (RoW)  4.8% treatment of waste polyethylene terephthalate, open dump, very wet infiltration class (300mm) (GLO)  1.2% treatment of waste polyethylene, open dump, very wet infiltration class (1000mm) (GLO) | Refer to Item 1 |
| 44 | Polyvinylchloride Collected by Informal Waste Collectors | 0 |  | kg | 70% plastic, recycled  24% treatment of waste polyvinylchloride, sanitary landfill (RoW)  4.8% treatment of waste polyvinylchloride, open dump, very wet infiltration class (300mm) (GLO)  1.2% treatment of waste polyvinylchloride, open dump, very wet infiltration class (1000mm) (GLO) | Refer to Item 1 |
| 45 | Polystyrene Collected by Informal Waste Collectors | 0 |  | kg | 70% plastic, recycled  24% treatment of waste polystyrene, sanitary landfill (RoW)  4.8% treatment of waste polystyrene, open dump, very wet infiltration class (300mm) (GLO)  1.2% treatment of waste polystyrene, open dump, very wet infiltration class (1000mm) (GLO) | Refer to Item 1 |
| 46 | Not specified Plastics Collected by Informal Waste Collectors | 0 |  | kg | 70% plastic, recycled  24% treatment of waste plastic, mixture, sanitary landfill (RoW)  4.8% treatment of waste plastic, mixture, open dump, very wet infiltration class (300mm) (GLO)  1.2% treatment of waste plastic mixture, open dump, very wet infiltration class (1000mm) (GLO) | Refer to Item 1 |
| 47 | Polyethylene Other End-of-Life | 0 |  | kg | 31% Polyethylene Collected by Formal Waste Collectors  8% Polyethylene Collected by Formal Waste Collectors  61% Uncollected Waste (59% treatment of waste polyethylene, open burning (GLO)  32.8% treatment of waste polyethylene, open dump, very wet infiltration class (300mm) (GLO)  8.2% treatment of polyethylene, open dump, very wet infiltration class (1000mm) (GLO)) | Refer to items 35 &41 |
| 48 | Polypropylene Other End-of-Life | 0 |  | kg | 31% Polypropylene Collected by Formal Waste Collectors  8% Polypropylene Collected by Formal Waste Collectors  61% Uncollected Waste (59% treatment of waste polypropylene, open burning (GLO)  32.8% treatment of waste polypropylene, open dump, very wet infiltration class (300mm) (GLO)  8.2% treatment of polypropylene, open dump, very wet infiltration class (1000mm) (GLO)) | Refer to items 36 &42 |
| 49 | Polyethylene Terephthalate Other End-of-Life | 0 |  | kg | 31% Polyethylene Terephthalate Collected by Formal Waste Collectors  8% Polyethylene Terephthalate Collected by Formal Waste Collectors  61% Uncollected Waste (59% treatment of waste polyethylene terephthalate, open burning (GLO)  32.8% treatment of waste polyethylene terephthalate, open dump, very wet infiltration class (300mm) (GLO)  8.2% treatment of polyethylene terephthalate, open dump, very wet infiltration class (1000mm) (GLO)) | Refer to items 37 &43 |
| 50 | Polyvinylchloride Other End-of-Life | 0 |  | kg | 31% Polyvinylchloride Collected by Formal Waste Collectors  8% Polyvinylchloride Collected by Formal Waste Collectors  61% Uncollected Waste (59% treatment of waste polyvinylchloride, open burning (GLO)  32.8% treatment of waste polyvinylchloride, open dump, very wet infiltration class (300mm) (GLO)  8.2% treatment of polyvinylchloride, open dump, very wet infiltration class (1000mm) (GLO)) | Refer to items 38 &44 |
| 51 | Polystyrene Other End-of-Life | 0 |  | kg | 31% Polystyrene Collected by Formal Waste Collectors  8% Polystyrene Collected by Formal Waste Collectors  61% Uncollected Waste (59% treatment of waste polystyrene, open burning (GLO)  32.8% treatment of waste polystyrene, open dump, very wet infiltration class (300mm) (GLO)  8.2% treatment of polystyrene, open dump, very wet infiltration class (1000mm) (GLO)) | Refer to items 39 &45 |
| 52 | Not specified Plastics Other End-of-Life | 0 |  | kg | 31% Not specified Plastic Collected by Formal Waste Collectors  8% Not specified Plastic Collected by Formal Waste Collectors  61% Uncollected Waste (59% treatment of waste plastic, mixture, open burning (GLO)  32.8% treatment of waste plastic, mixture, open dump, very wet infiltration class (300mm) (GLO)  8.2% treatment of plastic, mixture, open dump, very wet infiltration class (1000mm) (GLO)) | Refer to items 40 &46 |

Table S33: Public Facilities

| No. | Description | Input | Output | Unit | Activity(ies) Name in Ecoinvent v3.10 Database | Comment |
| --- | --- | --- | --- | --- | --- | --- |
| 1 | Recycling of Plastic | 0 |  | kg | -0.01 kg of plastic flake, consumer electronics, unsorted  0.1915kWh market for electricity, low voltage (ID)  0.00778 kg market for liquefied petroleum gas (RoW)  1.01 kg of plastic flake, consumer for recycling (GLO) | From Neo et al 2021 |
| 2 | Incineration of Polyethylene | 0 |  | kg | treatment of waste polyethylene, municipal incineration (GLO) |  |
| 3 | Incineration of Polypropylene | 0 |  | kg | treatment of waste polypropylene, municipal incineration (GLO) |  |
| 4 | Incineration of Polyethylene Terephthalate | 0 |  | kg | treatment of waste polyethylene terephthalate, municipal incineration (GLO) |  |
| 5 | Incineration of Polyvinylchloride | 0 |  | kg | treatment of waste polyvinylchloride, municipal incineration (GLO) |  |
| 6 | Incineration of Polystyrene | 0 |  | kg | treatment of waste polystyrene, municipal incineration (GLO) |  |
| 7 | Incineration of Not specified Plastic | 0 |  | kg | treatment of waste plastic, mixture, municipal incineration (GLO) |  |
| 8 | Landfilling of Polyethylene | 0 |  | kg | treatment of waste polyethylene, sanitary landfill (RoW) |  |
| 9 | Landfilling of Polypropylene | 0 |  | kg | treatment of waste polypropylene, sanitary landfill (RoW) |  |
| 10 | Landfilling of Polyethylene Terephthalate | 0 |  | kg | treatment of waste polyethylene terephthalate, sanitary landfill (RoW) |  |
| 11 | Landfilling of Polyvinylchloride | 0 |  | kg | treatment of waste polyvinylchloride, sanitary landfill (RoW) |  |
| 12 | Landfilling of Polystyrene | 0 |  | kg | treatment of waste polystyrene, sanitary landfill (RoW) |  |
| 13 | Landfilling of Not specified Plastics | 0 |  | kg | treatment of waste plastic, mixture, sanitary landfill (RoW) |  |
| 14 | Open dumping (on land) of Polyethylene | 0 |  | kg | treatment of waste polyethylene, open dump, moist infiltration class (300mm) (GLO) |  |
| 15 | Open dumping (on land) of Polypropylene | 0 |  | kg | treatment of waste polypropylene, open dump, moist infiltration class (300mm) (GLO) |  |
| 16 | Open dumping (on land) of Polyethylene Terephthalate | 0 |  | kg | treatment of waste polyethylene terephthalate, open dump, moist infiltration class (300mm) (GLO) |  |
| 17 | Open dumping (on land) of Polyvinylchloride | 0 |  | kg | treatment of waste polyvinylchloride, open dump, moist infiltration class (300mm) (GLO) |  |
| 18 | Open dumping (on land) of Polystyrene | 0 |  | kg | treatment of waste polystyrene, open dump, moist infiltration class (300mm) (GLO) |  |
| 19 | Open dumping (on land) of Not specified Plastics | 0 |  | kg | treatment of waste plastic, mixture, open dump, moist infiltration class (300mm) (GLO) |  |
| 20 | Open burning of Polyethylene | -0.203 |  | kg | treatment of waste polyethylene, open burning (GLO) |  |
| 21 | Open burning of Polypropylene | -0.0194 |  | kg | treatment of waste polypropylene, open burning (GLO) |  |
| 22 | Open burning of Polyethylene Terephthalate | -0.1573 |  | kg | treatment of waste polyethylene terephthalate, open burning (GLO) |  |
| 23 | Open burning of Polyvinylchloride | 0 |  | kg | treatment of waste polyvinylchloride, open burning (GLO) |  |
| 24 | Open burning of Polystyrene | -0.01731 |  | kg | treatment of waste polystyrene, open burning (GLO) |  |
| 25 | Open burning of Not specified Plastics | 0 |  | kg | treatment of waste plastic, mixture, open burning (GLO) |  |
| 26 | Open dump (in water) of Polyethylene | -0.0254 |  | kg | treatment of waste polyethylene, open dump, very wet infiltration class (1000mm) (GLO) |  |
| 30 | Open dump (in water) of Polypropylene | -0.0024 |  | kg | treatment of waste polypropylene, open dump, very wet infiltration class (1000mm) (GLO) |  |
| 31 | Open dump (in water) of Polyethylene Terephthalate | -0.197 |  | kg | treatment of waste polyethylene terephthalate, open dump, very wet infiltration class (1000mm) (GLO) |  |
| 32 | Open dump (in water) of Polyvinylchloride | 0 |  | kg | treatment of waste polyvinylchloride, open dump, very wet infiltration class (1000mm) (GLO) |  |
| 33 | Open dump (in water) of Polystyrene | -0.0025 |  | kg | treatment of waste polystyrene, open dump, very wet infiltration class (1000mm) (GLO) |  |
| 34 | Open dump (in water) of Not specified Plastics | 0 |  | kg | treatment of waste plastic, mixture, open dump, very wet infiltration class (1000mm) (GLO) |  |
| 35 | Polyethylene Collected by Formal Waste Collectors | 0.127 |  | kg | 79% treatment of waste polyethylene, sanitary landfill (RoW)  16% treatment of waste polyethylene, open dump, very wet infiltration class (300mm) (GLO)  4% treatment of waste polyethylene, open dump, very wet infiltration class (1000mm) (GLO) |  |
| 36 | Polypropylene Collected by Formal Waste Collectors | 0.121 |  | kg | 79% treatment of waste polypropylene, sanitary landfill (RoW)  16% treatment of waste polypropylene, open dump, very wet infiltration class (300mm) (GLO)  0.01214% treatment of waste polypropylene, open dump, very w0.0983et infiltration class (1000mm) (GLO) |  |
| 37 | Polyethylene Terephthalate Collected by Formal Waste Collectors | 0.0121 |  | kg | 79% treatment of waste polyethylene terephthalate, sanitary landfill (RoW)  16% treatment of waste polyethylene terephthalate, open dump, very wet infiltration class (300mm) (GLO)  4% treatment of waste polyethylene terephthalate, open dump, very wet infiltration class (1000mm) (GLO) |  |
| 38 | Polyvinylchloride Collected by Formal Waste Collectors | 0.0983 |  | kg | 79% treatment of waste polyvinylchloride, sanitary landfill (RoW)  16% treatment of waste polyvinylchloride, open dump, very wet infiltration class (300mm) (GLO)  4% treatment of waste polyvinylchloride, open dump, very wet infiltration class (1000mm) (GLO) |  |
| 39 | Polystyrene Collected by Formal Waste Collectors | 0.0126 |  | kg | 79% treatment of waste polystyrene, sanitary landfill (RoW)  16% treatment of waste polystyrene, open dump, very wet infiltration class (300mm) (GLO)  4% treatment of waste polystyrene, open dump, very wet infiltration class (1000mm) (GLO) |  |
| 40 | Not specified Plastics Collected by Formal Waste Collectors | 0 |  | kg | 79% treatment of waste plastic, mixture, sanitary landfill (RoW)  16% treatment of waste plastic, mixture, open dump, very wet infiltration class (300mm) (GLO)  4% treatment of waste plastic, mixture, open dump, very wet infiltration class (1000mm) (GLO) |  |
| 41 | Polyethylene Collected by Informal Waste Collectors | 0.09737 |  | kg | 70% plastic, recycled  24% treatment of waste polyethylene, sanitary landfill (RoW)  4.8% treatment of waste polyethylene, open dump, very wet infiltration class (300mm) (GLO)  1.2% treatment of waste polyethylene, open dump, very wet infiltration class (1000mm) (GLO) | Refer to Item 1 |
| 42 | Polypropylene Collected by Informal Waste Collectors | 0.0954 |  | kg | 70% plastic, recycled  24% treatment of waste polypropylene, sanitary landfill (RoW)  4.8% treatment of waste polypropylene, open dump, very wet infiltration class (300mm) (GLO)  1.2% treatment of waste polypropylene, open dump, very wet infiltration class (1000mm) (GLO) | Refer to Item 1 |
| 43 | Polyethylene Terephthalate Collected by Informal Waste Collectors | 0.0093 |  | kg | 70% plastic, recycled  24% treatment of waste polyethylene terephthalate, sanitary landfill (RoW)  4.8% treatment of waste polyethylene terephthalate, open dump, very wet infiltration class (300mm) (GLO)  1.2% treatment of waste polyethylene, open dump, very wet infiltration class (1000mm) (GLO) | Refer to Item 1 |
| 44 | Polyvinylchloride Collected by Informal Waste Collectors | 0.0754 |  | kg | 70% plastic, recycled  24% treatment of waste polyvinylchloride, sanitary landfill (RoW)  4.8% treatment of waste polyvinylchloride, open dump, very wet infiltration class (300mm) (GLO)  1.2% treatment of waste polyvinylchloride, open dump, very wet infiltration class (1000mm) (GLO) | Refer to Item 1 |
| 45 | Polystyrene Collected by Informal Waste Collectors | 0 |  | kg | 70% plastic, recycled  24% treatment of waste polystyrene, sanitary landfill (RoW)  4.8% treatment of waste polystyrene, open dump, very wet infiltration class (300mm) (GLO)  1.2% treatment of waste polystyrene, open dump, very wet infiltration class (1000mm) (GLO) | Refer to Item 1 |
| 46 | Not specified Plastics Collected by Informal Waste Collectors | 0.0097 |  | kg | 70% plastic, recycled  24% treatment of waste plastic, mixture, sanitary landfill (RoW)  4.8% treatment of waste plastic, mixture, open dump, very wet infiltration class (300mm) (GLO)  1.2% treatment of waste plastic mixture, open dump, very wet infiltration class (1000mm) (GLO) | Refer to Item 1 |
| 47 | Polyethylene Other End-of-Life | 0.055 |  | kg | 31% Polyethylene Collected by Formal Waste Collectors  8% Polyethylene Collected by Formal Waste Collectors  61% Uncollected Waste (59% treatment of waste polyethylene, open burning (GLO)  32.8% treatment of waste polyethylene, open dump, very wet infiltration class (300mm) (GLO)  8.2% treatment of polyethylene, open dump, very wet infiltration class (1000mm) (GLO)) | Refer to items 35 &41 |
| 48 | Polypropylene Other End-of-Life | 0.0052 |  | kg | 31% Polypropylene Collected by Formal Waste Collectors  8% Polypropylene Collected by Formal Waste Collectors  61% Uncollected Waste (59% treatment of waste polypropylene, open burning (GLO)  32.8% treatment of waste polypropylene, open dump, very wet infiltration class (300mm) (GLO)  8.2% treatment of polypropylene, open dump, very wet infiltration class (1000mm) (GLO)) | Refer to items 36 &42 |
| 49 | Polyethylene Terephthalate Other End-of-Life | 0.0426 |  | kg | 31% Polyethylene Terephthalate Collected by Formal Waste Collectors  8% Polyethylene Terephthalate Collected by Formal Waste Collectors  61% Uncollected Waste (59% treatment of waste polyethylene terephthalate, open burning (GLO)  32.8% treatment of waste polyethylene terephthalate, open dump, very wet infiltration class (300mm) (GLO)  8.2% treatment of polyethylene terephthalate, open dump, very wet infiltration class (1000mm) (GLO)) | Refer to items 37 &43 |
| 50 | Polyvinylchloride Other End-of-Life | 0 |  | kg | 31% Polyvinylchloride Collected by Formal Waste Collectors  8% Polyvinylchloride Collected by Formal Waste Collectors  61% Uncollected Waste (59% treatment of waste polyvinylchloride, open burning (GLO)  32.8% treatment of waste polyvinylchloride, open dump, very wet infiltration class (300mm) (GLO)  8.2% treatment of polyvinylchloride, open dump, very wet infiltration class (1000mm) (GLO)) | Refer to items 38 &44 |
| 51 | Polystyrene Other End-of-Life | 0.0054 |  | kg | 31% Polystyrene Collected by Formal Waste Collectors  8% Polystyrene Collected by Formal Waste Collectors  61% Uncollected Waste (59% treatment of waste polystyrene, open burning (GLO)  32.8% treatment of waste polystyrene, open dump, very wet infiltration class (300mm) (GLO)  8.2% treatment of polystyrene, open dump, very wet infiltration class (1000mm) (GLO)) | Refer to items 39 &45 |
| 52 | Not specified Plastics Other End-of-Life | 0 |  | kg | 31% Not specified Plastic Collected by Formal Waste Collectors  8% Not specified Plastic Collected by Formal Waste Collectors  61% Uncollected Waste (59% treatment of waste plastic, mixture, open burning (GLO)  32.8% treatment of waste plastic, mixture, open dump, very wet infiltration class (300mm) (GLO)  8.2% treatment of plastic, mixture, open dump, very wet infiltration class (1000mm) (GLO)) | Refer to items 40 &46 |

Table S34: Public Offices

| No. | Description | Input | Output | Unit | Activity(ies) Name in Ecoinvent v3.10 Database | Comment |
| --- | --- | --- | --- | --- | --- | --- |
| 1 | Recycling of Plastic | 0 |  | kg | -0.01 kg of plastic flake, consumer electronics, unsorted  0.1915kWh market for electricity, low voltage (ID)  0.00778 kg market for liquefied petroleum gas (RoW)  1.01 kg of plastic flake, consumer for recycling (GLO) | From Neo et al 2021 |
| 2 | Incineration of Polyethylene | 0 |  | kg | treatment of waste polyethylene, municipal incineration (GLO) |  |
| 3 | Incineration of Polypropylene | 0 |  | kg | treatment of waste polypropylene, municipal incineration (GLO) |  |
| 4 | Incineration of Polyethylene Terephthalate | 0 |  | kg | treatment of waste polyethylene terephthalate, municipal incineration (GLO) |  |
| 5 | Incineration of Polyvinylchloride | 0 |  | kg | treatment of waste polyvinylchloride, municipal incineration (GLO) |  |
| 6 | Incineration of Polystyrene | 0 |  | kg | treatment of waste polystyrene, municipal incineration (GLO) |  |
| 7 | Incineration of Not specified Plastic | 0 |  | kg | treatment of waste plastic, mixture, municipal incineration (GLO) |  |
| 8 | Landfilling of Polyethylene | 0 |  | kg | treatment of waste polyethylene, sanitary landfill (RoW) |  |
| 9 | Landfilling of Polypropylene | 0 |  | kg | treatment of waste polypropylene, sanitary landfill (RoW) |  |
| 10 | Landfilling of Polyethylene Terephthalate | 0 |  | kg | treatment of waste polyethylene terephthalate, sanitary landfill (RoW) |  |
| 11 | Landfilling of Polyvinylchloride | 0 |  | kg | treatment of waste polyvinylchloride, sanitary landfill (RoW) |  |
| 12 | Landfilling of Polystyrene | 0 |  | kg | treatment of waste polystyrene, sanitary landfill (RoW) |  |
| 13 | Landfilling of Not specified Plastics | 0 |  | kg | treatment of waste plastic, mixture, sanitary landfill (RoW) |  |
| 14 | Open dumping (on land) of Polyethylene | 0 |  | kg | treatment of waste polyethylene, open dump, moist infiltration class (300mm) (GLO) |  |
| 15 | Open dumping (on land) of Polypropylene | 0 |  | kg | treatment of waste polypropylene, open dump, moist infiltration class (300mm) (GLO) |  |
| 16 | Open dumping (on land) of Polyethylene Terephthalate | 0 |  | kg | treatment of waste polyethylene terephthalate, open dump, moist infiltration class (300mm) (GLO) |  |
| 17 | Open dumping (on land) of Polyvinylchloride | 0 |  | kg | treatment of waste polyvinylchloride, open dump, moist infiltration class (300mm) (GLO) |  |
| 18 | Open dumping (on land) of Polystyrene | 0 |  | kg | treatment of waste polystyrene, open dump, moist infiltration class (300mm) (GLO) |  |
| 19 | Open dumping (on land) of Not specified Plastics | 0 |  | kg | treatment of waste plastic, mixture, open dump, moist infiltration class (300mm) (GLO) |  |
| 20 | Open burning of Polyethylene | -0.111 |  | kg | treatment of waste polyethylene, open burning (GLO) |  |
| 21 | Open burning of Polypropylene | -0.0609 |  | kg | treatment of waste polypropylene, open burning (GLO) |  |
| 22 | Open burning of Polyethylene Terephthalate | -0.0528 |  | kg | treatment of waste polyethylene terephthalate, open burning (GLO) |  |
| 23 | Open burning of Polyvinylchloride | 0 |  | kg | treatment of waste polyvinylchloride, open burning (GLO) |  |
| 24 | Open burning of Polystyrene | -0.0058 |  | kg | treatment of waste polystyrene, open burning (GLO) |  |
| 25 | Open burning of Not specified Plastics | 0 |  | kg | treatment of waste plastic, mixture, open burning (GLO) |  |
| 26 | Open dump (in water) of Polyethylene | -0.0481 |  | kg | treatment of waste polyethylene, open dump, very wet infiltration class (1000mm) (GLO) |  |
| 30 | Open dump (in water) of Polypropylene | -0.0265 |  | kg | treatment of waste polypropylene, open dump, very wet infiltration class (1000mm) (GLO) |  |
| 31 | Open dump (in water) of Polyethylene Terephthalate | -0.0229 |  | kg | treatment of waste polyethylene terephthalate, open dump, very wet infiltration class (1000mm) (GLO) |  |
| 32 | Open dump (in water) of Polyvinylchloride | 0 |  | kg | treatment of waste polyvinylchloride, open dump, very wet infiltration class (1000mm) (GLO) |  |
| 33 | Open dump (in water) of Polystyrene | -0.0025 |  | kg | treatment of waste polystyrene, open dump, very wet infiltration class (1000mm) (GLO) |  |
| 34 | Open dump (in water) of Not specified Plastics | 0 |  | kg | treatment of waste plastic, mixture, open dump, very wet infiltration class (1000mm) (GLO) |  |
| 35 | Polyethylene Collected by Formal Waste Collectors | 0.125 |  | kg | 79% treatment of waste polyethylene, sanitary landfill (RoW)  16% treatment of waste polyethylene, open dump, very wet infiltration class (300mm) (GLO)  4% treatment of waste polyethylene, open dump, very wet infiltration class (1000mm) (GLO) |  |
| 36 | Polypropylene Collected by Formal Waste Collectors | 0.0688 |  | kg | 79% treatment of waste polypropylene, sanitary landfill (RoW)  16% treatment of waste polypropylene, open dump, very wet infiltration class (300mm) (GLO)  4% treatment of waste polypropylene, open dump, very wet infiltration class (1000mm) (GLO) |  |
| 37 | Polyethylene Terephthalate Collected by Formal Waste Collectors | 0.0597 |  | kg | 79% treatment of waste polyethylene terephthalate, sanitary landfill (RoW)  16% treatment of waste polyethylene terephthalate, open dump, very wet infiltration class (300mm) (GLO)  4% treatment of waste polyethylene terephthalate, open dump, very wet infiltration class (1000mm) (GLO) |  |
| 38 | Polyvinylchloride Collected by Formal Waste Collectors | 0 |  | kg | 79% treatment of waste polyvinylchloride, sanitary landfill (RoW)  16% treatment of waste polyvinylchloride, open dump, very wet infiltration class (300mm) (GLO)  4% treatment of waste polyvinylchloride, open dump, very wet infiltration class (1000mm) (GLO) |  |
| 39 | Polystyrene Collected by Formal Waste Collectors | 0.0065 |  | kg | 79% treatment of waste polystyrene, sanitary landfill (RoW)  16% treatment of waste polystyrene, open dump, very wet infiltration class (300mm) (GLO)  4% treatment of waste polystyrene, open dump, very wet infiltration class (1000mm) (GLO) |  |
| 40 | Not specified Plastics Collected by Formal Waste Collectors | 0 |  | kg | 79% treatment of waste plastic, mixture, sanitary landfill (RoW)  16% treatment of waste plastic, mixture, open dump, very wet infiltration class (300mm) (GLO)  4% treatment of waste plastic, mixture, open dump, very wet infiltration class (1000mm) (GLO) |  |
| 41 | Polyethylene Collected by Informal Waste Collectors | 0.1298 |  | kg | 70% plastic, recycled  24% treatment of waste polyethylene, sanitary landfill (RoW)  4.8% treatment of waste polyethylene, open dump, very wet infiltration class (300mm) (GLO)  1.2% treatment of waste polyethylene, open dump, very wet infiltration class (1000mm) (GLO) | Refer to Item 1 |
| 42 | Polypropylene Collected by Informal Waste Collectors | 0.0715 |  | kg | 70% plastic, recycled  24% treatment of waste polypropylene, sanitary landfill (RoW)  4.8% treatment of waste polypropylene, open dump, very wet infiltration class (300mm) (GLO)  1.2% treatment of waste polypropylene, open dump, very wet infiltration class (1000mm) (GLO) | Refer to Item 1 |
| 43 | Polyethylene Terephthalate Collected by Informal Waste Collectors | 0.062 |  | kg | 70% plastic, recycled  24% treatment of waste polyethylene terephthalate, sanitary landfill (RoW)  4.8% treatment of waste polyethylene terephthalate, open dump, very wet infiltration class (300mm) (GLO)  1.2% treatment of waste polyethylene, open dump, very wet infiltration class (1000mm) (GLO) | Refer to Item 1 |
| 44 | Polyvinylchloride Collected by Informal Waste Collectors | 0 |  | kg | 70% plastic, recycled  24% treatment of waste polyvinylchloride, sanitary landfill (RoW)  4.8% treatment of waste polyvinylchloride, open dump, very wet infiltration class (300mm) (GLO)  1.2% treatment of waste polyvinylchloride, open dump, very wet infiltration class (1000mm) (GLO) | Refer to Item 1 |
| 45 | Polystyrene Collected by Informal Waste Collectors | 0.0068 |  | kg | 70% plastic, recycled  24% treatment of waste polystyrene, sanitary landfill (RoW)  4.8% treatment of waste polystyrene, open dump, very wet infiltration class (300mm) (GLO)  1.2% treatment of waste polystyrene, open dump, very wet infiltration class (1000mm) (GLO) | Refer to Item 1 |
| 46 | Not specified Plastics Collected by Informal Waste Collectors | 0 |  | kg | 70% plastic, recycled  24% treatment of waste plastic, mixture, sanitary landfill (RoW)  4.8% treatment of waste plastic, mixture, open dump, very wet infiltration class (300mm) (GLO)  1.2% treatment of waste plastic mixture, open dump, very wet infiltration class (1000mm) (GLO) | Refer to Item 1 |
| 47 | Polyethylene Other End-of-Life | 0.0673 |  | kg | 31% Polyethylene Collected by Formal Waste Collectors  8% Polyethylene Collected by Formal Waste Collectors  61% Uncollected Waste (59% treatment of waste polyethylene, open burning (GLO)  32.8% treatment of waste polyethylene, open dump, very wet infiltration class (300mm) (GLO)  8.2% treatment of polyethylene, open dump, very wet infiltration class (1000mm) (GLO)) | Refer to items 35 &41 |
| 48 | Polypropylene Other End-of-Life | 0.0371 |  | kg | 31% Polypropylene Collected by Formal Waste Collectors  8% Polypropylene Collected by Formal Waste Collectors  61% Uncollected Waste (59% treatment of waste polypropylene, open burning (GLO)  32.8% treatment of waste polypropylene, open dump, very wet infiltration class (300mm) (GLO)  8.2% treatment of polypropylene, open dump, very wet infiltration class (1000mm) (GLO)) | Refer to items 36 &42 |
| 49 | Polyethylene Terephthalate Other End-of-Life | 0.0321 |  | kg | 31% Polyethylene Terephthalate Collected by Formal Waste Collectors  8% Polyethylene Terephthalate Collected by Formal Waste Collectors  61% Uncollected Waste (59% treatment of waste polyethylene terephthalate, open burning (GLO)  32.8% treatment of waste polyethylene terephthalate, open dump, very wet infiltration class (300mm) (GLO)  8.2% treatment of polyethylene terephthalate, open dump, very wet infiltration class (1000mm) (GLO)) | Refer to items 37 &43 |
| 50 | Polyvinylchloride Other End-of-Life | 0 |  | kg | 31% Polyvinylchloride Collected by Formal Waste Collectors  8% Polyvinylchloride Collected by Formal Waste Collectors  61% Uncollected Waste (59% treatment of waste polyvinylchloride, open burning (GLO)  32.8% treatment of waste polyvinylchloride, open dump, very wet infiltration class (300mm) (GLO)  8.2% treatment of polyvinylchloride, open dump, very wet infiltration class (1000mm) (GLO)) | Refer to items 38 &44 |
| 51 | Polystyrene Other End-of-Life | 0.0035 |  | kg | 31% Polystyrene Collected by Formal Waste Collectors  8% Polystyrene Collected by Formal Waste Collectors  61% Uncollected Waste (59% treatment of waste polystyrene, open burning (GLO)  32.8% treatment of waste polystyrene, open dump, very wet infiltration class (300mm) (GLO)  8.2% treatment of polystyrene, open dump, very wet infiltration class (1000mm) (GLO)) | Refer to items 39 &45 |
| 52 | Not specified Plastics Other End-of-Life | 0 |  | kg | 31% Not specified Plastic Collected by Formal Waste Collectors  8% Not specified Plastic Collected by Formal Waste Collectors  61% Uncollected Waste (59% treatment of waste plastic, mixture, open burning (GLO)  32.8% treatment of waste plastic, mixture, open dump, very wet infiltration class (300mm) (GLO)  8.2% treatment of plastic, mixture, open dump, very wet infiltration class (1000mm) (GLO)) | Refer to items 40 &46 |

Table S35: Recycling Industry

| No. | Description | Input | Output | Unit | Activity(ies) Name in Ecoinvent v3.10 Database | Comment |
| --- | --- | --- | --- | --- | --- | --- |
| 1 | Recycling of Plastic | 1 |  | kg | -0.01 kg of plastic flake, consumer electronics, unsorted  0.1915kWh market for electricity, low voltage (ID)  0.00778 kg market for liquefied petroleum gas (RoW)  1.01 kg of plastic flake, consumer for recycling (GLO) | From Neo et al 2021 |
| 2 | Incineration of Polyethylene | 0 |  | kg | treatment of waste polyethylene, municipal incineration (GLO) |  |
| 3 | Incineration of Polypropylene | 0 |  | kg | treatment of waste polypropylene, municipal incineration (GLO) |  |
| 4 | Incineration of Polyethylene Terephthalate | 0 |  | kg | treatment of waste polyethylene terephthalate, municipal incineration (GLO) |  |
| 5 | Incineration of Polyvinylchloride | 0 |  | kg | treatment of waste polyvinylchloride, municipal incineration (GLO) |  |
| 6 | Incineration of Polystyrene | 0 |  | kg | treatment of waste polystyrene, municipal incineration (GLO) |  |
| 7 | Incineration of Not specified Plastic | 0 |  | kg | treatment of waste plastic, mixture, municipal incineration (GLO) |  |
| 8 | Landfilling of Polyethylene | 0 |  | kg | treatment of waste polyethylene, sanitary landfill (RoW) |  |
| 9 | Landfilling of Polypropylene | 0 |  | kg | treatment of waste polypropylene, sanitary landfill (RoW) |  |
| 10 | Landfilling of Polyethylene Terephthalate | 0 |  | kg | treatment of waste polyethylene terephthalate, sanitary landfill (RoW) |  |
| 11 | Landfilling of Polyvinylchloride | 0 |  | kg | treatment of waste polyvinylchloride, sanitary landfill (RoW) |  |
| 12 | Landfilling of Polystyrene | 0 |  | kg | treatment of waste polystyrene, sanitary landfill (RoW) |  |
| 13 | Landfilling of Not specified Plastics | 0 |  | kg | treatment of waste plastic, mixture, sanitary landfill (RoW) |  |
| 14 | Open dumping (on land) of Polyethylene | 0 |  | kg | treatment of waste polyethylene, open dump, moist infiltration class (300mm) (GLO) |  |
| 15 | Open dumping (on land) of Polypropylene | 0 |  | kg | treatment of waste polypropylene, open dump, moist infiltration class (300mm) (GLO) |  |
| 16 | Open dumping (on land) of Polyethylene Terephthalate | 0 |  | kg | treatment of waste polyethylene terephthalate, open dump, moist infiltration class (300mm) (GLO) |  |
| 17 | Open dumping (on land) of Polyvinylchloride | 0 |  | kg | treatment of waste polyvinylchloride, open dump, moist infiltration class (300mm) (GLO) |  |
| 18 | Open dumping (on land) of Polystyrene | 0 |  | kg | treatment of waste polystyrene, open dump, moist infiltration class (300mm) (GLO) |  |
| 19 | Open dumping (on land) of Not specified Plastics | 0 |  | kg | treatment of waste plastic, mixture, open dump, moist infiltration class (300mm) (GLO) |  |
| 20 | Open burning of Polyethylene | 0 |  | kg | treatment of waste polyethylene, open burning (GLO) |  |
| 21 | Open burning of Polypropylene | 0 |  | kg | treatment of waste polypropylene, open burning (GLO) |  |
| 22 | Open burning of Polyethylene Terephthalate | 0 |  | kg | treatment of waste polyethylene terephthalate, open burning (GLO) |  |
| 23 | Open burning of Polyvinylchloride | 0 |  | kg | treatment of waste polyvinylchloride, open burning (GLO) |  |
| 24 | Open burning of Polystyrene | 0 |  | kg | treatment of waste polystyrene, open burning (GLO) |  |
| 25 | Open burning of Not specified Plastics | 0 |  | kg | treatment of waste plastic, mixture, open burning (GLO) |  |
| 26 | Open dump (in water) of Polyethylene | 0 |  | kg | treatment of waste polyethylene, open dump, very wet infiltration class (1000mm) (GLO) |  |
| 30 | Open dump (in water) of Polypropylene | 0 |  | kg | treatment of waste polypropylene, open dump, very wet infiltration class (1000mm) (GLO) |  |
| 31 | Open dump (in water) of Polyethylene Terephthalate | 0 |  | kg | treatment of waste polyethylene terephthalate, open dump, very wet infiltration class (1000mm) (GLO) |  |
| 32 | Open dump (in water) of Polyvinylchloride | 0 |  | kg | treatment of waste polyvinylchloride, open dump, very wet infiltration class (1000mm) (GLO) |  |
| 33 | Open dump (in water) of Polystyrene | 0 |  | kg | treatment of waste polystyrene, open dump, very wet infiltration class (1000mm) (GLO) |  |
| 34 | Open dump (in water) of Not specified Plastics | 0 |  | kg | treatment of waste plastic, mixture, open dump, very wet infiltration class (1000mm) (GLO) |  |
| 35 | Polyethylene Collected by Formal Waste Collectors | 0 |  | kg | 79% treatment of waste polyethylene, sanitary landfill (RoW)  16% treatment of waste polyethylene, open dump, very wet infiltration class (300mm) (GLO)  4% treatment of waste polyethylene, open dump, very wet infiltration class (1000mm) (GLO) |  |
| 36 | Polypropylene Collected by Formal Waste Collectors | 0 |  | kg | 79% treatment of waste polypropylene, sanitary landfill (RoW)  16% treatment of waste polypropylene, open dump, very wet infiltration class (300mm) (GLO)  4% treatment of waste polypropylene, open dump, very wet infiltration class (1000mm) (GLO) |  |
| 37 | Polyethylene Terephthalate Collected by Formal Waste Collectors | 0 |  | kg | 79% treatment of waste polyethylene terephthalate, sanitary landfill (RoW)  16% treatment of waste polyethylene terephthalate, open dump, very wet infiltration class (300mm) (GLO)  4% treatment of waste polyethylene terephthalate, open dump, very wet infiltration class (1000mm) (GLO) |  |
| 38 | Polyvinylchloride Collected by Formal Waste Collectors | 0 |  | kg | 79% treatment of waste polyvinylchloride, sanitary landfill (RoW)  16% treatment of waste polyvinylchloride, open dump, very wet infiltration class (300mm) (GLO)  4% treatment of waste polyvinylchloride, open dump, very wet infiltration class (1000mm) (GLO) |  |
| 39 | Polystyrene Collected by Formal Waste Collectors | 0 |  | kg | 79% treatment of waste polystyrene, sanitary landfill (RoW)  16% treatment of waste polystyrene, open dump, very wet infiltration class (300mm) (GLO)  4% treatment of waste polystyrene, open dump, very wet infiltration class (1000mm) (GLO) |  |
| 40 | Not specified Plastics Collected by Formal Waste Collectors | 0 |  | kg | 79% treatment of waste plastic, mixture, sanitary landfill (RoW)  16% treatment of waste plastic, mixture, open dump, very wet infiltration class (300mm) (GLO)  4% treatment of waste plastic, mixture, open dump, very wet infiltration class (1000mm) (GLO) |  |
| 41 | Polyethylene Collected by Informal Waste Collectors | 0 |  | kg | 70% plastic, recycled  24% treatment of waste polyethylene, sanitary landfill (RoW)  4.8% treatment of waste polyethylene, open dump, very wet infiltration class (300mm) (GLO)  1.2% treatment of waste polyethylene, open dump, very wet infiltration class (1000mm) (GLO) | Refer to Item 1 |
| 42 | Polypropylene Collected by Informal Waste Collectors | 0 |  | kg | 70% plastic, recycled  24% treatment of waste polypropylene, sanitary landfill (RoW)  4.8% treatment of waste polypropylene, open dump, very wet infiltration class (300mm) (GLO)  1.2% treatment of waste polypropylene, open dump, very wet infiltration class (1000mm) (GLO) | Refer to Item 1 |
| 43 | Polyethylene Terephthalate Collected by Informal Waste Collectors | 0 |  | kg | 70% plastic, recycled  24% treatment of waste polyethylene terephthalate, sanitary landfill (RoW)  4.8% treatment of waste polyethylene terephthalate, open dump, very wet infiltration class (300mm) (GLO)  1.2% treatment of waste polyethylene, open dump, very wet infiltration class (1000mm) (GLO) | Refer to Item 1 |
| 44 | Polyvinylchloride Collected by Informal Waste Collectors | 0 |  | kg | 70% plastic, recycled  24% treatment of waste polyvinylchloride, sanitary landfill (RoW)  4.8% treatment of waste polyvinylchloride, open dump, very wet infiltration class (300mm) (GLO)  1.2% treatment of waste polyvinylchloride, open dump, very wet infiltration class (1000mm) (GLO) | Refer to Item 1 |
| 45 | Polystyrene Collected by Informal Waste Collectors | 0 |  | kg | 70% plastic, recycled  24% treatment of waste polystyrene, sanitary landfill (RoW)  4.8% treatment of waste polystyrene, open dump, very wet infiltration class (300mm) (GLO)  1.2% treatment of waste polystyrene, open dump, very wet infiltration class (1000mm) (GLO) | Refer to Item 1 |
| 46 | Not specified Plastics Collected by Informal Waste Collectors | 0 |  | kg | 70% plastic, recycled  24% treatment of waste plastic, mixture, sanitary landfill (RoW)  4.8% treatment of waste plastic, mixture, open dump, very wet infiltration class (300mm) (GLO)  1.2% treatment of waste plastic mixture, open dump, very wet infiltration class (1000mm) (GLO) | Refer to Item 1 |
| 47 | Polyethylene Other End-of-Life | 0 |  | kg | 31% Polyethylene Collected by Formal Waste Collectors  8% Polyethylene Collected by Formal Waste Collectors  61% Uncollected Waste (59% treatment of waste polyethylene, open burning (GLO)  32.8% treatment of waste polyethylene, open dump, very wet infiltration class (300mm) (GLO)  8.2% treatment of polyethylene, open dump, very wet infiltration class (1000mm) (GLO)) | Refer to items 35 &41 |
| 48 | Polypropylene Other End-of-Life | 0 |  | kg | 31% Polypropylene Collected by Formal Waste Collectors  8% Polypropylene Collected by Formal Waste Collectors  61% Uncollected Waste (59% treatment of waste polypropylene, open burning (GLO)  32.8% treatment of waste polypropylene, open dump, very wet infiltration class (300mm) (GLO)  8.2% treatment of polypropylene, open dump, very wet infiltration class (1000mm) (GLO)) | Refer to items 36 &42 |
| 49 | Polyethylene Terephthalate Other End-of-Life | 0 |  | kg | 31% Polyethylene Terephthalate Collected by Formal Waste Collectors  8% Polyethylene Terephthalate Collected by Formal Waste Collectors  61% Uncollected Waste (59% treatment of waste polyethylene terephthalate, open burning (GLO)  32.8% treatment of waste polyethylene terephthalate, open dump, very wet infiltration class (300mm) (GLO)  8.2% treatment of polyethylene terephthalate, open dump, very wet infiltration class (1000mm) (GLO)) | Refer to items 37 &43 |
| 50 | Polyvinylchloride Other End-of-Life | 0 |  | kg | 31% Polyvinylchloride Collected by Formal Waste Collectors  8% Polyvinylchloride Collected by Formal Waste Collectors  61% Uncollected Waste (59% treatment of waste polyvinylchloride, open burning (GLO)  32.8% treatment of waste polyvinylchloride, open dump, very wet infiltration class (300mm) (GLO)  8.2% treatment of polyvinylchloride, open dump, very wet infiltration class (1000mm) (GLO)) | Refer to items 38 &44 |
| 51 | Polystyrene Other End-of-Life | 0 |  | kg | 31% Polystyrene Collected by Formal Waste Collectors  8% Polystyrene Collected by Formal Waste Collectors  61% Uncollected Waste (59% treatment of waste polystyrene, open burning (GLO)  32.8% treatment of waste polystyrene, open dump, very wet infiltration class (300mm) (GLO)  8.2% treatment of polystyrene, open dump, very wet infiltration class (1000mm) (GLO)) | Refer to items 39 &45 |
| 52 | Not specified Plastics Other End-of-Life | 0 |  | kg | 31% Not specified Plastic Collected by Formal Waste Collectors  8% Not specified Plastic Collected by Formal Waste Collectors  61% Uncollected Waste (59% treatment of waste plastic, mixture, open burning (GLO)  32.8% treatment of waste plastic, mixture, open dump, very wet infiltration class (300mm) (GLO)  8.2% treatment of plastic, mixture, open dump, very wet infiltration class (1000mm) (GLO)) | Refer to items 40 &46 |
| 53 | Avoided plastic production | -1 |  | kg | Polypropylene production, granulate (RoW) |  |

# 3. Extended Results

Table S36: Net Results for Villages Current Scenario in Midpoint Impact Category ReCiPe v3.10 (H)

| Village | National Average | Bakalan | Bedali | Gampingan | Krebet Senggrong | Krebet | Mangunrejo | Pagak | Rekasan | Sumberejo | Tegalsari | Tlogorejo |
| --- | --- | --- | --- | --- | --- | --- | --- | --- | --- | --- | --- | --- |
| Acidification: terrestrial \| terrestrial acidification potential (TAP) | 7.08E-04 | 5.79E-04 | -1.28E-03 | 8.82E-05 | 2.18E-03 | -2.29E-04 | -1.09E-03 | 6.95E-04 | 4.52E-05 | 8.73E-04 | 7.76E-04 | 3.51E-05 |
| Climate change \| global warming potential (GWP100) | 1.13E+00 | 1.07E+00 | -4.58E-01 | 1.83E-01 | 2.12E+00 | 7.50E-01 | -3.75E-01 | 1.11E+00 | 1.38E-01 | 1.15E+00 | 9.53E-01 | 7.41E-01 |
| Ecotoxicity: freshwater \| freshwater ecotoxicity potential (FETP) | 9.97E-02 | 9.29E-02 | 1.19E-01 | 1.64E-02 | 2.06E-01 | 7.86E-02 | 1.20E-01 | 3.45E-01 | 1.65E-01 | 8.12E-02 | 7.02E-02 | 8.44E-02 |
| Ecotoxicity: marine \| marine ecotoxicity potential (METP) | 1.45E-01 | 1.36E-01 | 1.66E-01 | 2.41E-02 | 2.90E-01 | 1.17E-01 | 1.68E-01 | 4.84E-01 | 2.32E-01 | 1.20E-01 | 1.04E-01 | 1.23E-01 |
| Ecotoxicity: terrestrial \| terrestrial ecotoxicity potential (TETP) | 1.26E+01 | 1.24E+01 | 3.61E-02 | 2.68E+00 | 1.61E+01 | 1.22E+01 | 3.46E-02 | 7.90E+00 | 3.79E-02 | 1.35E+01 | 1.18E+01 | 9.57E+00 |
| Energy resources: non-renewable, fossil \| fossil fuel potential (FFP) | 7.83E-03 | 9.08E-03 | -2.85E-01 | -1.25E-02 | 3.28E-02 | -1.83E-01 | -2.43E-01 | 3.36E-02 | 5.65E-03 | 6.34E-03 | 8.78E-03 | -1.31E-01 |
| Eutrophication: freshwater \| freshwater eutrophication potential (FEP) | 2.79E-05 | 4.01E-05 | 7.72E-05 | 1.06E-05 | 8.13E-05 | 8.12E-06 | 6.62E-05 | 9.87E-05 | 1.18E-06 | 3.25E-05 | 2.86E-05 | 9.91E-06 |
| Eutrophication: marine \| marine eutrophication potential (MEP) | 4.57E-04 | 3.35E-04 | 1.46E-04 | 1.08E-04 | 1.50E-03 | 2.84E-04 | 1.49E-04 | 1.28E-03 | 2.05E-04 | 6.02E-04 | 5.51E-04 | 4.18E-04 |
| Human toxicity: carcinogenic \| human toxicity potential (HTPc) | 1.45E-02 | 1.44E-01 | 2.25E-03 | 5.61E-03 | 1.36E-01 | 4.56E-03 | 2.28E-03 | 2.91E-02 | 3.24E-03 | 5.72E-02 | 2.24E-02 | -2.37E-02 |
| Human toxicity: non-carcinogenic \| human toxicity potential (HTPnc) | 2.11E+00 | 1.93E+00 | 2.35E+00 | 2.90E-01 | 4.78E+00 | 1.52E+00 | 2.41E+00 | 7.63E+00 | 3.32E+00 | 1.51E+00 | 1.25E+00 | 1.81E+00 |
| Ionising radiation \| ionising radiation potential (IRP) | 3.04E-04 | 5.71E-04 | 8.88E-04 | 2.25E-04 | 1.93E-03 | -1.39E-02 | 7.94E-04 | 1.87E-03 | 2.11E-04 | 2.96E-04 | 5.61E-04 | -9.88E-03 |
| Land use \| agricultural land occupation (LOP) | 1.89E-03 | 3.14E-03 | 3.07E-03 | 6.43E-04 | 5.72E-03 | -2.61E-03 | 3.03E-03 | 8.15E-03 | 2.91E-03 | 2.71E-03 | 2.64E-03 | -1.24E-03 |
| Material resources: metals/minerals \| surplus ore potential (SOP) | 1.02E-03 | 2.26E-03 | 2.86E-03 | 5.31E-04 | 5.37E-03 | -1.51E-03 | 2.47E-03 | 7.23E-03 | 2.10E-04 | 1.88E-03 | 1.73E-03 | -4.85E-04 |
| Ozone depletion \| ozone depletion potential (ODPinfinite) | 4.78E-07 | 4.35E-07 | 2.90E-08 | 9.78E-08 | 9.48E-07 | -3.20E-08 | 2.53E-08 | 3.30E-07 | 4.64E-09 | 4.43E-07 | 4.28E-07 | 3.87E-09 |
| Particulate matter formation \| particulate matter formation potential (PMFP) | 1.53E-03 | 7.82E-03 | 5.02E-04 | 2.22E-04 | 9.18E-03 | 3.46E-03 | 4.32E-04 | 1.61E-03 | 2.25E-05 | 3.05E-03 | 8.80E-04 | 9.11E-04 |
| Photochemical oxidant formation: human health \| photochemical oxidant formation potential: humans (HOFP) | 1.54E-03 | 1.18E-03 | -6.91E-04 | 3.26E-04 | 4.71E-03 | 5.49E-04 | -5.83E-04 | 1.16E-03 | 8.10E-05 | 2.09E-03 | 1.84E-03 | 8.60E-04 |
| Photochemical oxidant formation: terrestrial ecosystems \| photochemical oxidant formation potential: ecosystems (EOFP) | 1.54E-03 | 1.19E-03 | -7.39E-04 | 3.25E-04 | 4.73E-03 | 4.46E-04 | -6.23E-04 | 1.18E-03 | 8.47E-05 | 2.09E-03 | 1.85E-03 | 7.85E-04 |
| ReCiPe 2016 v1.03, midpoint (H) \| water use \| water consumption potential (WCP) | 4.69E-04 | 4.87E-04 | -8.52E-03 | -3.53E-04 | 1.13E-03 | -2.08E-03 | -7.29E-03 | 6.53E-04 | 2.84E-05 | 4.31E-04 | 4.09E-04 | -1.24E-03 |
| ReCiPe 2016 v1.03, midpoint (H) \| water use \| water consumption potential (WCP)2 | 4.69E-04 | 4.87E-04 | 2.91E-04 | 1.22E-04 | 1.13E-03 | -2.08E-03 | 2.53E-04 | 6.53E-04 | 2.84E-05 | 5.59E-04 | 4.80E-04 | -1.24E-03 |

## 3.1. Results by Stakeholder Group

The results above aggregate the stakeholders interviewed by the village in which they are located, making the results more digestible and easier to understand. An alternative comparison metric is by stakeholder group, of which the corresponding LCIs and the corresponding results are shown below.

Fig S3: Breakdown of Global Warming Potential by Stakeholder Groups in Malang District.

kg of CO2 Eq

|  | Rest (+) | Rest(-) | Other EoL | Informal Waste Collector | Formal Waste Collector | Open burning | Open dumping (in water) | Open dumping (in land) | Incineration | Recycling | Avoided plastic production | Sanitary Landfill | Transport | Score |
| --- | --- | --- | --- | --- | --- | --- | --- | --- | --- | --- | --- | --- | --- | --- |
| 1kg Formal Waste Collector | 2.22045e-16 | -1.70509e-08 | 1.26929 | 0 | 0 | 0 | 0 | 0 | 0 | 0.0102031 | 0 | 0 | 0.0658475 | 1.34534 |
| 1kg Formal Waste Picker | 1.0934e-08 | 0 | 1.09333 | 0 | 0 | 0.00121413 | 0.000173034 | 0 | 0 | 0 | 0 | 0 | 0.073681 | 1.16839 |
| 1kg Household | 2.16361e-08 | 0 | 0 | 0.0979321 | 0.0167922 | 1.39075 | 0.0411245 | 0 | 0 | 0 | 0 | 0 | 0 | 1.54659 |
| 1kg Recycling Industry | 0 | -7.10543e-15 | 0 | 0 | 0 | 0 | 0 | 0 | 0 | 0.500154 | -3.79433 | 0 | 0 | -3.29417 |
| 1kg Landfill | 0 | 0 | 0 | 0 | 0 | 0 | 0 | 0 | 0 | 0 | 0 | 0.107086 | 0 | 0.107086 |
| 1kg Industry | 1.51916e-08 | 0 | 0.0615707 | 0 | 0 | 0.188736 | 0.00978831 | 0.0234119 | 0.592269 | 0.155048 | 0 | 0.00788548 | 0 | 1.03966 |
| 1kg Informal Waste Collector | 2.78583e-08 | 0 | 0 | 0 | 0 | 0.0812581 | 0 | 0 | 0 | 0.482648 | -2.55303 | 0.107086 | 0.175902 | -1.70613 |
| 1kg Informal Waste Pickers | 5.36657e-06 | 0 | 1.37258 | 0 | 0 | 0.0102222 | 0 | 0 | 0 | 0.00472145 | 0 | 0 | 0.448003 | 1.83553 |
| 1kg Local Shop | 4.44089e-16 | -1.23288e-08 | 1.22788 | 0 | 0 | 1.71599 | 0 | 0.0222453 | 0 | 0 | 0 | 0 | 0 | 2.96612 |
| 1kg Market | 6.54575e-09 | 0 | 0 | 0.207815 | 0.100533 | 0 | 0 | 0 | 0 | 0 | 0 | 0 | 0 | 0.308348 |
| 1kg Public Facilities | 0 | -3.21824e-08 | 0.123819 | 0.196883 | 0.0438812 | 1.11471 | 0 | 0.0275824 | 0 | 0 | 0 | 0 | 0 | 1.50687 |
| 1kg Public Offices | 1.06804e-09 | 0 | 0.163608 | 0.190459 | 0.033095 | 0.66207 | 0 | 0.0152433 | 0 | 0 | 0 | 0 | 0 | 1.06448 |

Fig S4: Breakdown of Primary Energy Depletion Potential by Stakeholder Groups in Malang District.

kg of oil Eq

|  | Rest (+) | Rest(-) | Other EoL | Informal Waste Collector | Formal Waste Collector | Open burning | Incineration | Recycling | Avoided plastic production | Sanitary Landfill | Transport | Score |
| --- | --- | --- | --- | --- | --- | --- | --- | --- | --- | --- | --- | --- |
| 1kg Formal Waste Collector | 1.29142e-10 | 0 | 0.0143733 | 0 | 0 | 0 | 0 | 0.0023091 | 0 | 0 | 0.0195346 | 0.0362483 |
| 1kg Formal Waste Picker | 2.61652e-10 | 0 | 0.0143722 | 0 | 0 | 0 | 0 | 0 | 0 | 0 | 0.0197876 | 0.0342047 |
| 1kg Household | 3.46945e-18 | -5.34923e-10 | 0 | 0.0237346 | 0.000569233 | 0.000569233 | 0 | 0 | 0 | 0 | 0 | 0.0243038 |
| 1kg Recycling Industry | 0 | -3.10862e-15 | 0 | 0 | 0 | 0 | 0 | 0.113191 | -1.94057 | 0 | 0 | -1.82738 |
| 1kg Landfill | 0 | 0 | 0 | 0 | 0 | 0 | 0 | 0 | 0 | 0.00570544 | 0 | 0.00570544 |
| 1kg Industry | 2.5073e-10 | 0 | 0.000735938 | 0 | 0 | 0 | 0.00378396 | 0.0350893 | 0 | 0.000395543 | 0 | 0.0400048 |
| 1kg Informal Waste Collector | 1.56836e-08 | 0 | 0 | 0 | 0 | 0 | 0 | 0.10923 | -1.24982 | 0.00570544 | 0.0484496 | -1.08644 |
| 1kg Informal Waste Pickers | 2.44508e-09 | 0 | 0.01349 | 0 | 0 | 0 | 0 | 0.00106853 | 0 | 0 | 0.120315 | 0.138633 |
| 1kg Local Shop | 1.14688e-10 | 0 | 0.0103644 | 0 | 0 | 0 | 0 | 0 | 0 | 0 | 0 | 0.0141584 |
| 1kg Market | 1.9585e-09 | 0 | 0 | 0.0506258 | 0.00312313 | 0.00312313 | 0 | 0 | 0 | 0 | 0 | 0.053749 |
| 1kg Public Facilities | 0 | -1.05967e-10 | 0.00162379 | 0.0481007 | 0.00162164 | 0.00162164 | 0 | 0 | 0 | 0 | 0 | 0.0513461 |
| 1kg Public Offices | 1.51611e-09 | 0 | 0.00209088 | 0.0459079 | 0.0011219 | 0.0011219 | 0 | 0 | 0 | 0 | 0 | 0.0491206 |

Fig S5: Breakdown of Particulate Matter Formation Potential by Stakeholder Groups in Malang District.

kg of PM 2.5 Eq

|  | Rest (+) | Rest(-) | Other EoL | Informal Waste Collector | Formal Waste Collector | Open burning | Incineration | Recycling | Avoided plastic production | Sanitary Landfill | Transport | Score |
| --- | --- | --- | --- | --- | --- | --- | --- | --- | --- | --- | --- | --- |
| 1kg Formal Waste Collector | 0 | -9.26661e-12 | 0.00100589 | 0 | 0 | 0 | 0 | 0.000120754 | 0 | 0 | 7.27532e-05 | 0.0011994 |
| 1kg Formal Waste Picker | 2.1907e-10 | 0 | 0.00179718 | 0 | 0 | 0.000163737 | 0 | 0 | 0 | 0 | 6.95658e-05 | 0.00203049 |
| 1kg Household | 5.47945e-08 | -1.65122e-10 | 0 | 0.000637583 | 2.19538e-06 | 0.00785948 | 0 | 0 | 0 | 0 | 0 | 0.00849931 |
| 1kg Recycling Industry | 0 | -1.30104e-17 | 0 | 0 | 0 | 0 | 0 | 0.00591931 | -0.00404582 | 0 | 0 | 0.00187348 |
| 1kg Landfill | 0 | 0 | 0 | 0 | 0 | 0 | 0 | 0 | 0 | 2.33614e-05 | 0 | 2.33614e-05 |
| 1kg Industry | 8.06547e-08 | -9.52046e-11 | 4.09013e-05 | 0 | 0 | 0.00485634 | 2.67039e-05 | 0.00183498 | 0 | 1.57515e-06 | 0 | 0.00676058 |
| 1kg Informal Waste Collector | 0 | -1.08032e-10 | 0 | 0 | 0 | 3.81109e-05 | 0 | 0.00571213 | -0.00288197 | 2.33614e-05 | 0.000168707 | 0.00306034 |
| 1kg Informal Waste Pickers | 5.75108e-12 | -2.47382e-10 | 0.0139267 | 0 | 0 | 3.95426e-06 | 0 | 5.58782e-05 | 0 | 0 | 0.000422981 | 0.0144095 |
| 1kg Local Shop | 5.69683e-08 | 0 | 0.00247074 | 0 | 0 | 0.00350284 | 0 | 0 | 0 | 0 | 0 | 0.00597364 |
| 1kg Market | 5.39464e-11 | 0 | 0 | 0.00136295 | 1.2477e-05 | 0 | 0 | 0 | 0 | 0 | 0 | 0.00137543 |
| 1kg Public Facilities | 1.86648e-08 | -1.88247e-11 | 9.26821e-05 | 0.00130451 | 6.2019e-06 | 0.000535967 | 0 | 0 | 0 | 0 | 0 | 0.00193938 |
| 1kg Public Offices | 1.52645e-08 | 0 | 0.000115631 | 0.00123177 | 4.21509e-06 | 0.000291469 | 0 | 0 | 0 | 0 | 0 | 0.0016431 |

Table S37: Net Results of Current Scenario in Malang broken down into Stakeholder group.

| Stakeholder Group | Household | Industry | Formal Waste Picker | Informal Waste Collector | Formal Waste Collector | Informal Waste Pickers | Market | Landfill | Public Facilities | Public Offices | Recycling Industry | Local Shop |
| --- | --- | --- | --- | --- | --- | --- | --- | --- | --- | --- | --- | --- |
| Acidification: terrestrial \| terrestrial acidification potential (TAP) | 9.36E-04 | 6.17E-04 | 8.40E-04 | -4.24E-03 | 6.78E-04 | 1.91E-03 | 5.35E-04 | 4.87E-05 | 1.29E-03 | 8.69E-04 | -6.97E-03 | 1.78E-03 |
| Climate change \| global warming potential (GWP100) | 1.55E+00 | 1.04E+00 | 1.17E+00 | -1.71E+00 | 1.35E+00 | 1.84E+00 | 3.08E-01 | 1.07E-01 | 1.51E+00 | 1.06E+00 | -3.29E+00 | 2.97E+00 |
| Ecotoxicity: freshwater \| freshwater ecotoxicity potential (FETP) | 8.82E-02 | 1.04E-01 | 8.73E-02 | 1.48E-01 | 1.18E-01 | 1.34E-01 | 1.57E-01 | 1.72E-01 | 1.14E-01 | 1.02E-01 | -4.73E-02 | 1.54E-01 |
| Ecotoxicity: marine \| marine ecotoxicity potential (METP) | 1.31E-01 | 1.47E-01 | 1.29E-01 | 2.02E-01 | 1.71E-01 | 1.92E-01 | 2.19E-01 | 2.33E-01 | 1.67E-01 | 1.47E-01 | -6.23E-02 | 2.26E-01 |
| Ecotoxicity: terrestrial \| terrestrial ecotoxicity potential (TETP) | 1.62E+01 | 5.21E+00 | 1.34E+01 | -2.55E+00 | 1.38E+01 | 1.41E+01 | 1.24E+00 | 3.68E-02 | 1.77E+01 | 1.26E+01 | -4.85E+00 | 2.81E+01 |
| Energy resources: non-renewable, fossil \| fossil fuel potential (FFP) | 2.43E-02 | 4.00E-02 | 3.42E-02 | -1.09E+00 | 3.62E-02 | 1.39E-01 | 5.37E-02 | 5.71E-03 | 5.13E-02 | 4.91E-02 | -1.83E+00 | 1.42E-02 |
| Eutrophication: freshwater \| freshwater eutrophication potential (FEP) | 5.59E-05 | 1.58E-04 | 3.82E-05 | -2.93E-05 | 5.01E-05 | 8.78E-05 | 1.20E-04 | 1.25E-06 | 1.18E-04 | 1.12E-04 | -3.38E-04 | 3.29E-05 |
| Eutrophication: marine \| marine eutrophication potential (MEP) | 2.95E-04 | 1.49E-04 | 4.34E-04 | 1.74E-03 | 1.96E-04 | 5.97E-04 | 2.21E-04 | 1.81E-03 | 6.29E-04 | 3.58E-04 | -4.32E-05 | 7.89E-04 |
| Human toxicity: carcinogenic \| human toxicity potential (HTPc) | 1.63E-01 | 1.15E-01 | 5.37E-02 | -3.79E-01 | 5.08E-02 | 2.94E-01 | 7.47E-02 | 4.54E-03 | 7.77E-02 | 7.37E-02 | -5.68E-01 | 9.16E-02 |
| Human toxicity: non-carcinogenic \| human toxicity potential (HTPnc) | 1.93E+00 | 1.37E+00 | 1.76E+00 | 3.10E+00 | 2.70E+00 | 3.15E+00 | 3.03E+00 | 4.71E+00 | 2.14E+00 | 1.94E+00 | -2.33E+00 | 4.10E+00 |
| Ionising radiation \| ionising radiation potential (IRP) | 1.52E-03 | 1.88E-03 | 2.18E-03 | -6.69E-02 | 2.06E-03 | 8.94E-03 | 3.31E-03 | 2.30E-04 | 3.19E-03 | 3.06E-03 | -9.53E-02 | 8.36E-04 |
| Land use \| agricultural land occupation (LOP) | 3.68E-03 | 2.97E-03 | 3.60E-03 | -3.13E-02 | 4.52E-03 | 7.80E-03 | 7.53E-03 | 2.91E-03 | 7.19E-03 | 6.46E-03 | -4.73E-02 | 3.19E-03 |
| Material resources: metals/minerals \| surplus ore potential (SOP) | 3.24E-03 | 6.37E-03 | 2.92E-03 | -1.30E-02 | 4.02E-03 | 9.24E-03 | 6.99E-03 | 2.12E-04 | 6.90E-03 | 6.53E-03 | -2.79E-02 | 2.09E-03 |
| Ozone depletion \| ozone depletion potential (ODPinfinite) | 6.42E-07 | 2.03E-07 | 4.75E-07 | -2.56E-07 | 4.81E-07 | 6.77E-07 | 5.99E-08 | 1.21E-08 | 6.16E-07 | 4.15E-07 | -4.83E-07 | 1.23E-06 |
| Particulate matter formation \| particulate matter formation potential (PMFP) | 8.50E-03 | 6.76E-03 | 2.03E-03 | 3.06E-03 | 1.20E-03 | 1.44E-02 | 1.38E-03 | 2.34E-05 | 1.94E-03 | 1.64E-03 | 1.87E-03 | 5.97E-03 |
| Photochemical oxidant formation: human health \| photochemical oxidant formation potential: humans (HOFP) | 1.91E-03 | 7.82E-04 | 1.95E-03 | -2.70E-03 | 1.14E-03 | 4.59E-03 | 6.23E-04 | 8.22E-05 | 2.55E-03 | 1.48E-03 | -5.81E-03 | 4.00E-03 |
| Photochemical oxidant formation: terrestrial ecosystems \| photochemical oxidant formation potential: ecosystems (EOFP) | 1.92E-03 | 7.96E-04 | 2.11E-03 | -3.06E-03 | 1.16E-03 | 5.51E-03 | 6.49E-04 | 8.59E-05 | 2.58E-03 | 1.51E-03 | -6.75E-03 | 4.01E-03 |
| Water use \| water consumption potential (WCP) | 8.01E-04 | 3.05E-03 | 6.69E-04 | -1.43E-02 | 7.54E-04 | 1.54E-03 | 8.29E-04 | 3.00E-05 | 1.18E-03 | 1.05E-03 | -2.03E-02 | 1.04E-03 |

# 4. Surveys

# 5. Types of Plastic Collected

Table S38: Types of Plastic in Villages

| Types of Waste Plastic | HDPE | LDPE | PP | PET | PVC | PS | EPS | OTHER | Total |
| --- | --- | --- | --- | --- | --- | --- | --- | --- | --- |
| Bakalan | 83.067 | 158.111 | 43.786 | 79.481 | 33.775 | 29.349 | 15.945 | 1.595 | 454.473 |
| Unit | kg | kg | kg | kg | kg | kg | kg | kg | kg |
| Bedali | 0 | 0 | 20 | 0 | 0 | 0 | 0 | 0 | 20 |
| Unit | Kg | Kg | Kg | Kg | Kg | Kg | Kg | Kg | Kg |
| Gampingan | 480 | 589 | 3509 | 3092 | 11 | 10 | 20 | 23 | 36670 |
| Unit | Kg | Kg | Kg | Kg | Kg | Kg | Kg | Kg | Kg |
| Krebet Senggrong | 26.467 | 77.054 | 41.47 | 66.003 | 9.602 | 11.808 | 9.555 | 2.655 | 245.214 |
| Unit | kg | kg | kg | kg | kg | kg | kg | kg | kg |
| Krebet | 201.297 | 745.049 | 224.749 | 347.825 | 61.92 | 39.223 | 130.499 | 0 | 1749.516 |
| Unit | kg | kg | kg | kg | kg | kg | kg | kg | kg |
| Mangunrejo | 0.219 | 0.867 | 3501.803 | 0 | 0 | 0 | 0 | 0 | 3502.583 |
| Unit | Kg | Kg | Kg | Kg | Kg | Kg | Kg | Kg | Kg |
| Sumberejo | 60.256 | 1190.052 | 95.07 | 1258.34 | 65.909 | 90.617 | 64.939 | 142.179 | 2967.056 |
| Unit | kg | kg | kg | kg | kg | kg | kg | kg | kg |
| Rekasan |  | 250000 |  |  |  |  |  |  | 250000 |
| Unit | kg | kg | kg | kg | kg | kg | kg | kg | kg |
| Tegalsari | 2 | 0 | 4 | 0 | 0 | 0 | 0 | 10 | 16 |
| Unit | kg | kg | kg | kg | kg | kg | kg | kg | kg |
| Tlogorejo | 189.408 | 207.127 | 74.622 | 240.419 | 2.24 | 115.736 | 36.615 | 1.035 | 880.987 |
| Unit | kg | kg | kg | kg | kg | kg | kg | kg | 0 |
| Pagak | 11.45 | 69.246 | 37.245 | 33.978 | 0.86 | 30.692 | 2.405 | 0 | 185.876 |
| Unit | kg | kg | kg | kg | kg | kg | kg | kg | kg |
